# Supplementary material for: Screening for Small Molecule Modulators of Trypanosoma brucei Hsp70 Chaperone Activity Based upon Alcyonarian Coral-Derived Natural Products
Source: Mar Drugs. 2020 Jan 27;18(2):81. doi: 10.3390/md18020081 (PMC7074166; doi:10.3390/md18020081)
Supplement: Supplementary file 1 [file marinedrugs-18-00081-s001.pdf]

marine drugs

MDPI

Supplementary Materials for

Screening for small molecule modulators of *Trypanosoma brucei* Hsp70 chaperone activity based upon alcyonarian coral-derived natural products

Sarah K. Andreassend [1,2 †], Stephen J. Bentley [3 †], Gregory L. Blatch [4,5], Aileen Boshoff [3] and Robert A. Keyzers [1,2*]

[1] School of Chemical and Physical Sciences, Victoria University of Wellington, Wellington, New Zealand.
[2] Centre for Biodiscovery, Victoria University of Wellington, Wellington, New Zealand.
[3] Biotechnology Innovation Centre, Rhodes University, Grahamstown, South Africa.
[4] Biomedical Biotechnology Research Unit, Department of Biochemistry and Microbiology, Grahamstown, South Africa.
[5] Office of the PVC Research, The University of Notre Dame Australia, Fremantle, WA, Australia.
[*] Correspondence: robert.keyzers@vuw.ac.nz; Tel.: (+64)-4-463-5117 (R.A.K.).
[†] These authors contributed equally to this work.

Table of Contents

Figure S1. Assessment of the anti-trypanosomal activity of the compounds S6
Table S1. IC_{50} values and structures of 48 select compounds S7
Figure S2. Evaluation of the cytotoxicity on mammalian cells of the compounds........ S11
Figure S3. The solubility of Human HSPA8 after heat exposure S11
Figure S4. Investigation of the modulatory effect of the small molecules on the thermally induced aggregation of MDH S12
Figure S5. Purification of the recombinant trypanosomal and human heat shock proteins. S12
Figure S6. Preliminary screening of the effects of the compounds on the basal ATPase activities of the TbHsp70s and HsHSPA8 .... S13
Figure S7. Preliminary screening of the effects of the compounds on the J-stimulated ATPase activities of the TbHsp70s and HsHSPA8 .... S14
Figure S8. ^1H NMR spectrum (500 MHz, $CDCl_3$) of **4** S15
Figure S9. ^{13}C NMR spectrum (150 MHz, $CDCl_3$) of **4**........ S15
Figure S10. ^1H NMR spectrum (600 MHz, $CDCl_3$) of **5** S16
Figure S11. ^{13}C NMR spectrum (150 MHz, $CDCl_3$) of **5**........ S16
Figure S12. HMBC spectrum ($CDCl_3$) of **5** S17
Figure S13. ^1H NMR spectrum (600 MHz, $CDCl_3$) of **6** S18
Figure S14. ^{13}C NMR spectrum (150 MHz, $CDCl_3$) of **6**........ S18
Figure S15. ^1H NMR spectrum (500 MHz, $CDCl_3$) of **7** S19
Figure S16. ^{13}C NMR spectrum (150 MHz, $CDCl_3$) of **7**........ S19
Figure S17. ^1H NMR spectrum (500 MHz, $CDCl_3$) of **8** S20
Figure S18. ^{13}C NMR spectrum (150 MHz, $CDCl_3$) of **8**........ S20
Figure S19. ^1H NMR spectrum (600 MHz, $CDCl_3$) of **9** S21
Figure S20. ^{13}C NMR spectrum (150 MHz, $CDCl_3$) of **9**........ S21
Figure S21. ^1H NMR spectrum (600 MHz, $CDCl_3$) of **10** S22
Figure S22. ^{13}C NMR spectrum (150 MHz, $CDCl_3$) of **10**........ S22
Figure S23. ^1H NMR spectrum (500 MHz, $CDCl_3$) of **11** S23
Figure S24. ^{13}C NMR spectrum (150 MHz, $CDCl_3$) of **11**........ S23
Figure S25. ^1H NMR spectrum (600 MHz, $CDCl_3$) of **12** S24
Figure S26. ^{13}C NMR spectrum (150 MHz, $CDCl_3$) of **12**........ S24
Figure S27. ^1H NMR spectrum (500 MHz, $CDCl_3$) of **13** S25
Figure S28. ^{13}C NMR spectrum (150 MHz, $CDCl_3$) of **13**........ S25
Figure S29. ^1H NMR spectrum (500 MHz, $CDCl_3$) of **14** S26
Figure S30. ^{13}C NMR spectrum (150 MHz, $CDCl_3$) of **14**........ S26
Figure S31. ^1H NMR spectrum (300 MHz, $CDCl_3$) of **15** S27
Figure S32. ^{13}C NMR spectrum (150 MHz, $CDCl_3$) of **15**........ S27
Figure S33. ^1H NMR spectrum (300 MHz, $CDCl_3$) of **16** S28
Figure S34. ^{13}C NMR spectrum (150 MHz, $CDCl_3$) of **16**........ S28
Figure S35. ^1H NMR spectrum (600 MHz, $CDCl_3$) of **17** S29
Figure S36. ^{13}C NMR spectrum (150 MHz, $CDCl_3$) of **17**........ S29

Figure S37. ^{1}H NMR spectrum (600 MHz, $CDCl_3$) of **18** S30
Figure S38. ^{13}C NMR spectrum (150 MHz, $CDCl_3$) of **18**........ S30
Figure S39. ^{1}H NMR spectrum (500 MHz, $CDCl_3$) of **19** S31
Figure S40. ^{13}C NMR spectrum (150 MHz, $CDCl_3$) of **19**........ S31
Figure S41. ^{1}H NMR spectrum (500 MHz, $CDCl_3$) of **20** S32
Figure S42. ^{13}C NMR spectrum (150 MHz, $CDCl_3$) of **20**........ S32
Figure S43. ^{1}H NMR spectrum (500MHz, $CDCl_3$) of **21** S33
Figure S44. ^{13}C NMR spectra (150 MHz, $CDCl_3$) of **21**........ S33
Figure S45. ^{1}H NMR spectrum (600 MHz, $CDCl_3$) of **27** S34
Figure S46. ^{13}C NMR spectrum (150 MHz, $CDCl_3$) of **27**........ S34
Figure S47. ^{1}H NMR spectrum (600 MHz, $CDCl_3$) of **28** S35
Figure S48. ^{13}C NMR spectrum (150 MHz, $CDCl_3$) of **28**........ S35
Figure S49. ^{1}H NMR spectrum (600 MHz, $CDCl_3$) of **29** S36
Figure S50. ^{13}C NMR spectrum (150 MHz, $CDCl_3$) of **29**........ S36
Figure S51. ^{1}H NMR spectrum (600 MHz, $CDCl_3$) of **30** S37
Figure S52. ^{13}C NMR spectrum (150 MHz, $CDCl_3$) of **30**........ S37
Figure S53. ^{1}H NMR spectrum (600 MHz, $CDCl_3$) of **31** S38
Figure S54. ^{13}C NMR spectrum (150 MHz, $CDCl_3$) of **31**........ S38
Figure S55. ^{1}H NMR spectrum (600 MHz, $CDCl_3$) of **32** S39
Figure S56. ^{13}C NMR spectrum (150 MHz, $CDCl_3$) of **32**........ S39
Figure S57. ^{1}H NMR spectrum (500 MHz, $CDCl_3$) of **33** S40
Figure S58. ^{13}C NMR spectrum (150 MHz, $CDCl_3$) of **33**........ S40
Figure S59. ^{1}H NMR spectrum (600 MHz, $CDCl_3$) of **34** S41
Figure S60. ^{13}C NMR spectrum (150 MHz, $CDCl_3$) of **34**........ S41
Figure S61. ^{1}H NMR spectrum (600 MHz, $CDCl_3$) of **35** S42
Figure S62. ^{13}C NMR spectrum (150 MHz, $CDCl_3$) of **35**........ S42
Figure S63. ^{1}H NMR spectrum (500 MHz, $CDCl_3$) of **36** S43
Figure S64. ^{13}C NMR spectrum (150 MHz, $CDCl_3$) of **36**........ S43
Figure S65. ^{1}H NMR spectrum (500 MHz, $CDCl_3$) of **37** S44
Figure S66. ^{13}C NMR spectrum (150 MHz, $CDCl_3$) of **37**........ S44
Figure S67. ^{1}H NMR spectrum (600 MHz, $CDCl_3$) of **38** S45
Figure S68. ^{13}C NMR spectrum (150 MHz, $CDCl_3$) of **38**........ S45
Figure S69. ^{1}H NMR spectrum (600 MHz, $CDCl_3$) of **39** S46
Figure S70. ^{13}C NMR spectrum (150 MHz, $CDCl_3$) of **39**........ S46
Figure S71. ^{1}H NMR spectrum (500 MHz, $CDCl_3$) of **40** S47
Figure S72. ^{13}C NMR spectrum (150 MHz, $CDCl_3$) of **40**........ S47
Figure S73. ^{1}H NMR spectrum (500 MHz, $CDCl_3$) of **41** S48
Figure S74. ^{13}C NMR spectrum (150 MHz, $CDCl_3$) of **41**........ S48
Figure S75. ^{1}H NMR spectrum (600 MHz, $CDCl_3$) of **42** S49
Figure S76. ^{13}C NMR spectrum (150 MHz, $CDCl_3$) of **42**........ S49
Figure S77. ^{1}H NMR spectrum (600 MHz, $CDCl_3$) of **43** S50
Figure S78. ^{13}C NMR spectrum (150 MHz, $CDCl_3$) of **43**........ S50

Figure S79. ^{1}H NMR spectrum (600 MHz, $CDCl_3$) of **44** S51
Figure S80. ^{13}C NMR spectrum (150 MHz, $CDCl_3$) of **44**.......... S51
Figure S81. ^{1}H NMR spectrum (600 MHz, $CDCl_3$) of **46** S52
Figure S82. ^{13}C NMR spectrum (150 MHz, $CDCl_3$) of **46**.......... S52
Figure S83. ^{1}H NMR spectrum (500 MHz, $CDCl_3$) of **47** S53
Figure S84. ^{13}C NMR spectrum (150 MHz, $CDCl_3$) of **47**.......... S53
Figure S85. ^{1}H NMR spectrum (500 MHz, $CDCl_3$) of **48** S54
Figure S86. ^{13}C NMR spectrum (150 MHz, $CDCl_3$) of **48**.......... S54
Figure S87. ^{1}H NMR spectrum (600 MHz, $CDCl_3$) of **49** S55
Figure S88. ^{13}C NMR spectrum (150 MHz, $CDCl_3$) of **49**.......... S55
Figure S89. ^{1}H NMR spectrum (150 MHz, $CDCl_3$) of **50** S56
Figure S90. ^{13}C NMR spectrum (150 MHz, $CDCl_3$) of **50**.......... S56
Figure S91. ^{1}H NMR spectrum (500 MHz, $CDCl_3$) of **54** S57
Figure S92. ^{13}C NMR spectrum (150 MHz, $CDCl_3$) of **54**.......... S57
Figure S93. ^{1}H NMR spectrum (600 MHz, $CDCl_3$) of **55** S58
Figure S94. ^{13}C NMR spectrum (150 MHz, $CDCl_3$) of **55**.......... S58
Figure S95. ^{1}H NMR spectrum (500 MHz, $CDCl_3$) of **56** S59
Figure S96. ^{13}C NMR spectrum (150 MHz, $CDCl_3$) of **56**.......... S59
Figure S97. ^{1}H NMR spectrum (600 MHz, $CDCl_3$) of **57** S60
Figure S98. ^{13}C NMR spectrum (150 MHz, $CDCl_3$) of **57**.......... S60
Figure S99. ^{1}H NMR spectrum (600 MHz, $CDCl_3$) of **58** S61
Figure S100. ^{13}C NMR spectrum (150 MHz, $CDCl_3$) of **58**.......... S61
Figure S101. ^{1}H NMR spectrum (500 MHz, $CDCl_3$) of **59** S62
Figure S102. ^{13}C NMR spectrum (150 MHz, $CDCl_3$) of **59**.......... S62
Figure S103. ^{1}H NMR spectrum (500 MHz, $CDCl_3$) of **60** S63
Figure S104. ^{13}C NMR spectrum (150 MHz, $CDCl_3$) of **60**.......... S63
Figure S105. ^{1}H NMR spectrum (600 MHz, $CDCl_3$) of **61** S64
Figure S106. ^{13}C NMR spectrum (150 MHz, $CDCl_3$) of **61**.......... S64
Figure S107. ^{1}H NMR spectrum (600 MHz, $CDCl_3$) of **62** S65
Figure S108. ^{13}C NMR spectrum (150 MHz, $CDCl_3$) of **62**.......... S65
Figure S109. ^{1}H NMR spectrum (600 MHz, $CDCl_3$) of **63** S66
Figure S110. ^{13}C NMR spectrum (150 MHz, $CDCl_3$) of **63**.......... S66
Figure S111. ^{1}H NMR spectrum (600 MHz, $CDCl_3$) of **64** S67
Figure S112. ^{13}C NMR spectrum (150 MHz, $CDCl_3$) of **64**.......... S67
Figure S113. ^{1}H NMR spectrum (600 MHz, $CDCl_3$) of **67** S68
Figure S114. ^{13}C NMR spectrum (150 MHz, $CDCl_3$) of **67**.......... S68
Figure S115. ^{1}H NMR spectrum (500 MHz, $CDCl_3$) of **68** S69
Figure S116. ^{13}C NMR spectrum (150 MHz, $CDCl_3$) of **68**.......... S69
Figure S117. ^{1}H NMR spectrum (500 MHz, $CDCl_3$) of **69** S70
Figure S118. ^{13}C NMR spectrum (150 MHz, $CDCl_3$) of **69**.......... S70
Figure S119. ^{1}H NMR spectrum (600 MHz, $CDCl_3$) of **70** S71
Figure S120. ^{13}C NMR spectrum (150 MHz, $CDCl_3$) of **70**.......... S71

Figure S121. ^{1}H NMR spectrum (600 MHz, $CDCl_3$) of **71** S72
Figure S122. ^{13}C NMR spectrum (150 MHz, $CDCl_3$) of **71** S72
Figure S123. ^{1}H NMR spectrum (150 MHz, $CDCl_3$) of **72** S73
Figure S124. ^{13}C NMR spectrum (150 MHz, $CDCl_3$) of **72** S73
Figure S125. ^{1}H NMR spectrum (300 MHz, $CDCl_3$) of **73** S74
Figure S126. ^{13}C NMR spectrum (150 MHz, $CDCl_3$) of **73** S74
Figure S127. ^{1}H NMR spectrum (300 MHz, $CDCl_3$) of **74** S75
Figure S128. ^{13}C NMR spectrum (150 MHz, $CDCl_3$) of **74** S75
Figure S129. ^{1}H NMR spectrum (500 MHz, $CDCl_3$) of **75** S76
Figure S130. ^{13}C NMR spectrum (150 MHz, $CDCl_3$) of **75** S76
Figure S131. ^{13}C NMR spectrum (300 MHz, $CDCl_3$) of **76** S77
Figure S132. ^{13}C NMR spectrum (150 MHz, $CDCl_3$) of **76** S77
Figure S133. ^{1}H NMR spectrum (300 MHz, $CDCl_3$) of **77** S78
Figure S134. ^{13}C NMR spectrum (150 MHz, $CDCl_3$) of **77** S78
Figure S135. ^{1}H NMR spectrum (500 MHz, $CDCl_3$) of **78** S79
Figure S136. ^{13}C NMR spectrum (150 MHz, $CDCl_3$) of **78** S79
Figure S137. ^{1}H NMR spectrum (500 MHz, $CDCl_3$) of **79** S80
Figure S138. ^{13}C NMR spectrum (150 MHz, $CDCl_3$) of **79** S80
Figure S139. ^{1}H NMR spectrum (500 MHz, $CDCl_3$) of **80** S81
Figure S140. ^{13}C NMR spectrum (150 MHz, $CDCl_3$) of **80** S81
Figure S141. ^{1}H NMR spectrum (500 MHz, $CDCl_3$) of **81** S82
Figure S142. ^{13}C NMR spectrum (150 MHz, $CDCl_3$) of **81** S82
Figure S143. ^{1}H NMR spectrum (500 MHz, $CDCl_3$) of **82** S83
Figure S144. ^{13}C NMR spectrum (150 MHz, $CDCl_3$) of **82** S83
Figure S145. ^{1}H NMR spectrum (500 MHz, $CDCl_3$) of **83** S84
Figure S146. ^{13}C NMR spectrum (150 MHz, $CDCl_3$) of **83** S84
Figure S147. ^{1}H NMR spectrum (300 MHz, $CDCl_3$) of **84** S85
Figure S148. ^{13}C NMR spectrum (150 MHz, $CDCl_3$) of **84** S85
Figure S149. ^{1}H NMR spectrum (300 MHz, $CDCl_3$) of **85** S86
Figure S150. ^{13}C NMR spectrum (150 MHz, $CDCl_3$) of **85** S86
Figure S151. ^{1}H NMR spectrum (500 MHz, $CDCl_3$) of **86** S87
Figure S152. ^{13}C NMR spectrum (150 MHz, $CDCl_3$) of **86** S87
Figure S153. ^{1}H NMR spectrum (500 MHz, $CDCl_3$) of **87** S88
Figure S154. ^{13}C NMR spectrum (150 MHz, $CDCl_3$) of **87** S88
Figure S155. ^{1}H NMR spectrum (500 MHz, $CDCl_3$) of **88** S89
Figure S156. ^{13}C NMR spectrum (150 MHz, $CDCl_3$) of **88** S89

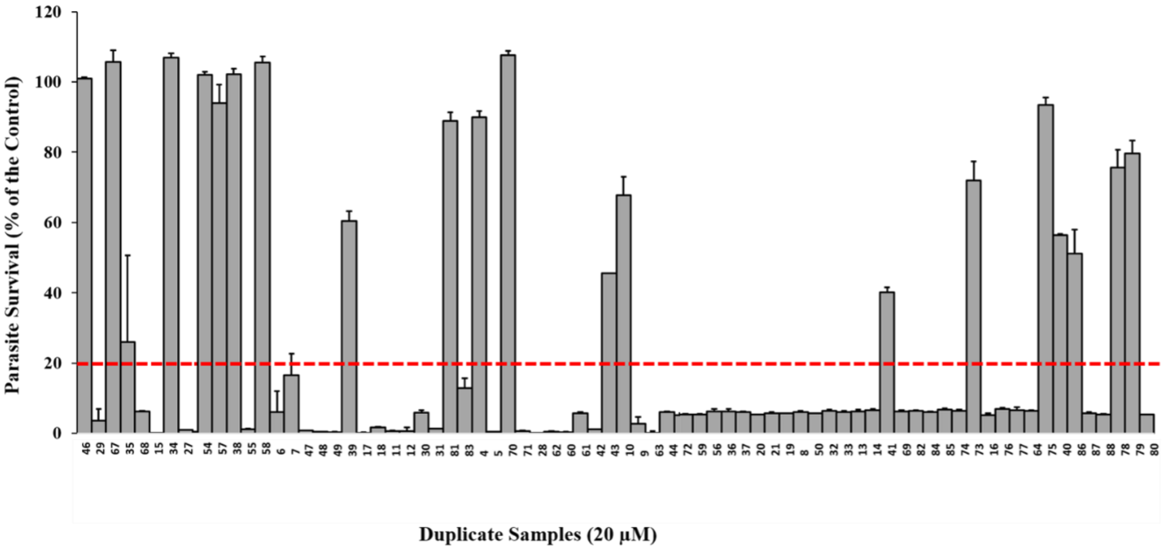

Figure S1. Assessment of the anti-trypanosomal activity of the compounds. The compounds were added to *in vitro* cultures of bloodstream stage *T. b. brucei* Lister 427 221 strain parasites at a fixed concentration of 20 μM and incubated for 48 hours. Data represented in the graph are the percentage of parasite survival in relation to untreated parasites after the 48-hour treatment measured using a resazurin-based cytotoxicity assay and the resorufin fluorescence was quantified ($Excitation_{560}$/$Emission_{590}$). The red dashed line highlights the compounds which reduced *in vitro* parasite growth to ≥ 80%. Data represent two replicates and error bars indicating standard deviation.

Table S1. Determined IC$_{50}$ values of 48 select compounds shown to be anti-trypanosomal in single concentration assays (<20% viable after treatment at 20 μM, Figure S1).

Compound	E/Z	Structure	IC$_{50}$/ μM
**6**	3:2	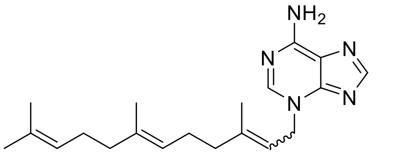	12.74
**7**	3:2	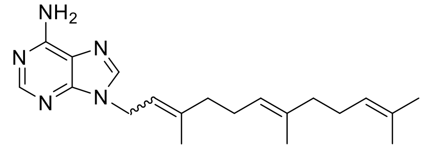	47.69
**8**	-	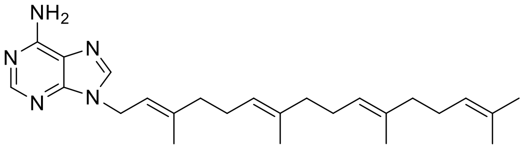	13.39
**9**	-	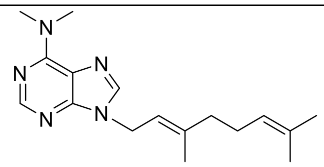	28.43
**11**	3:2	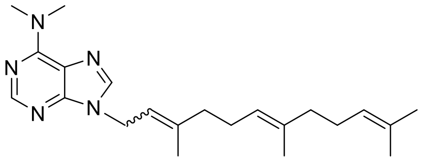	9.48
**12**	3:2	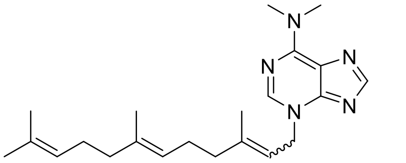	14.00
**13**	-	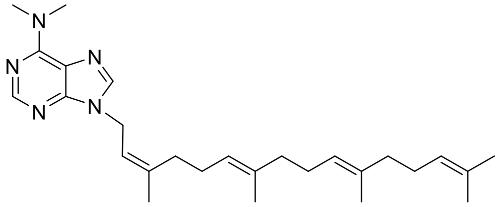	8.69
**14**	-	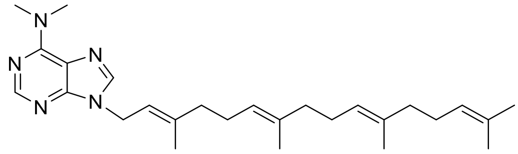	9.22
**15**	-	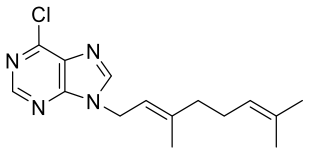	12.17
**16**	-	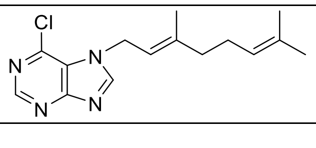	11.03

**17**	2:1	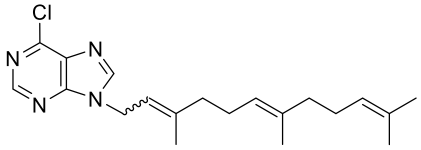	12.91
**18**	3:2	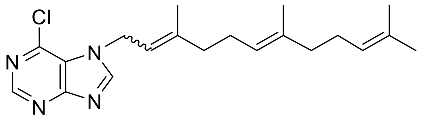	9.28
**19**	-	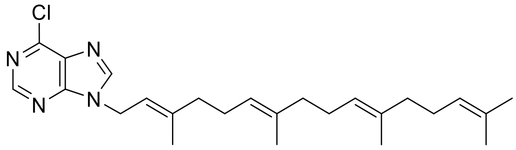	6.79
**20**	-	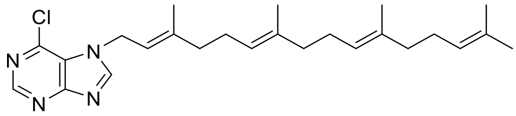	10.96
**21**	-	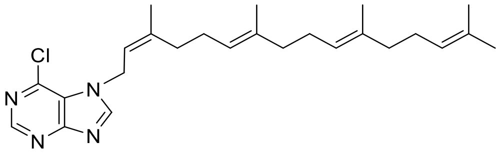	19.09
**27**	-	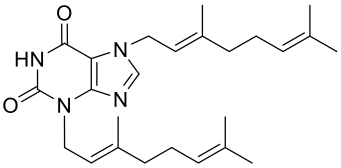	2.00
**28**	3:2	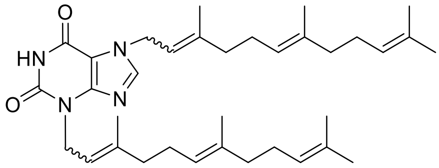	1.15
**29**	-	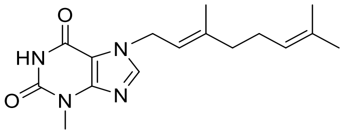	18.85
**30**	-	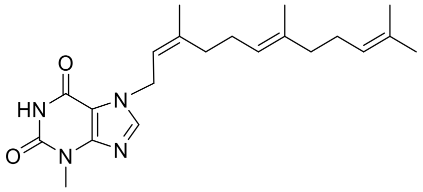	4.62
**31**	2:1	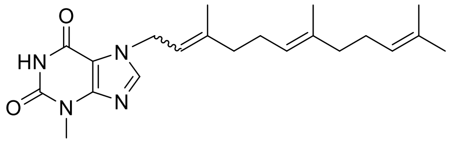	1.91
**32**	-	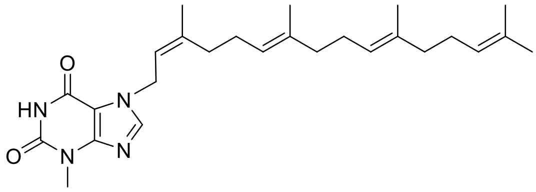	5.90

**33**	-	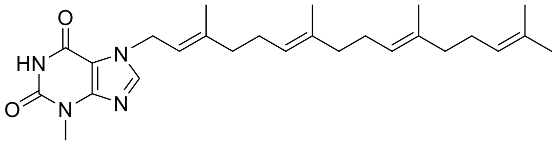	10.21
**36**	-	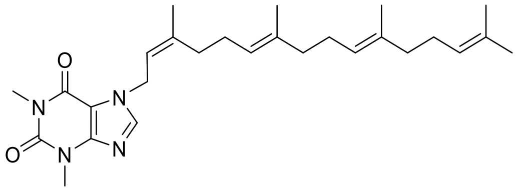	5.17
**37**	-	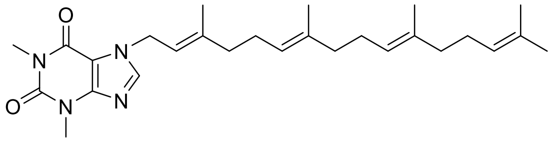	21.60
**42**	-	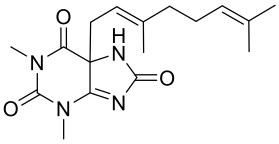	10.91
**44**	3:2	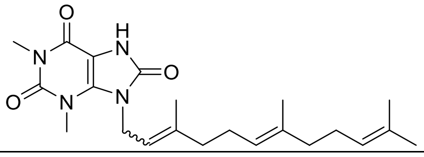	16.64
**47**	2:1	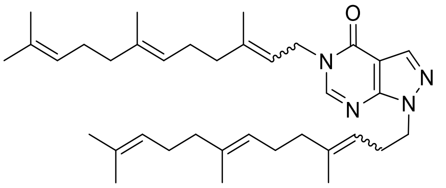	1.23
**48**	3:2	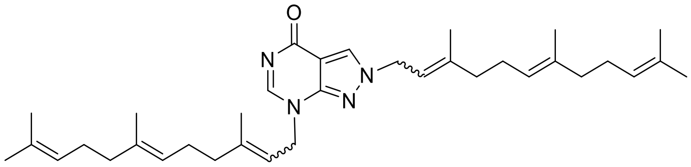	5.47
**49**	3:2	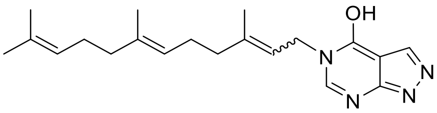	16.67
**50**	-	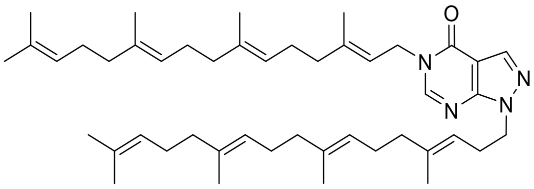	2.07
**55**	2:1	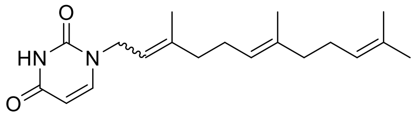	16.49
**56**	-	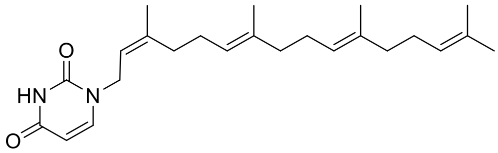	6.20

**59**	-	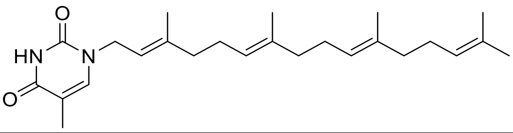	15.80
**60**	-	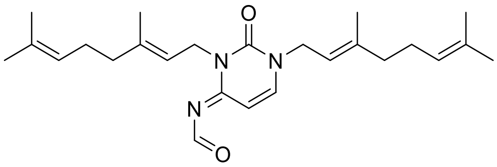	4.89
**63**	3:2	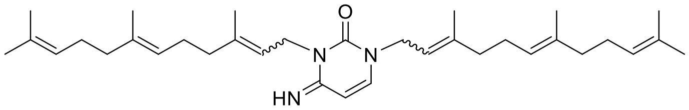	2.05
**68**	5:4	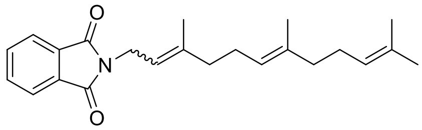	32.22
**69**	-	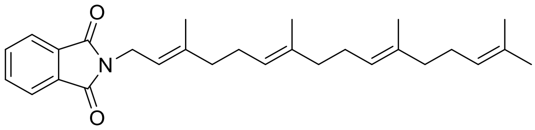	9.16
**71**	7:2	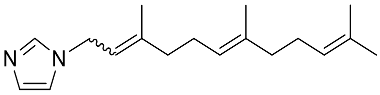	26.82
**72**	-	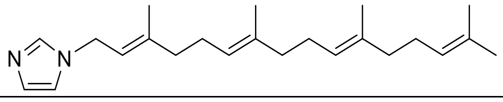	3.42
**74**	-	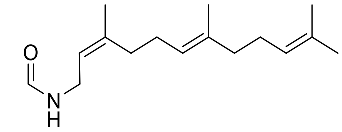	27.28
**76**	-	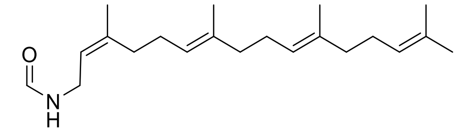	11.03
**77**	-	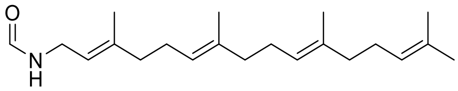	41.77
**80**	-	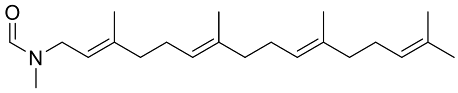	41.21
**82**	-	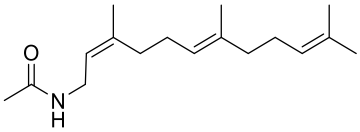	4.49
**83**	-	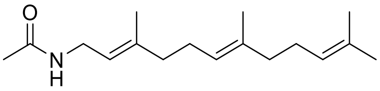	21.47
**84**	-	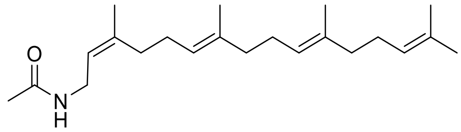	2.17
**85**	-	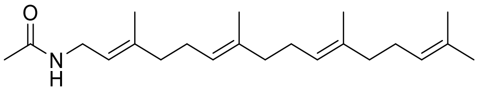	3.14

88	-	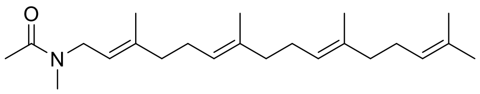	38.40

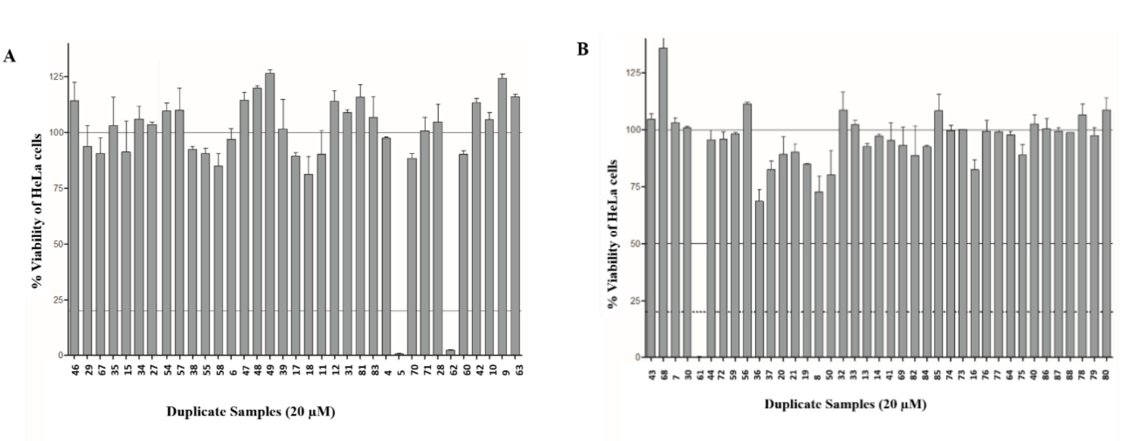

Figure S2. Evaluation of the cytotoxicity on mammalian cells of the compounds (A and B). The compounds were added to *in vitro* cultures of HeLa cells at a fixed concentration of 20 µM and incubated for 48 hours. Data represented in the graph are the percentage of cell survival in relation to untreated cells after the 48-hour treatment measured using a resazurin-based cytotoxicity assay and the resorufin fluorescence was quantified (Excitation$_{560}$/Emission$_{590}$). Data represent two replicates and error bars indicating standard deviation.

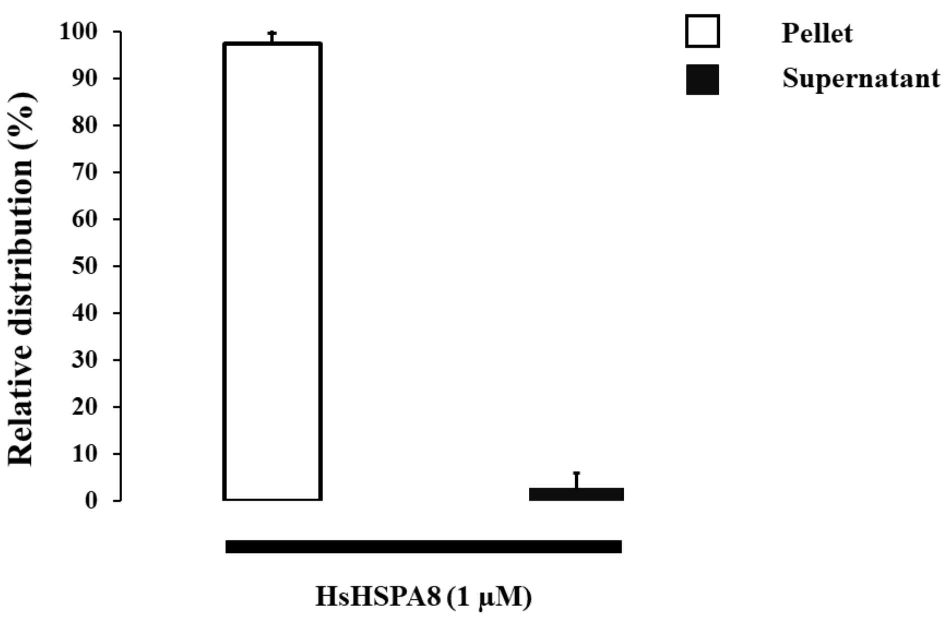

Figure S3. HsHSPA8 is prone to self-aggregation. The solubility of Human HSPA8 (1 µM) was monitored by incubation of the chaperone *in vitro* at 48 ºC for 1 hour and quantifying the pellet (insoluble; white bars) and supernatant (soluble; black bars) fractions after heat exposure. Standard deviations obtained from three replicate assays on three independent batches of recombinant protein.

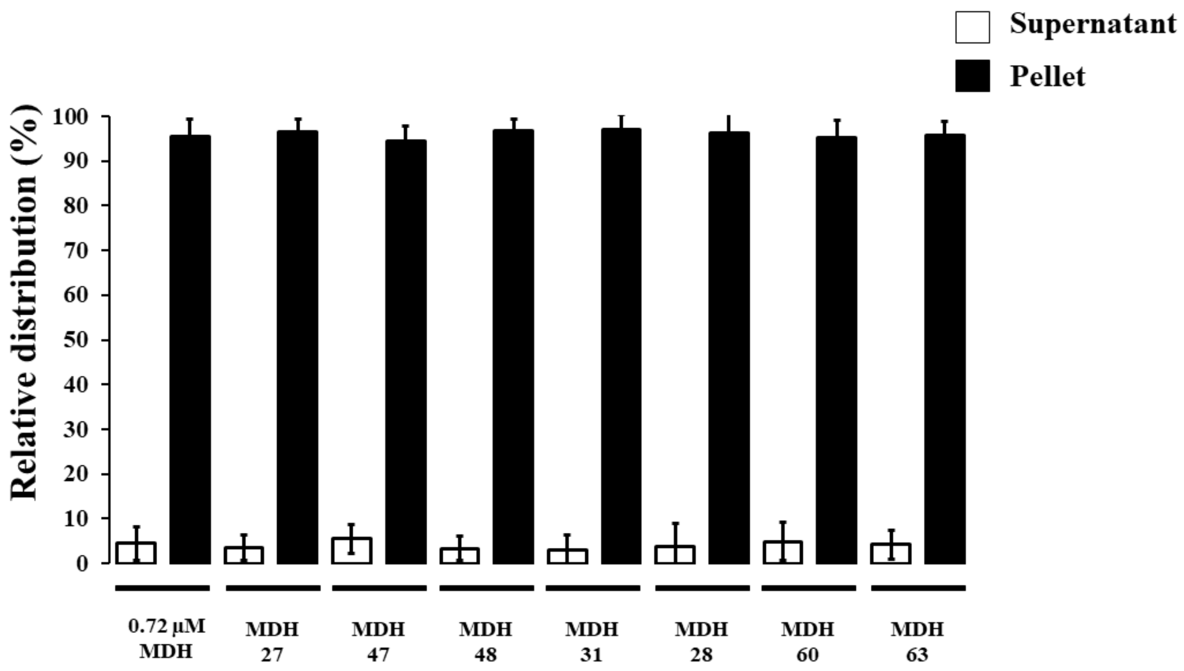

Figure S4. The compounds do not suppress the aggregation of MDH. Investigation of the modulatory effect of the small molecules on the thermally induced aggregation of MDH was conducted by monitoring the heat-induced aggregation of MDH (in the presence and absence of 300 µM small molecules) *in vitro* at 48 °C and quantitating the pellet (insoluble; black bars) and supernatant (soluble; white bars) fractions after heat exposure. Standard deviations obtained from three replicate assays on three independent experiments.

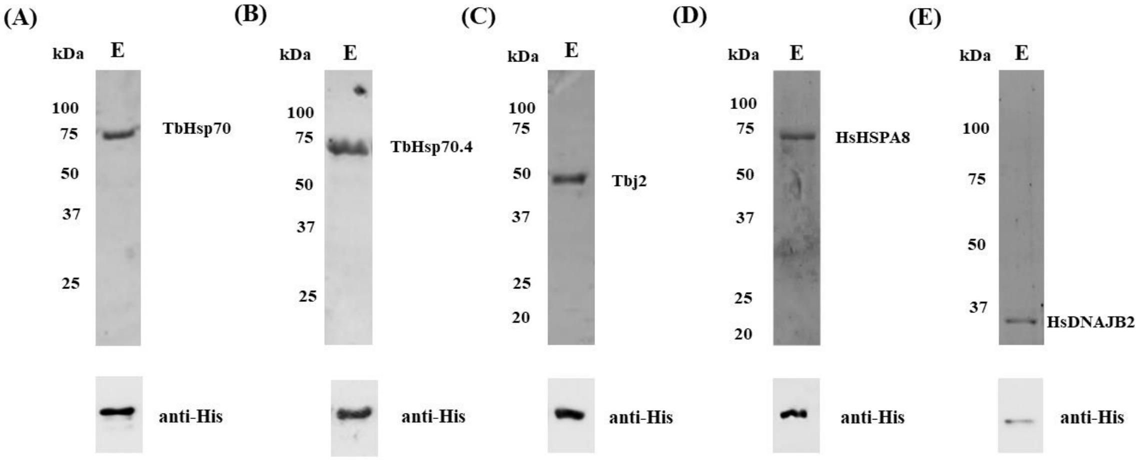

Figure S5. Purification of the recombinant trypanosomal and human heat shock proteins. SDS-PAGE (10%) and western blot images representing the purification of the recombinant forms of TbHsp70 (A), TbHsp70.4 (B), Tbj2 (C), HsHSPA8 (D) and HsDNAJB2 (E). *Lanes E*: Recombinant proteins eluted from the affinity matrix using 500 mM imidazole. *Lower panels*: Western analysis using anti-His antibody to purification of recombinant proteins. Marker in kilodalton (kDa) (Precision Plus Protein™ All Blue Prestained Protein Standard) is shown on the left-hand side.

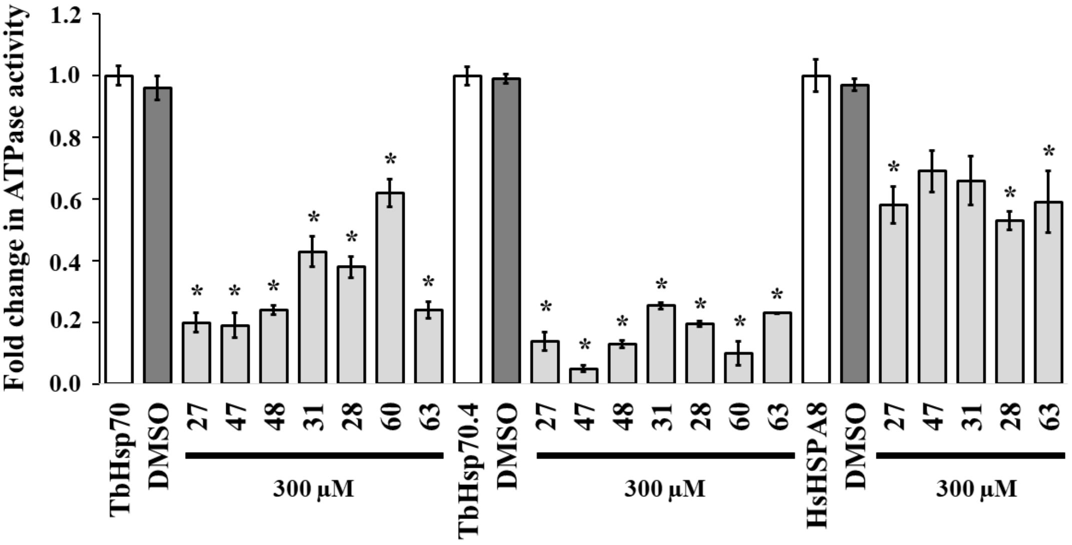

Figure S6. Preliminary screening of the effects of the compounds on the basal ATPase activities of the TbHsp70s and HsHSPA8. TbHsp70 (0.8 μM), TbHsp70.4 (0.8 μM) and HsHSPA8 (0.8 μM) alone and in the presence of 1% (v/v) DMSO or selected compounds at 300 μM were incubated with 1 mM ATP for 1 h, and the released Pi was monitored at 595 nm using a direct colorimetric assay. Results are represented as fold change in the untreated ATPase activity of the Hsp70s (black bar) in relation to the ATPase activity of the Hsp70s in the presence of compounds at 300 μM or 1% (v/v) DMSO (grey bars). Standard deviations were obtained from two replicate assays are shown on three independent batches of proteins. Significant differences relative to the no "small molecule" reaction (Hsp70; black bar) are indicated by * ($P < 0.05$) above the reaction using a Student's t-test.

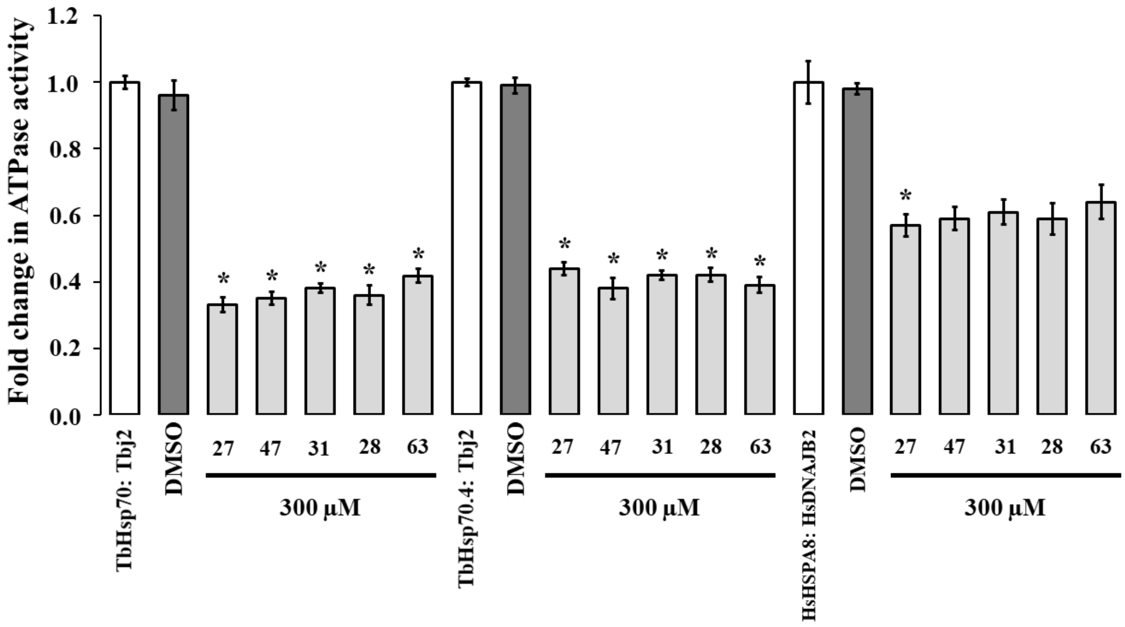

Figure S7. Preliminary screening of the effects of the compounds on the J-stimulated ATPase activities of the TbHsp70s and HsHSPA8. Recombinant J-proteins (0.4 µM) and Hsp70 proteins (0.8 µM) alone and in the presence of the compounds at 300 µM or 1% DMSO (v/v) were incubated with 1 mM ATP for 1 h, and the released Pi was monitored at 595 nm using a direct colorimetric assay. Results are represented as fold change in the untreated J-stimulated ATPase activity of the Hsp70s (white bar) in relation to the J-stimulated ATPase activity of the Hsp70s in the presence of compounds at 300 µM (light grey bars) or 1% (v/v) DMSO (dark grey bar). Standard deviations were obtained from two replicate assays are shown on three independent batches of proteins. Significant differences relative to the no "small molecule" reaction (Hsp70; white bar) are indicated by * ($P < 0.05$) above the reaction using a Student's t-test.

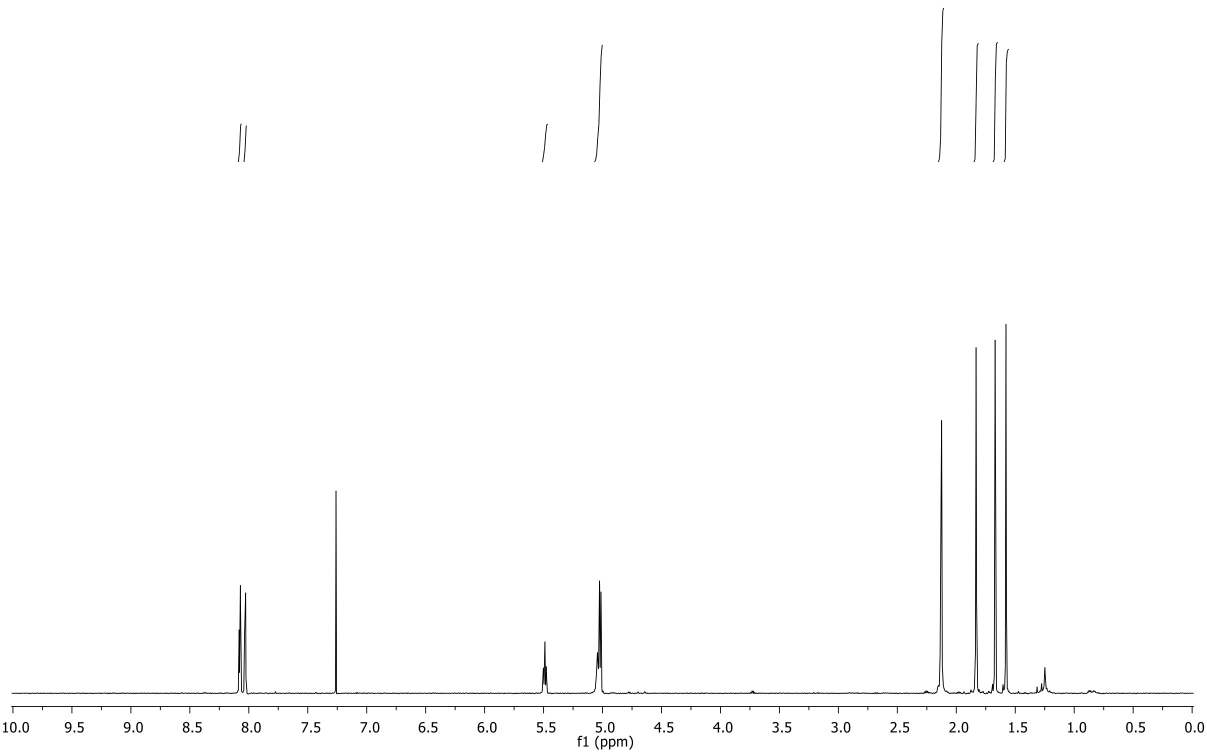

Figure S8. ^{1}H NMR spectrum (500 MHz, $CDCl_3$) of **4**.

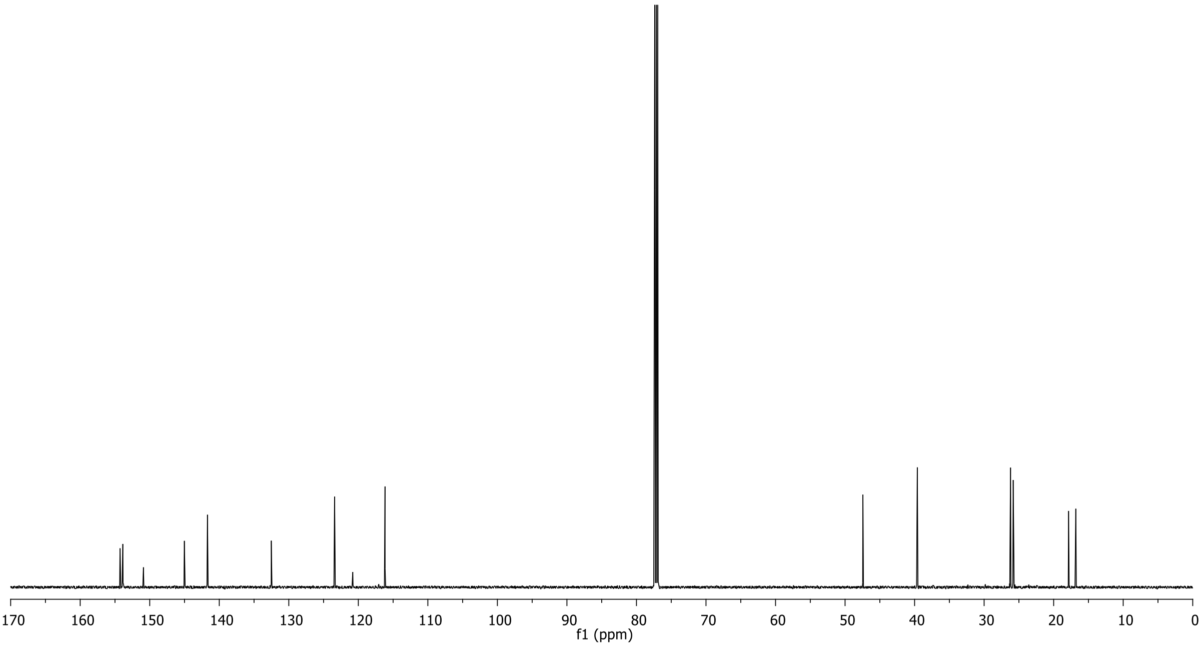

Figure S9. ^{13}C NMR spectrum (150 MHz, $CDCl_3$) of **4**.

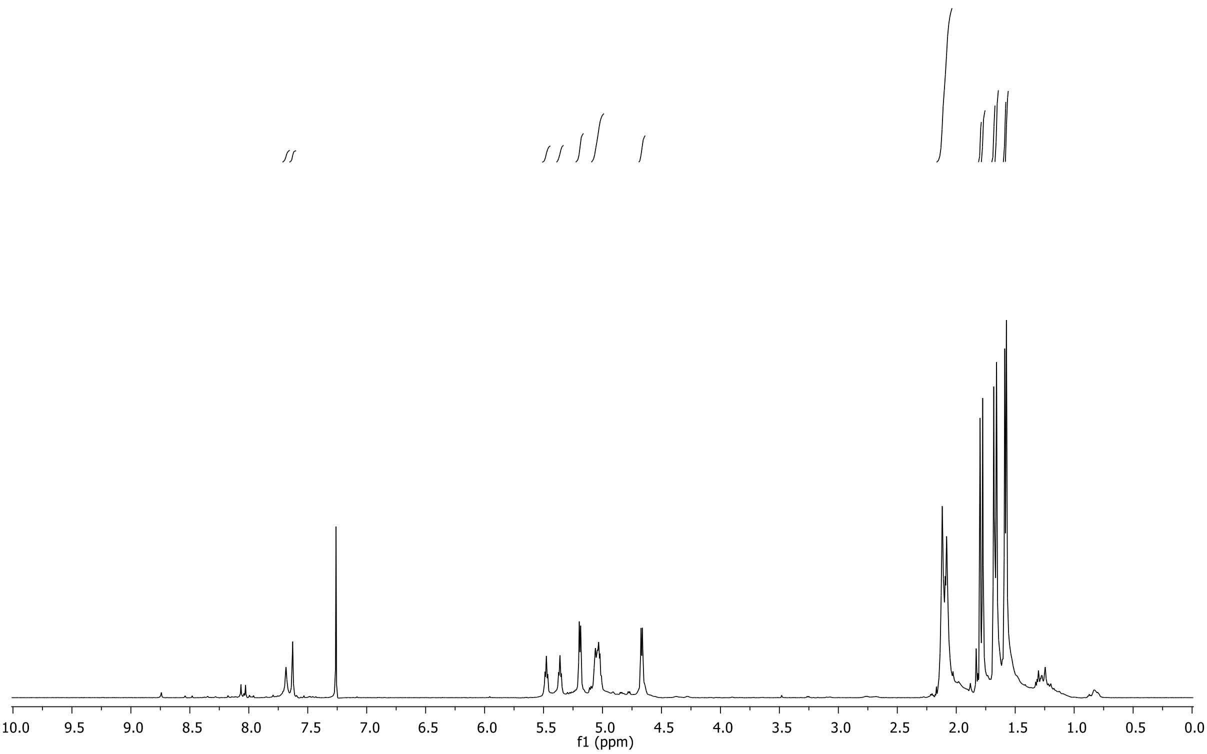

Figure S10. ^{1}H NMR spectrum (600 MHz, $CDCl_3$) of **5**.

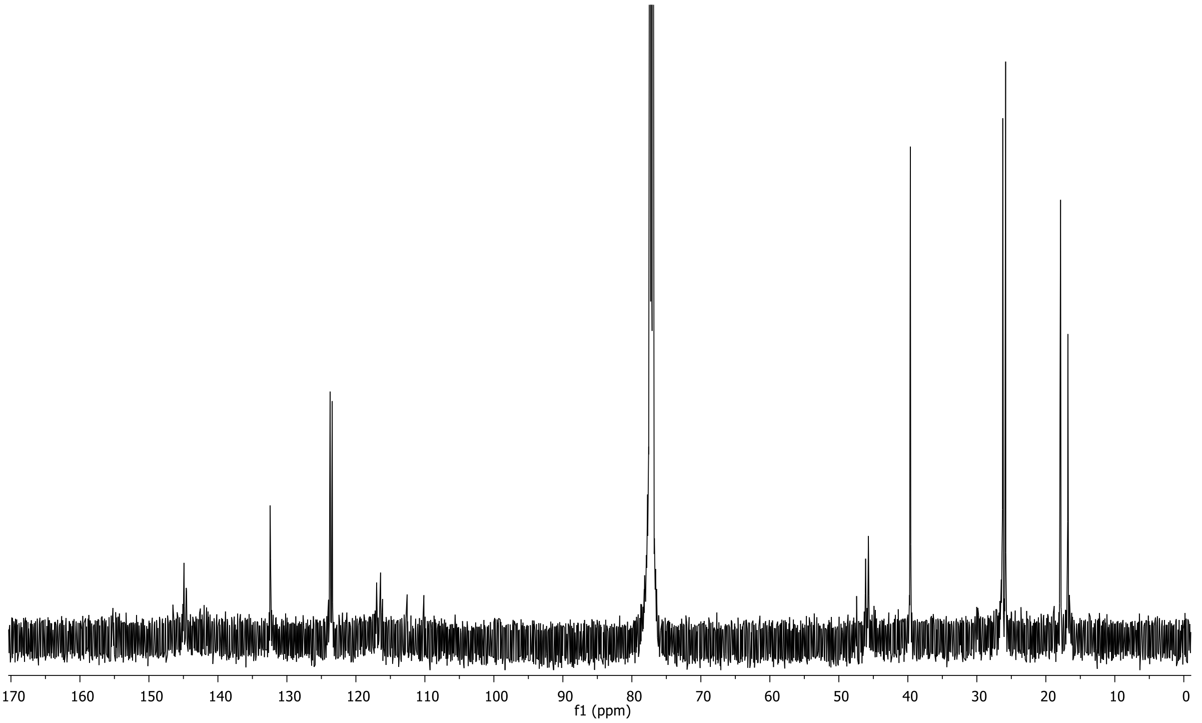

Figure S11. ^{13}C NMR spectrum (150 MHz, $CDCl_3$) of **5**.

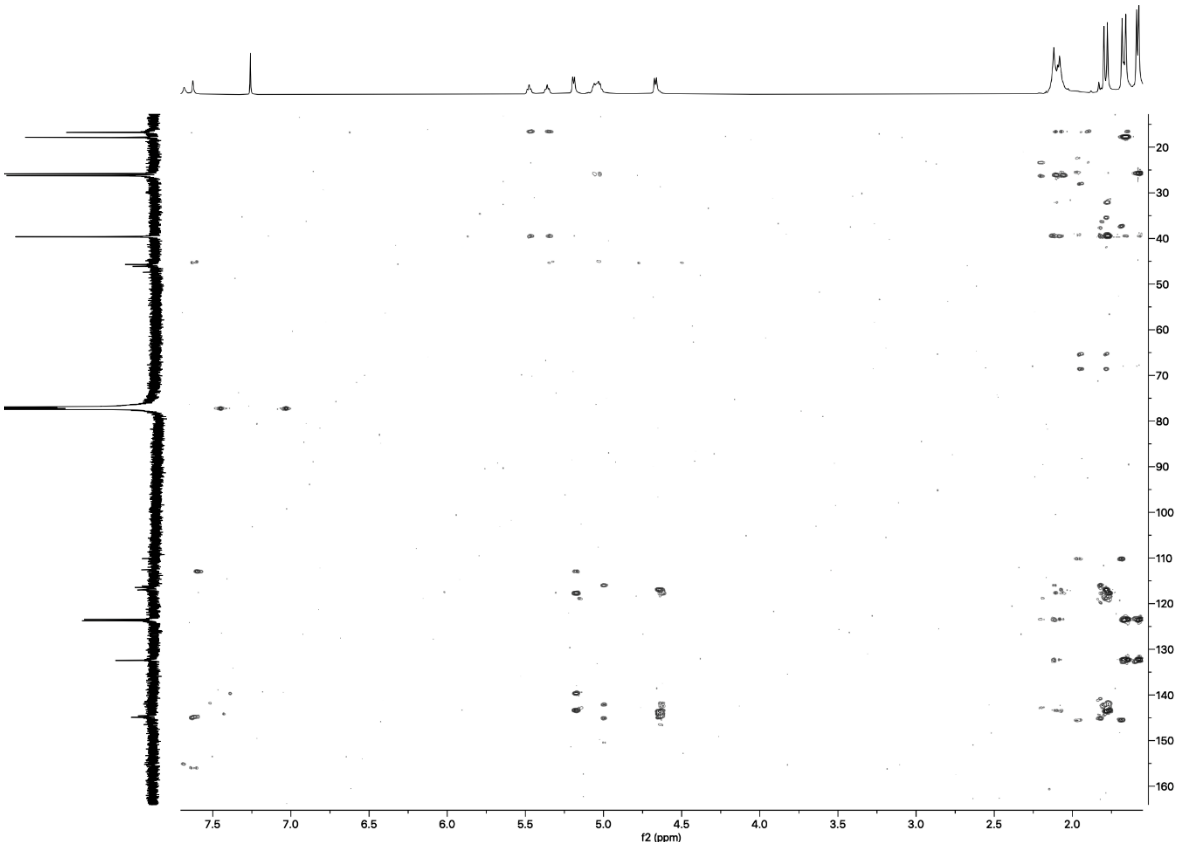

Figure S12. HMBC spectrum ($CDCl_3$) of **5**. Shows some ^{13}C signals not quite visible in the ^{13}C spectrum (Figure S11).

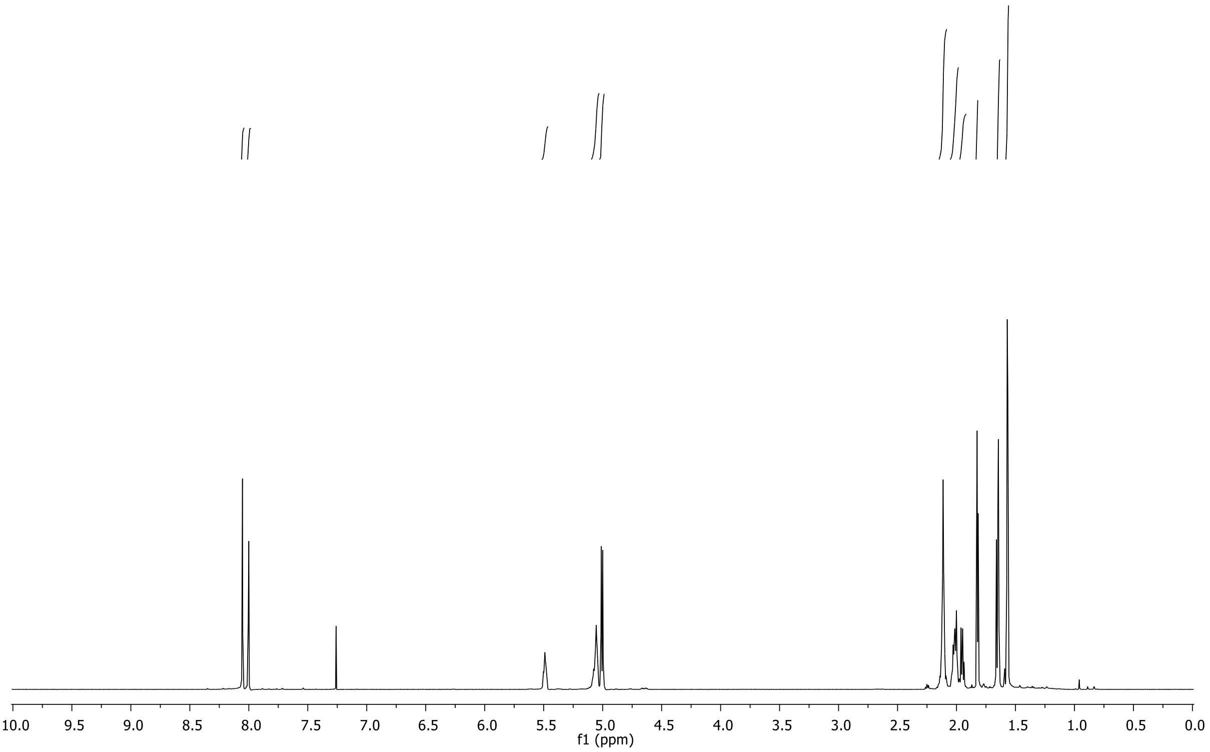

Figure S13. ^{1}H NMR spectrum (600 MHz, $CDCl_3$) of **6**.

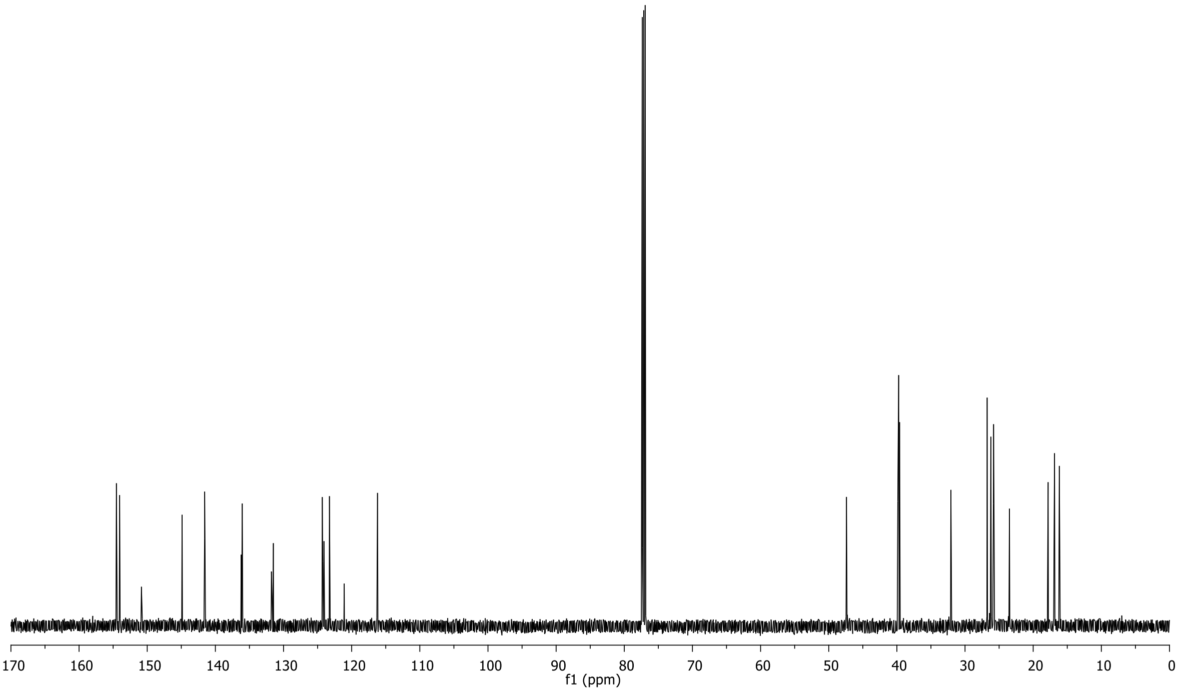

Figure S14. ^{13}C NMR spectrum (150 MHz, $CDCl_3$) of **6**.

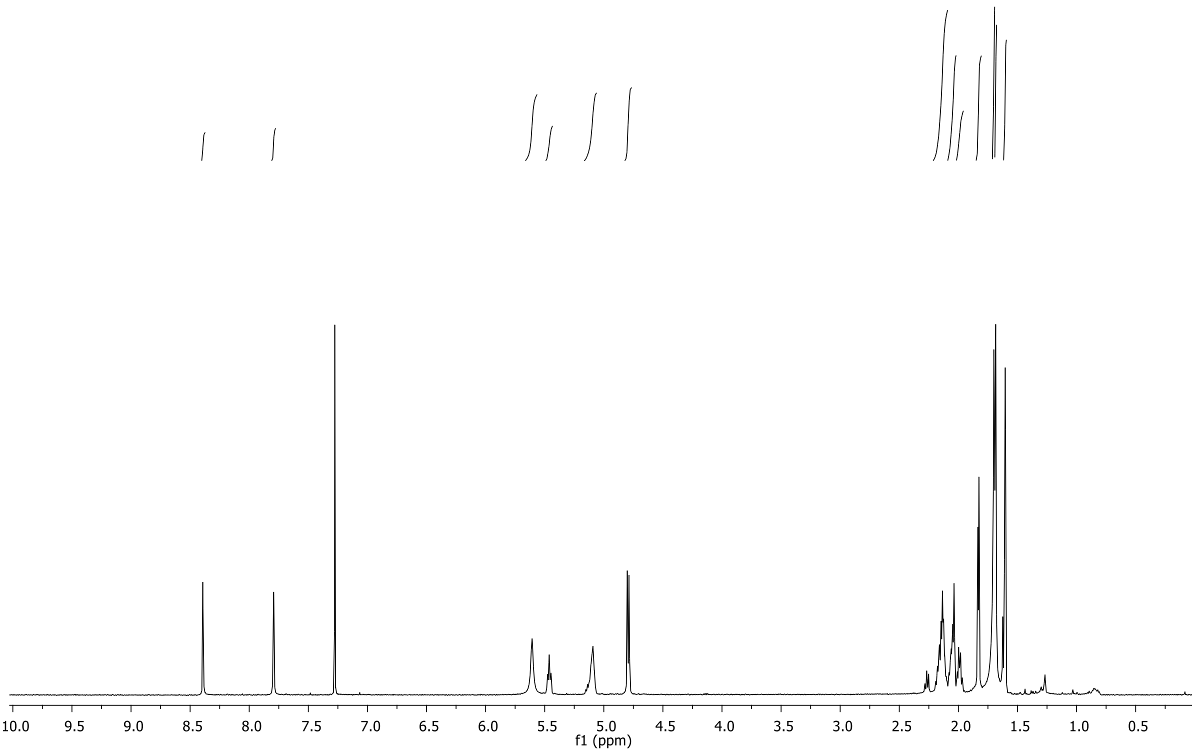

Figure S15. ^{1}H NMR spectrum (500 MHz, $CDCl_3$) of **7**.

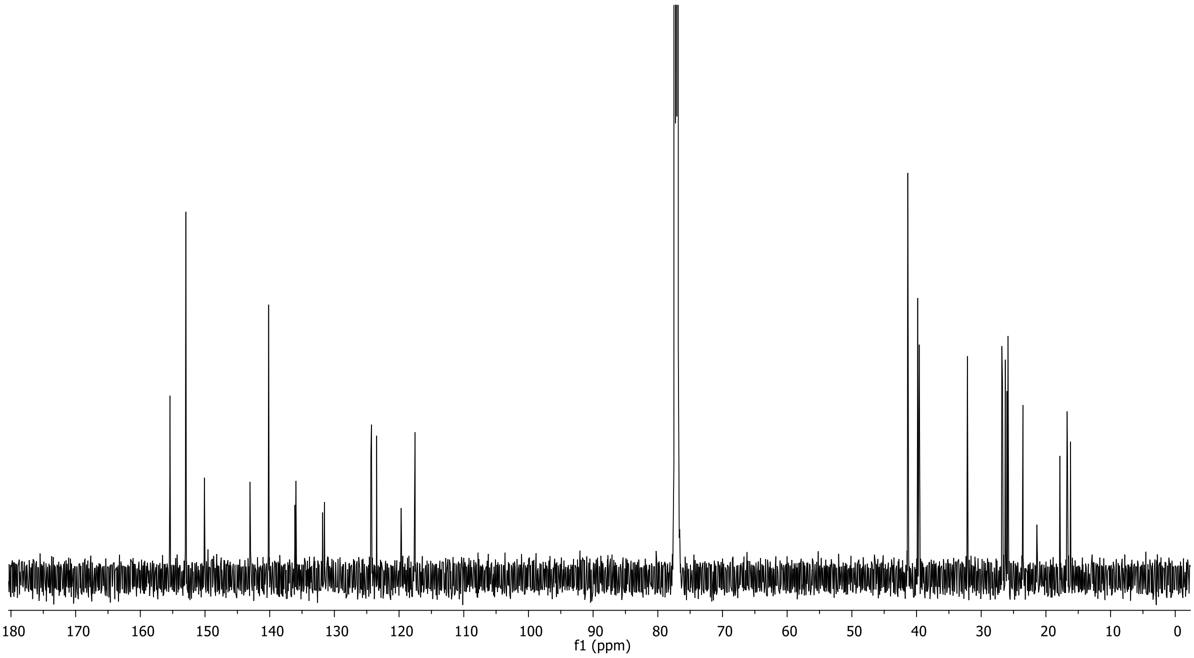

Figure S16. ^{13}C NMR spectrum (150 MHz, $CDCl_3$) of **7**.

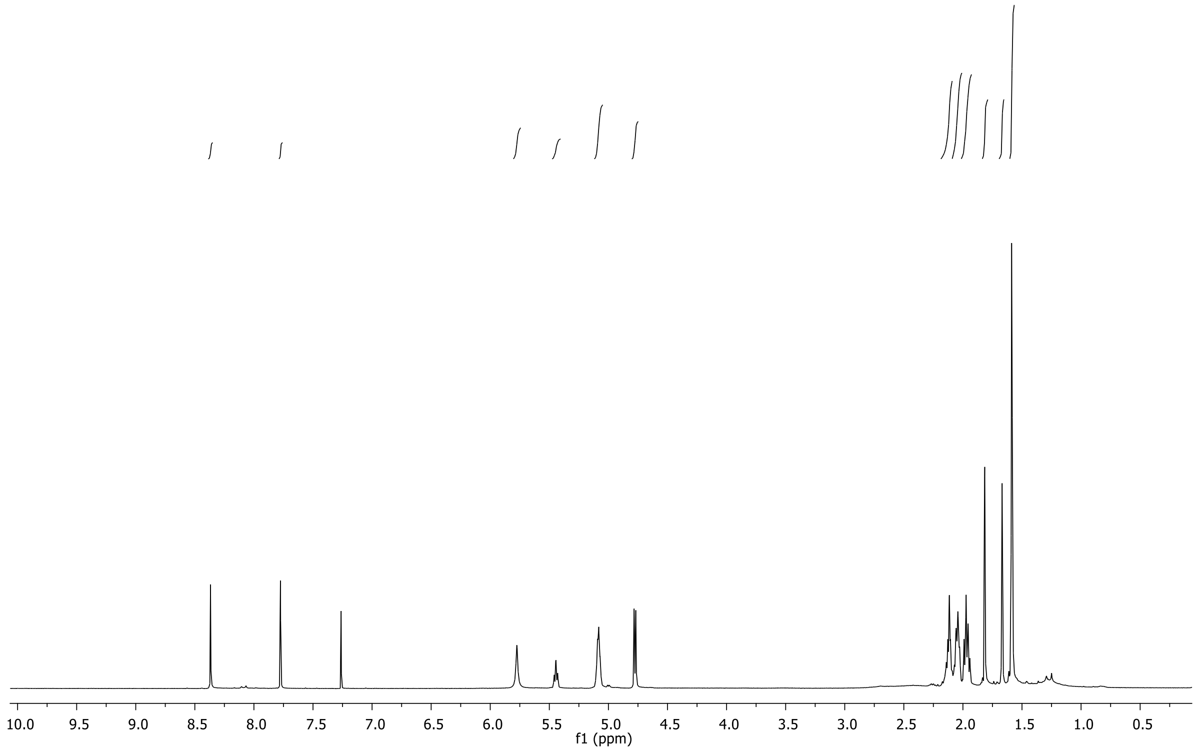

Figure S17. ^{1}H NMR spectrum (500 MHz, $CDCl_3$) of **8**.

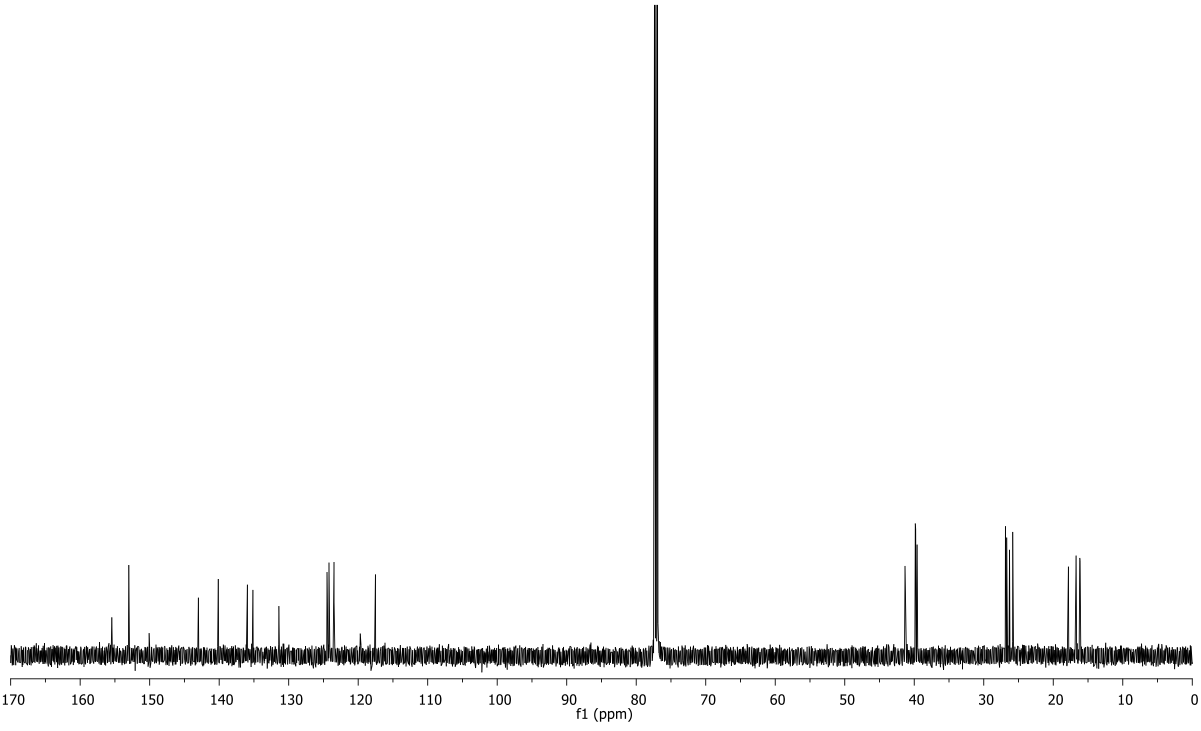

Figure S18. ^{13}C NMR spectrum (150 MHz, $CDCl_3$) of **8**.

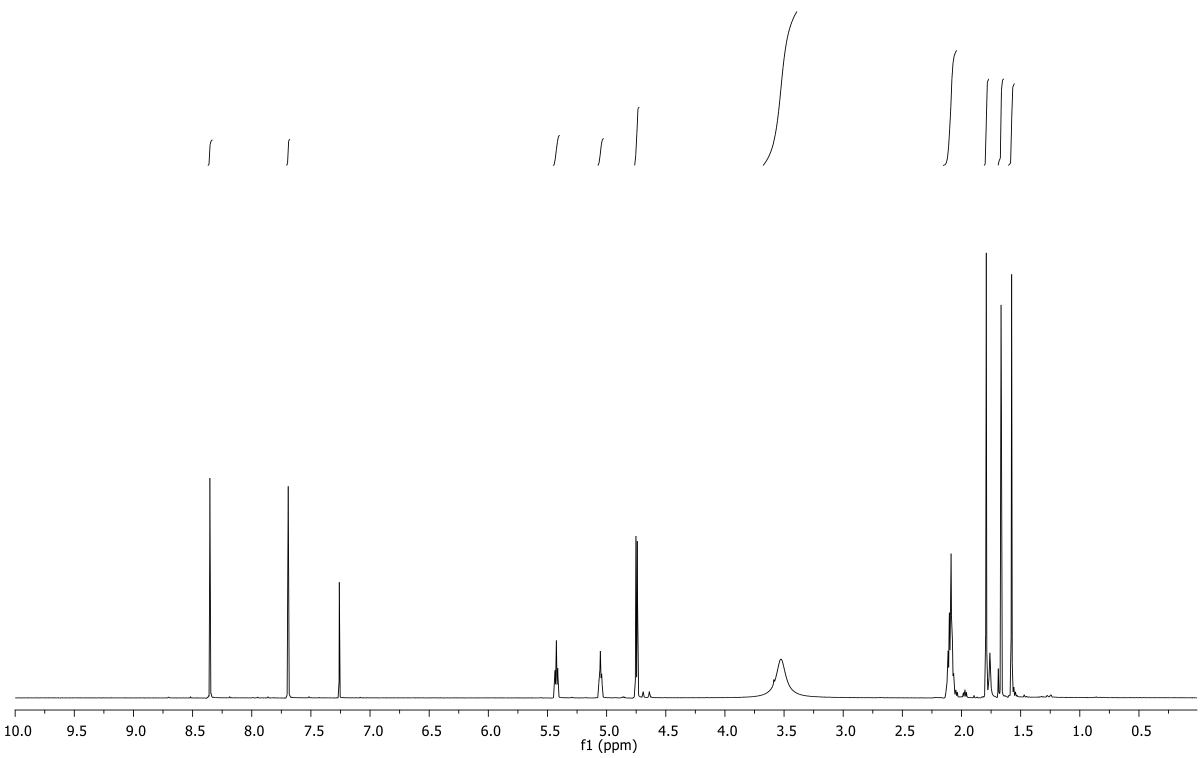

Figure S19. ^{1}H NMR spectrum (600 MHz, $CDCl_3$) of **9**.

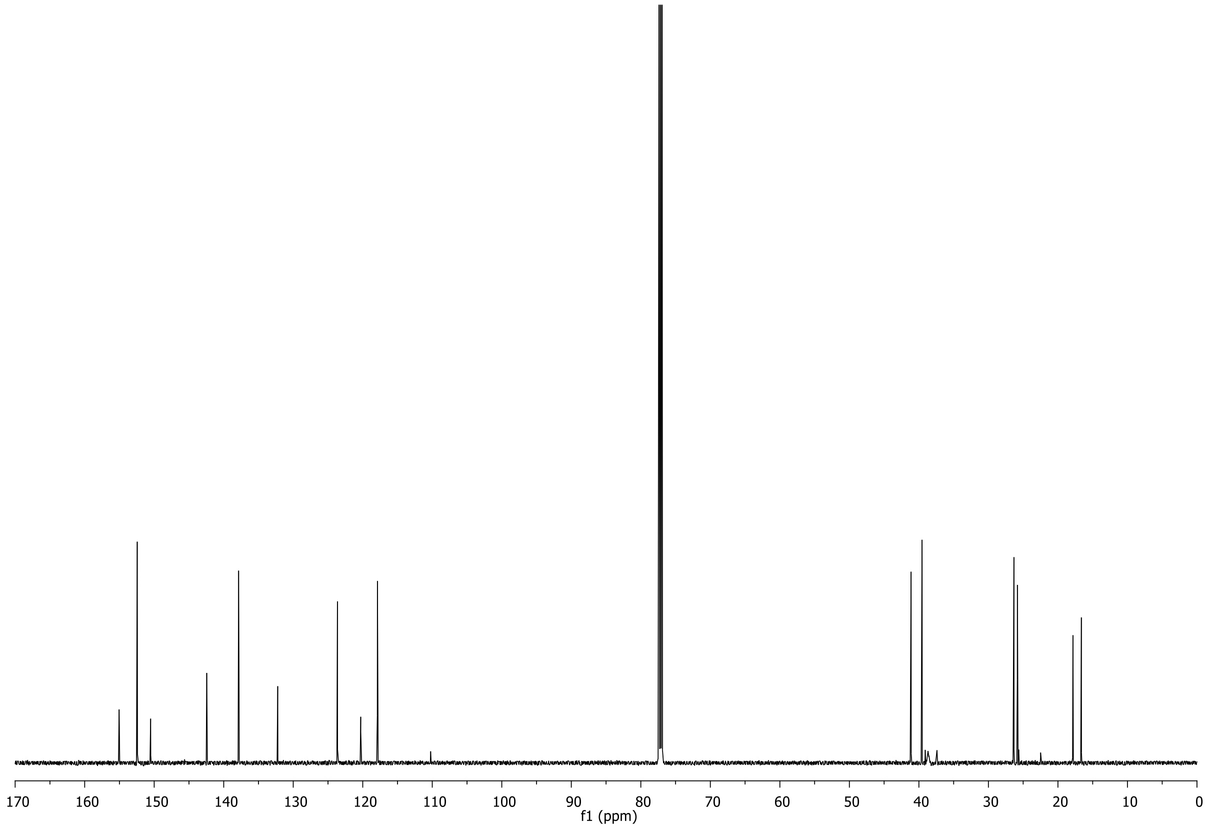

Figure S20. ^{13}C NMR spectrum (150 MHz, $CDCl_3$) of **9**.

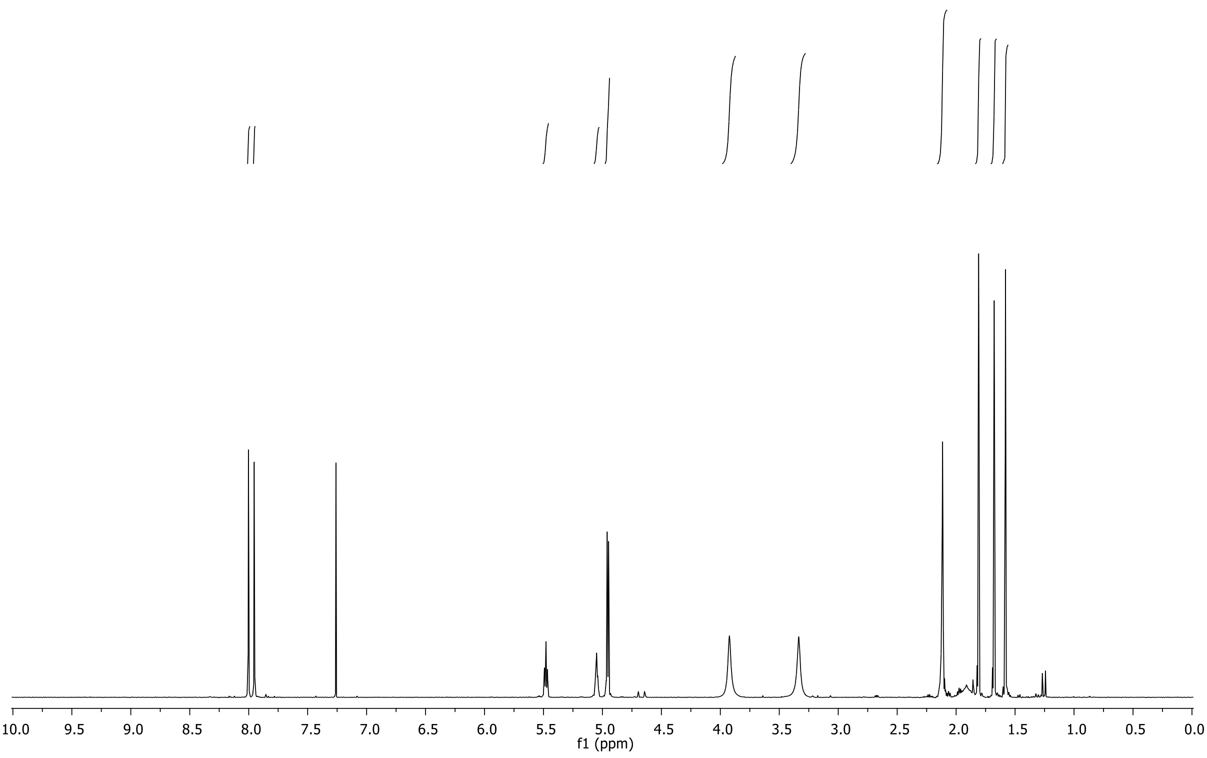

Figure S21. ^{1}H NMR spectrum (600 MHz, $CDCl_3$) of **10**.

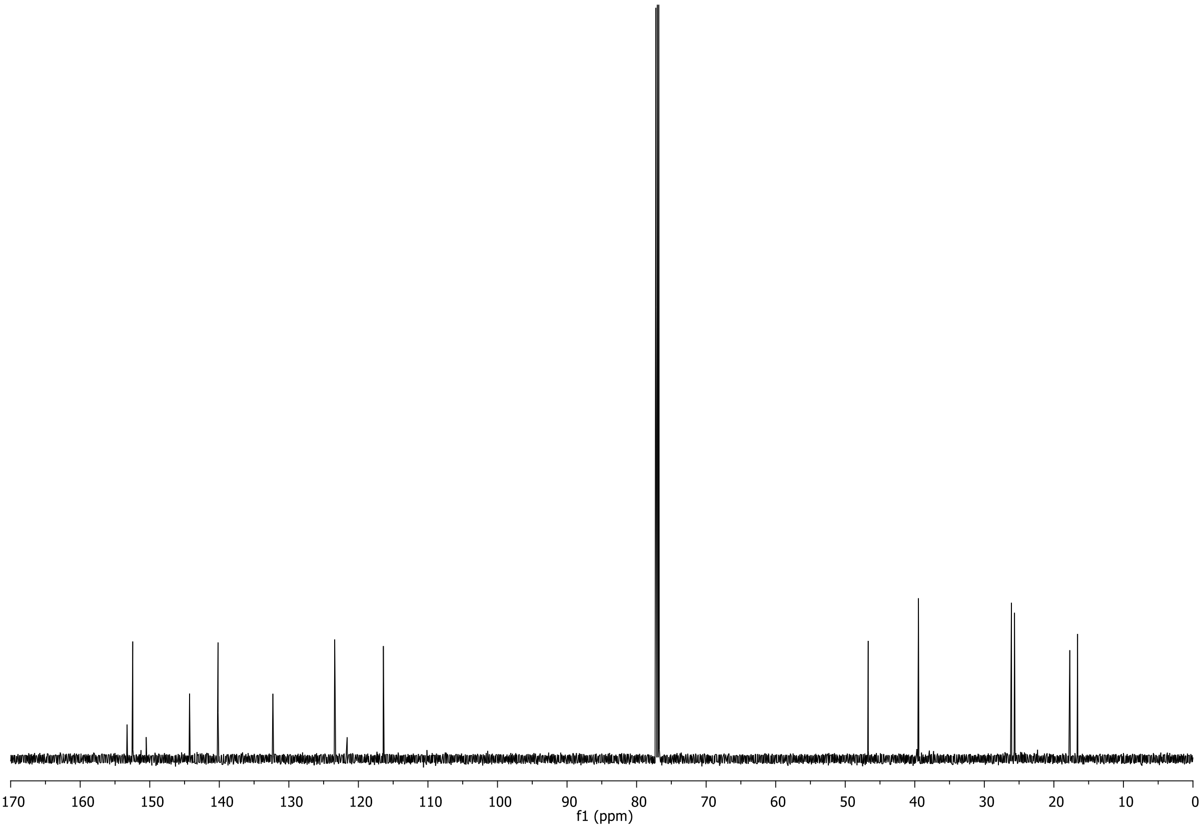

Figure S22. ^{13}C NMR spectrum (150 MHz, $CDCl_3$) of **10**.

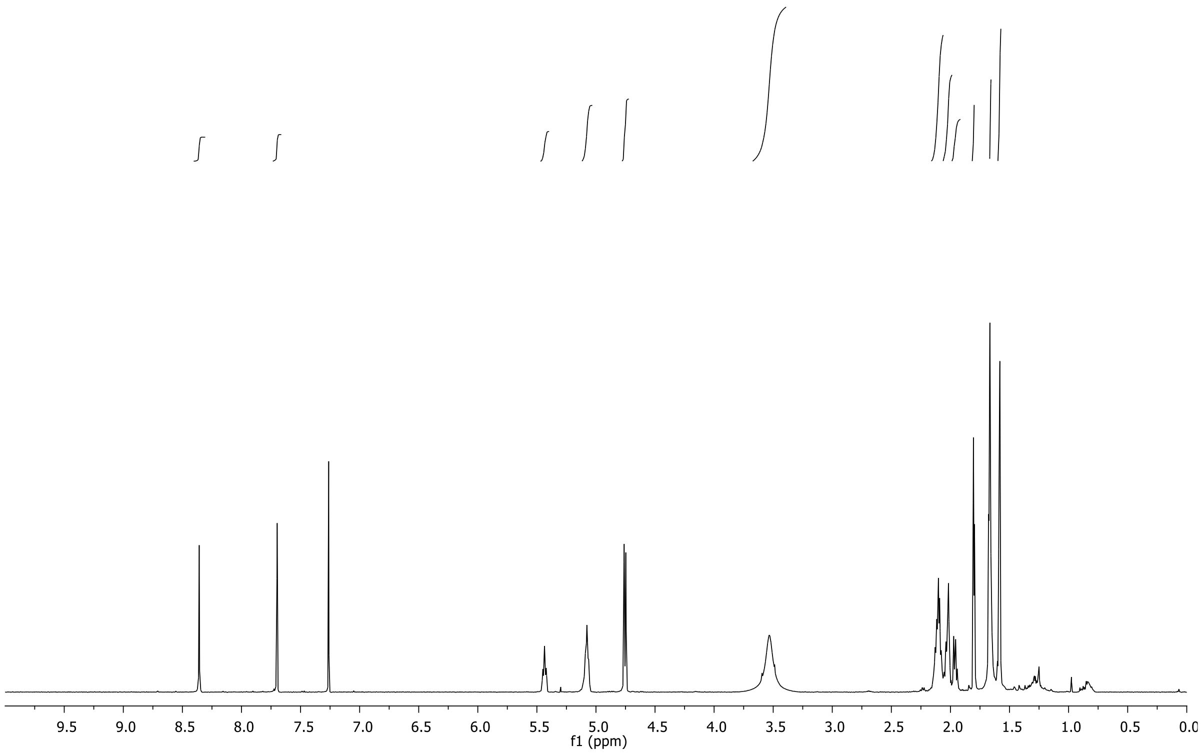

Figure S23. ^{1}H NMR spectrum (500 MHz, $CDCl_3$) of **11**.

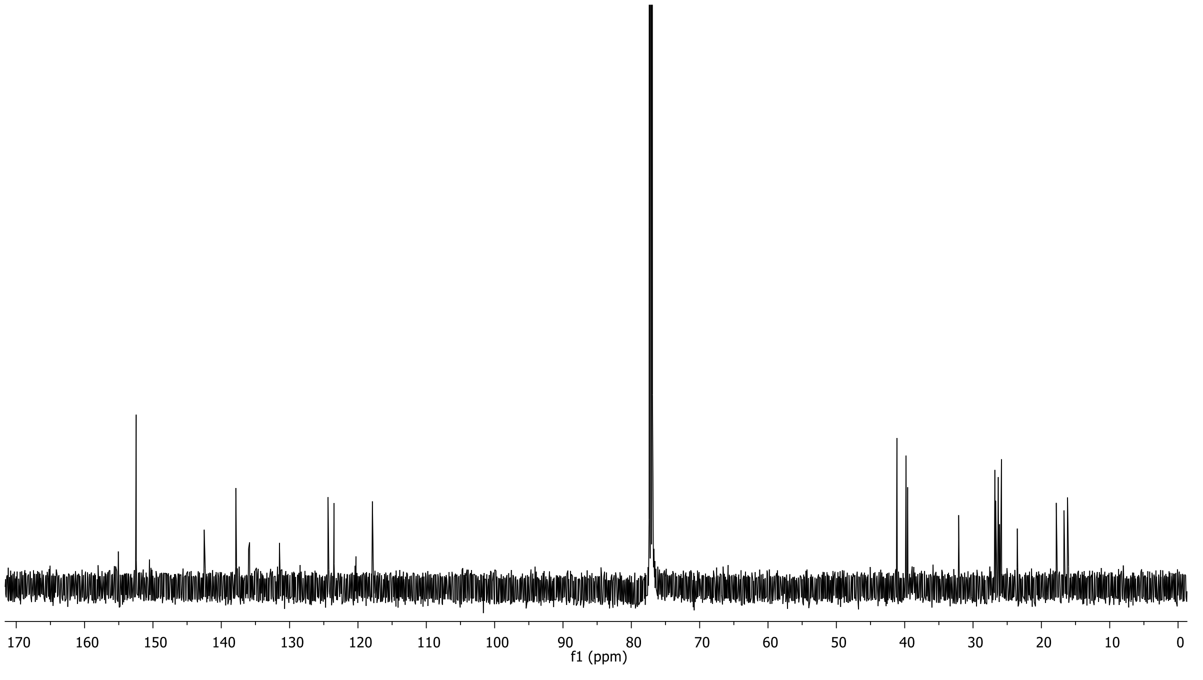

Figure S24. ^{13}C NMR spectrum (150 MHz, $CDCl_3$) of **11**.

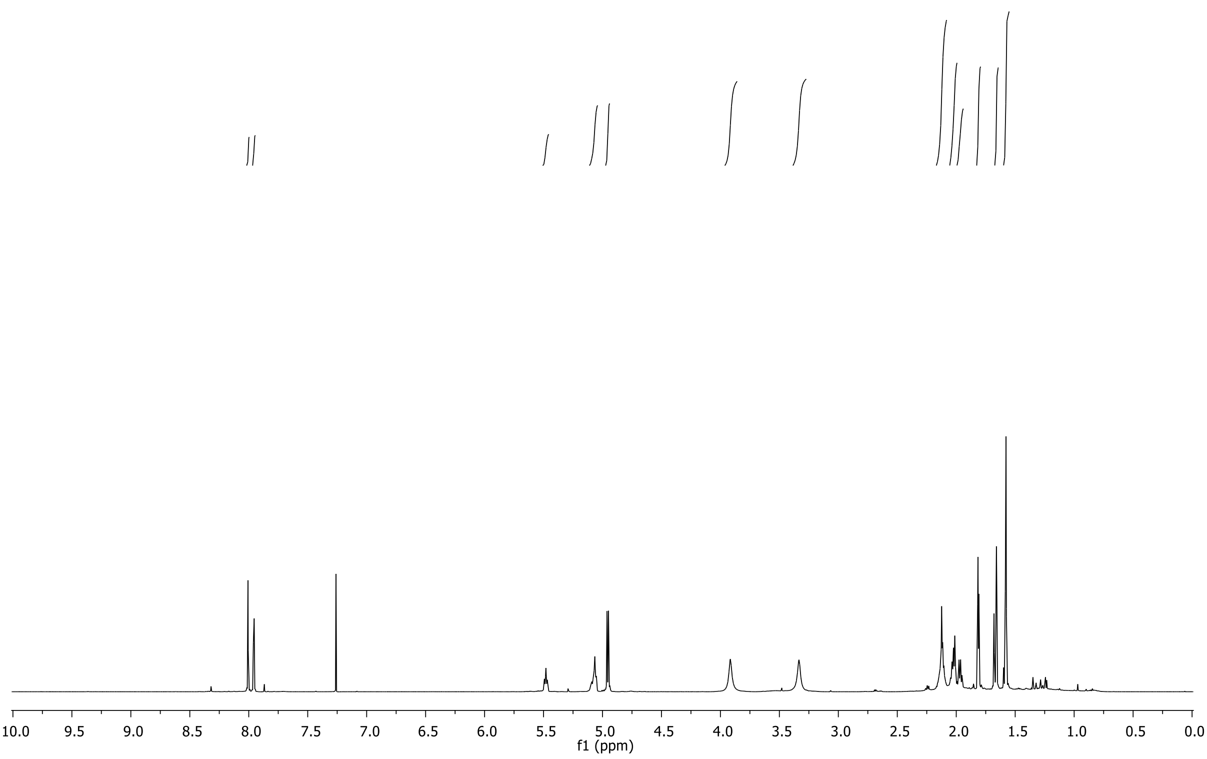

Figure S25. ^{1}H NMR spectrum (600 MHz, $CDCl_3$) of **12**.

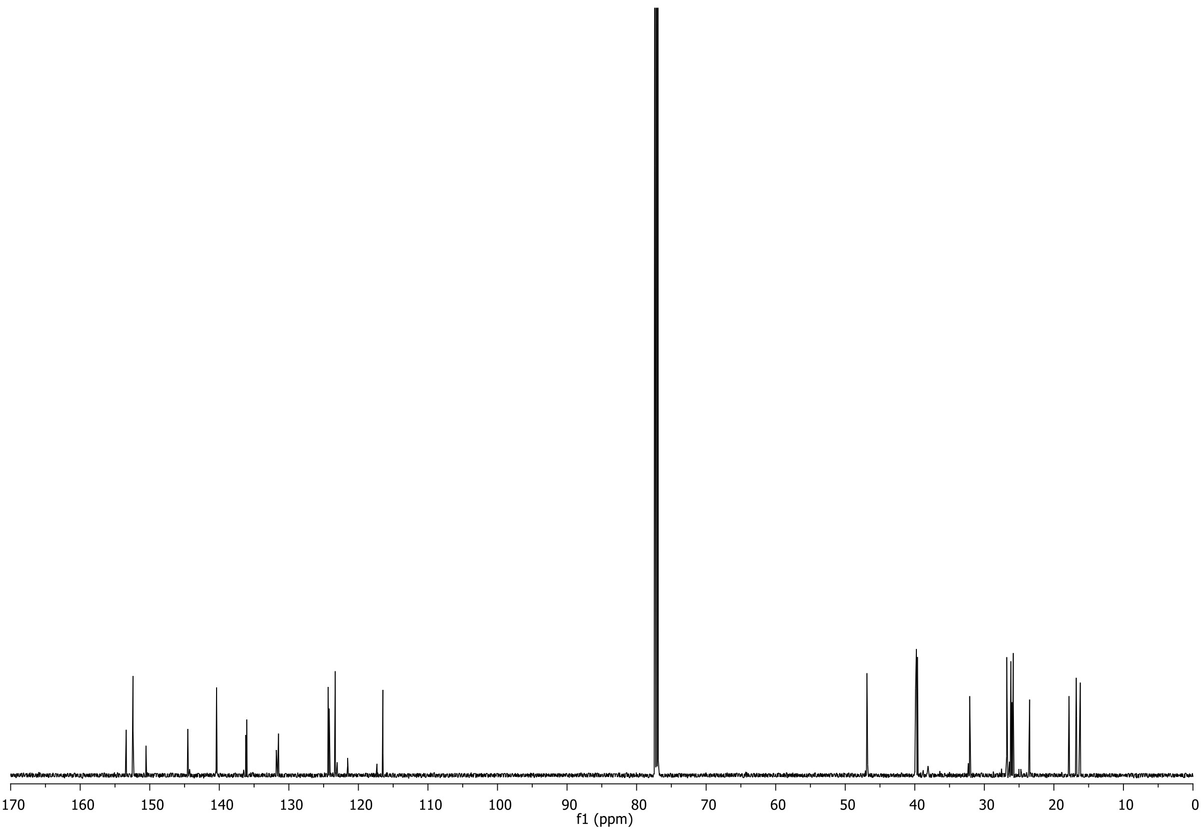

Figure S26. ^{13}C NMR spectrum (150 MHz, $CDCl_3$) of **12**.

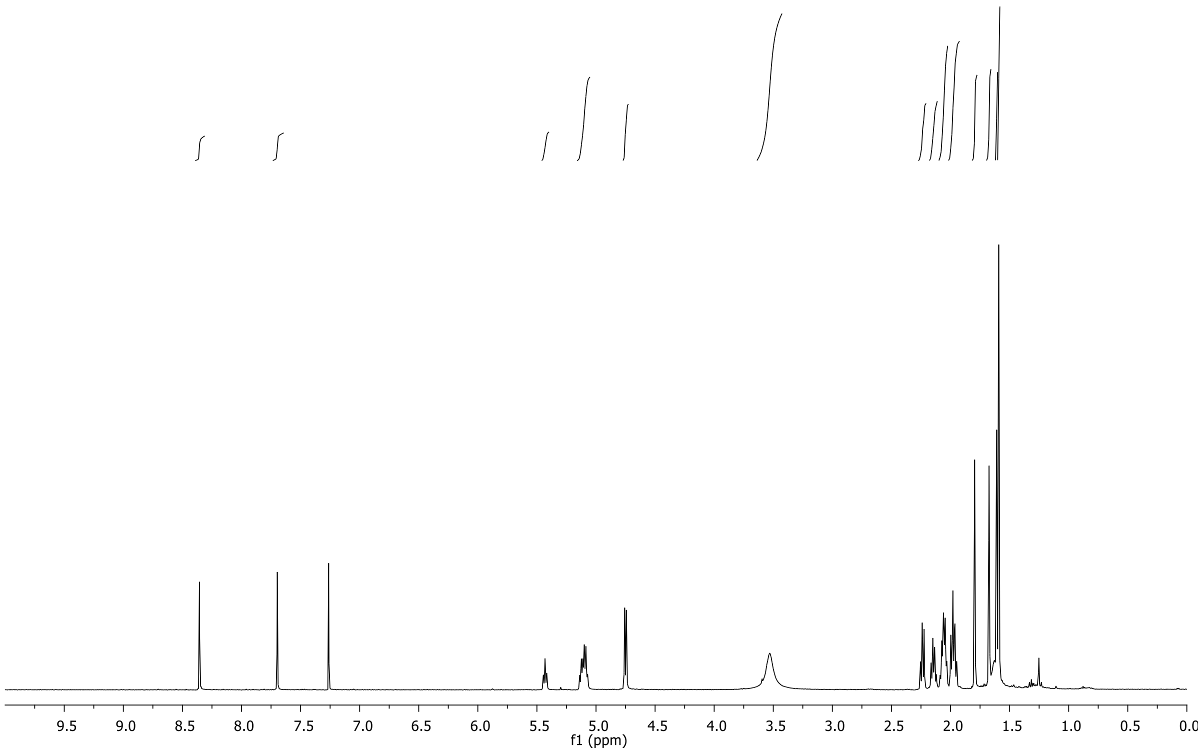

Figure S27. ^{1}H NMR spectrum (500 MHz, $CDCl_3$) of **13**.

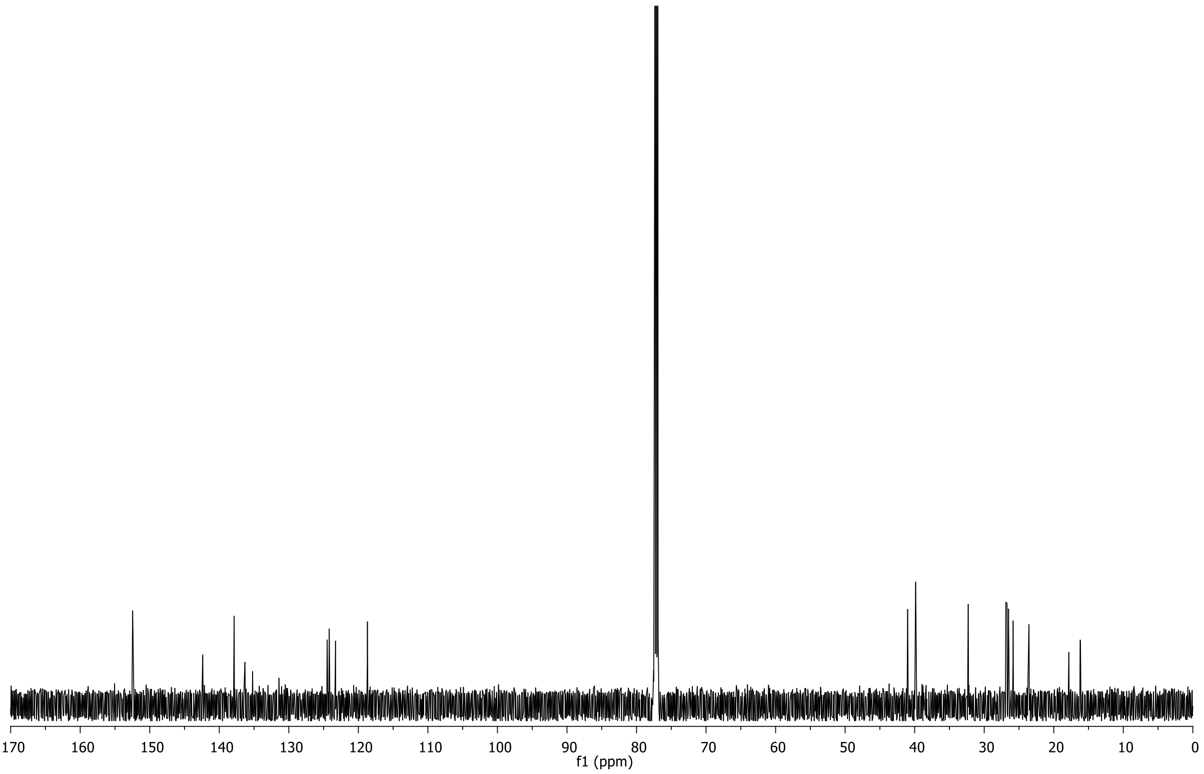

Figure S28. ^{13}C NMR spectrum (150 MHz, $CDCl_3$) of **13**.

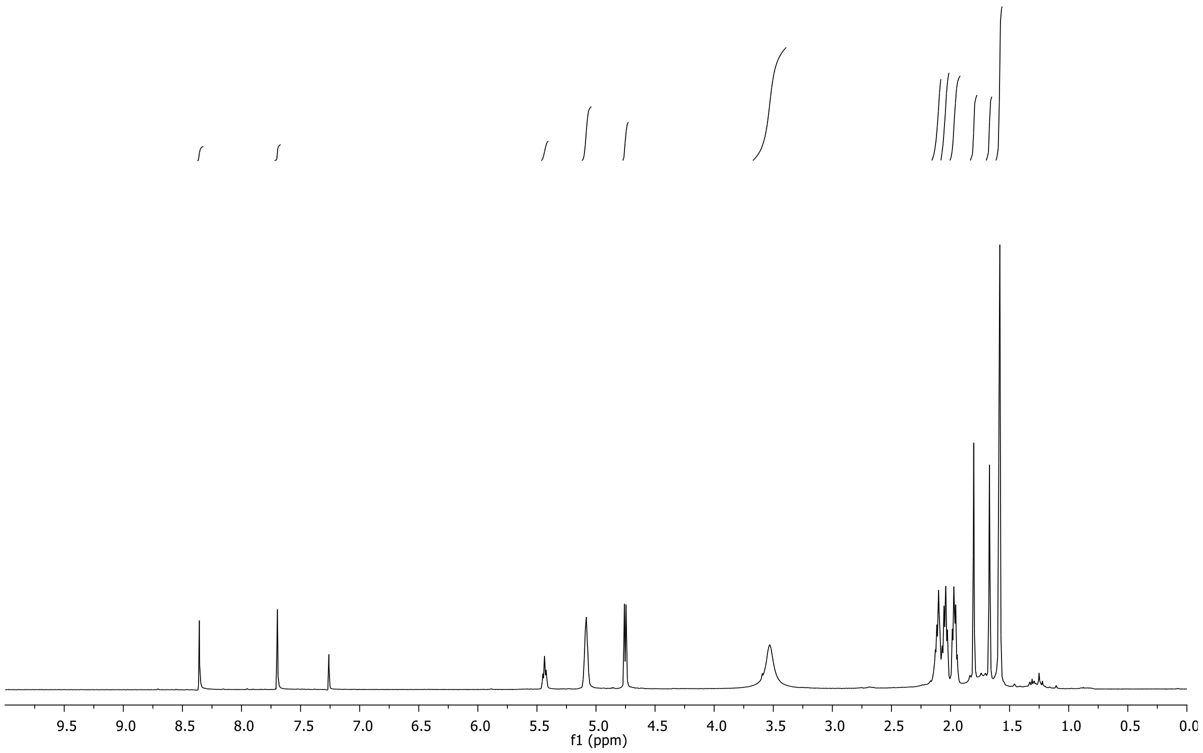

Figure S29. ^{1}H NMR spectrum (500 MHz, CDCl3) of **14**.

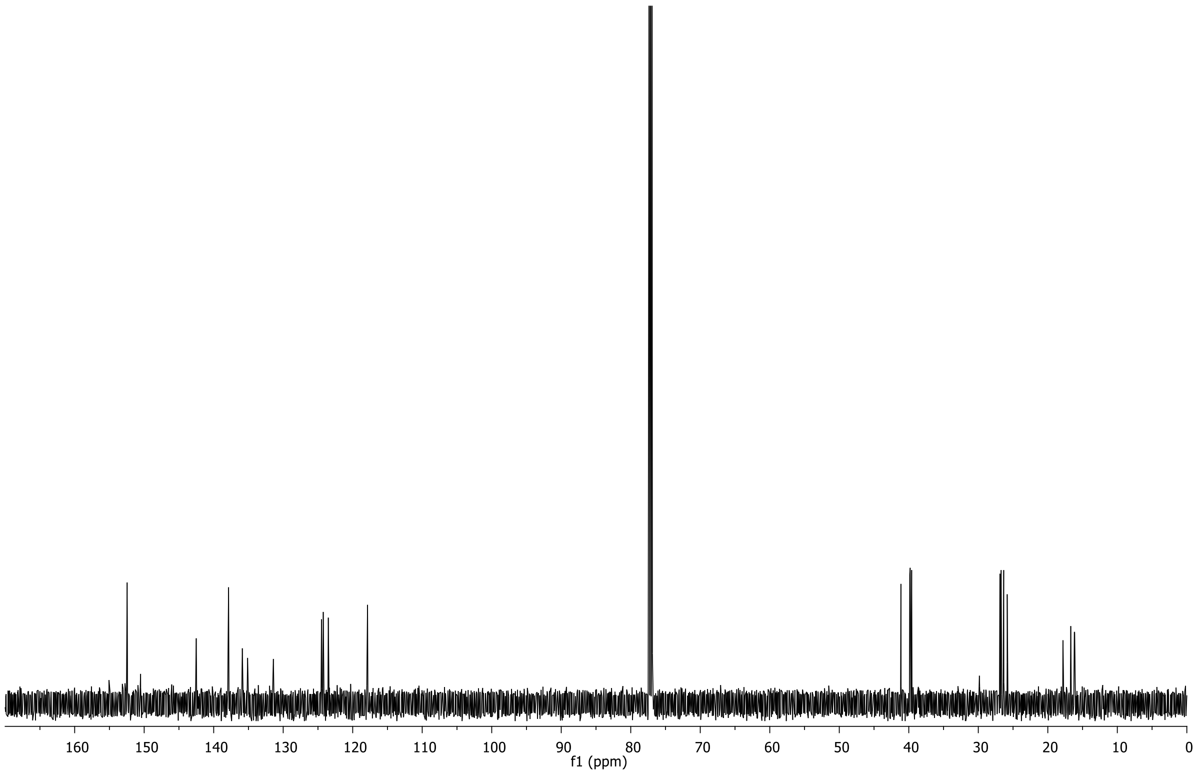

Figure S30. ^{13}C NMR spectrum (150 MHz, CDCl3) of **14**.

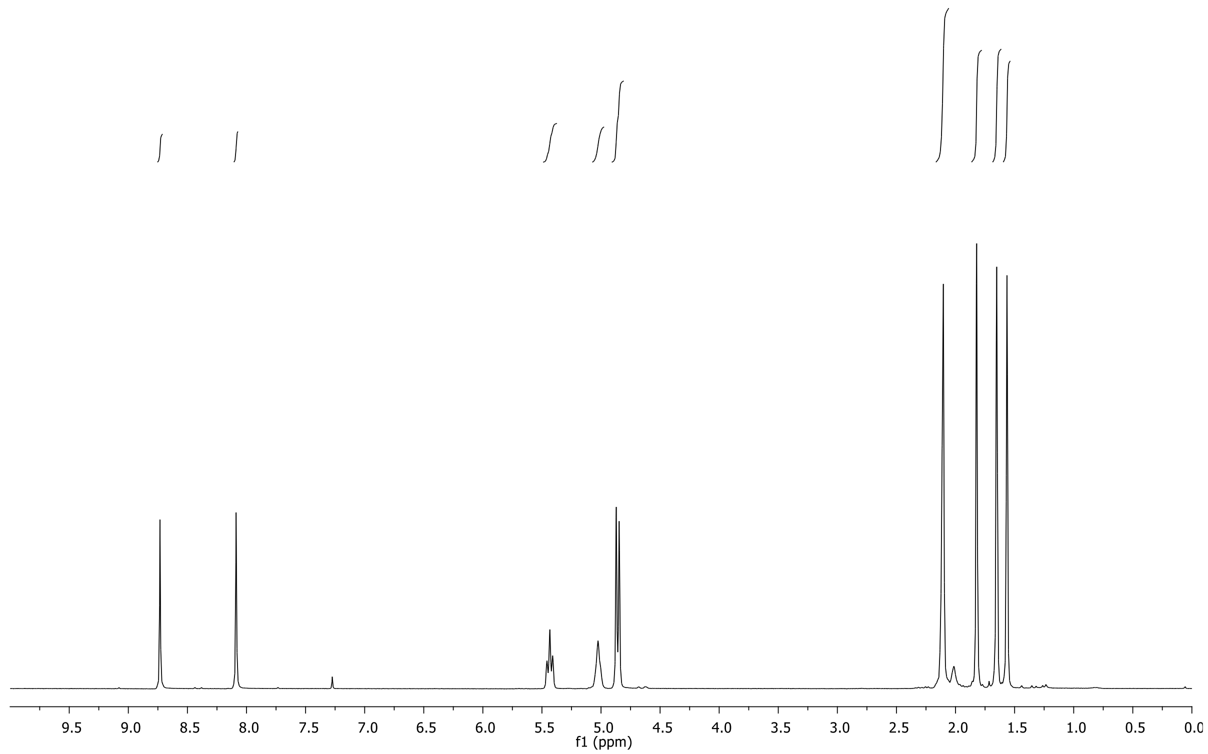

Figure S31. ^{1}H NMR spectrum (300 MHz, $CDCl_3$) of **15**.

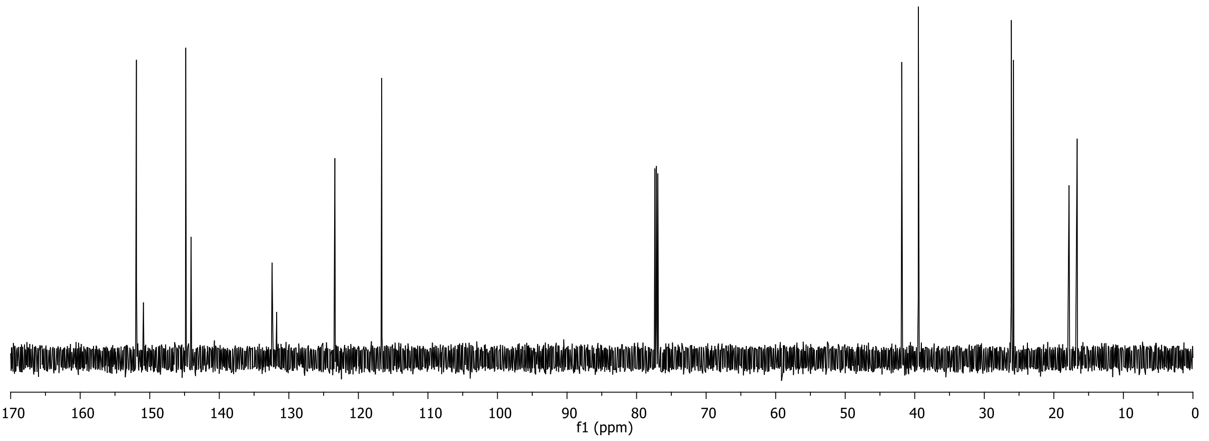

Figure S32. ^{13}C NMR spectrum (150 MHz, $CDCl_3$) of **15**.

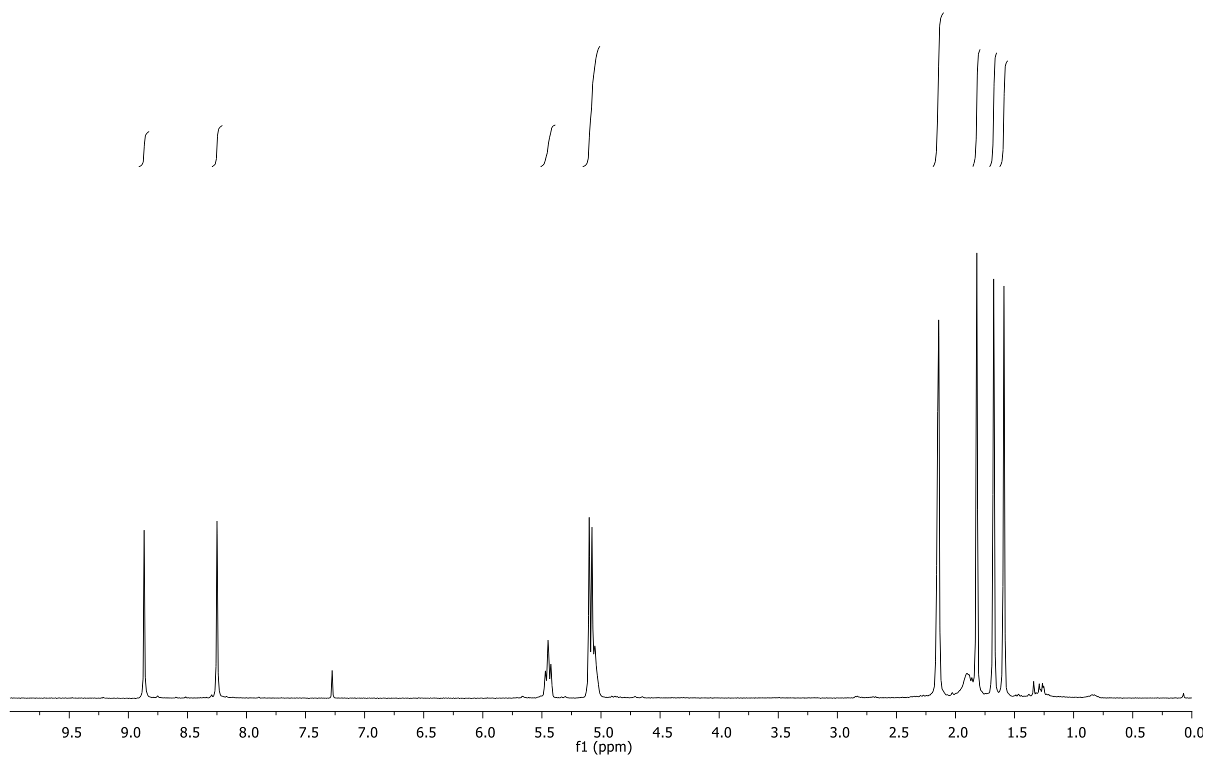

Figure S33. ^{1}H NMR spectrum (300 MHz, $CDCl_3$) of **16**.

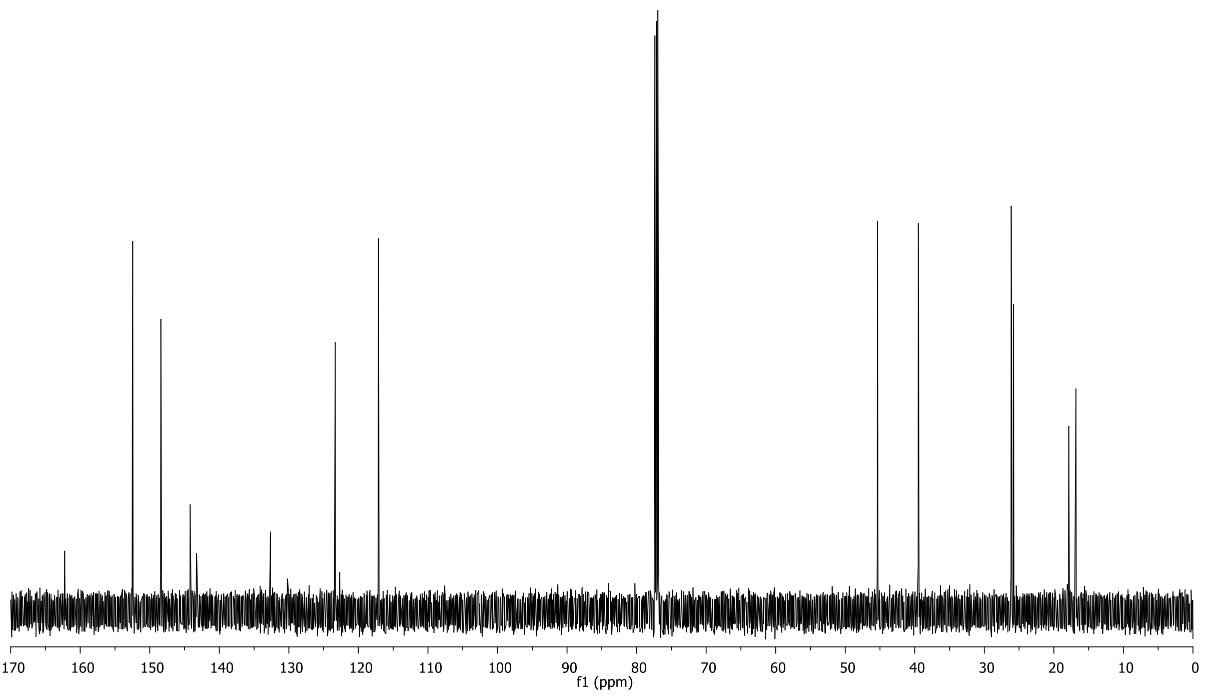

Figure S34. ^{13}C NMR spectrum (150 MHz, $CDCl_3$) of **16**.

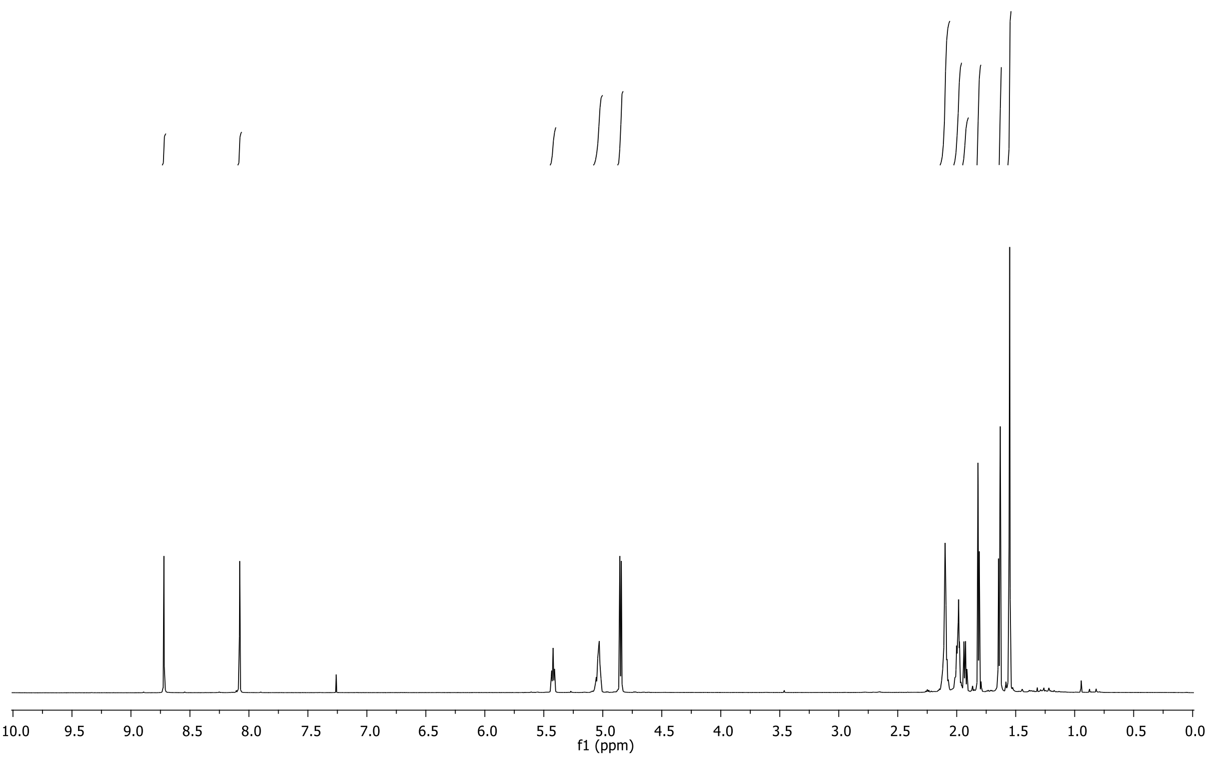

Figure S35. ^{1}H NMR spectrum (600 MHz, $CDCl_3$) of **17**.

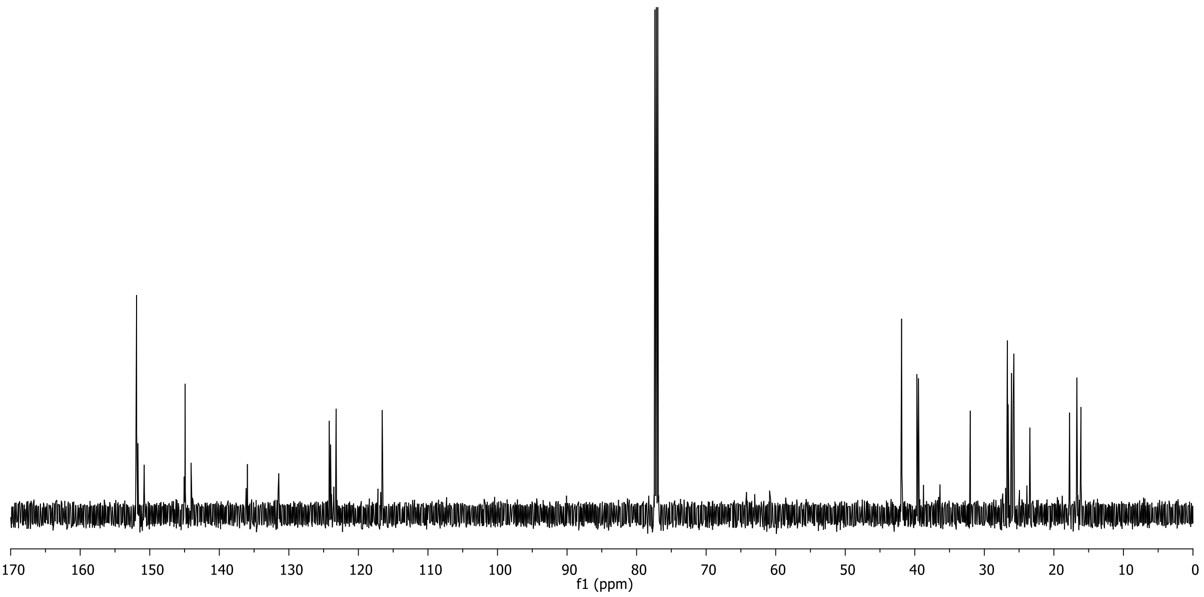

Figure S36. ^{13}C NMR spectrum (150 MHz, $CDCl_3$) of **17**.

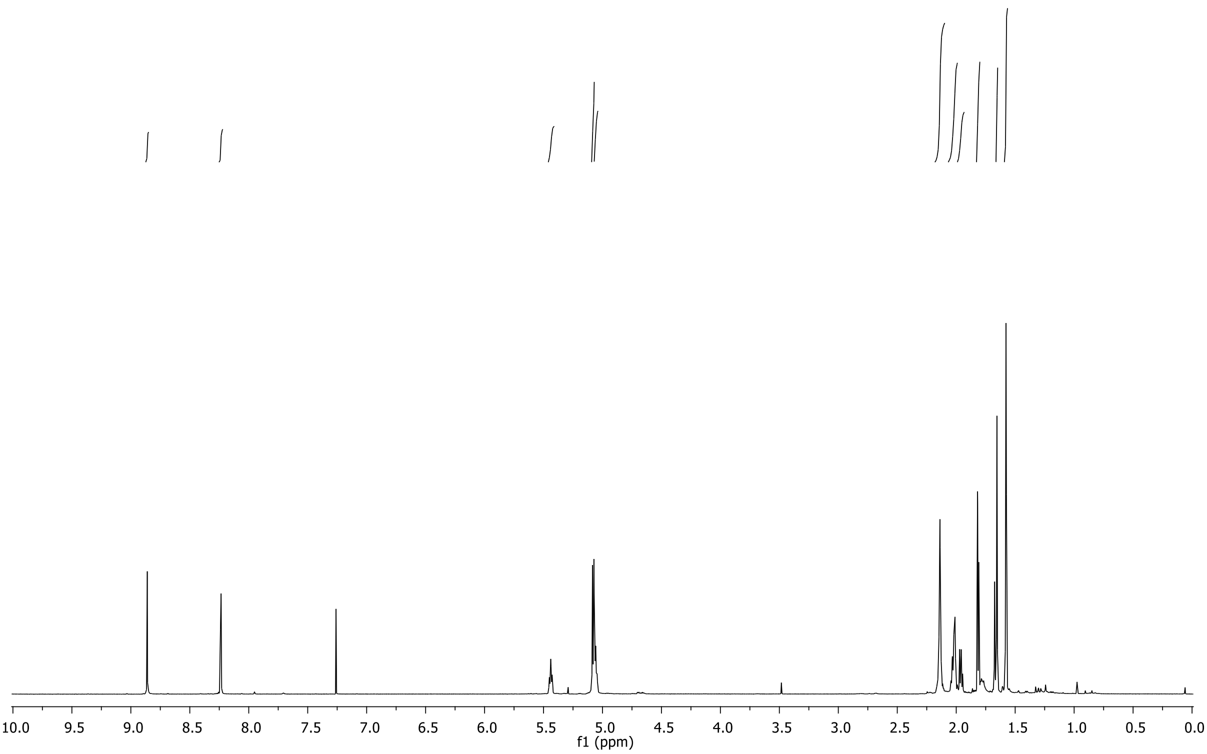

Figure S37. ^{1}H NMR spectrum (600 MHz, $CDCl_3$) of **18**.

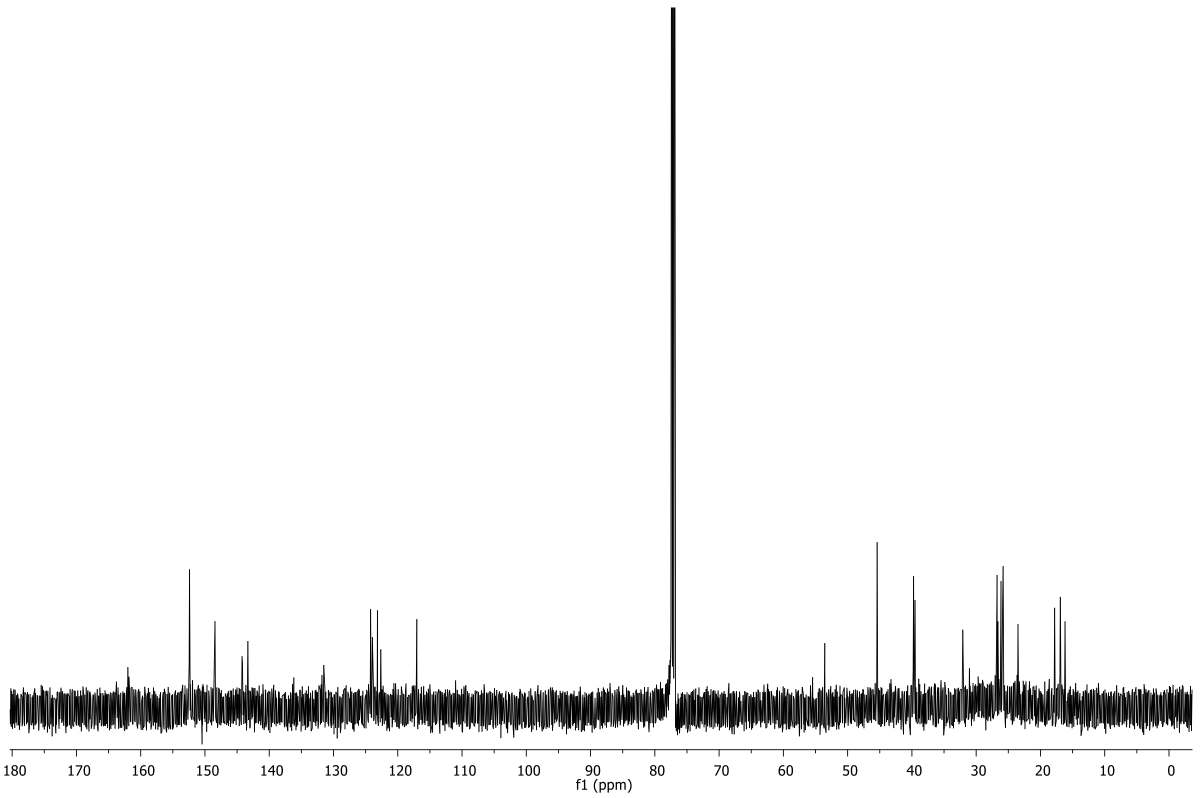

Figure S38. ^{13}C NMR spectrum (150 MHz, $CDCl_3$) of **18**.

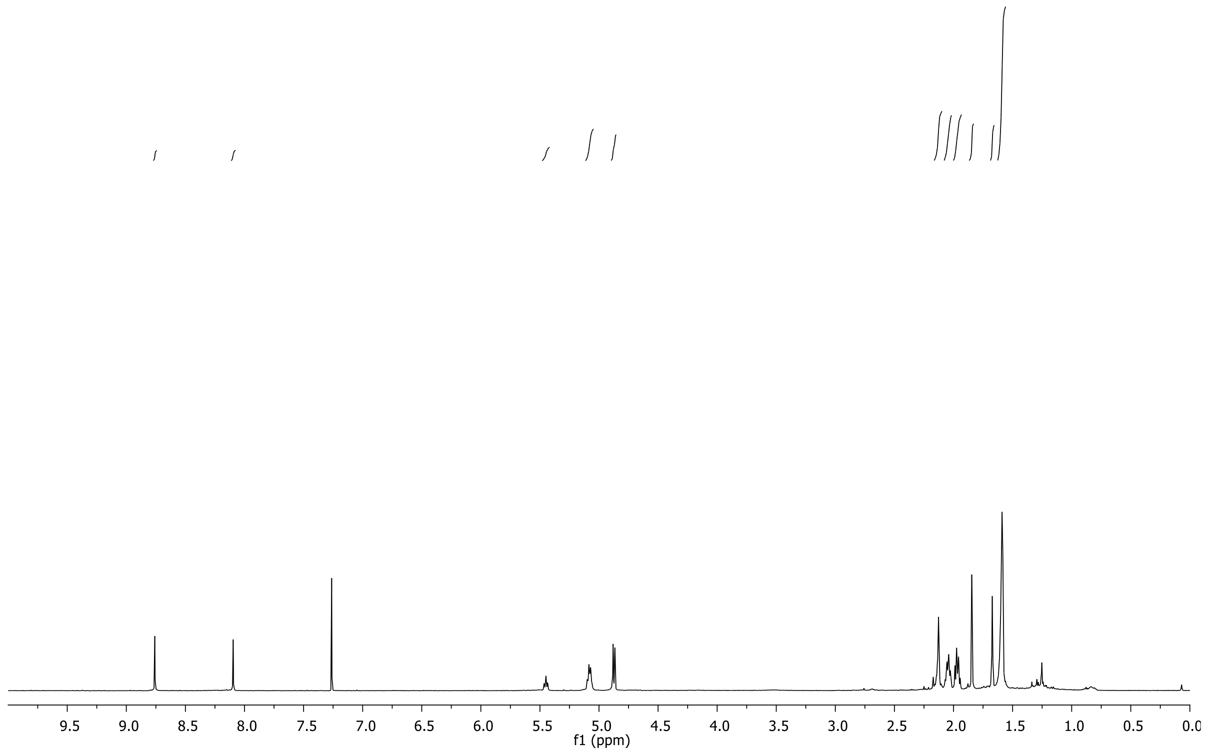

Figure S39. ^{1}H NMR spectrum (500 MHz, $CDCl_3$) of **19**.

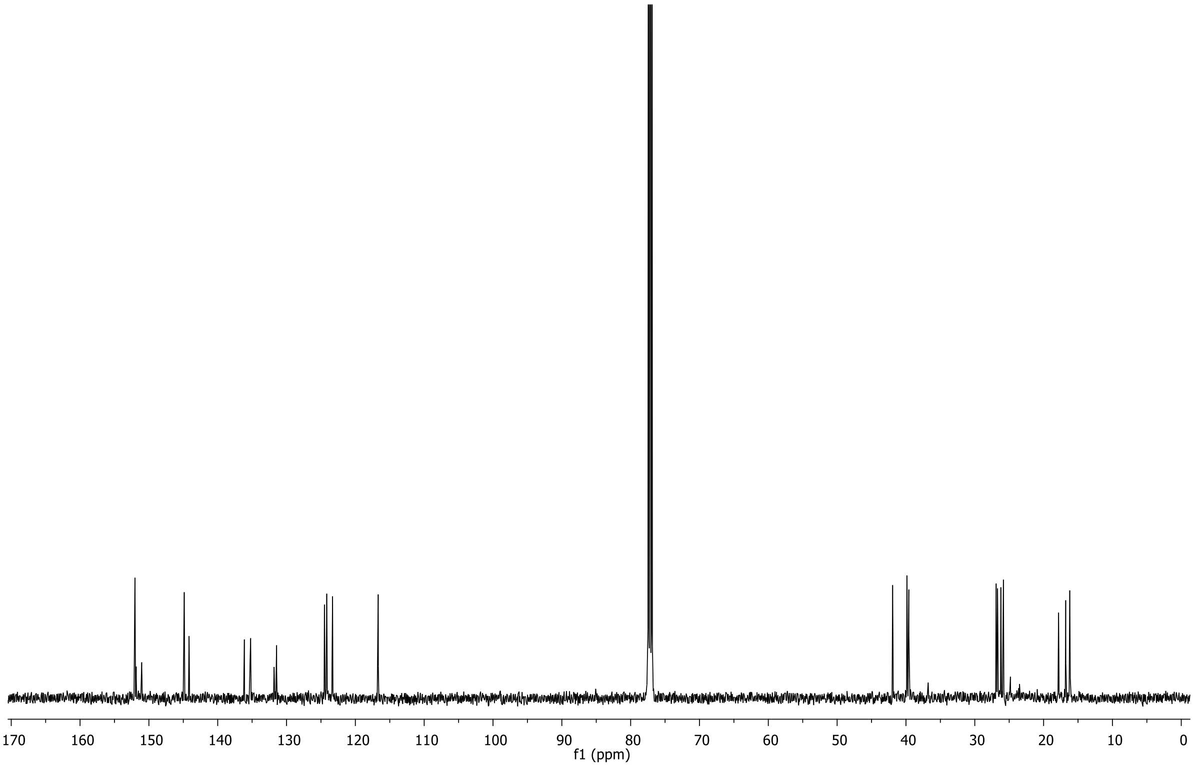

Figure S40. ^{13}C NMR spectrum (150 MHz, $CDCl_3$) of **19**.

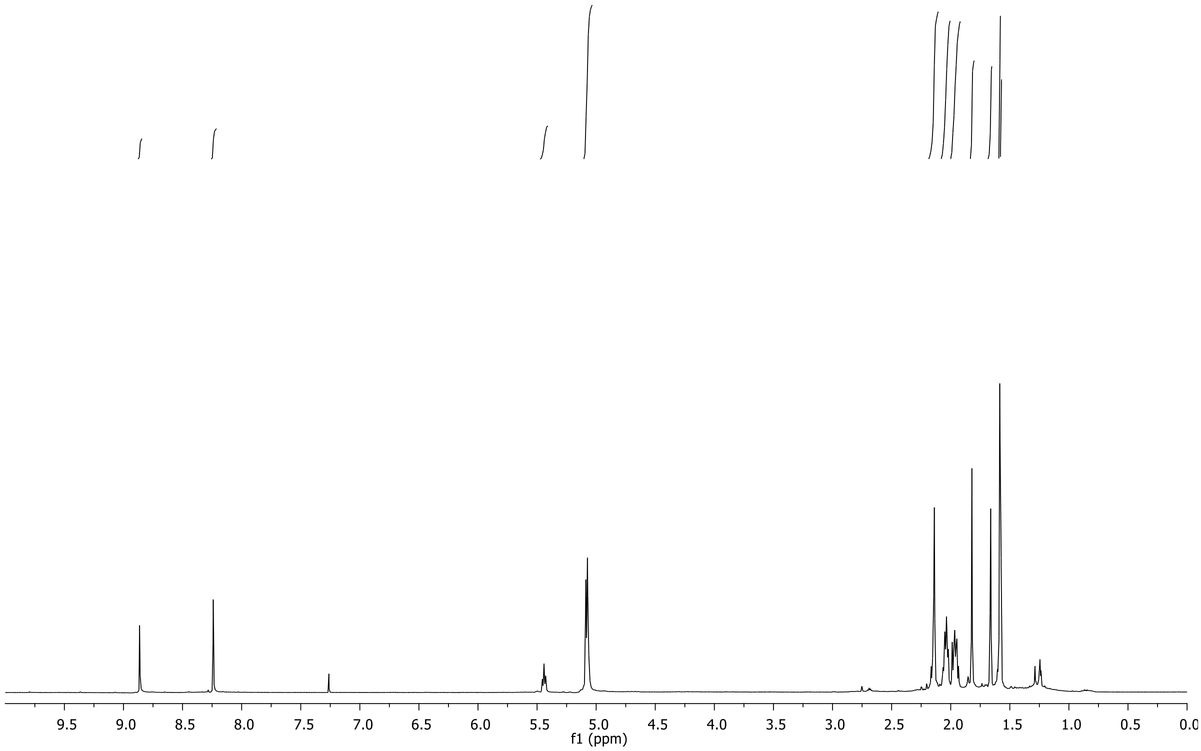

Figure S41. ^{1}H NMR spectrum (500 MHz, $CDCl_3$) of **20**.

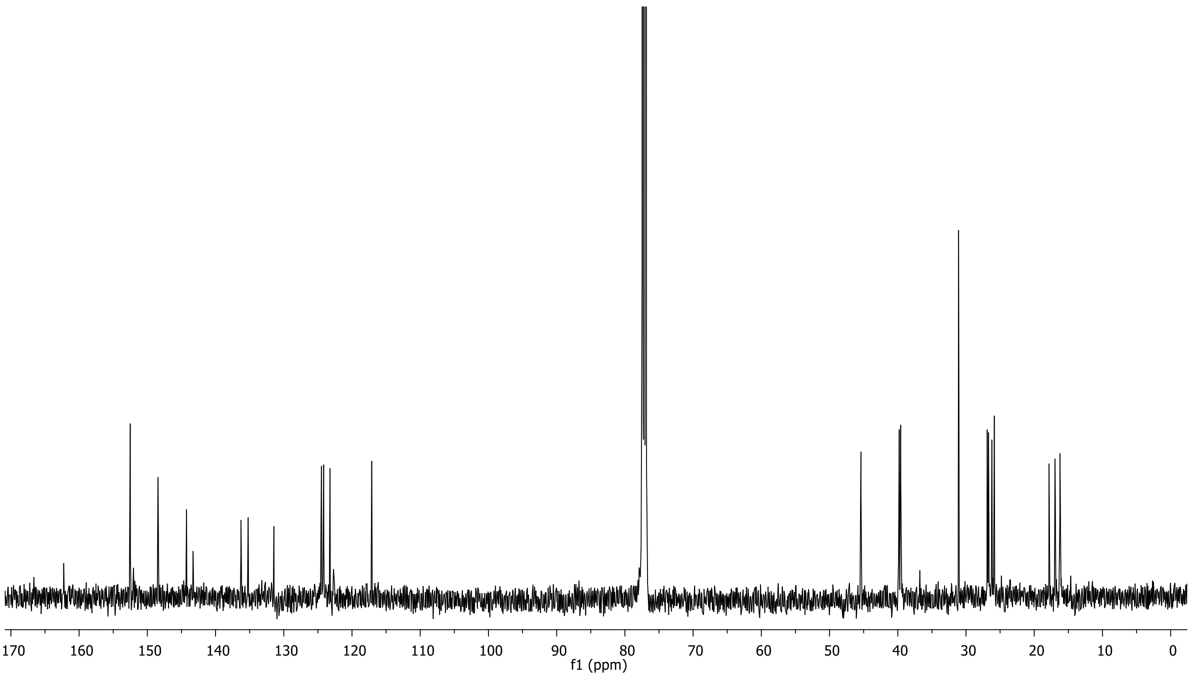

Figure S42. ^{13}C NMR spectrum (150 MHz, $CDCl_3$) of **20**.

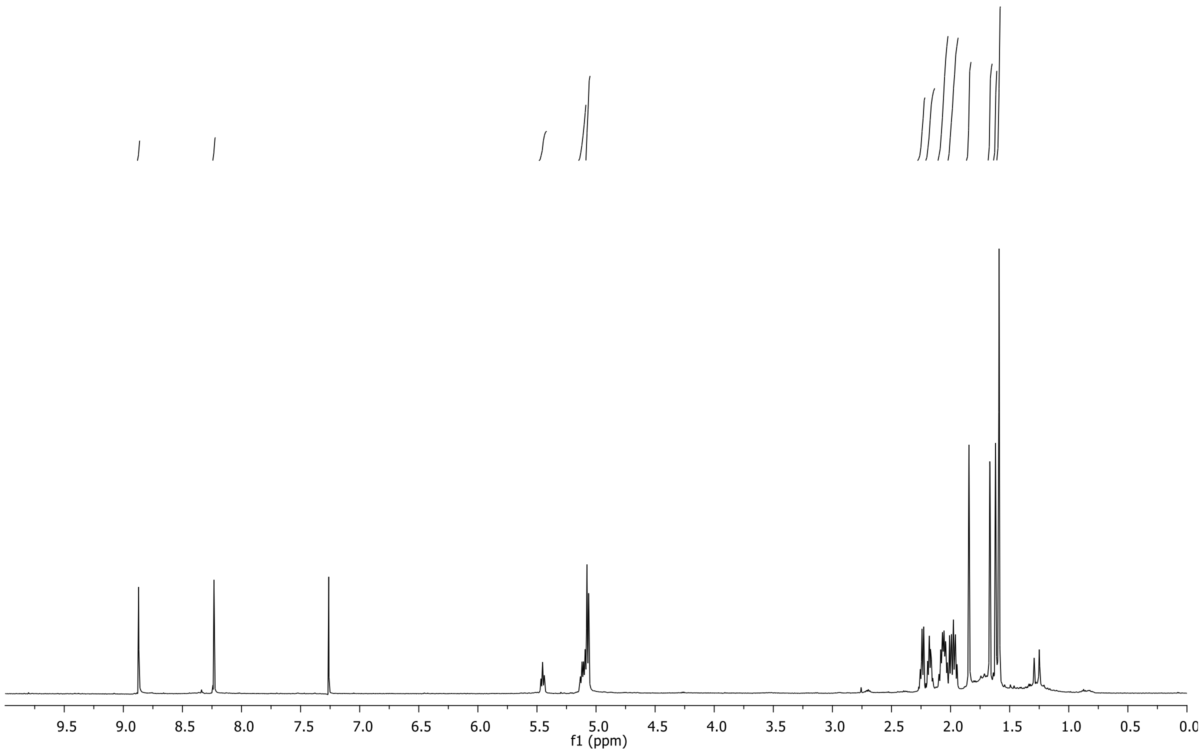

Figure S43. ^{1}H NMR spectrum (500MHz, $CDCl_3$) of **21**.

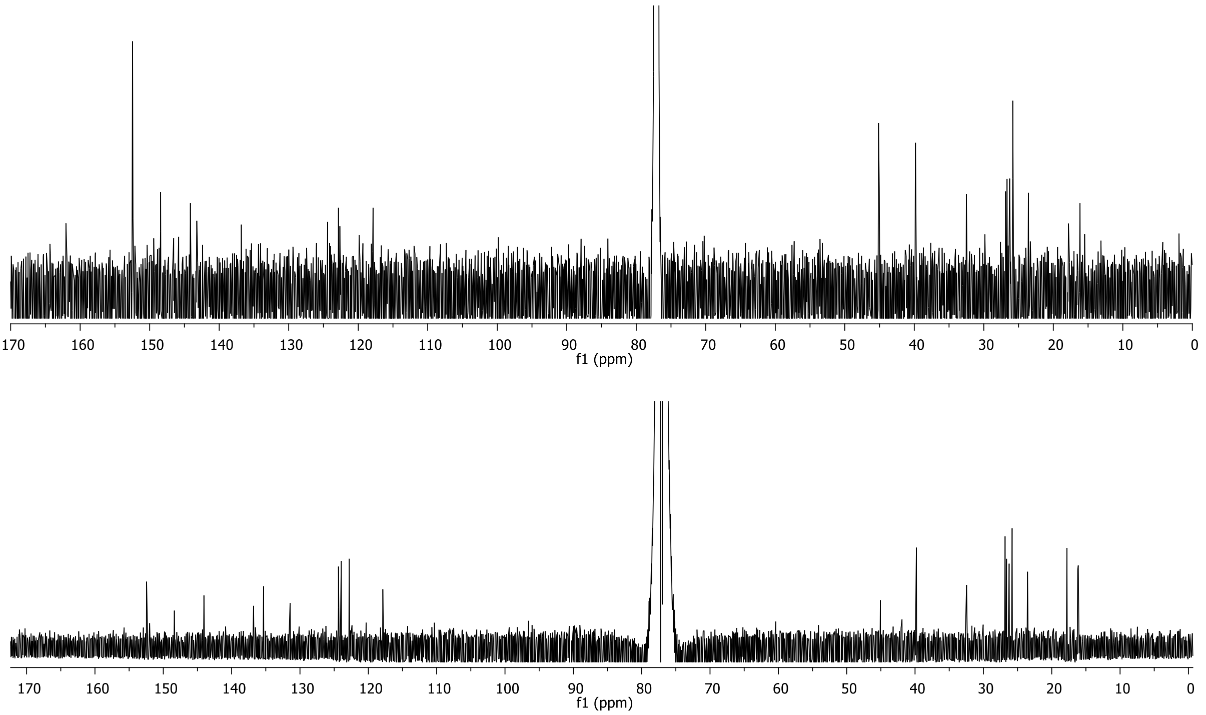

Figure S44. ^{13}C NMR spectra (150 MHz, $CDCl_3$) of **21**, two different samples; top spectrum shows δ 162.1 ppm which is low intensity on the bottom spectrum.

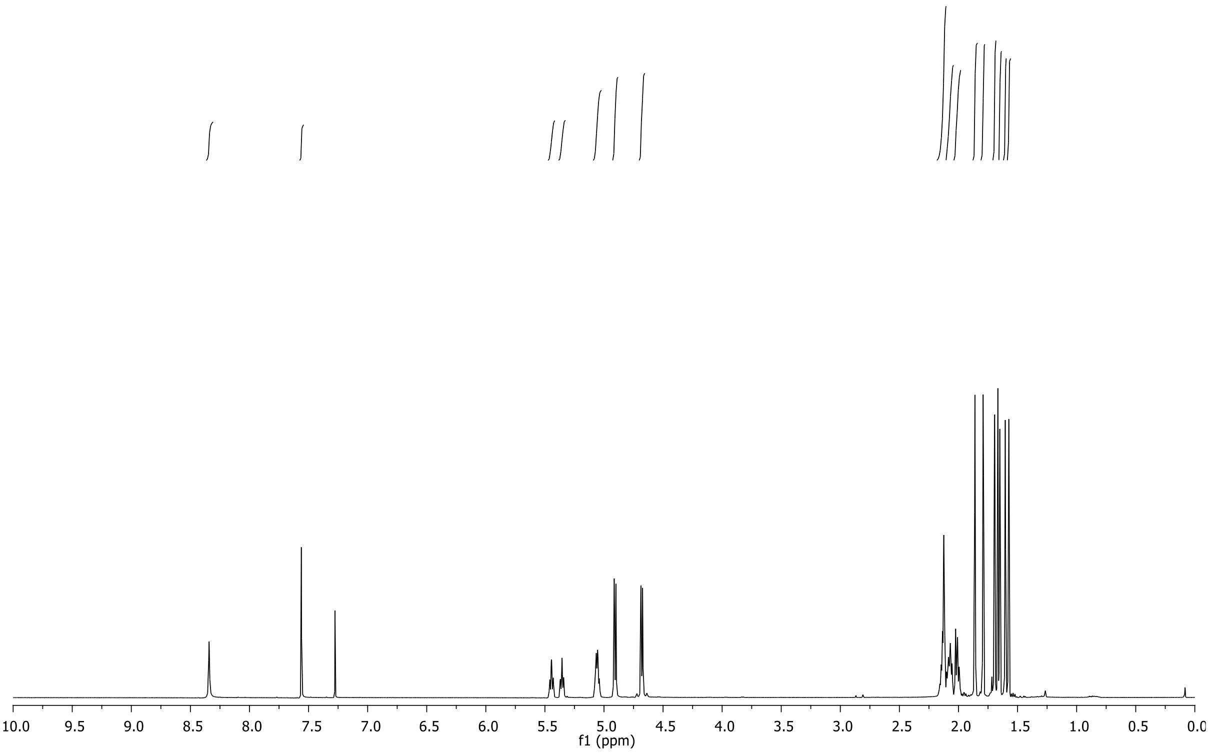

Figure S45. ^{1}H NMR spectrum (600 MHz, $CDCl_3$) of **27**.

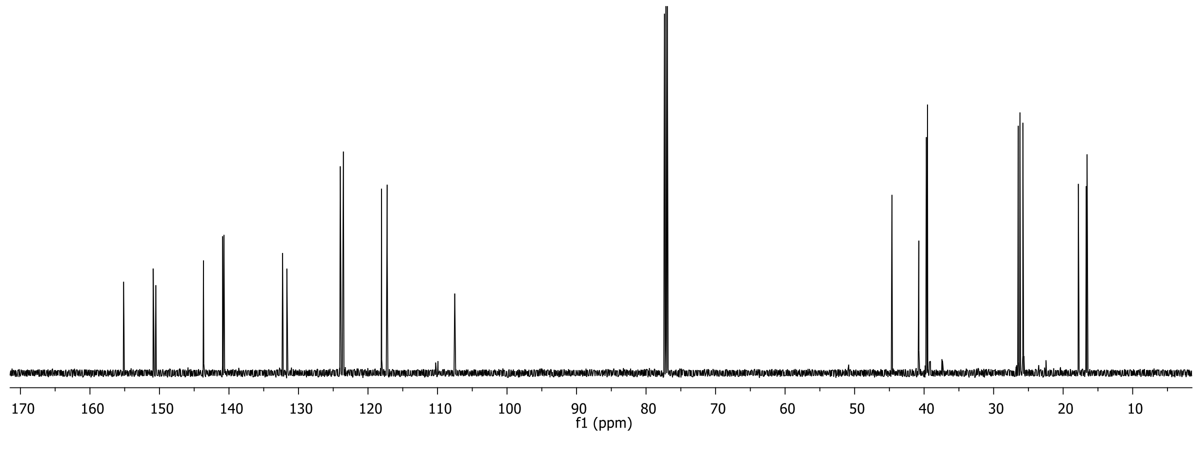

Figure S46. ^{13}C NMR spectrum (150 MHz, $CDCl_3$) of **27**.

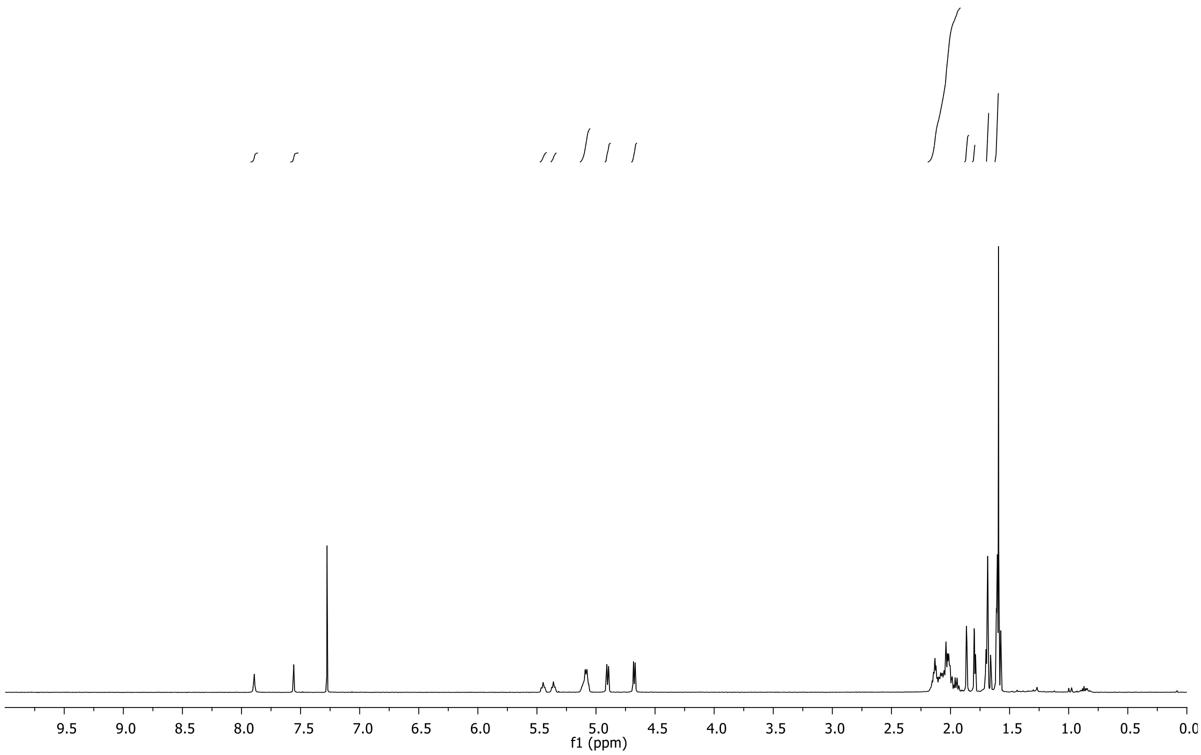

Figure S47. ^{1}H NMR spectrum (600 MHz, $CDCl_3$) of **28**.

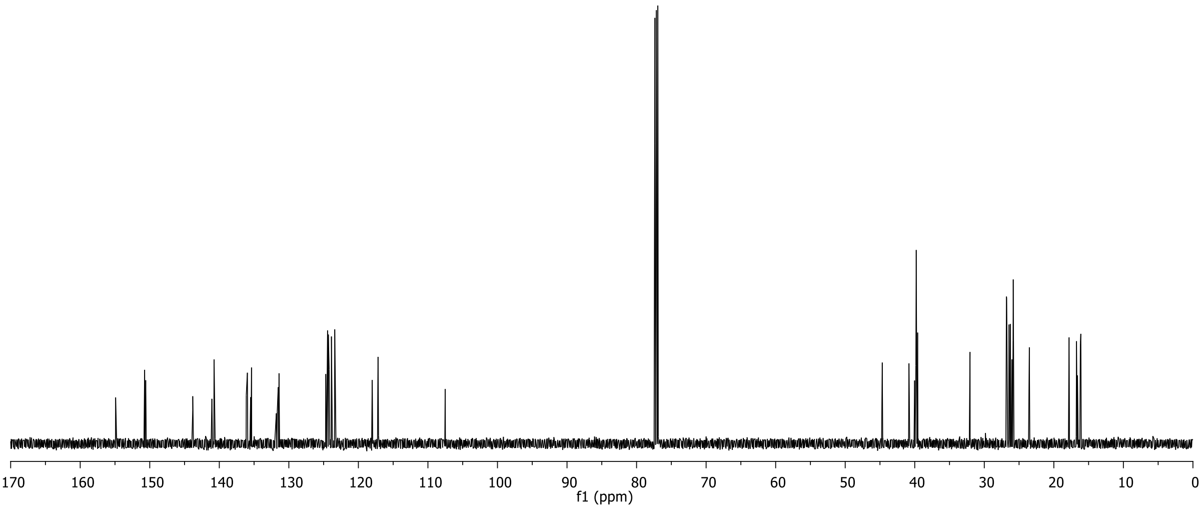

Figure S48. ^{13}C NMR spectrum (150 MHz, $CDCl_3$) of **28**.

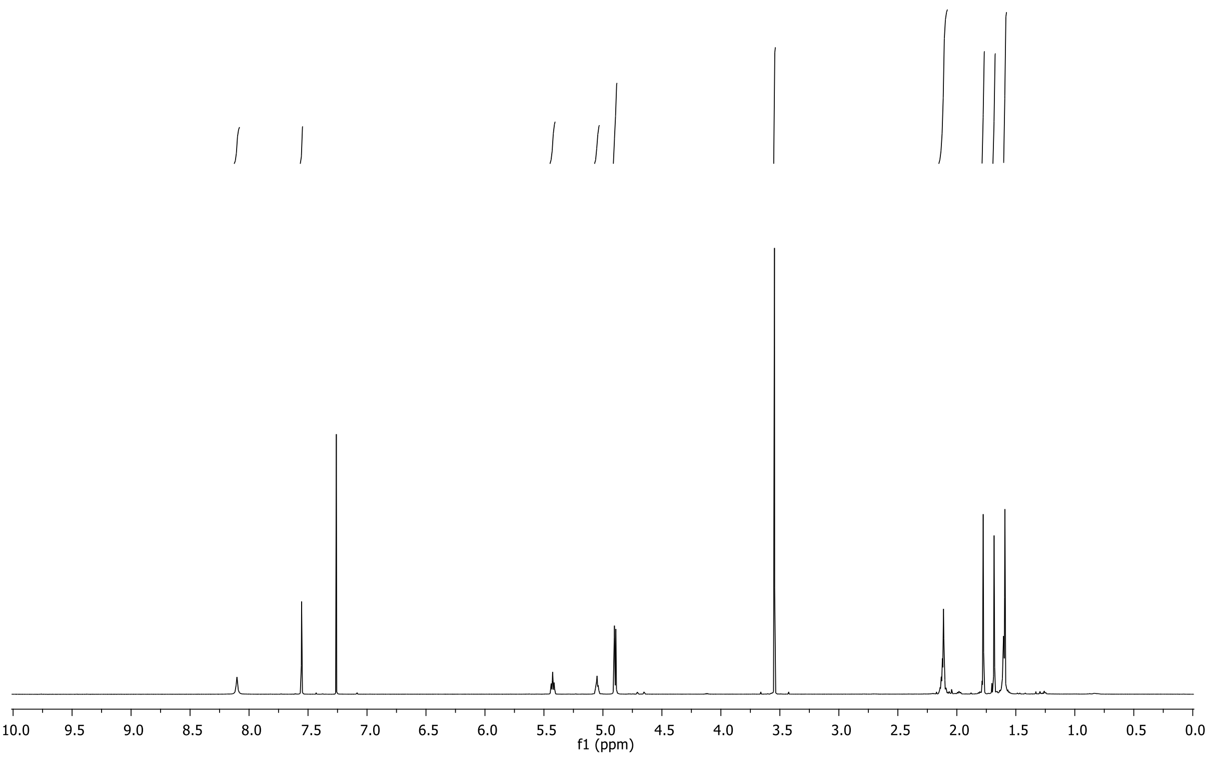

Figure S49. ^{1}H NMR spectrum (600 MHz, $CDCl_3$) of **29**.

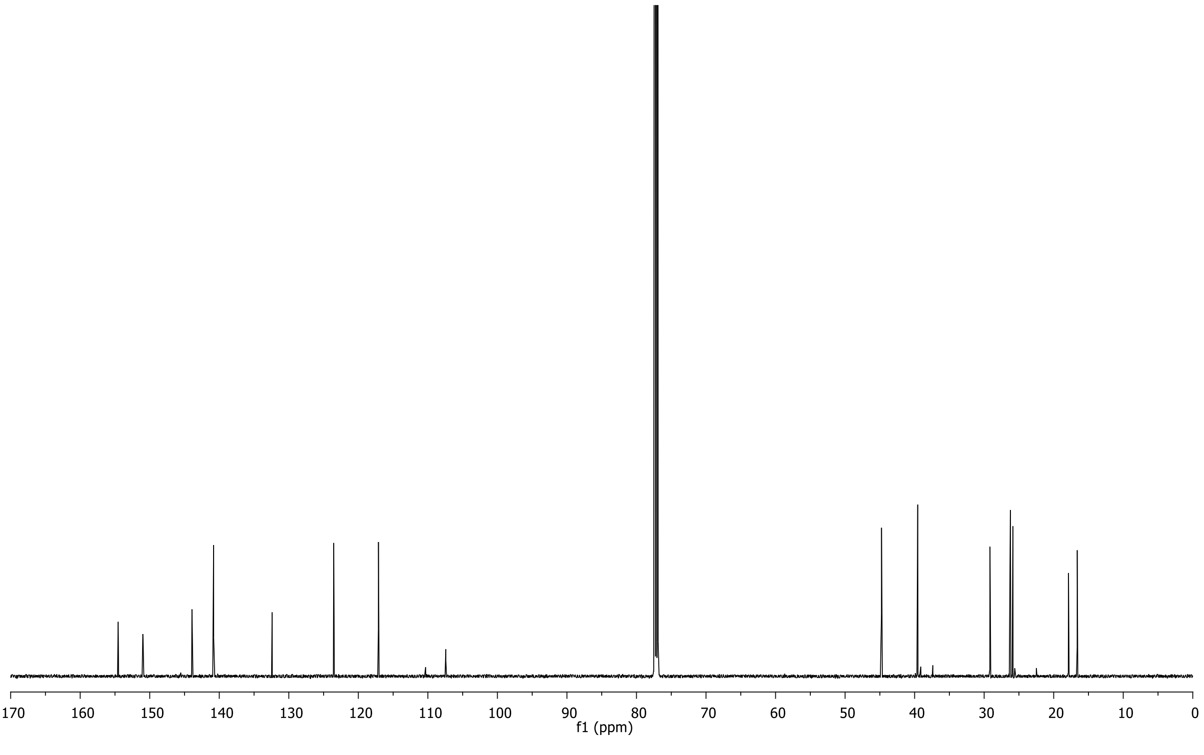

Figure S50. ^{13}C NMR spectrum (150 MHz, $CDCl_3$) of **29**.

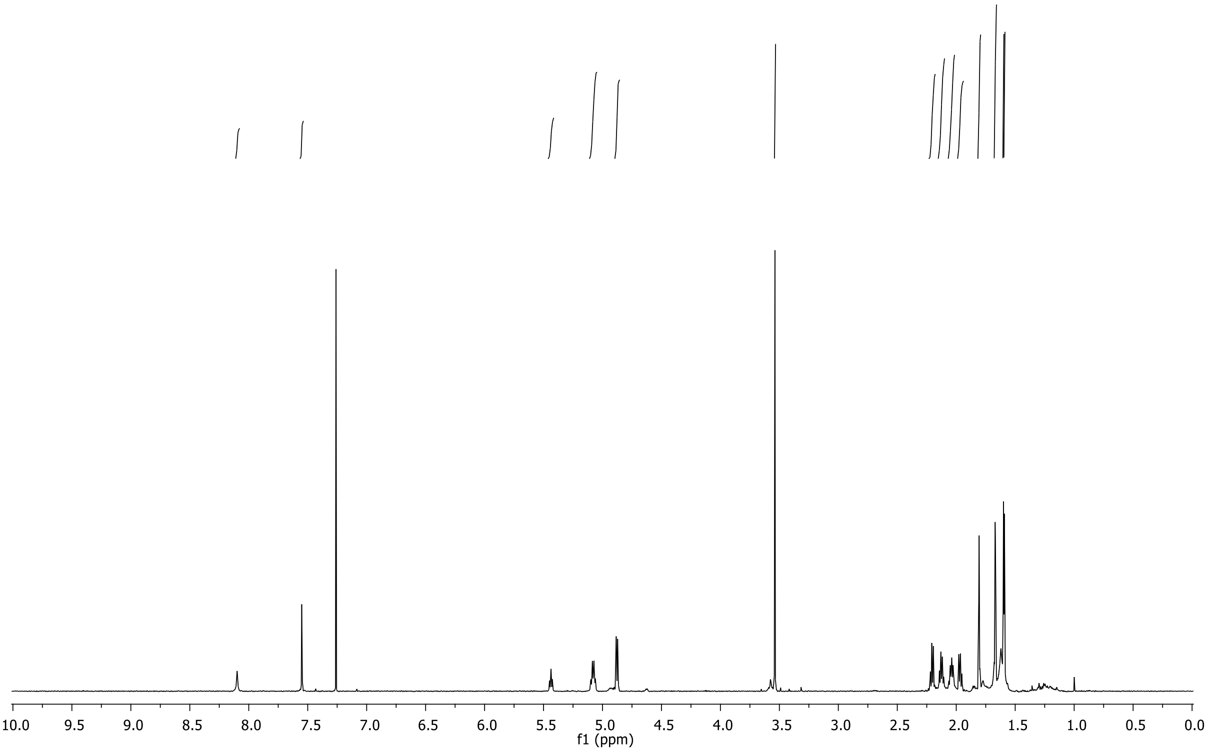

Figure S51. ^{1}H NMR spectrum (600 MHz, $CDCl_3$) of **30**.

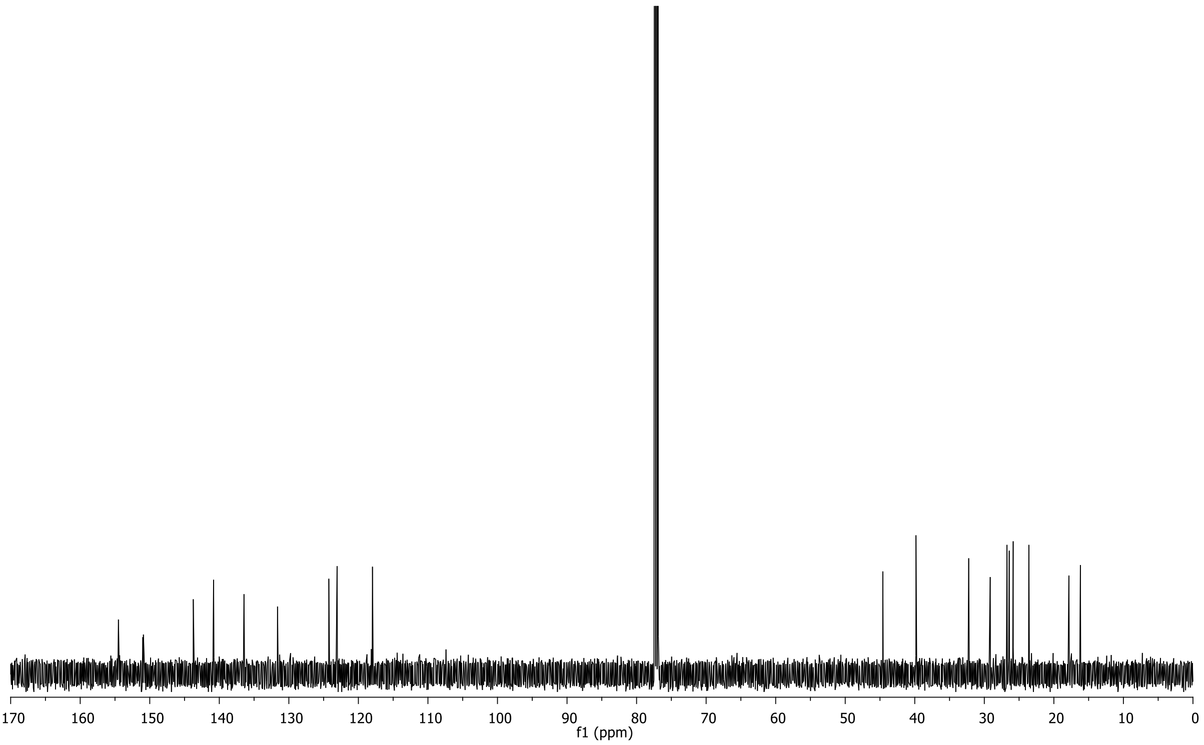

Figure S52. ^{13}C NMR spectrum (150 MHz, $CDCl_3$) of **30**.

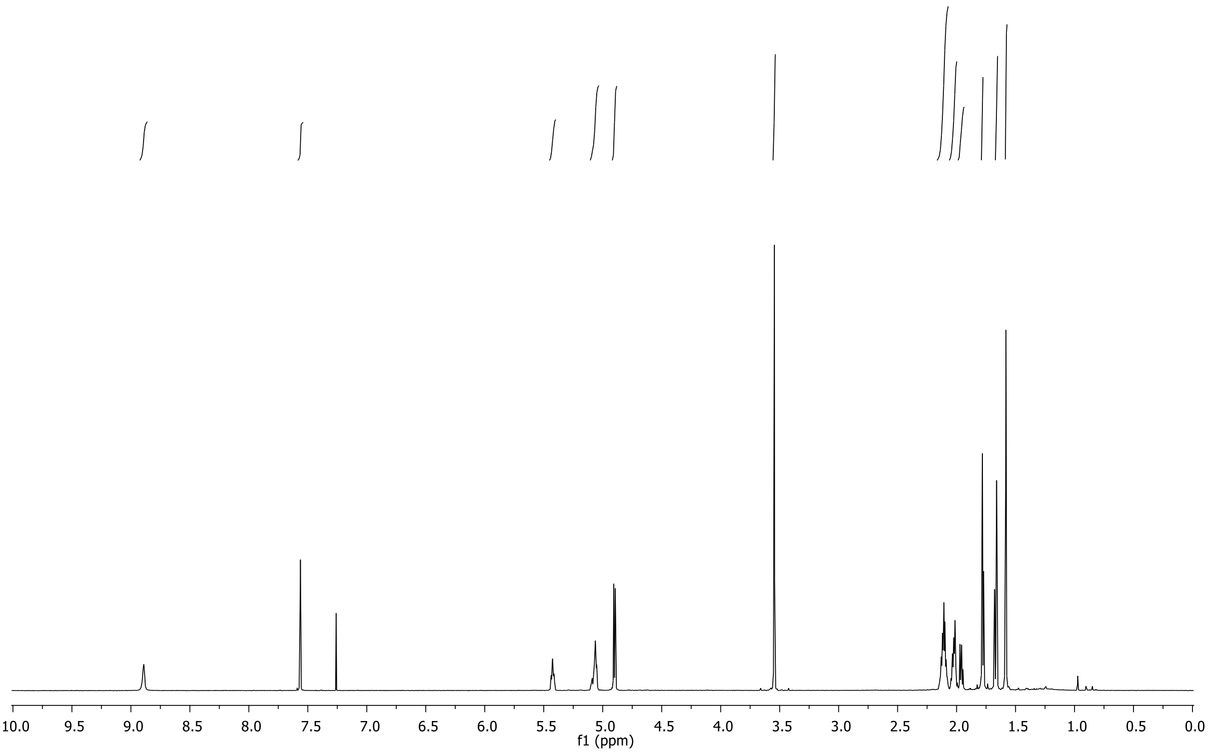

Figure S53. ^{1}H NMR spectrum (600 MHz, $CDCl_3$) of **31**.

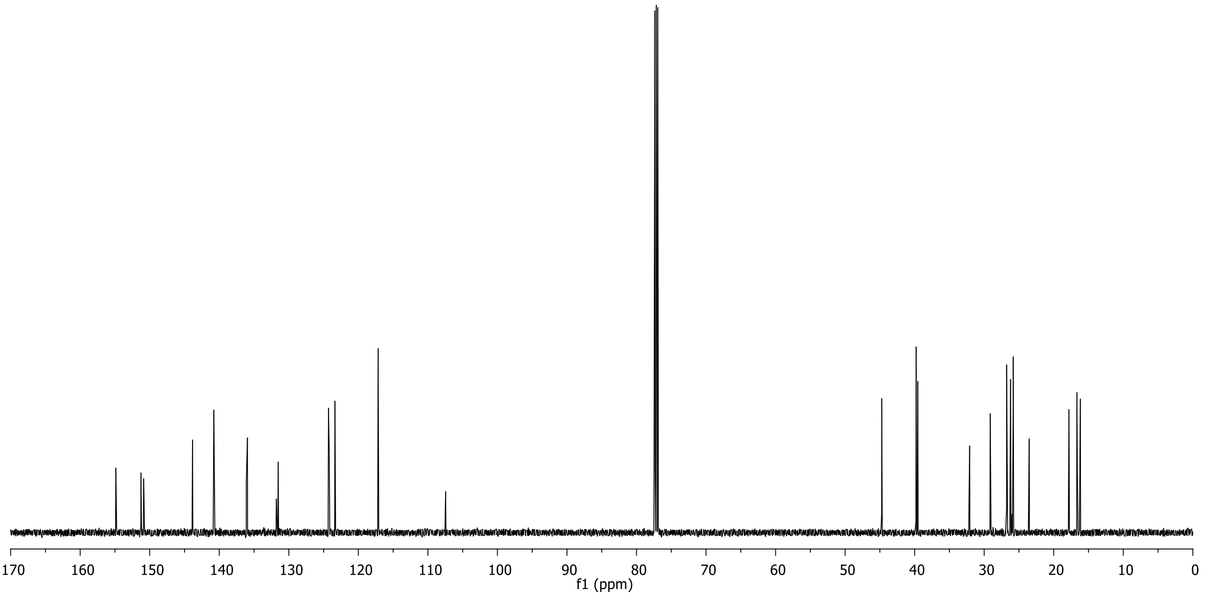

Figure S54. ^{13}C NMR spectrum (150 MHz, $CDCl_3$) of **31**.

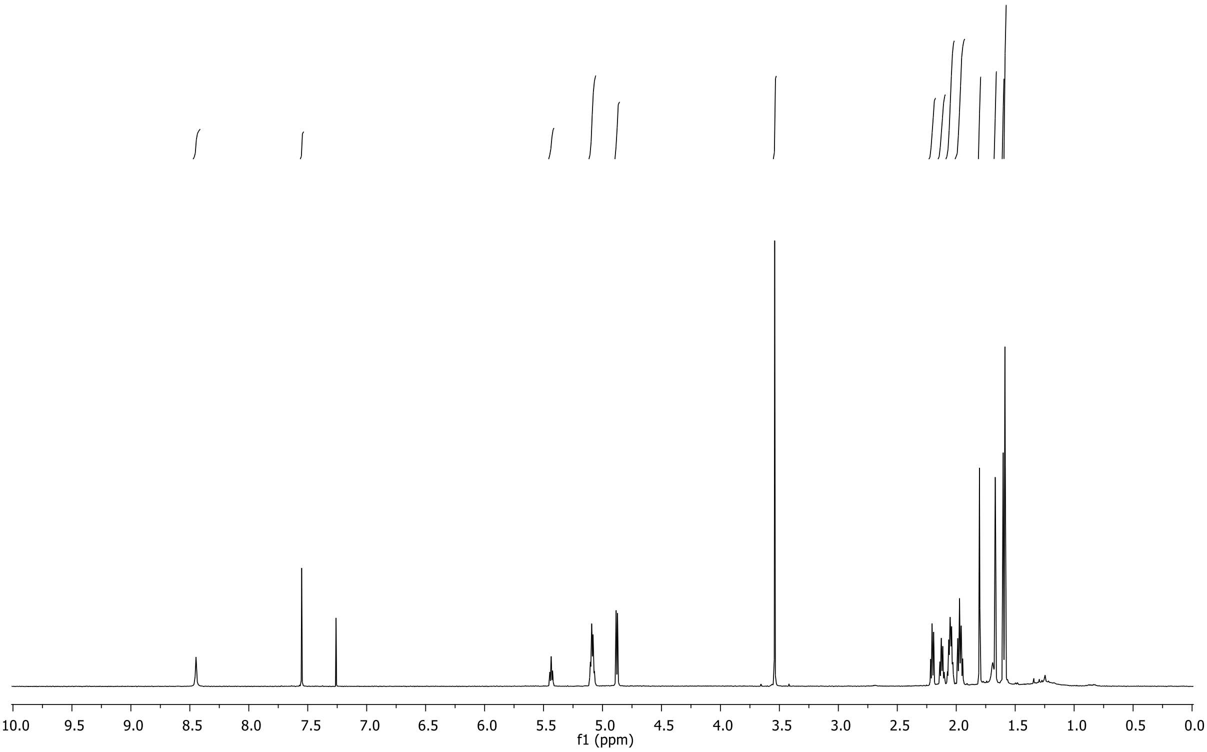

Figure S55. ^{1}H NMR spectrum (600 MHz, $CDCl_3$) of **32**.

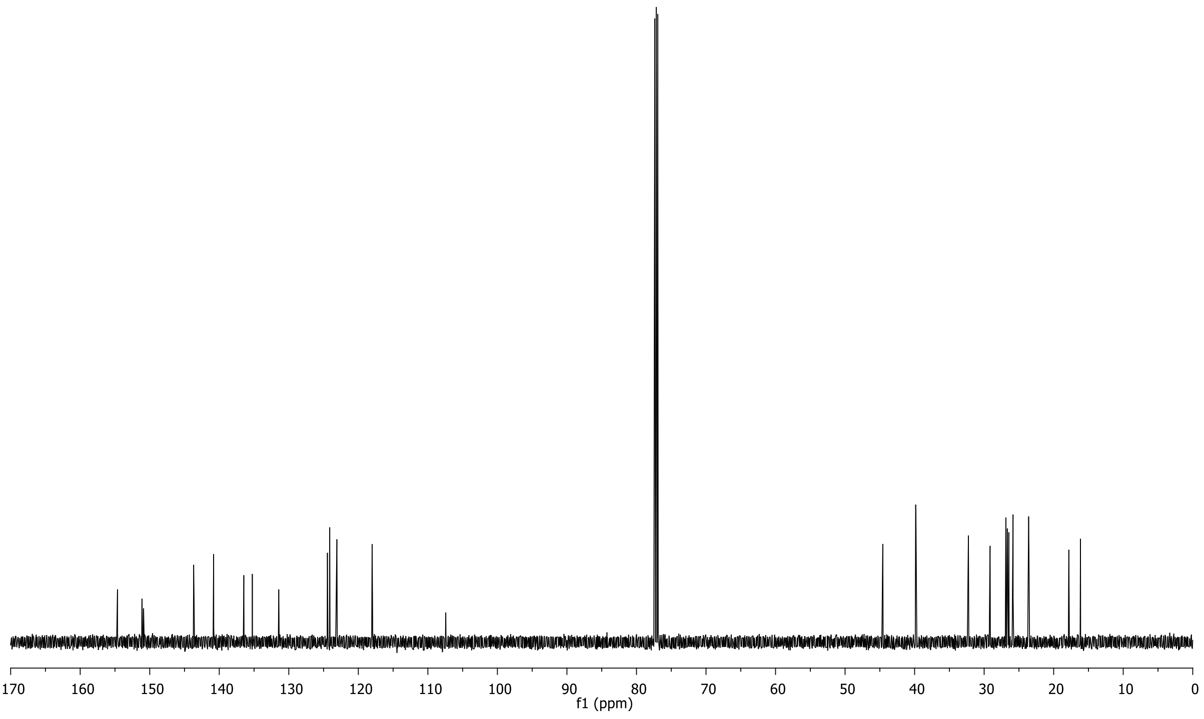

Figure S56. ^{13}C NMR spectrum (150 MHz, $CDCl_3$) of **32**.

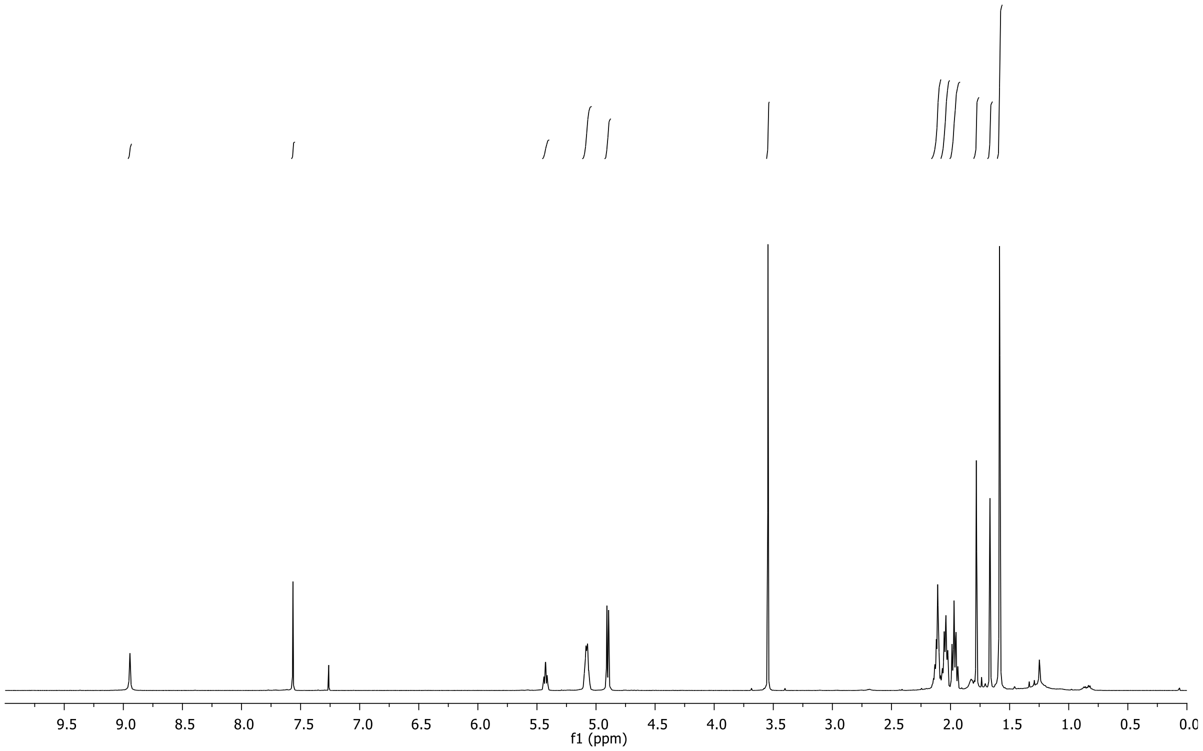

Figure S57. ^{1}H NMR spectrum (500 MHz, $CDCl_3$) of **33**.

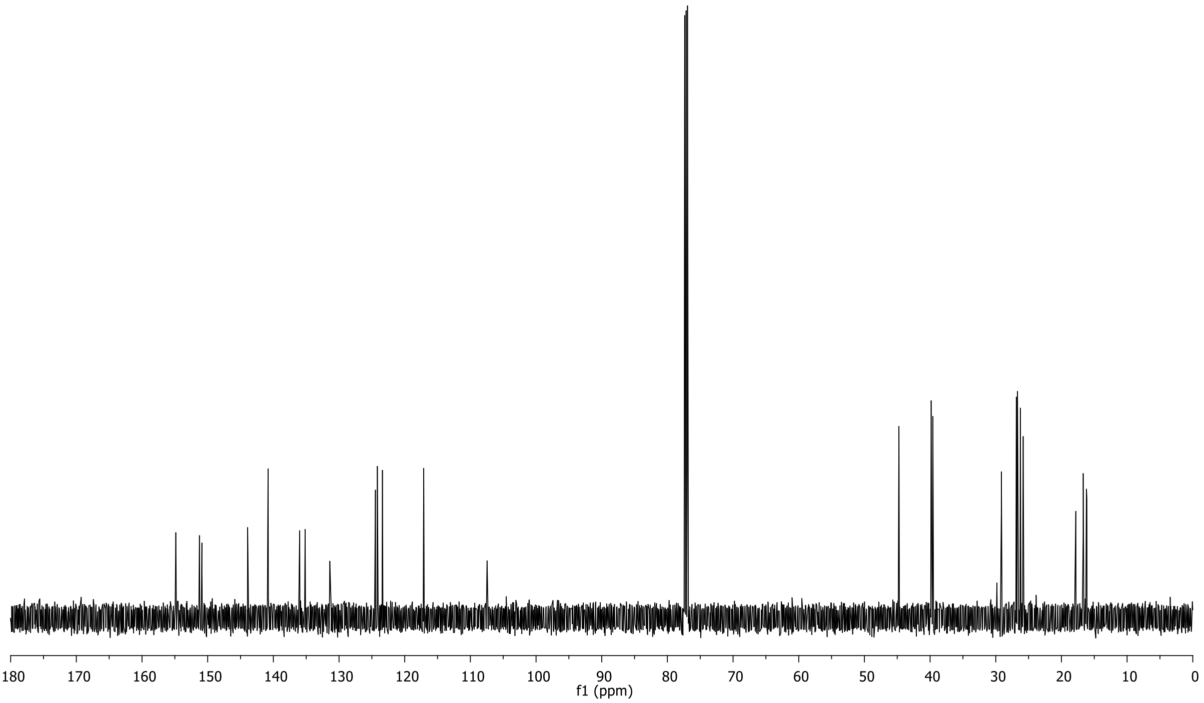

Figure S58. ^{13}C NMR spectrum (150 MHz, $CDCl_3$) of **33**.

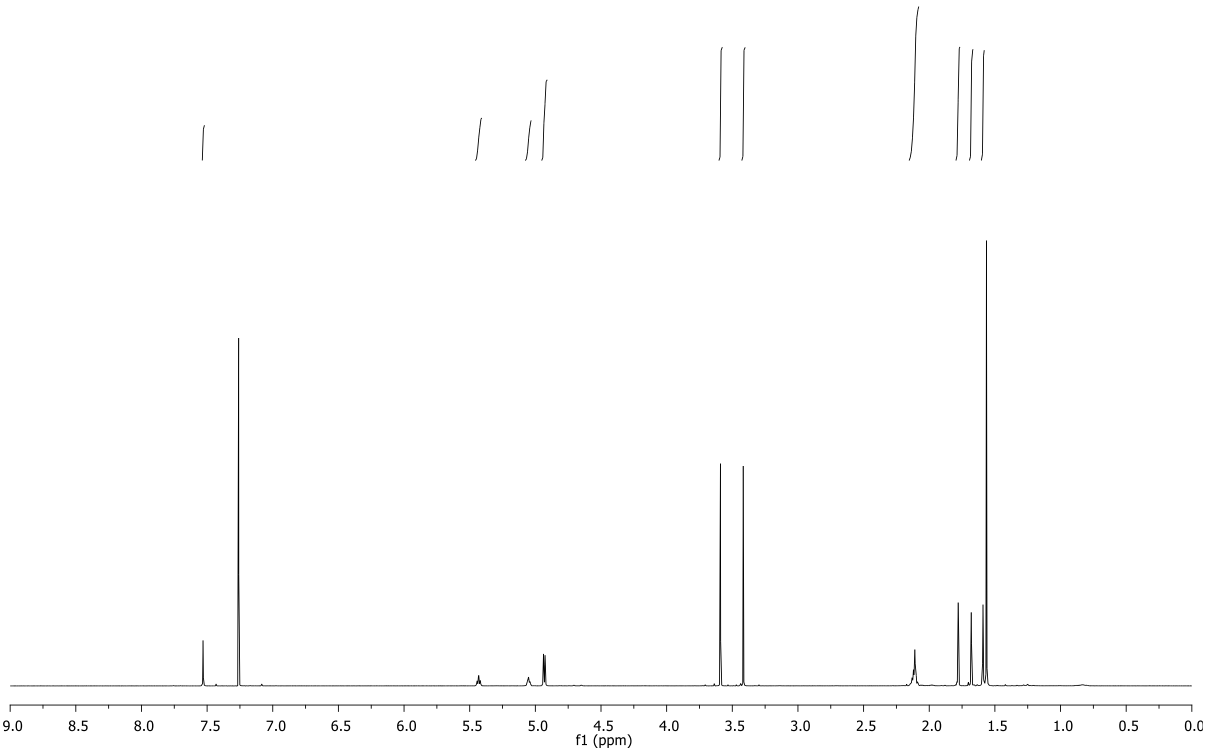

Figure S59. ^{1}H NMR spectrum (600 MHz, $CDCl_3$) of **34**.

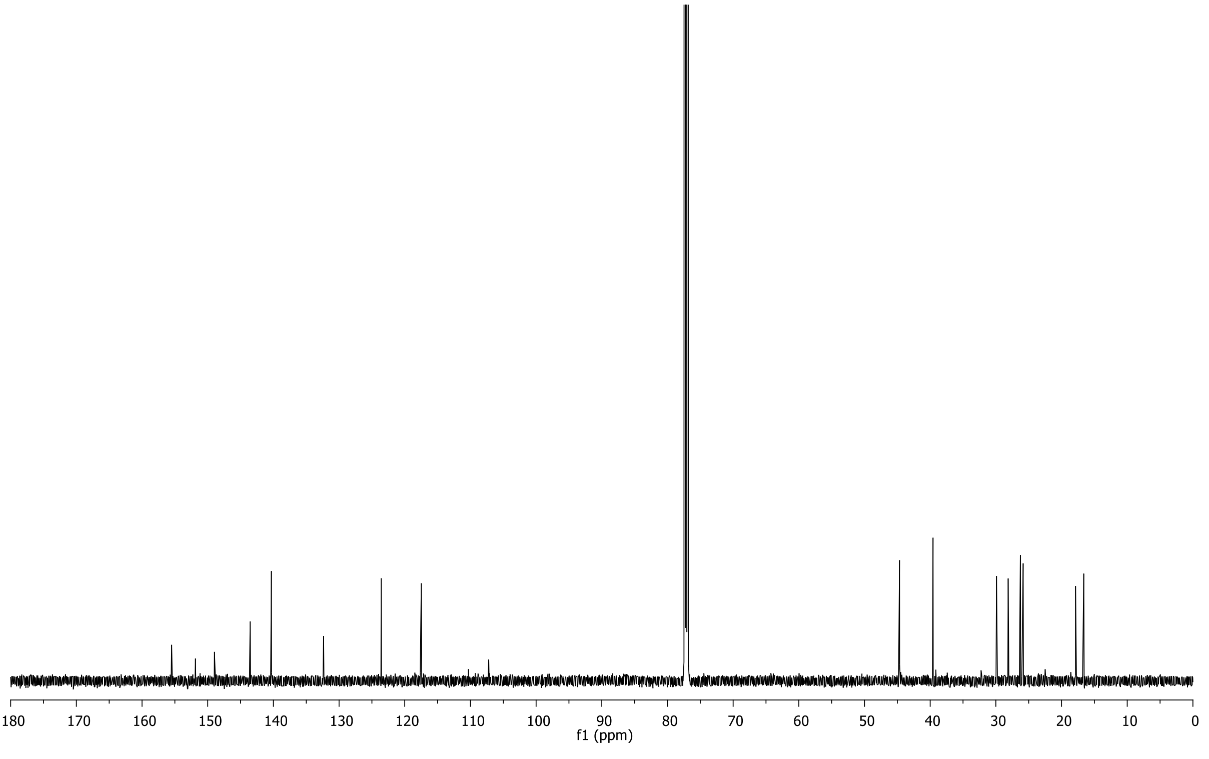

Figure S60. ^{13}C NMR spectrum (150 MHz, $CDCl_3$) of **34**.

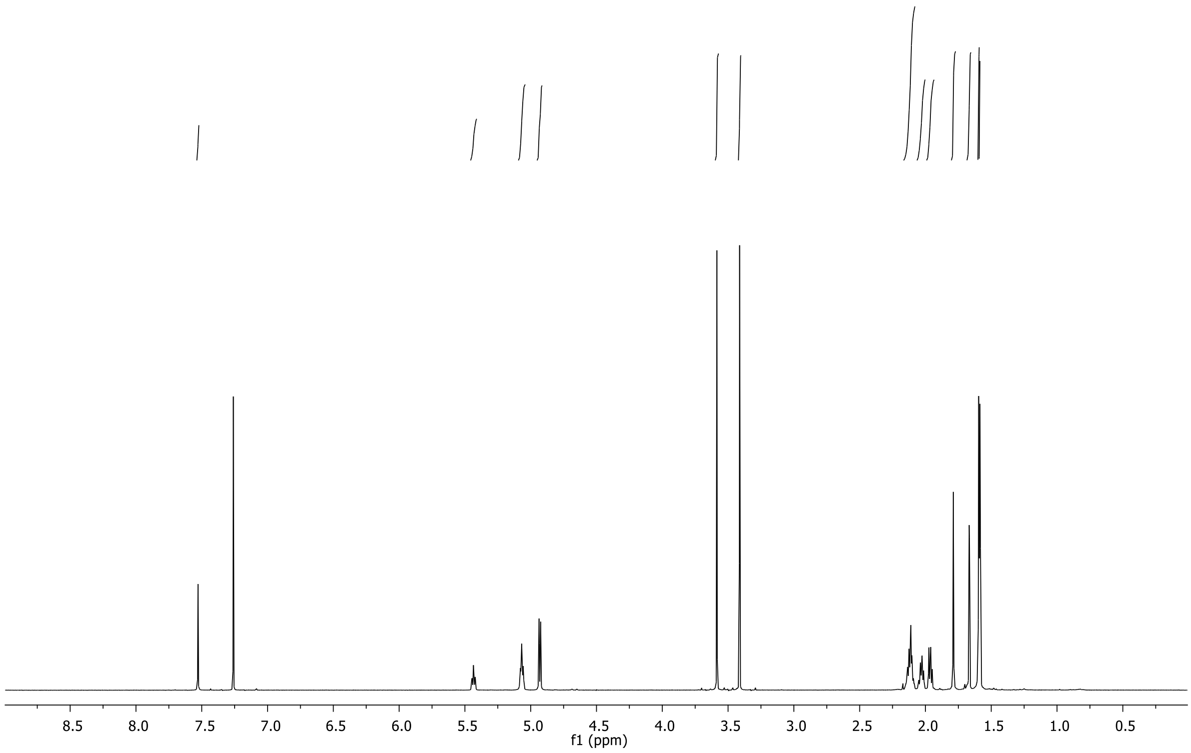

Figure S61. ^{1}H NMR spectrum (600 MHz, $CDCl_3$) of **35**.

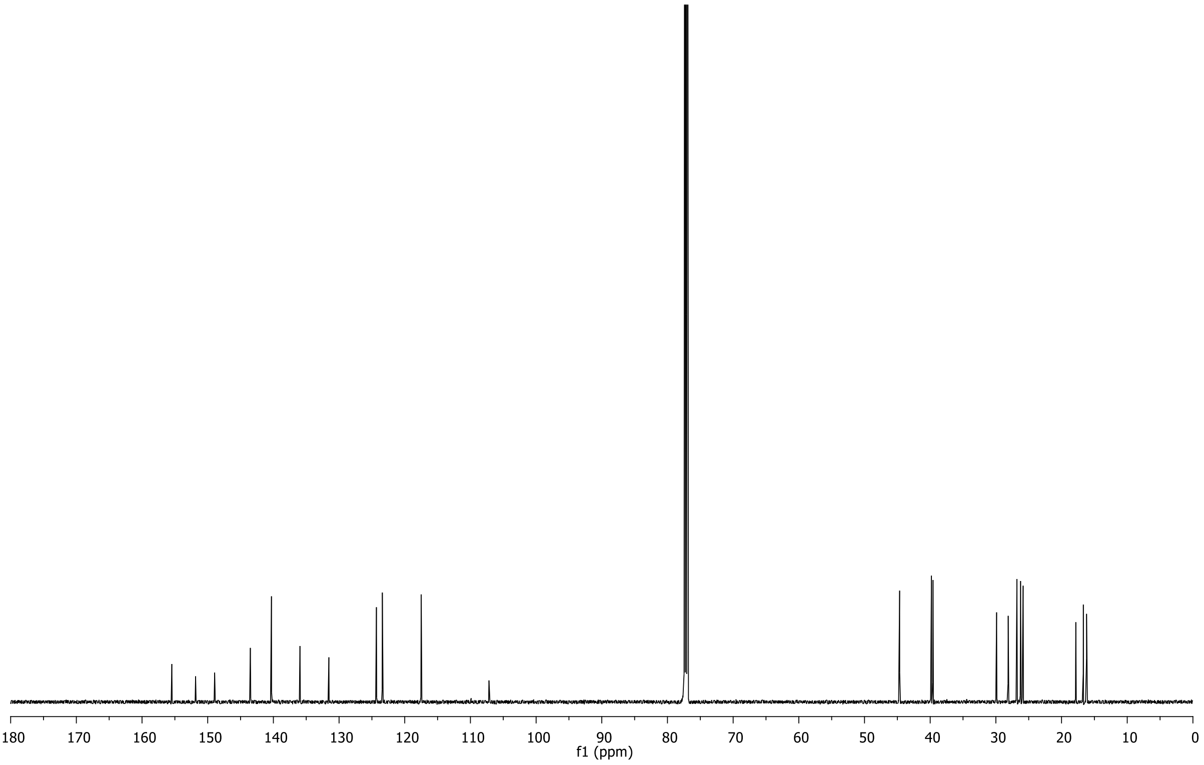

Figure S62. ^{13}C NMR spectrum (150 MHz, $CDCl_3$) of **35**.

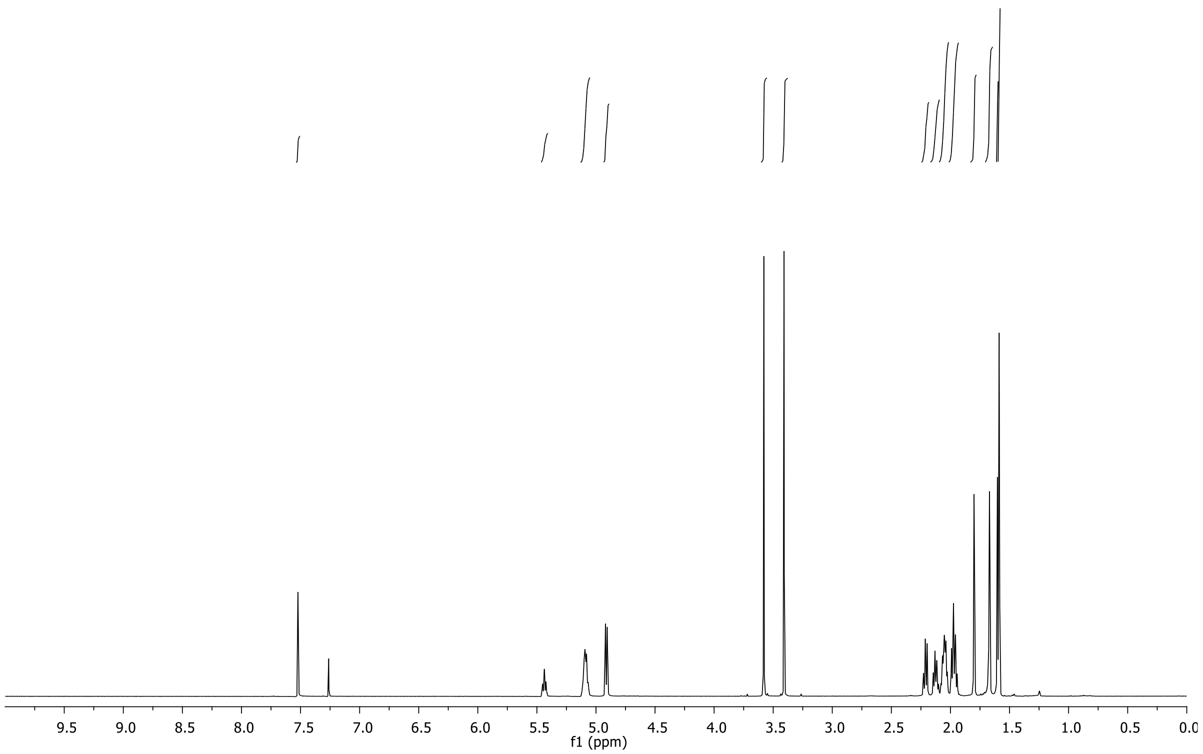

Figure S63. ^1H NMR spectrum (500 MHz, $CDCl_3$) of **36**.

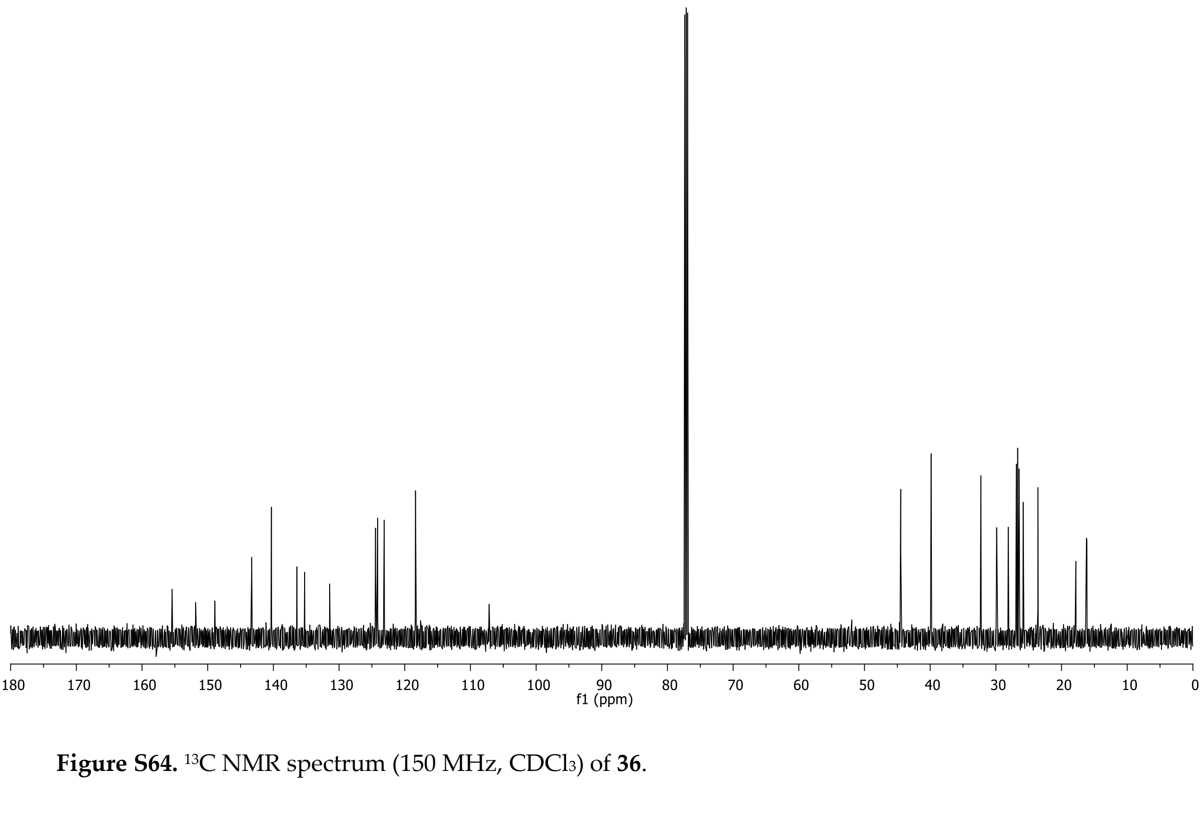

Figure S64. ^{13}C NMR spectrum (150 MHz, $CDCl_3$) of **36**.

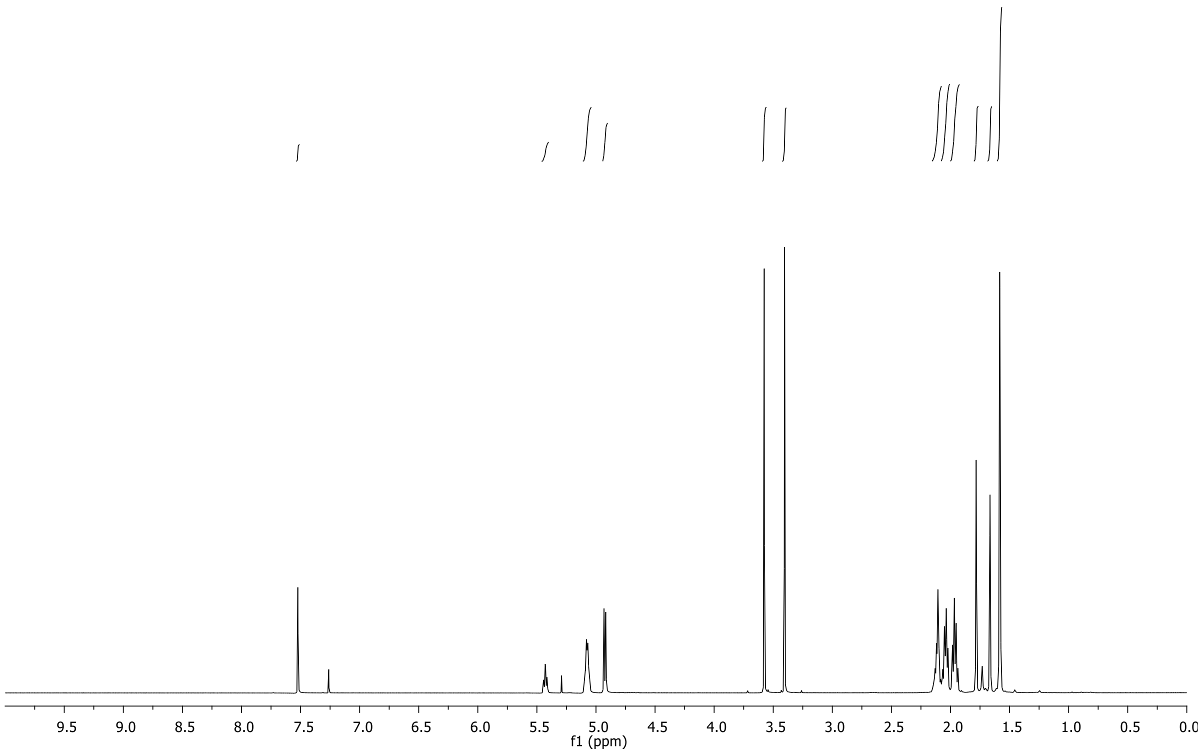

Figure S65. ^{1}H NMR spectrum (500 MHz, $CDCl_3$) of **37**.

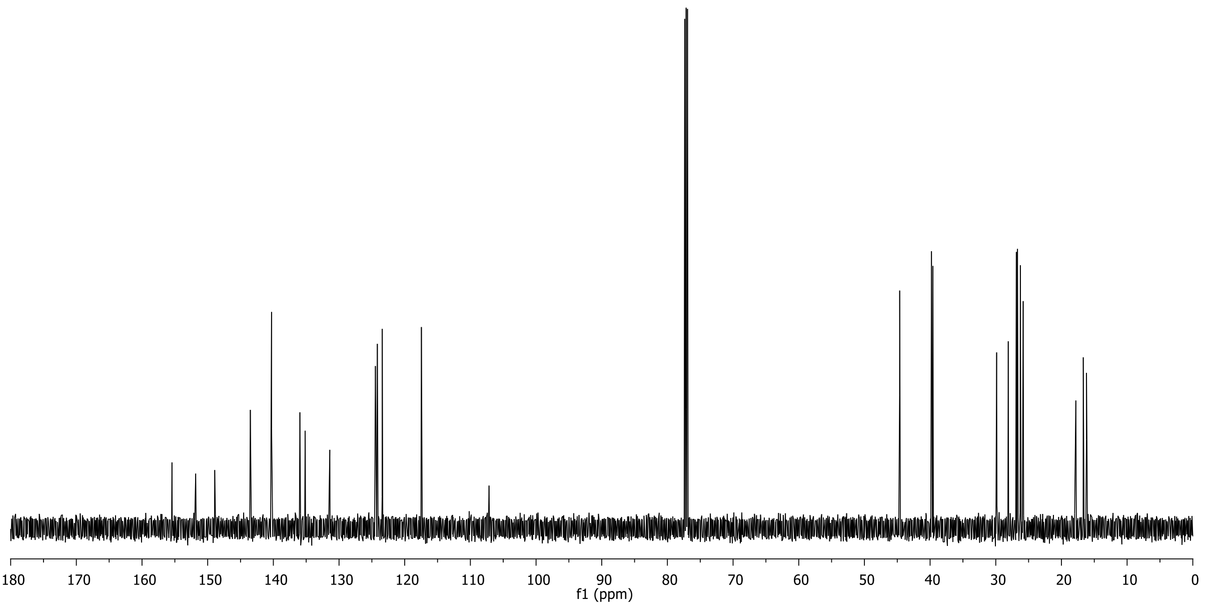

Figure S66. ^{13}C NMR spectrum (150 MHz, $CDCl_3$) of **37**.

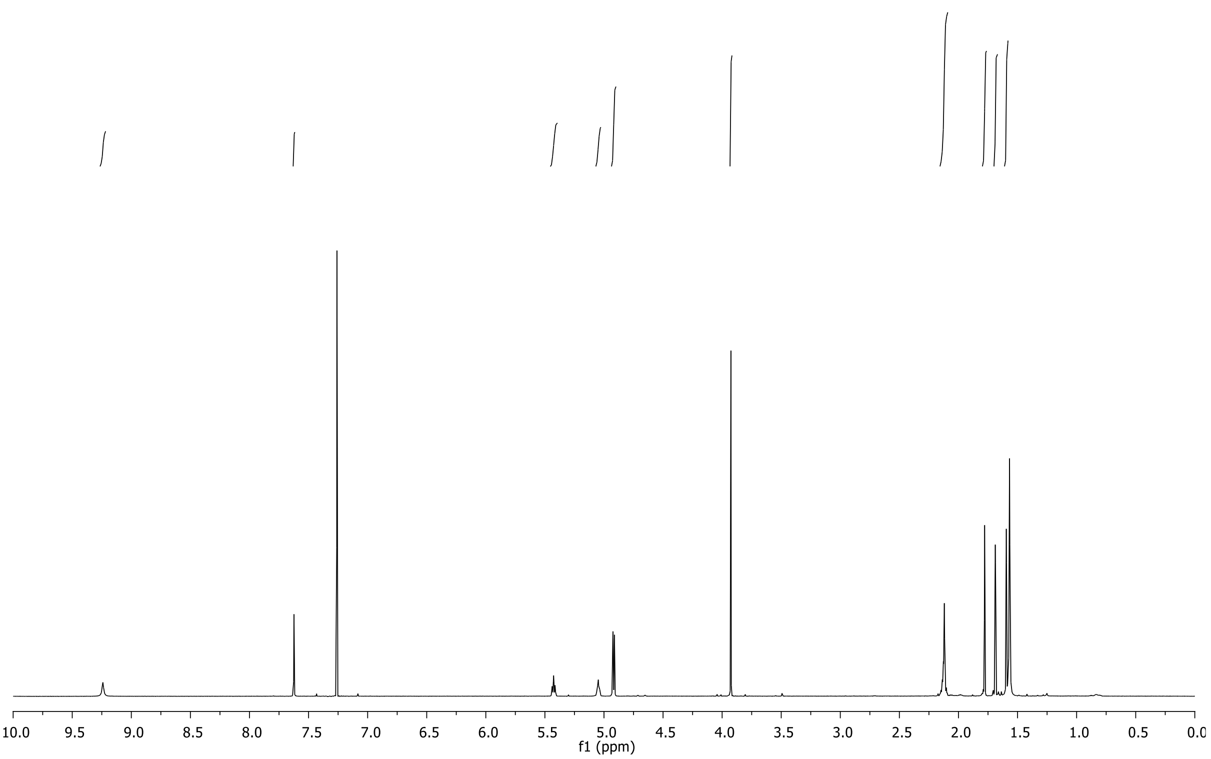

Figure S67. ^{1}H NMR spectrum (600 MHz, $CDCl_3$) of **38**.

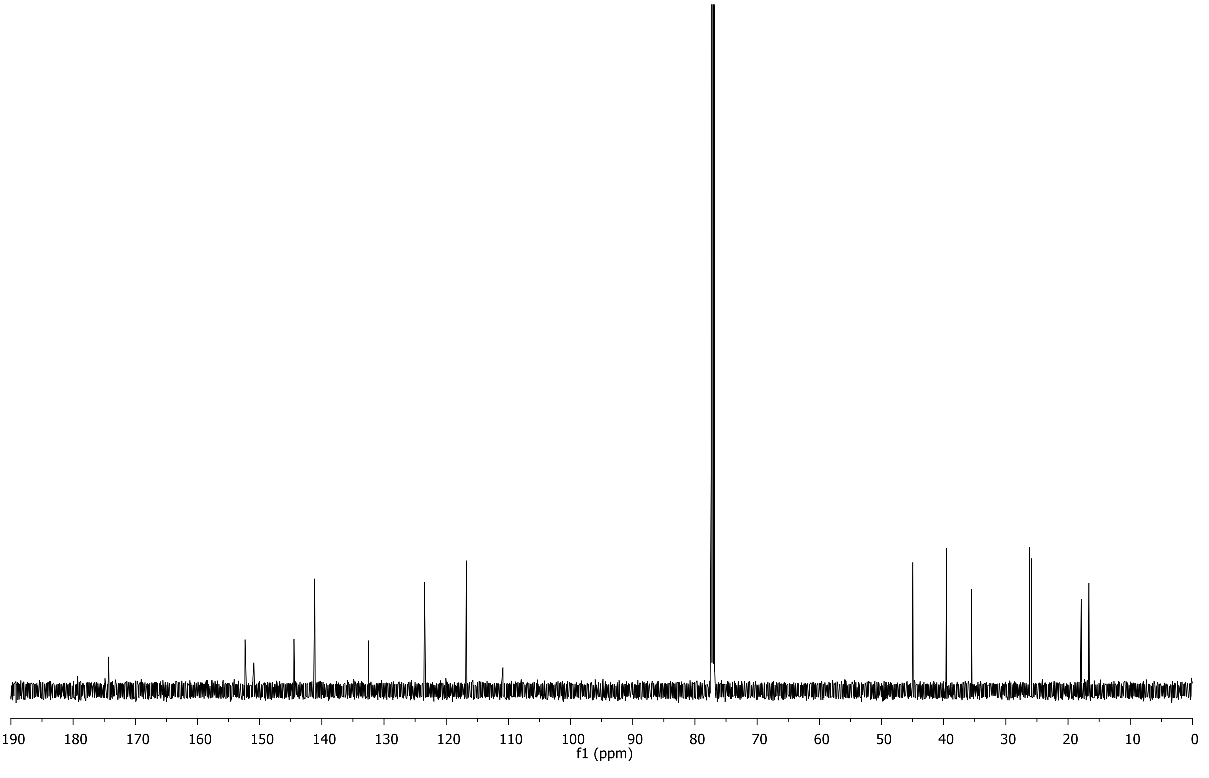

Figure S68. ^{13}C NMR spectrum (150 MHz, $CDCl_3$) of **38**.

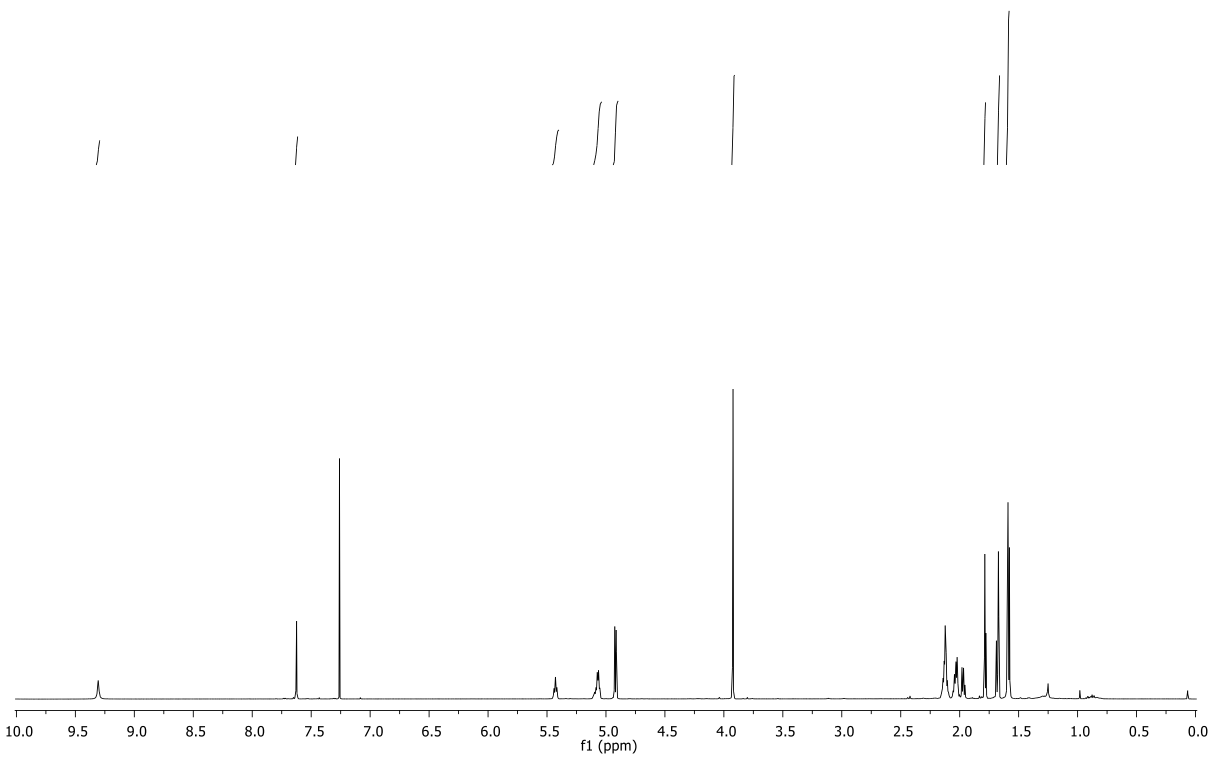

Figure S69. ^{1}H NMR spectrum (600 MHz, $CDCl_3$) of **39**.

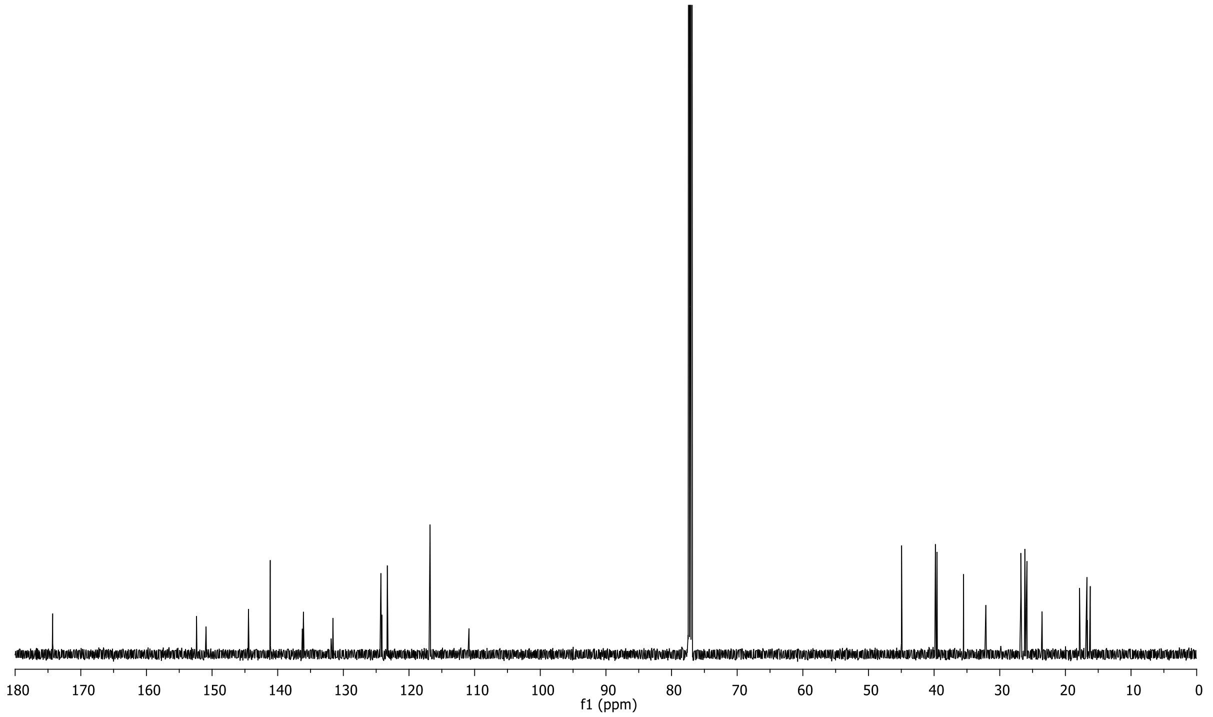

Figure S70. ^{13}C NMR spectrum (150 MHz, $CDCl_3$) of **39**.

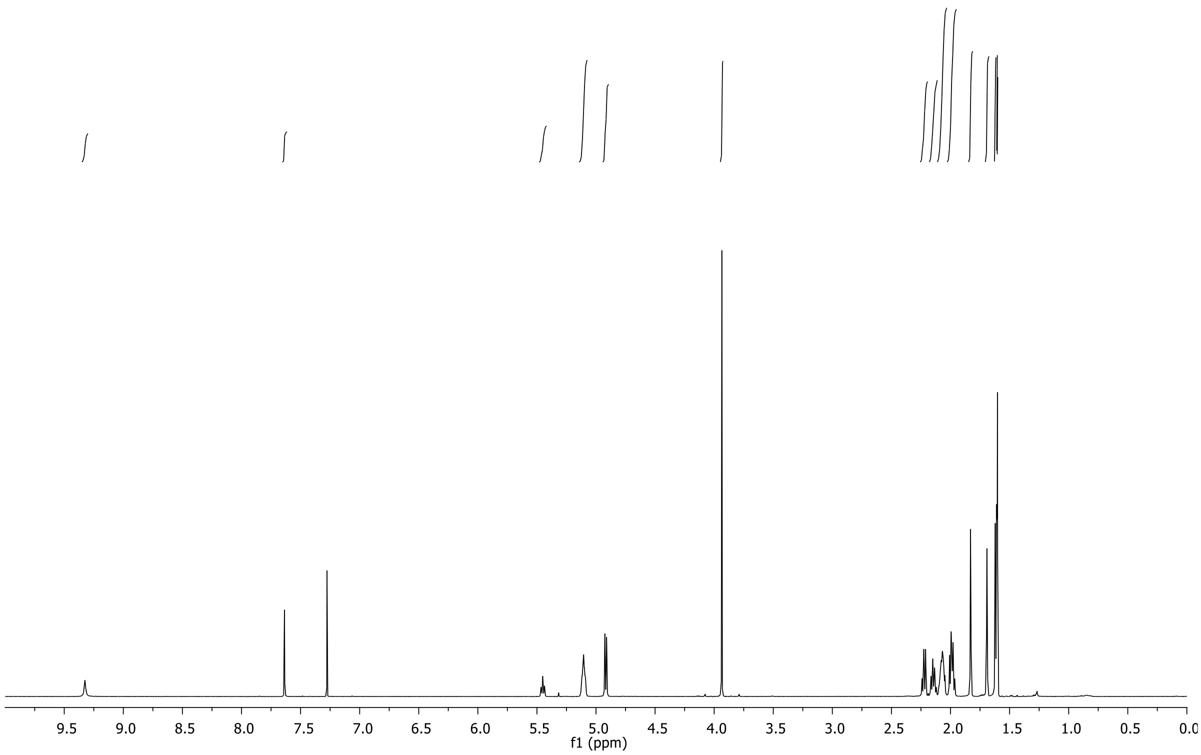

Figure S71. ^{1}H NMR spectrum (500 MHz, $CDCl_3$) of **40**.

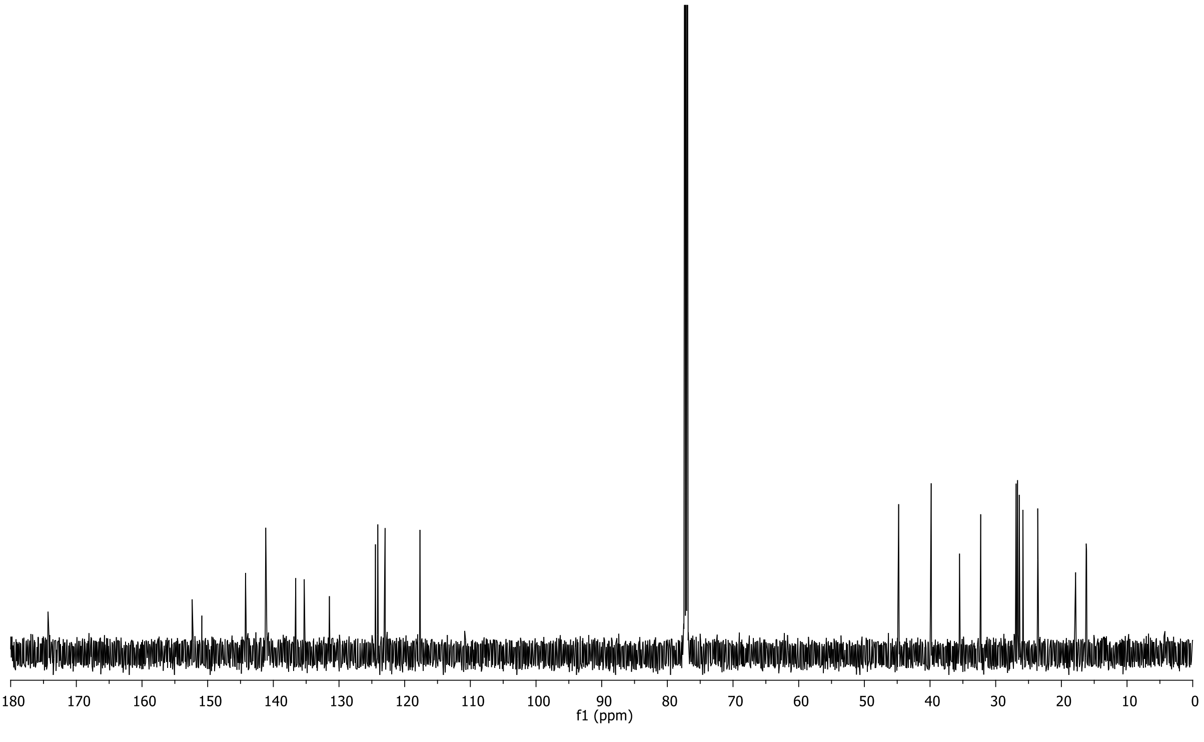

Figure S72. ^{13}C NMR spectrum (150 MHz, $CDCl_3$) of **40**.

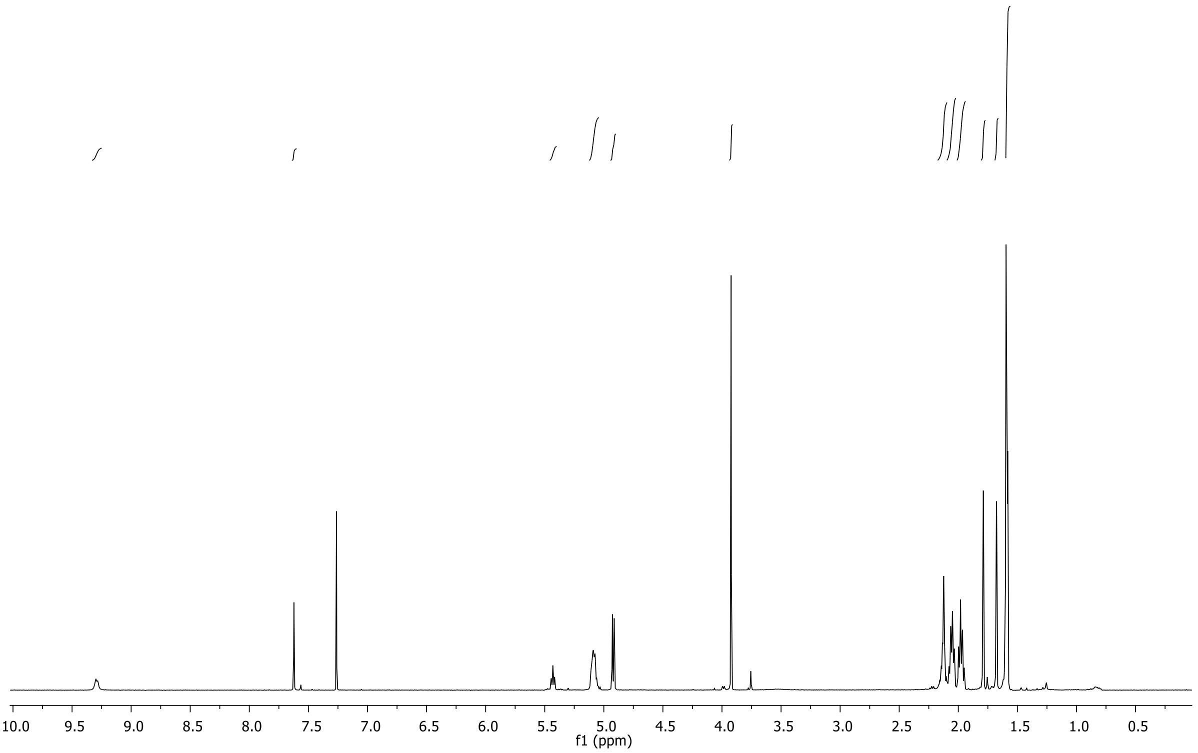

Figure S73. ^{1}H NMR spectrum (500 MHz, $CDCl_3$) of **41**.

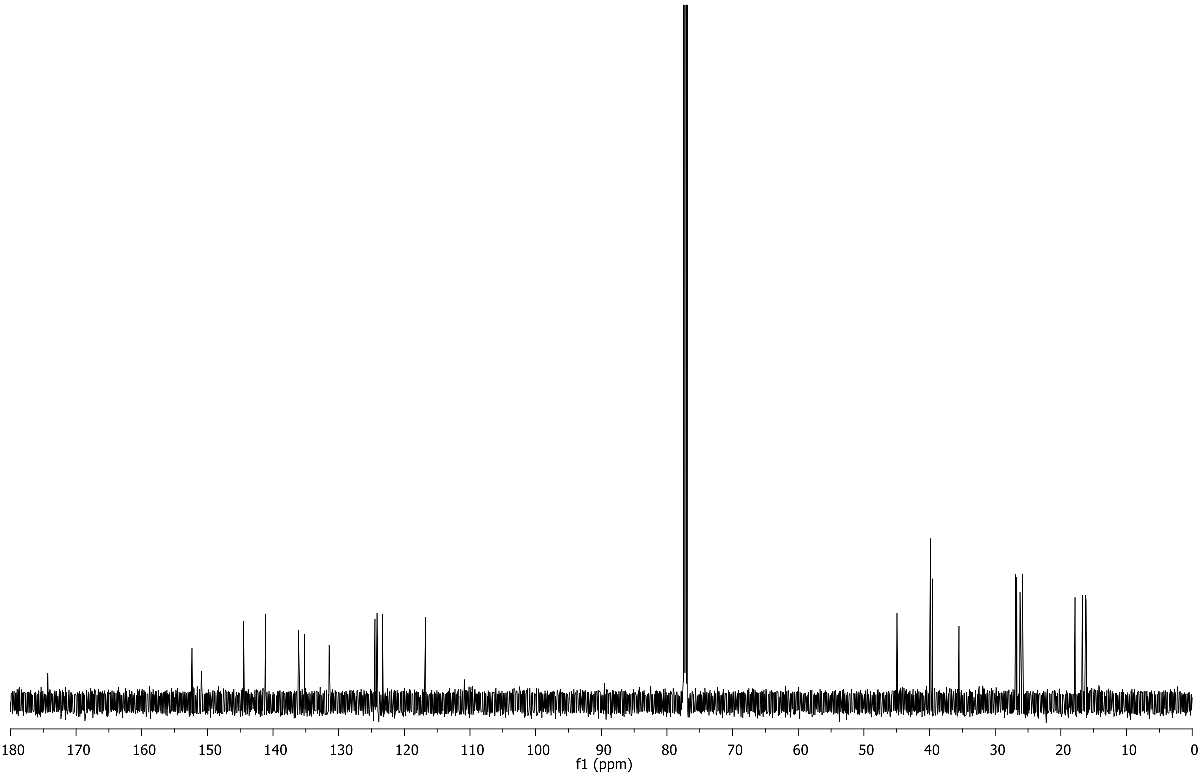

Figure S74. ^{13}C NMR spectrum (150 MHz, $CDCl_3$) of **41**.

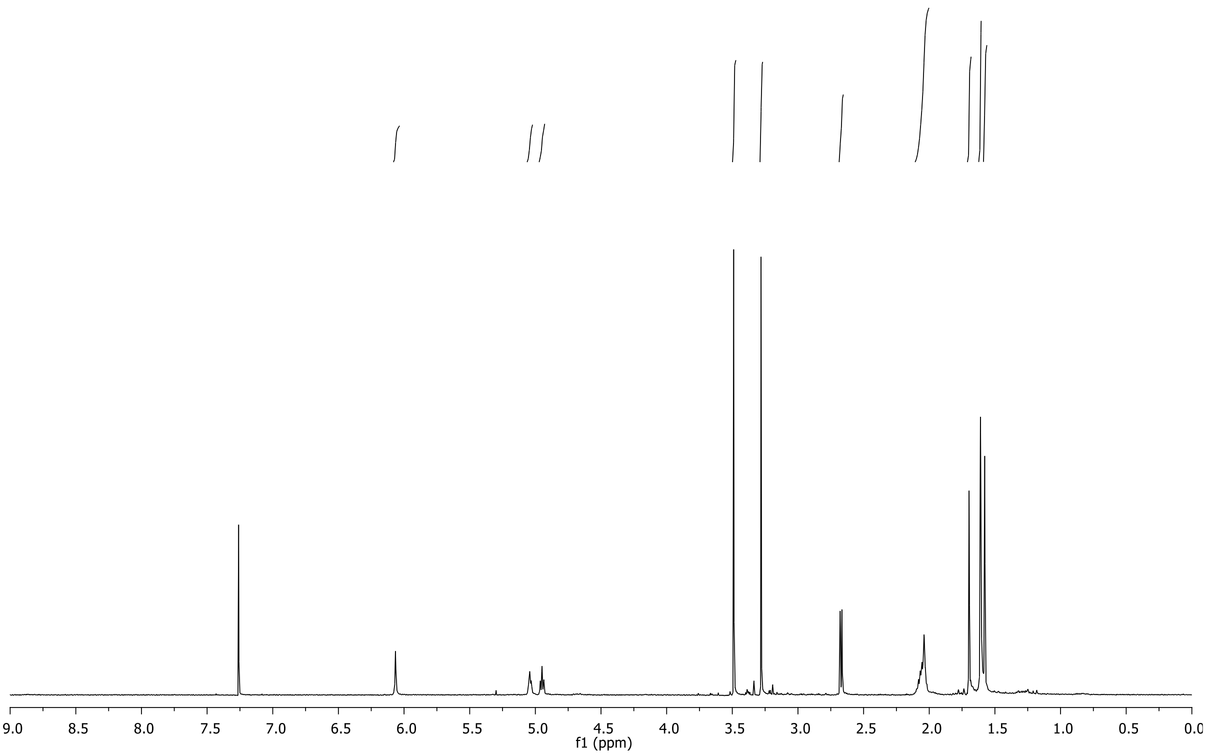

Figure S75. ^{1}H NMR spectrum (600 MHz, $CDCl_3$) of **42**.

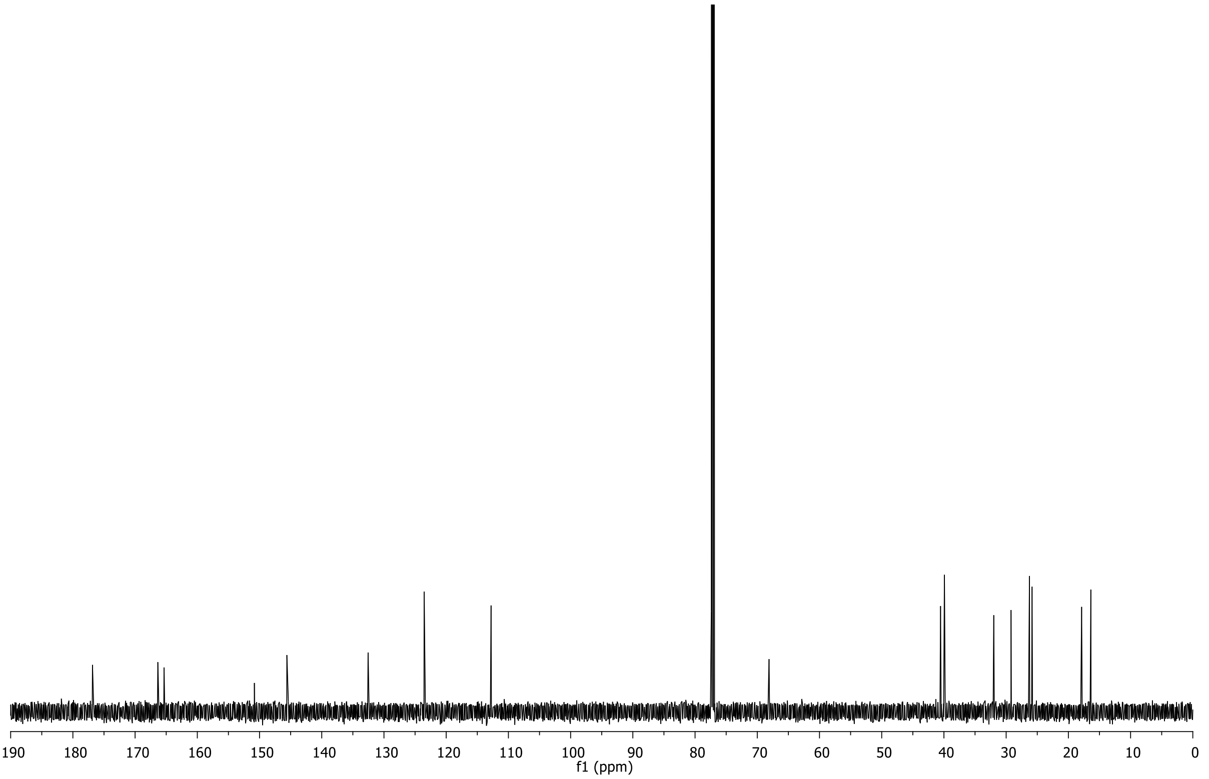

Figure S76. ^{13}C NMR spectrum (150 MHz, $CDCl_3$) of **42**.

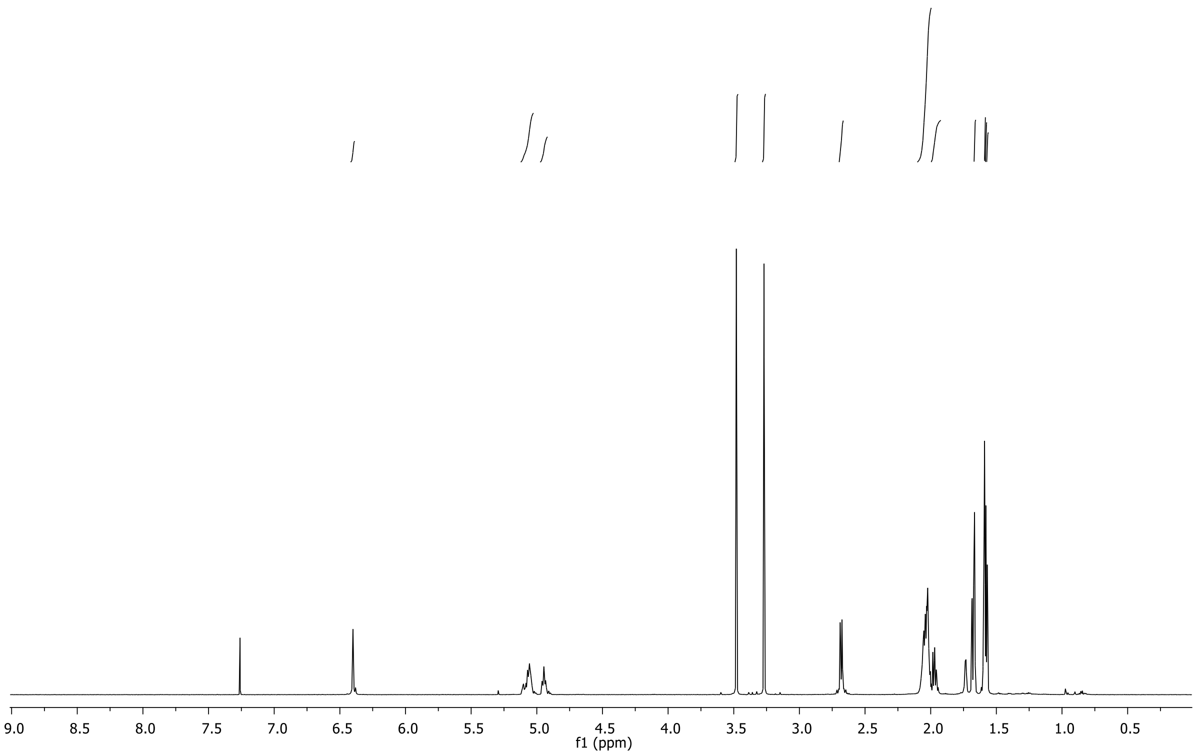

Figure S77. ^{1}H NMR spectrum (600 MHz, $CDCl_3$) of **43**.

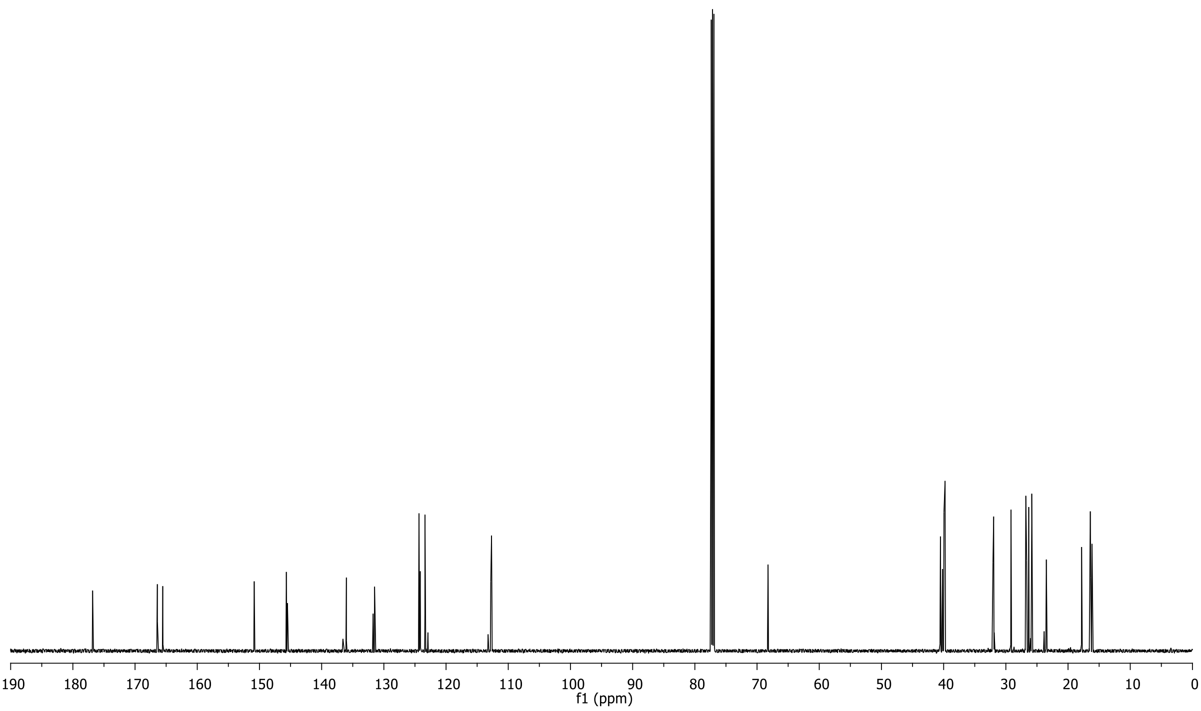

Figure S78. ^{13}C NMR spectrum (150 MHz, $CDCl_3$) of **43**.

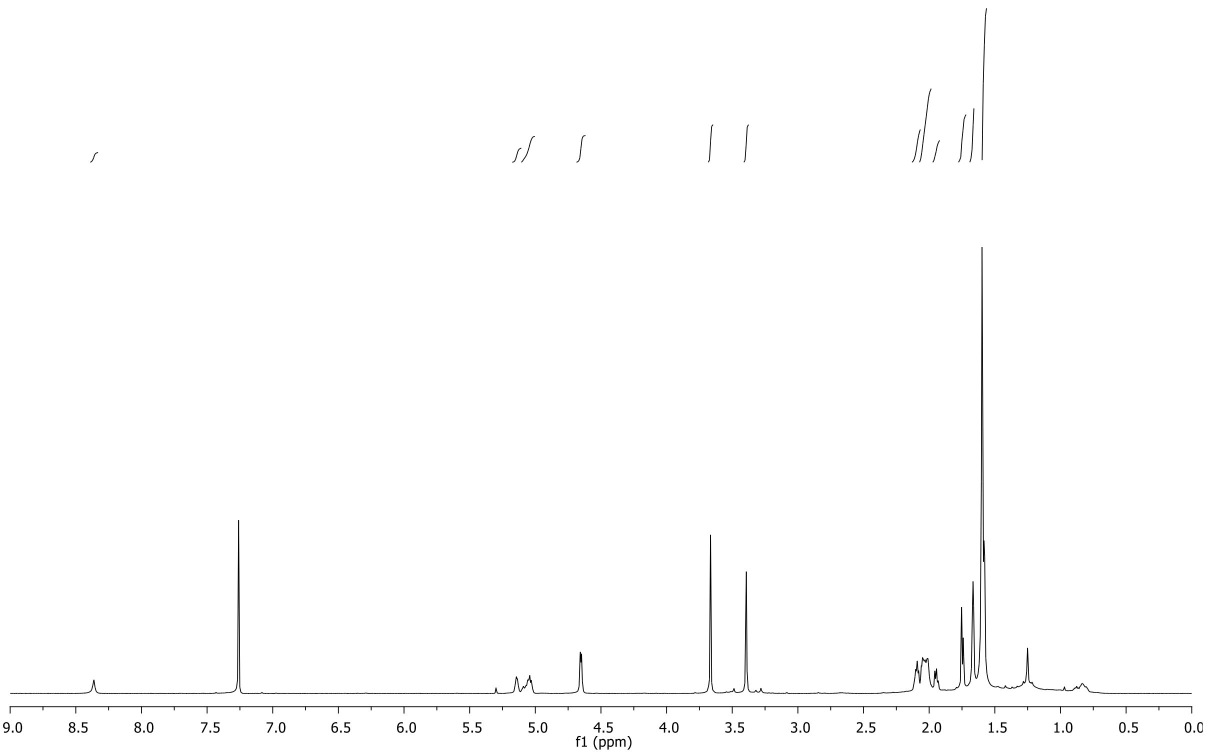

Figure S79. ^{1}H NMR spectrum (600 MHz, $CDCl_3$) of **44**.

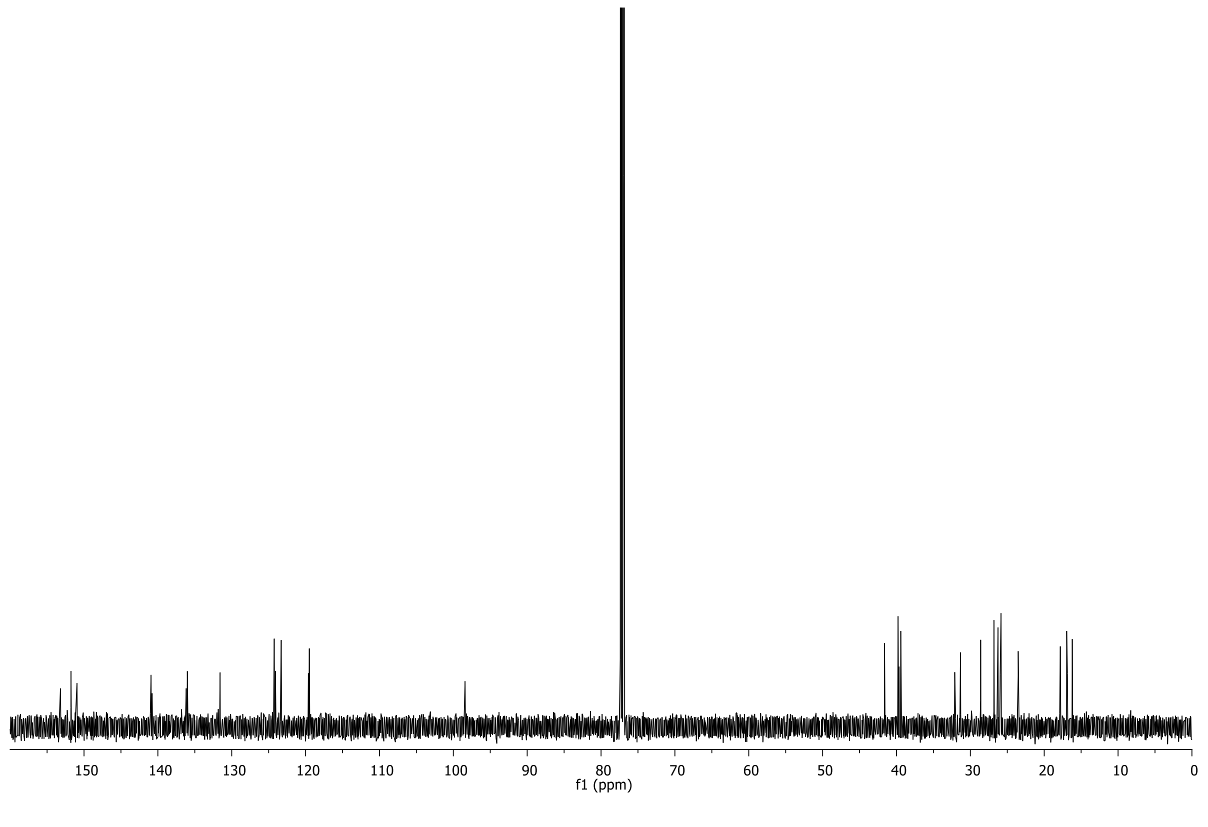

Figure S80. ^{13}C NMR spectrum (150 MHz, $CDCl_3$) of **44**.

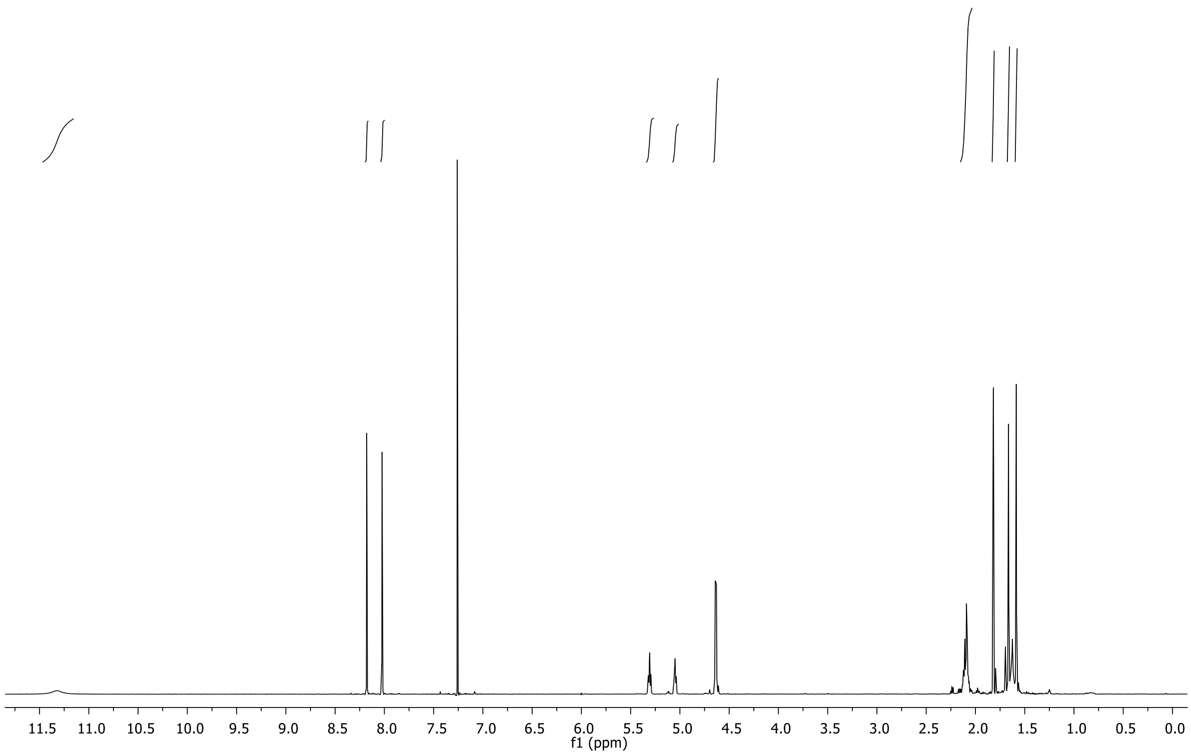

Figure S81. ^{1}H NMR spectrum (600 MHz, $CDCl_3$) of **46**.

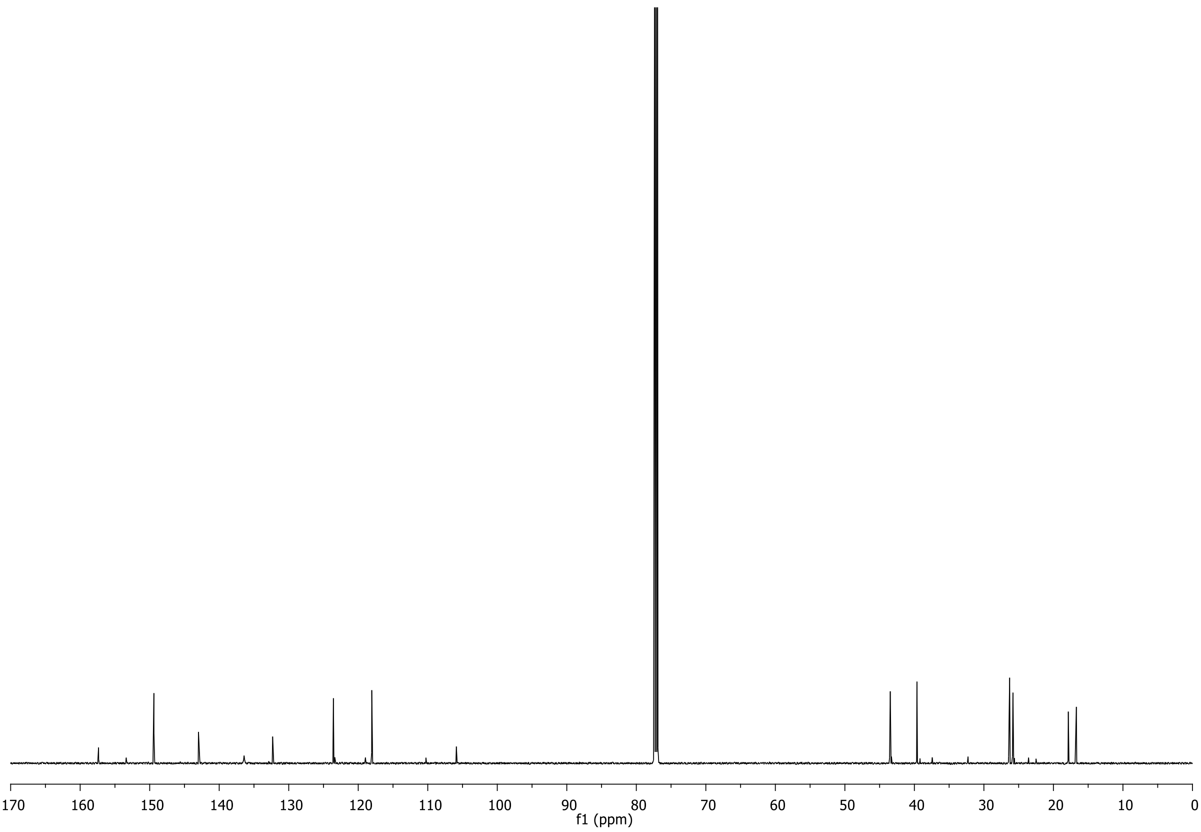

Figure S82. ^{13}C NMR spectrum (150 MHz, $CDCl_3$) of **46**.

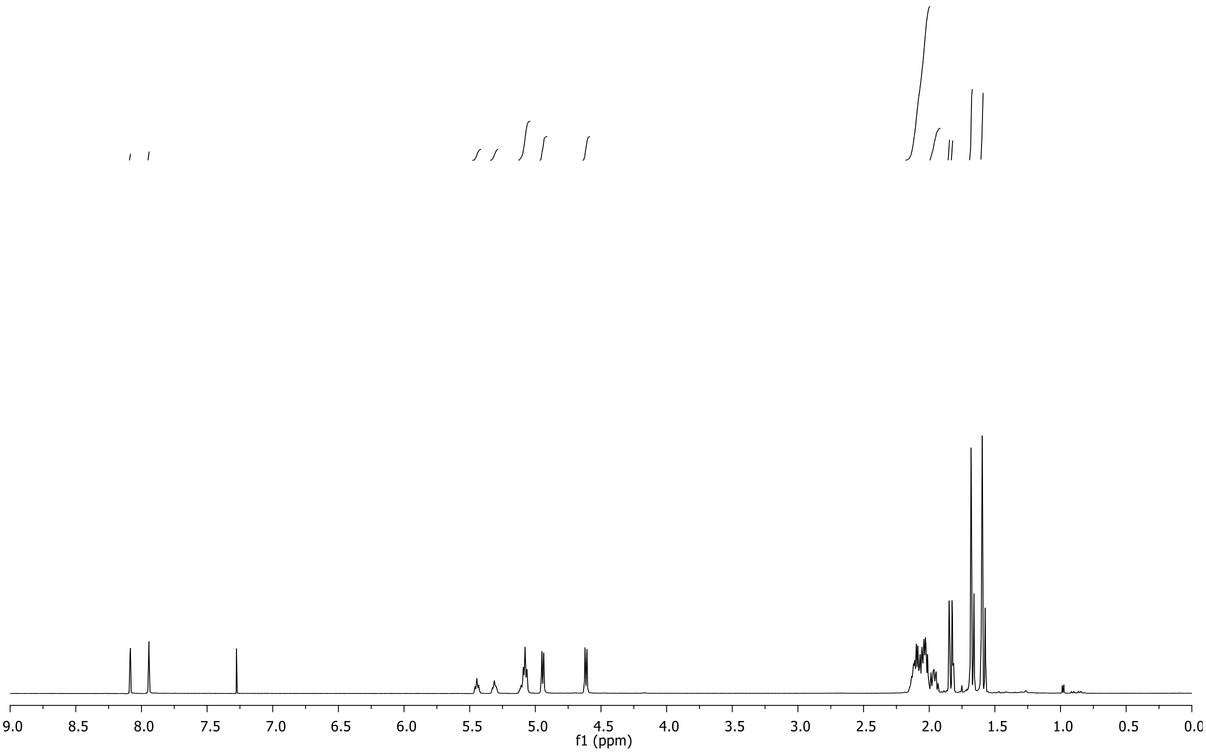

Figure S83. ^{1}H NMR spectrum (500 MHz, $CDCl_3$) of **47**.

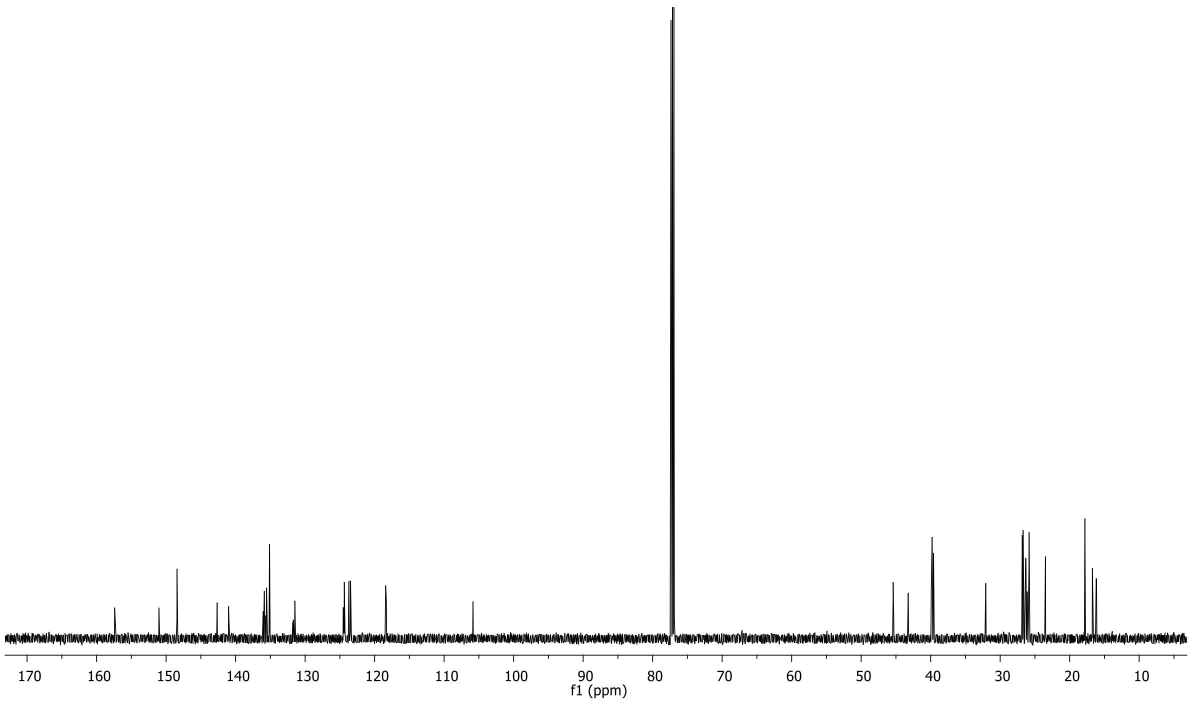

Figure S84. ^{13}C NMR spectrum (150 MHz, $CDCl_3$) of **47**.

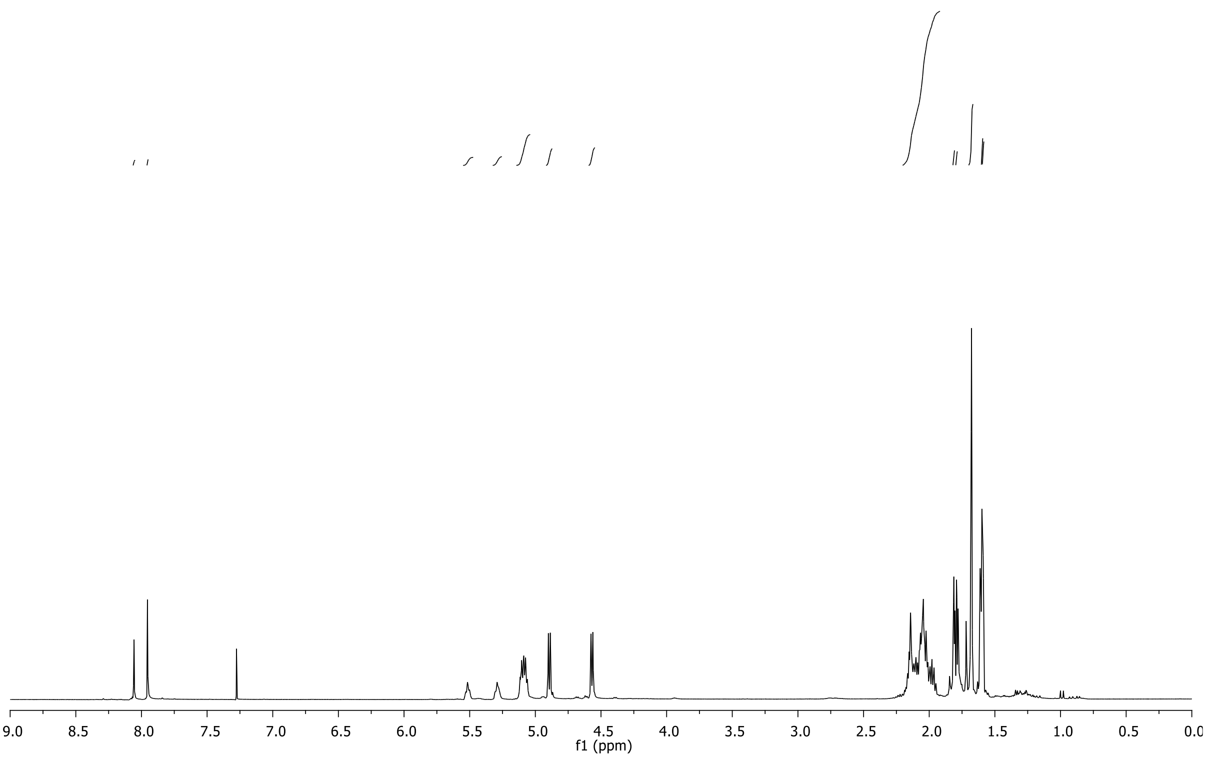

Figure S85. ^{1}H NMR spectrum (500 MHz, $CDCl_3$) of **48**.

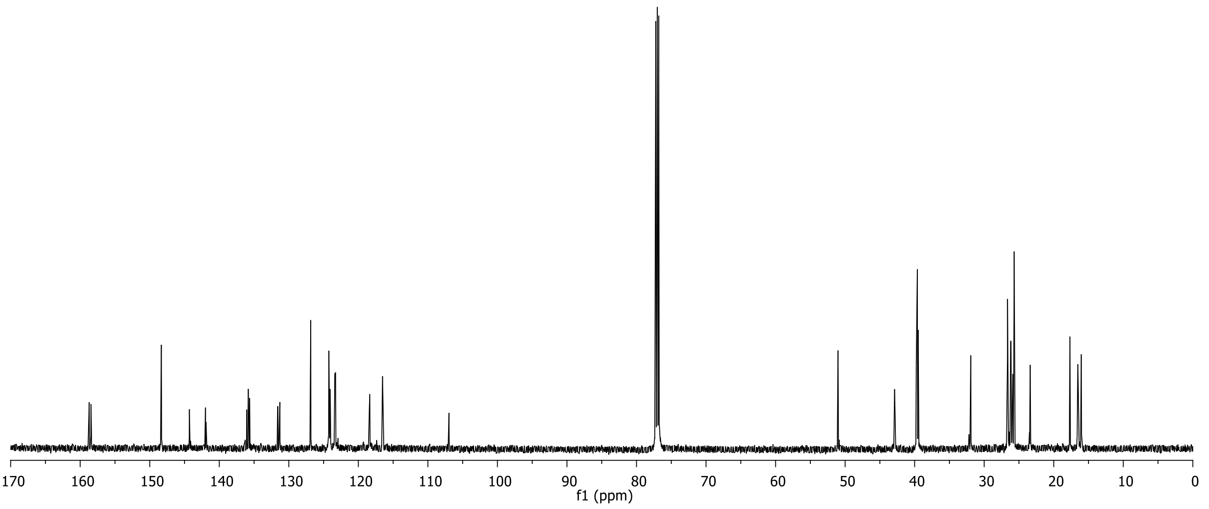

Figure S86. ^{13}C NMR spectrum (150 MHz, $CDCl_3$) of **48**.

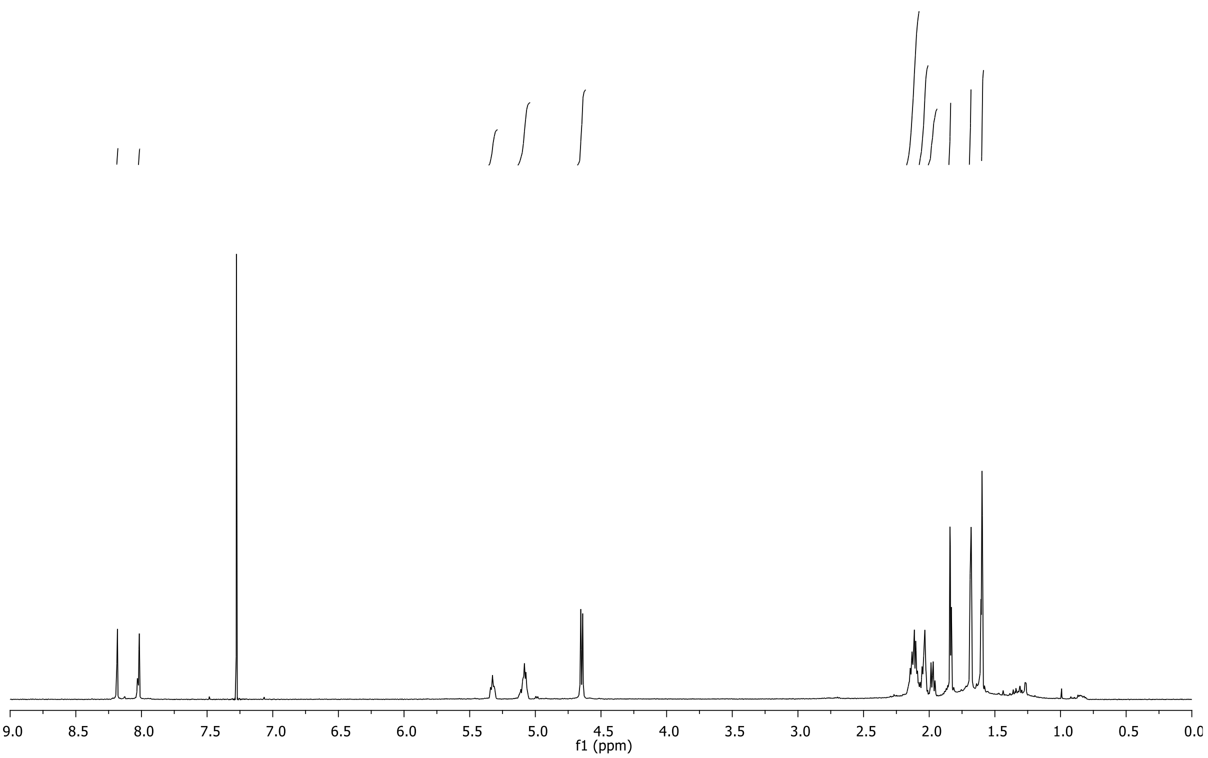

Figure S87. ^{1}H NMR spectrum (600 MHz, $CDCl_3$) of **49**.

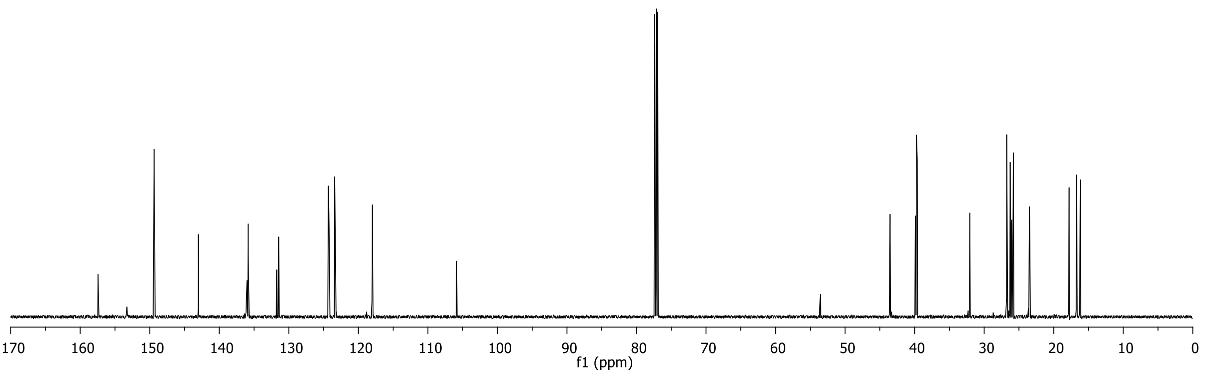

Figure S88. ^{13}C NMR spectrum (150 MHz, $CDCl_3$) of **49**.

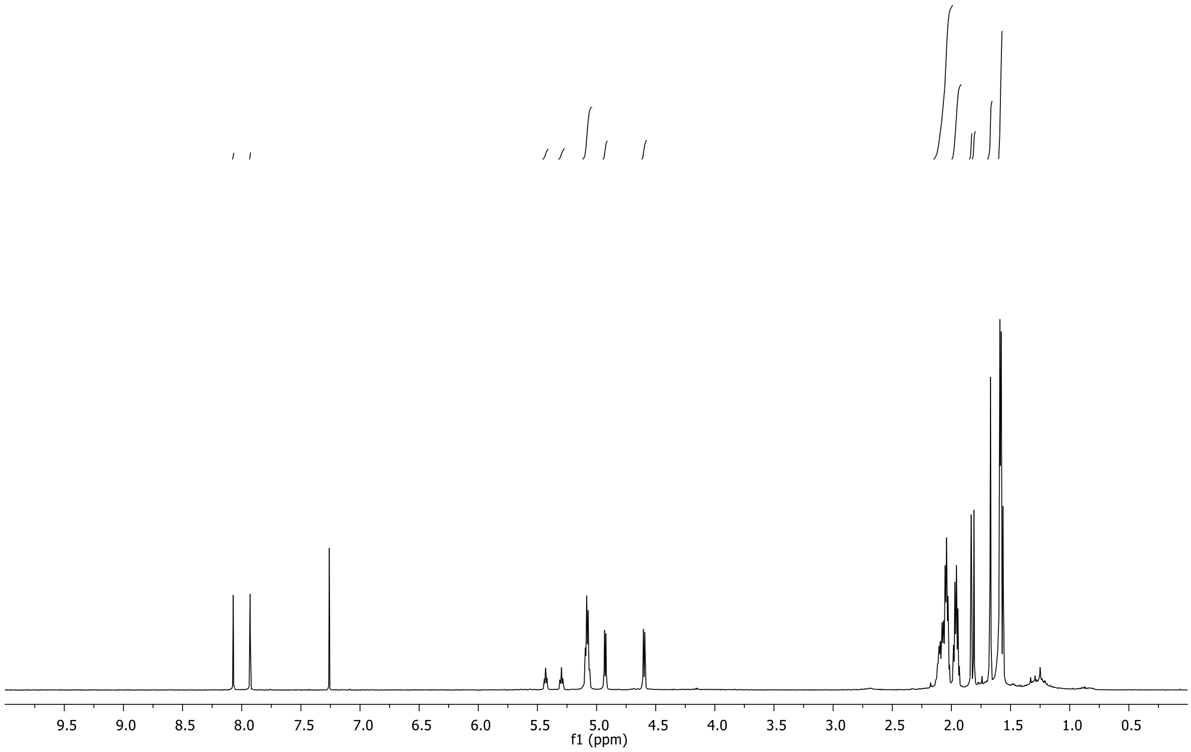

Figure S89. ^{1}H NMR spectrum (150 MHz, $CDCl_3$) of **50**.

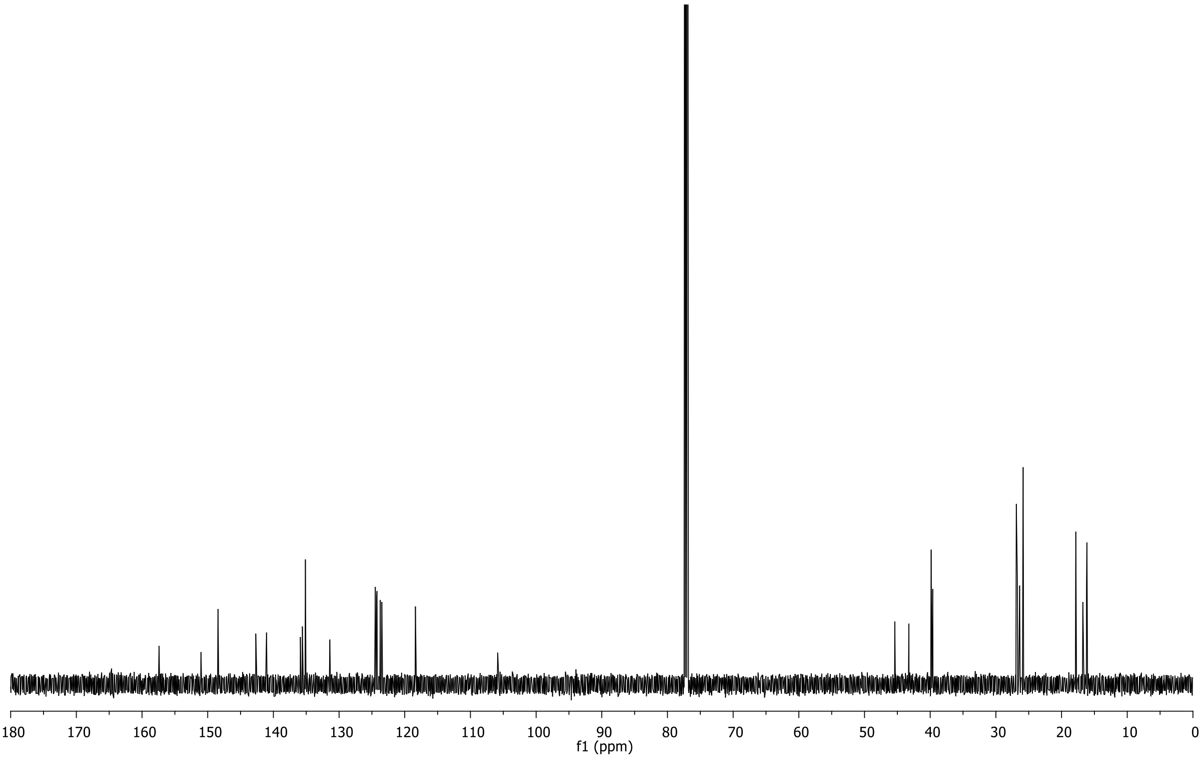

Figure S90. ^{13}C NMR spectrum (150 MHz, $CDCl_3$) of **50**.

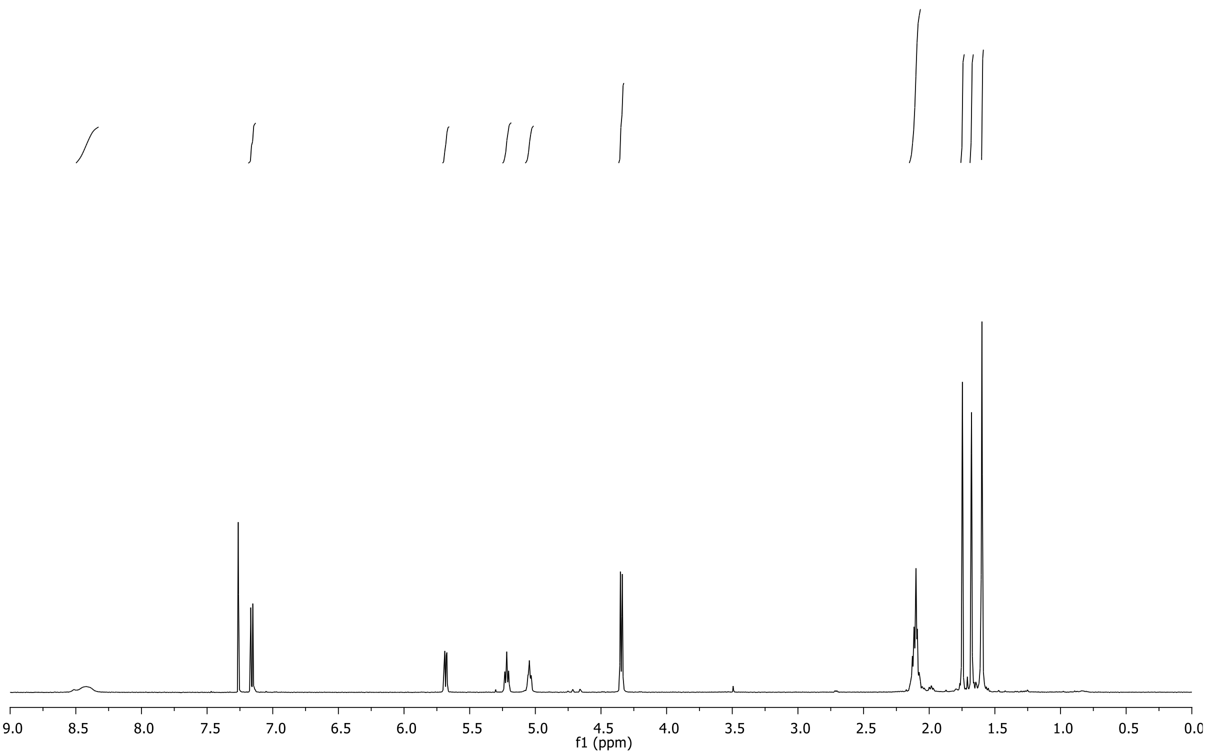

Figure S91. ^{1}H NMR spectrum (500 MHz, $CDCl_3$) of **54**.

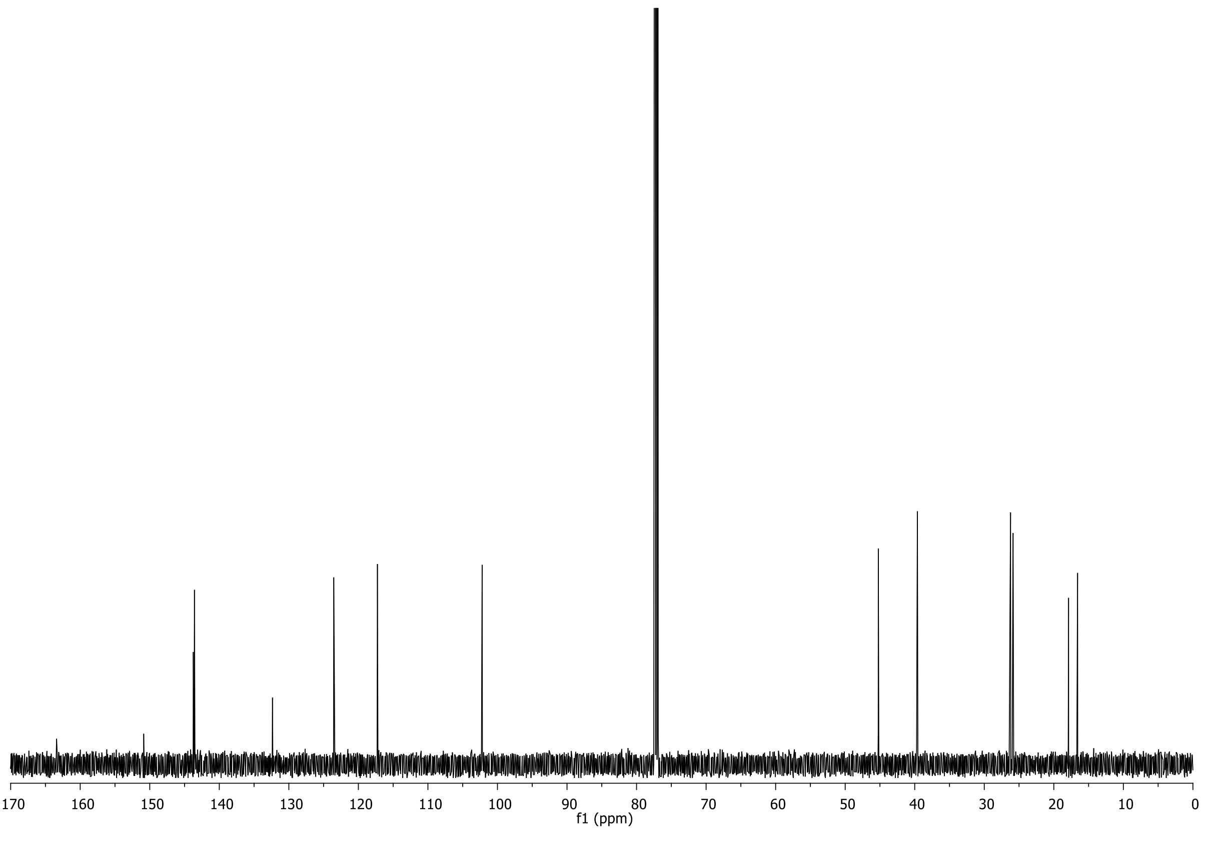

Figure S92. ^{13}C NMR spectrum (150 MHz, $CDCl_3$) of **54**.

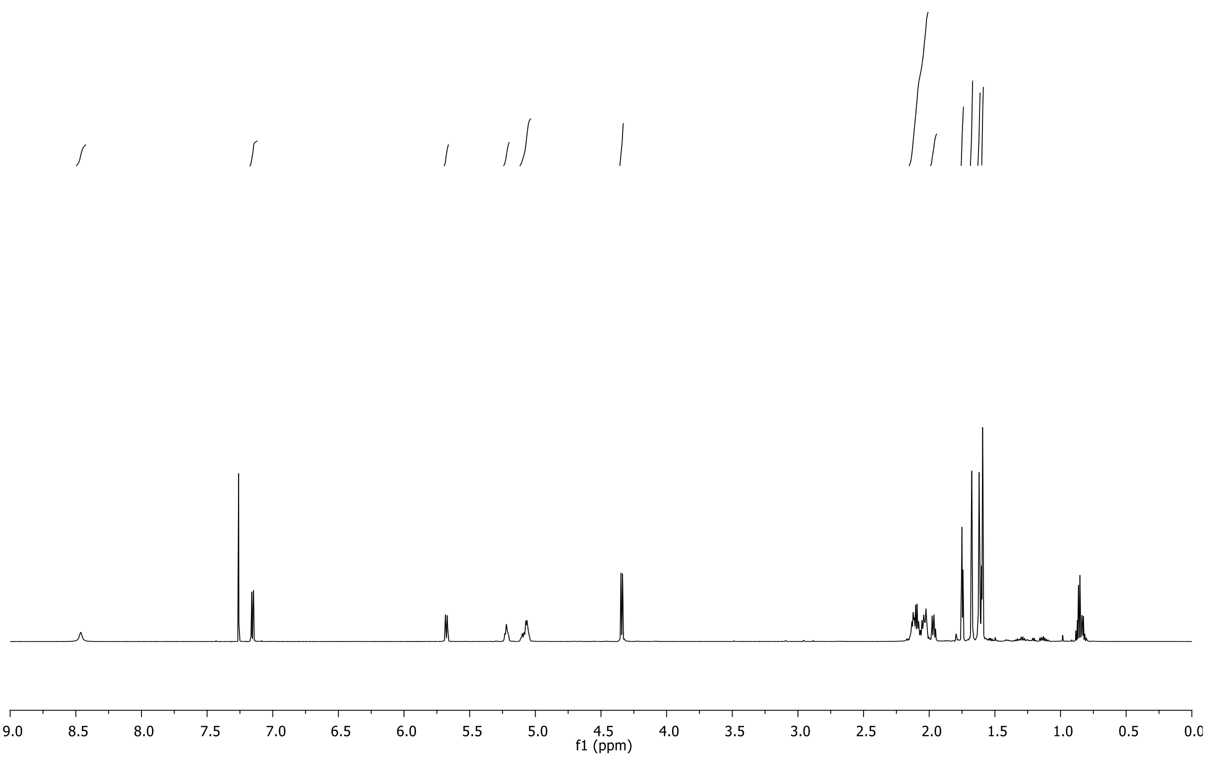

Figure S93. ^{1}H NMR spectrum (600 MHz, $CDCl_3$) of **55**.

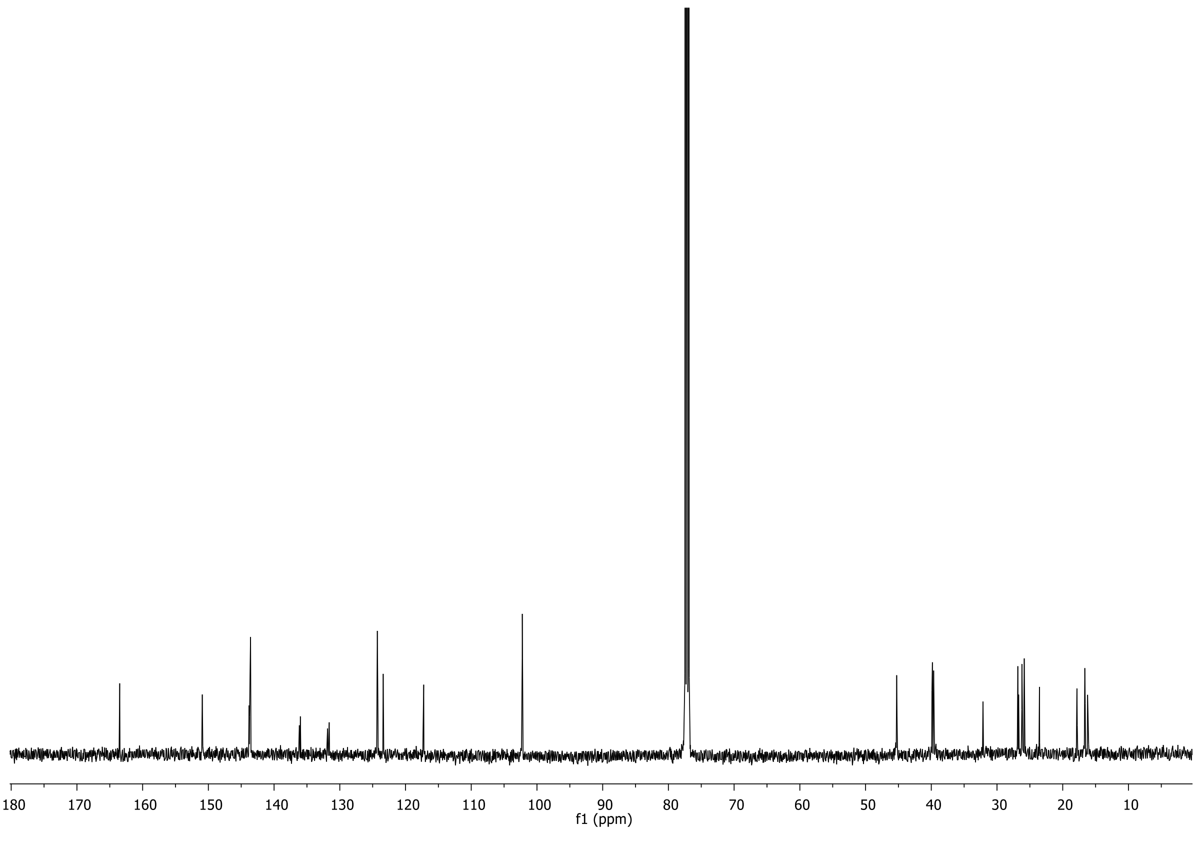

Figure S94. ^{13}C NMR spectrum (150 MHz, $CDCl_3$) of **55**.

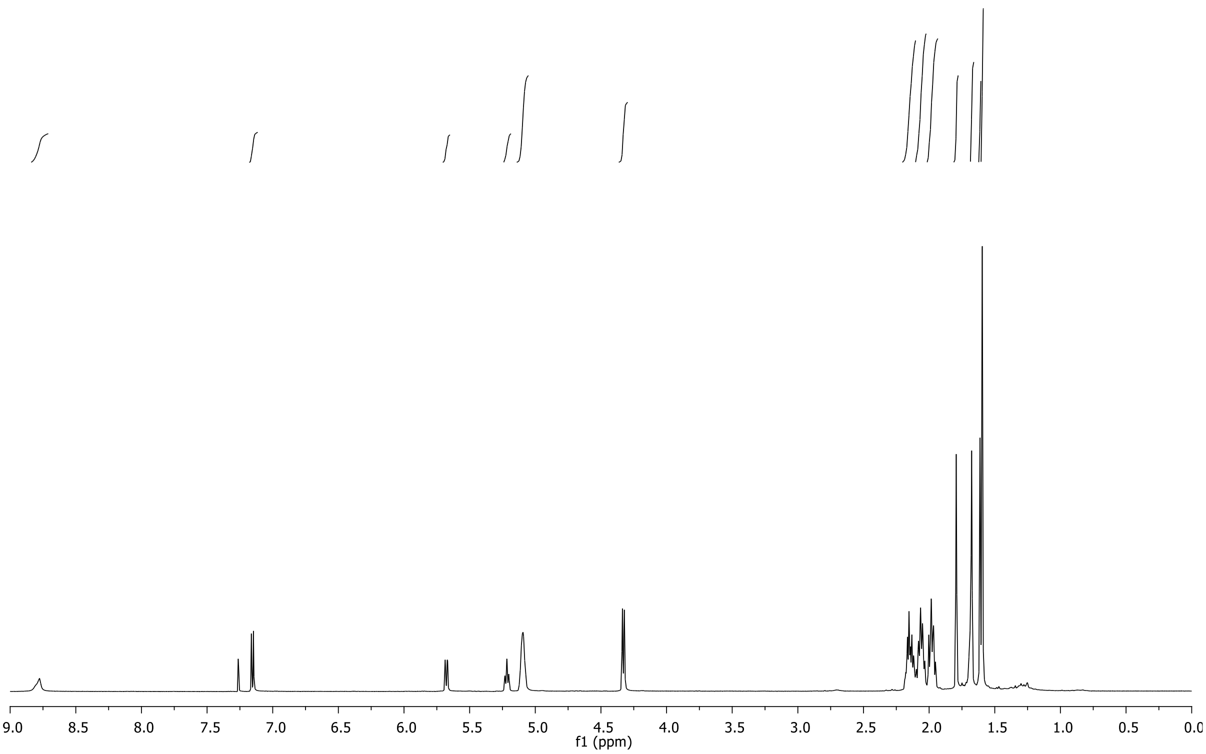

Figure S95. ^{1}H NMR spectrum (500 MHz, $CDCl_3$) of **56**.

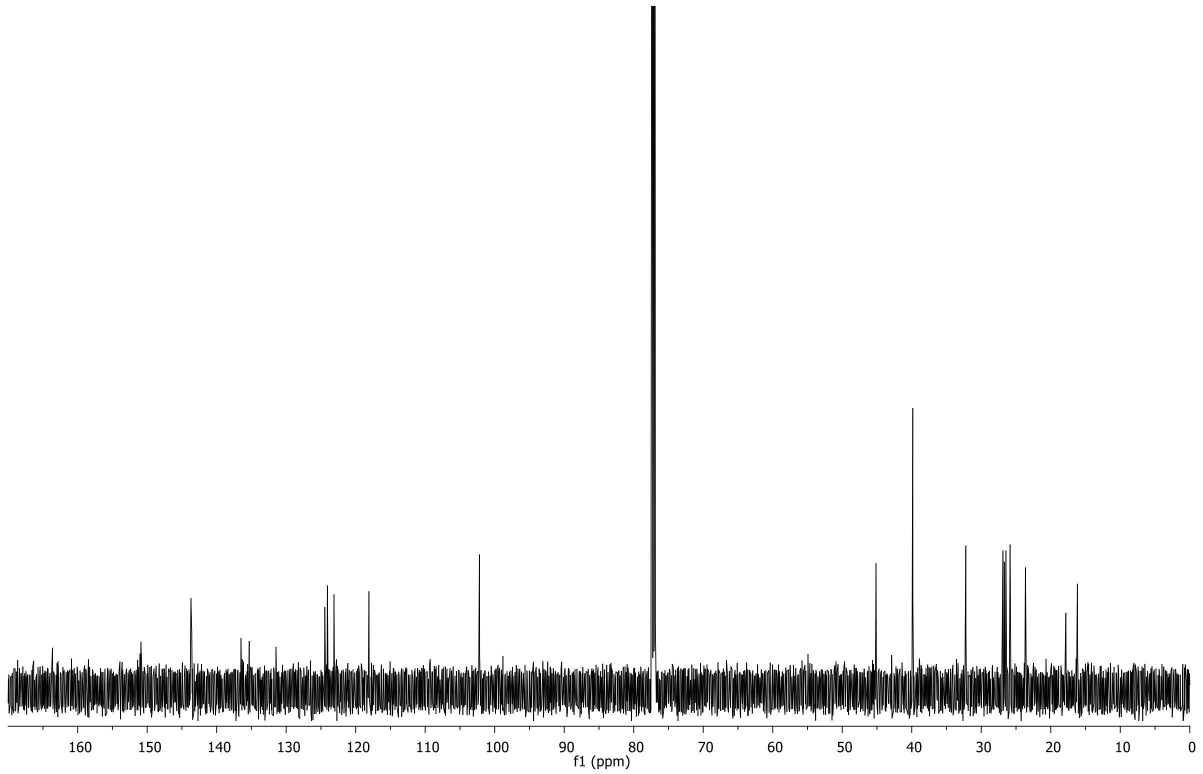

Figure S96. ^{13}C NMR spectrum (150 MHz, $CDCl_3$) of **56**.

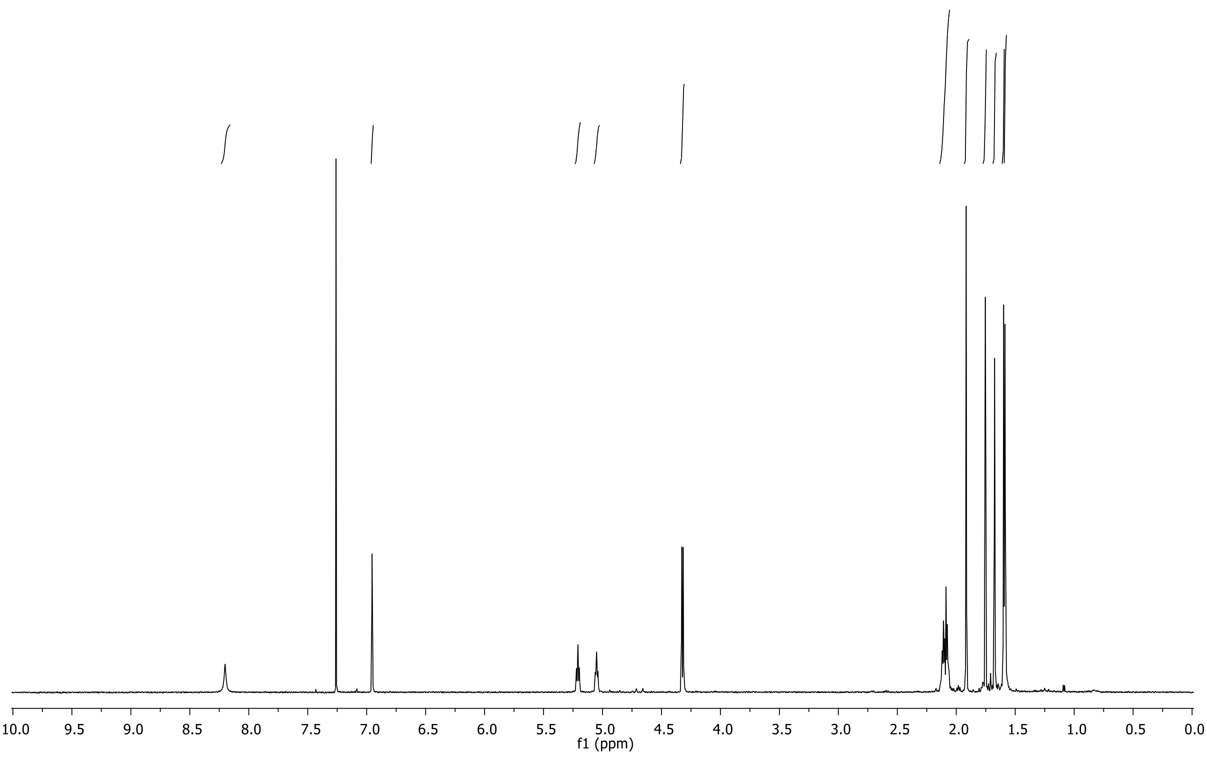

Figure S97. ^{1}H NMR spectrum (600 MHz, $CDCl_3$) of **57**.

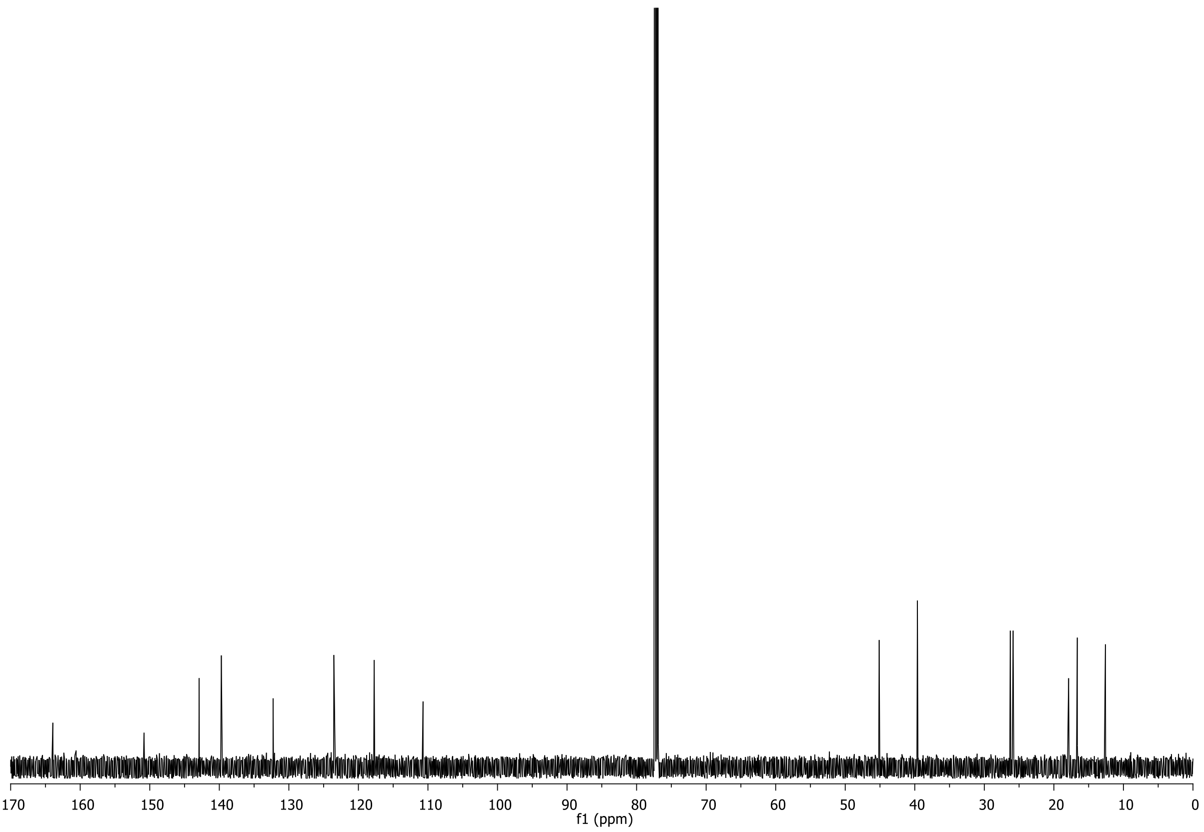

Figure S98. ^{13}C NMR spectrum (150 MHz, $CDCl_3$) of **57**.

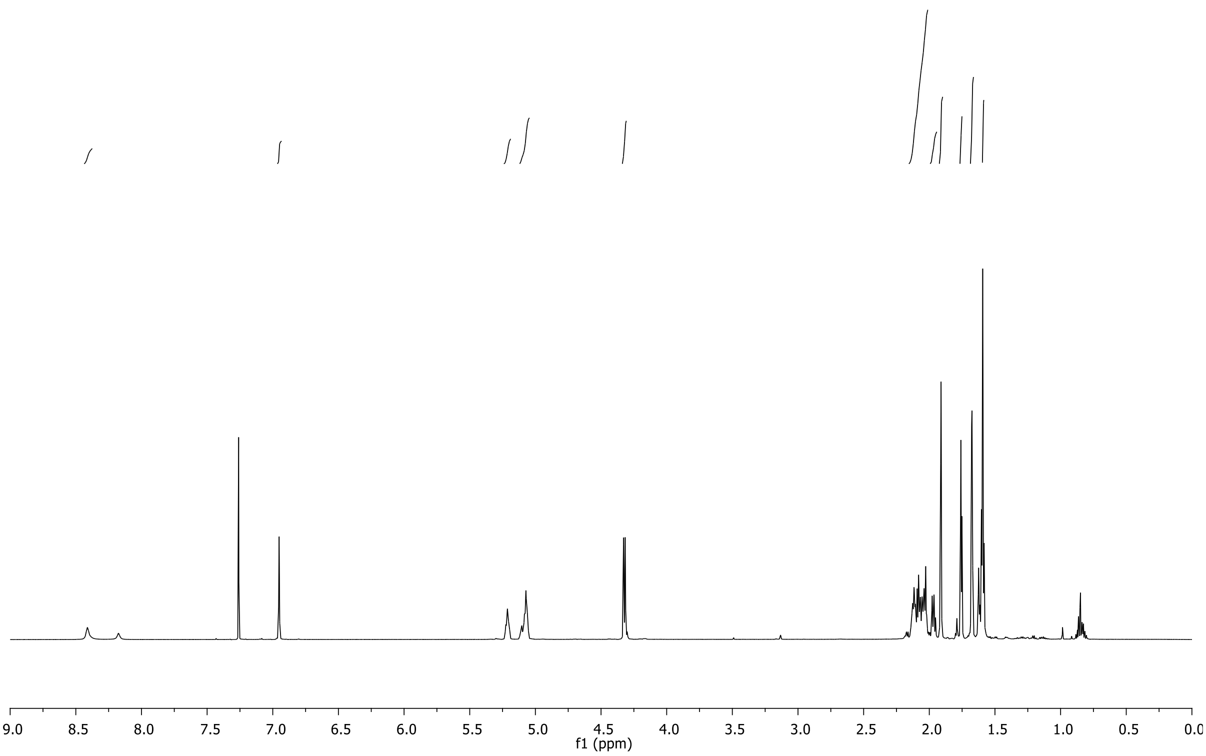

Figure S99. ^{1}H NMR spectrum (600 MHz, $CDCl_3$) of **58**.

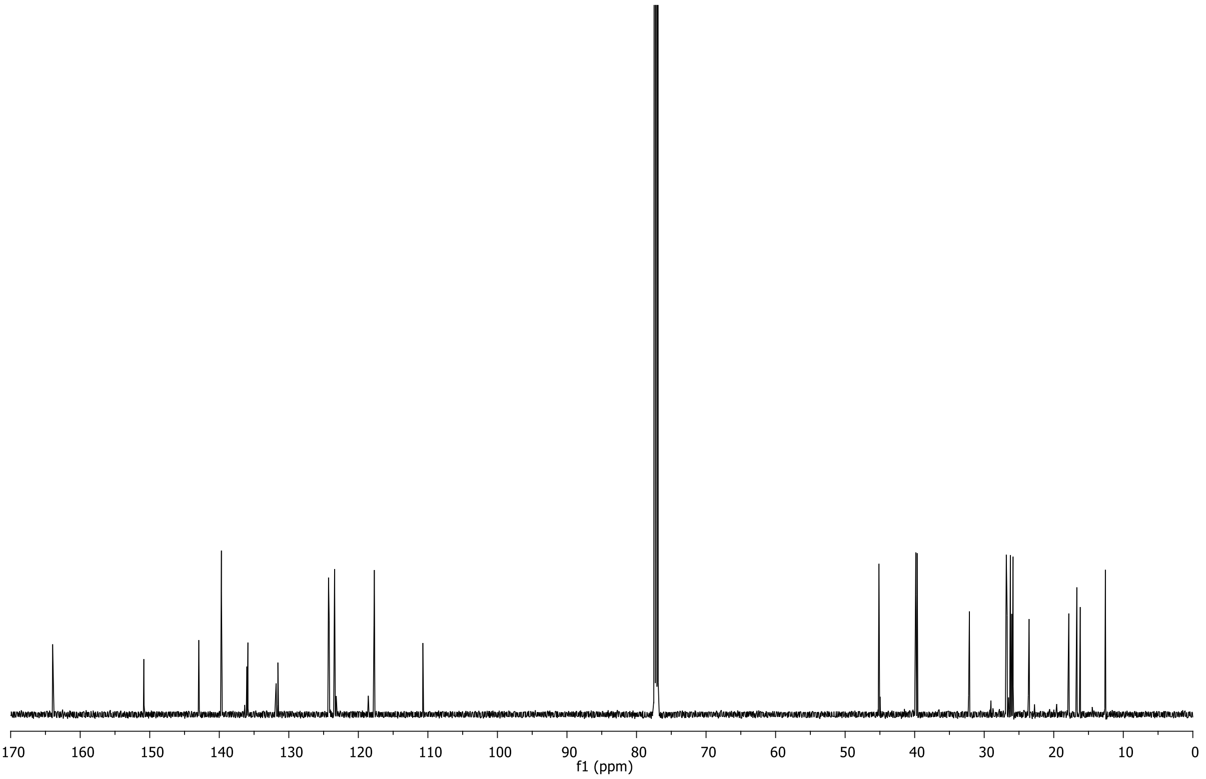

Figure S100. ^{13}C NMR spectrum (150 MHz, $CDCl_3$) of **58**.

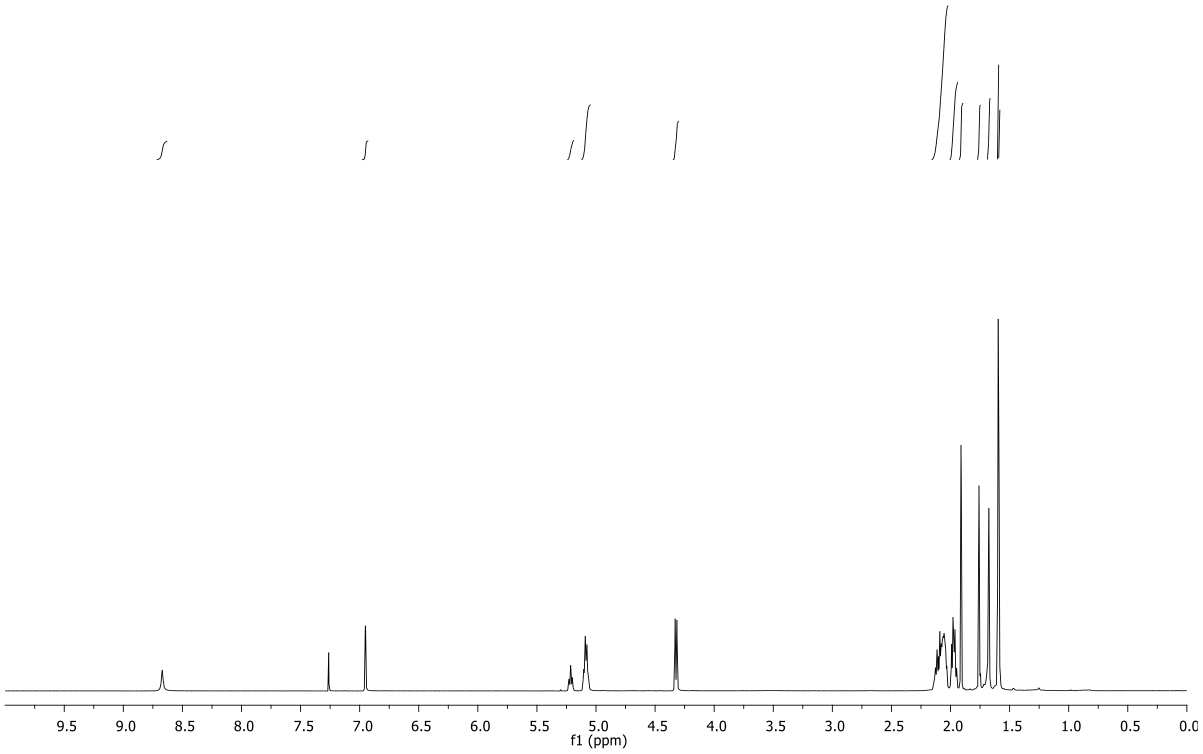

Figure S101. ^{1}H NMR spectrum (500 MHz, $CDCl_3$) of **59**.

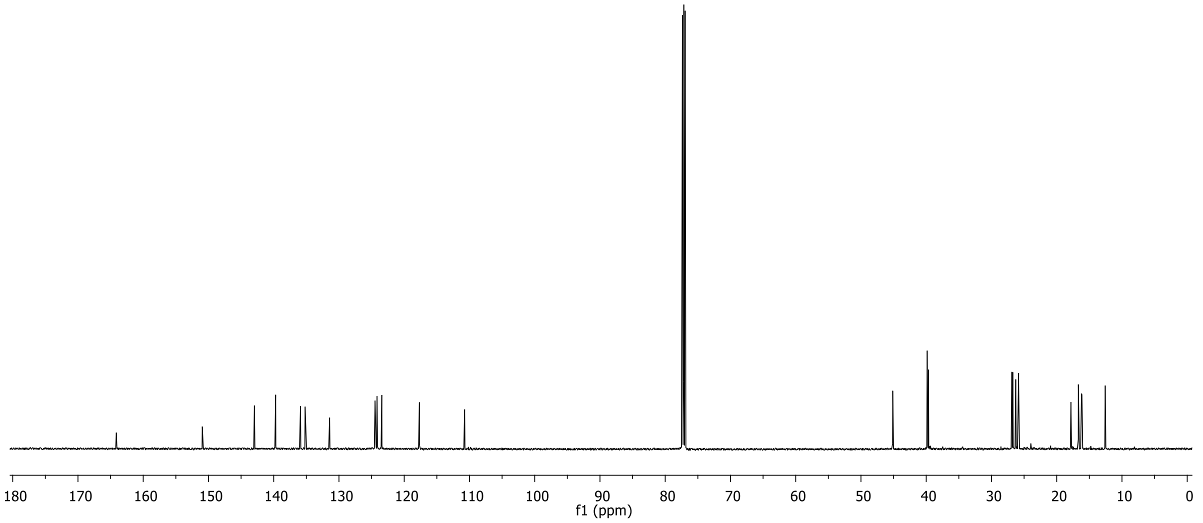

Figure S102. ^{13}C NMR spectrum (150 MHz, $CDCl_3$) of **59**.

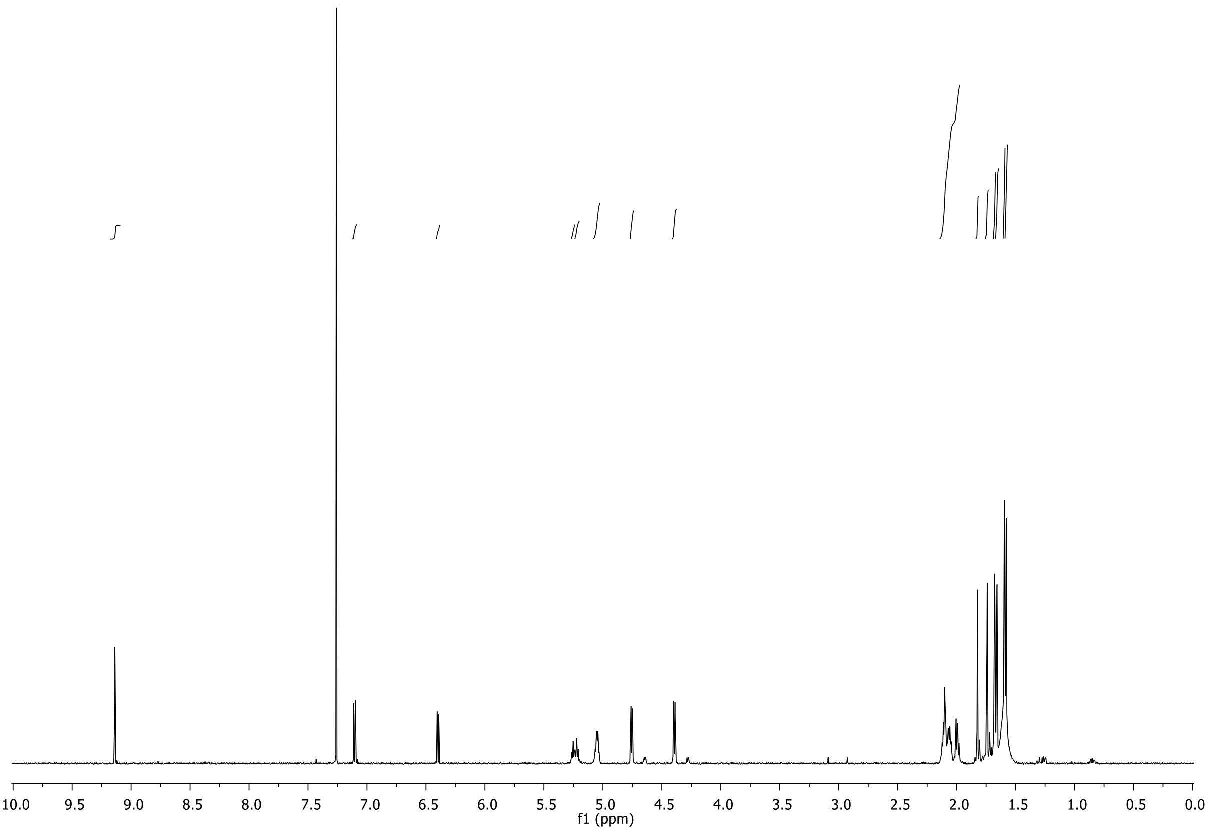

Figure S103. ^{1}H NMR spectrum (500 MHz, $CDCl_3$) of **60**.

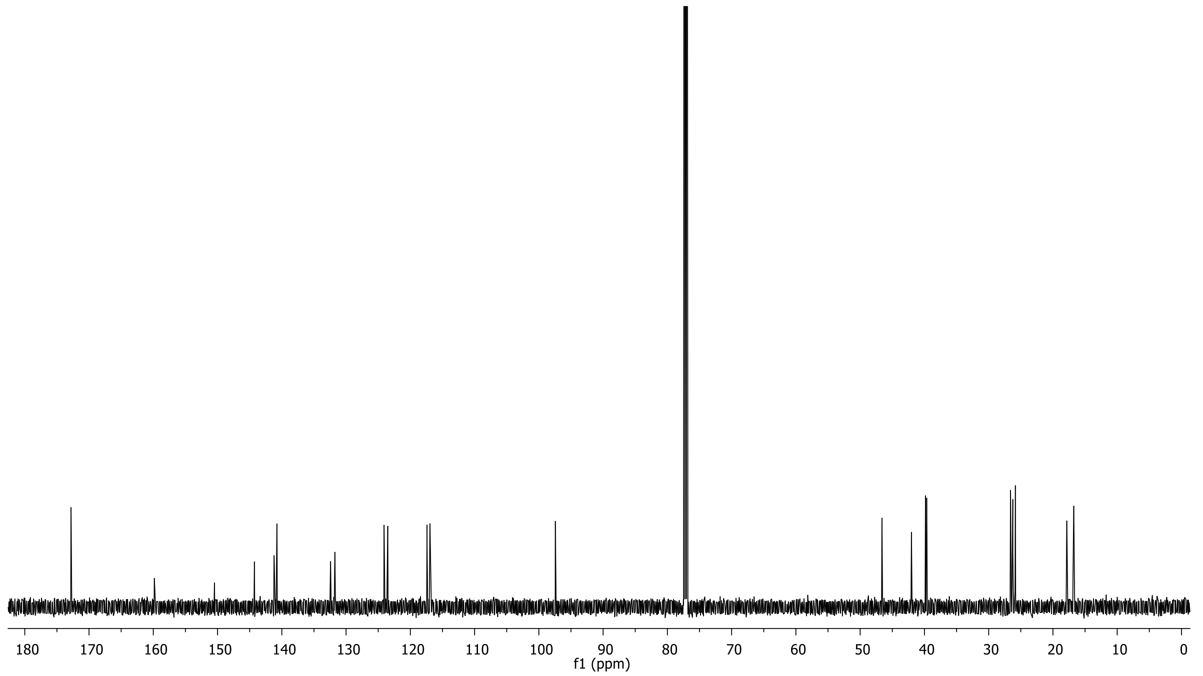

Figure S104. ^{13}C NMR spectrum (150 MHz, $CDCl_3$) of **60**.

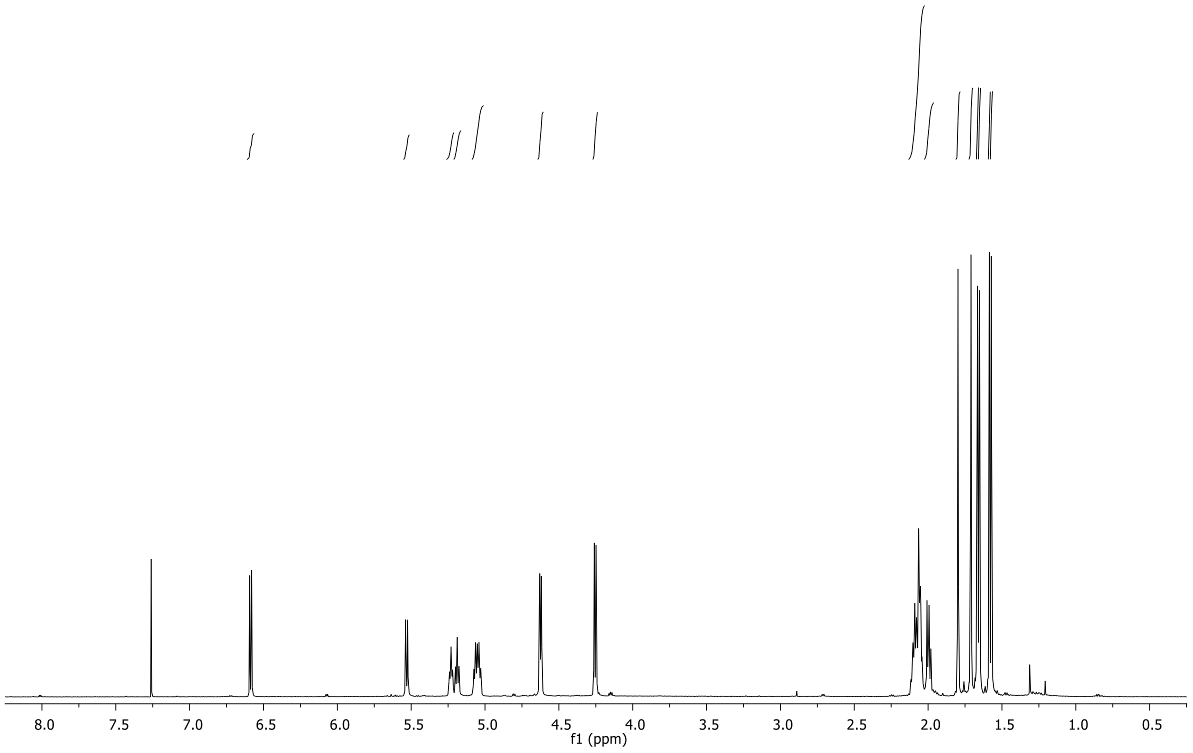

Figure S105. ^{1}H NMR spectrum (600 MHz, $CDCl_3$) of **61**.

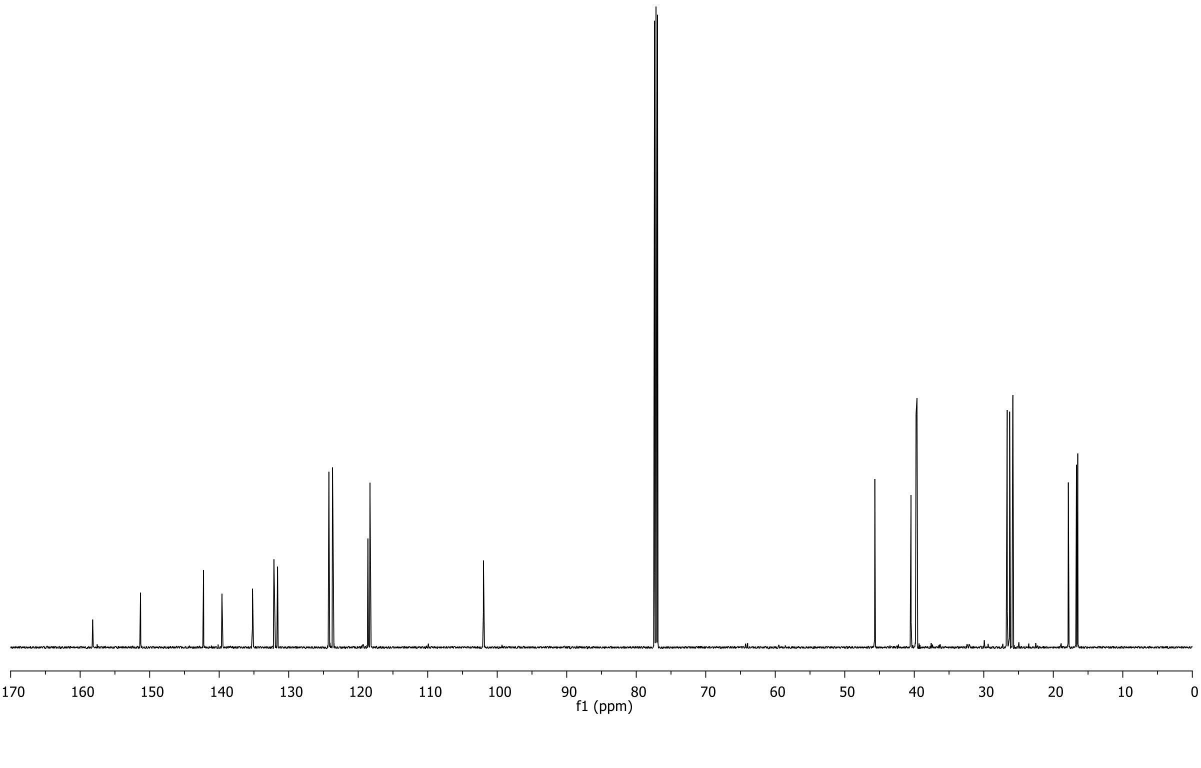

Figure S106. ^{13}C NMR spectrum (150 MHz, $CDCl_3$) of **61**.

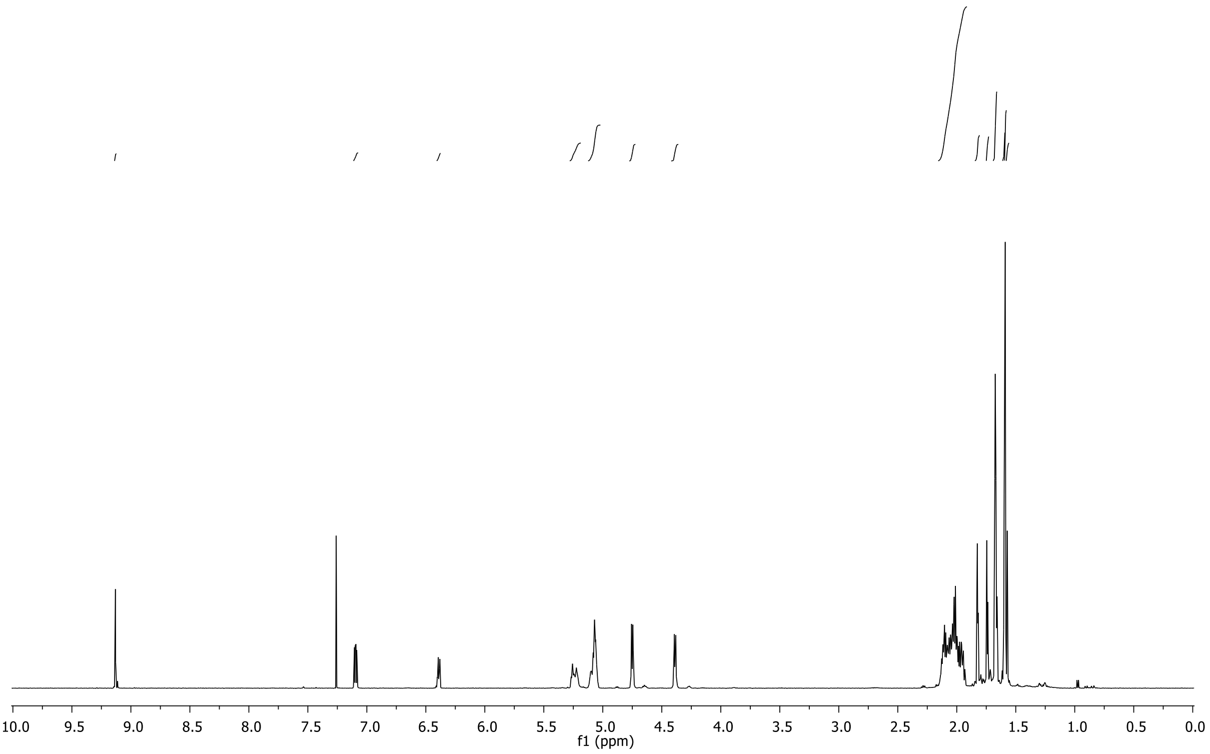

Figure S107. ^{1}H NMR spectrum (600 MHz, $CDCl_3$) of **62**.

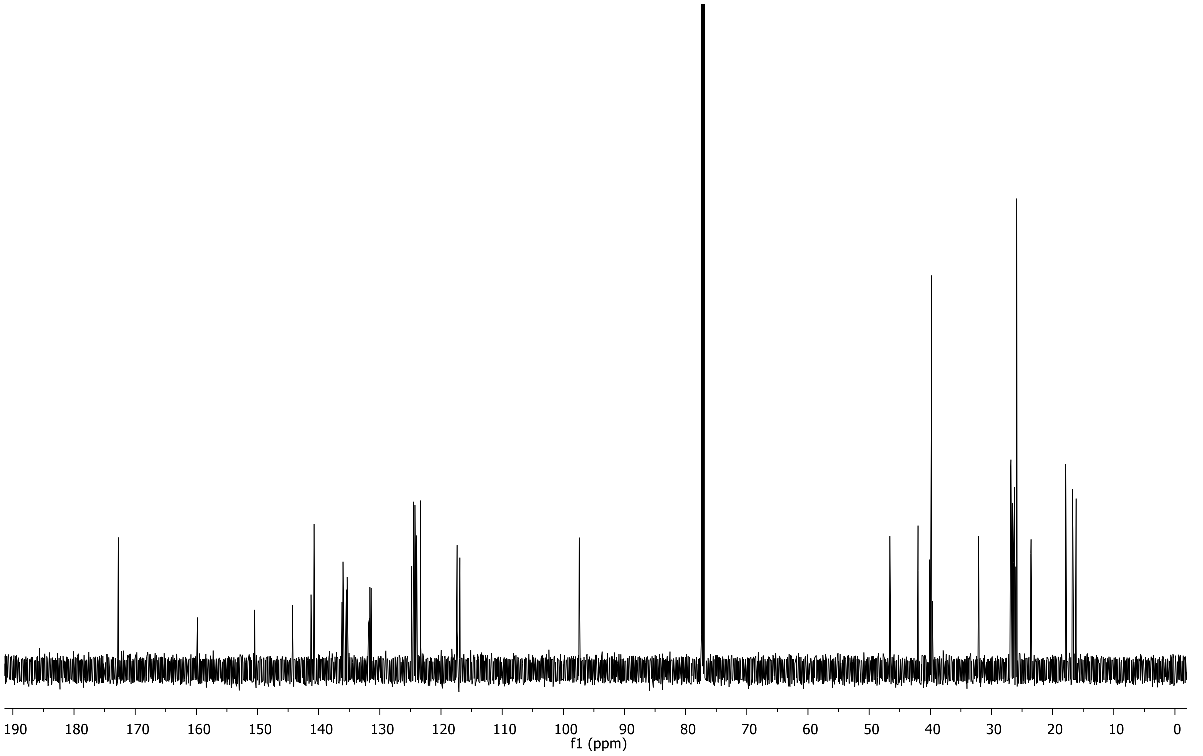

Figure S108. ^{13}C NMR spectrum (150 MHz, $CDCl_3$) of **62**.

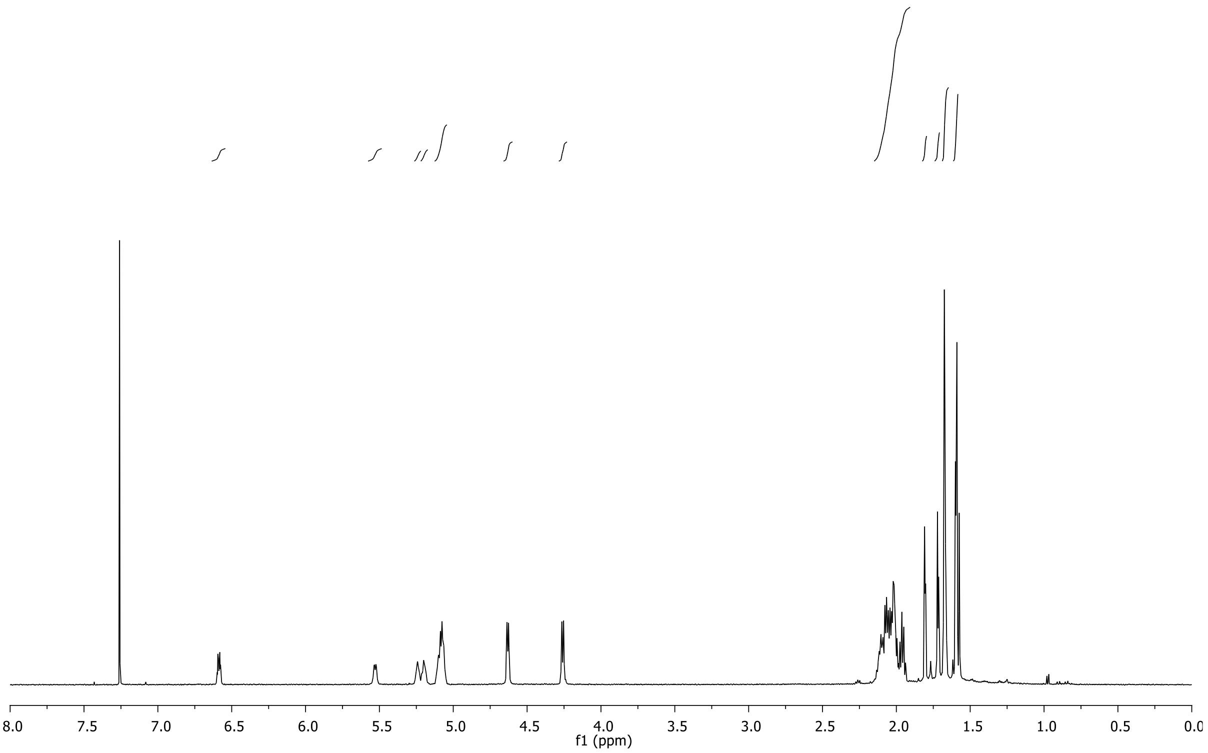

Figure S109. ^{1}H NMR spectrum (600 MHz, $CDCl_3$) of **63**.

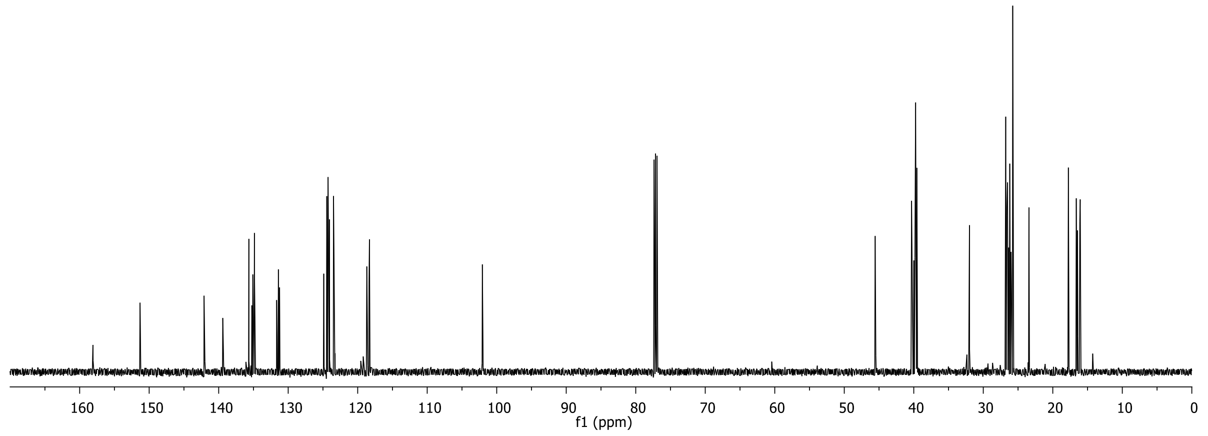

Figure S110. ^{13}C NMR spectrum (150 MHz, $CDCl_3$) of **63**.

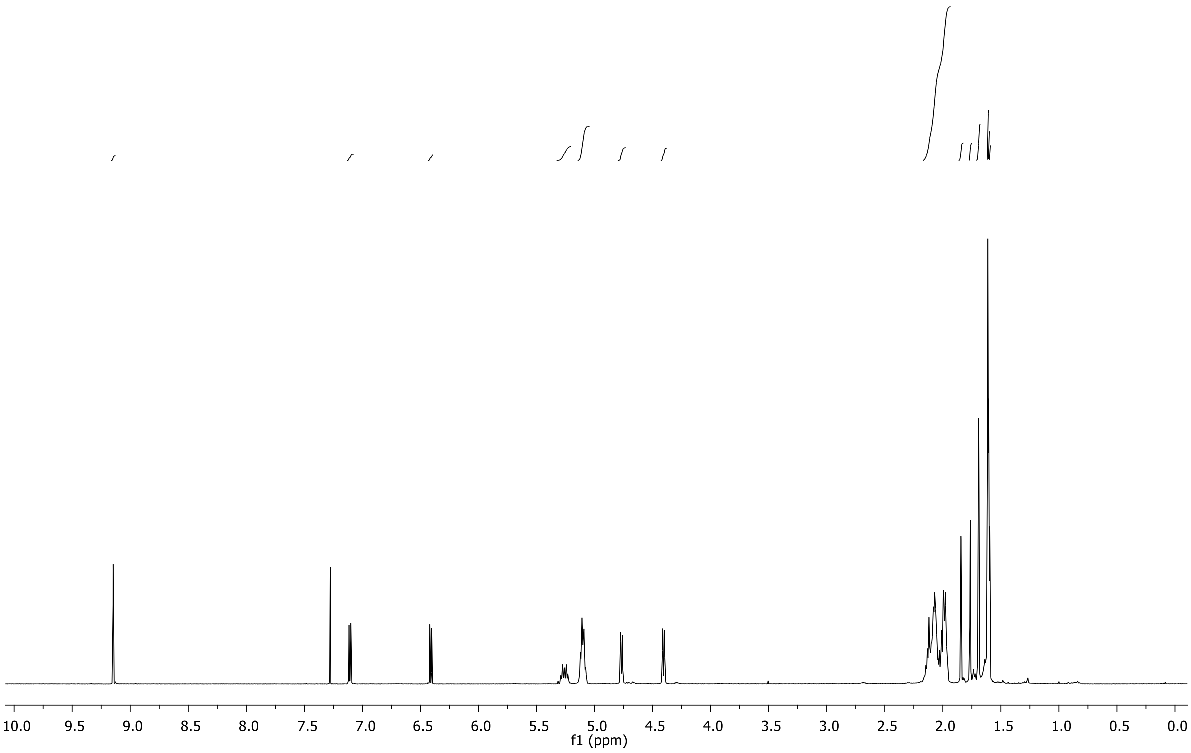

Figure S111. ^1H NMR spectrum (600 MHz, $CDCl_3$) of **64**.

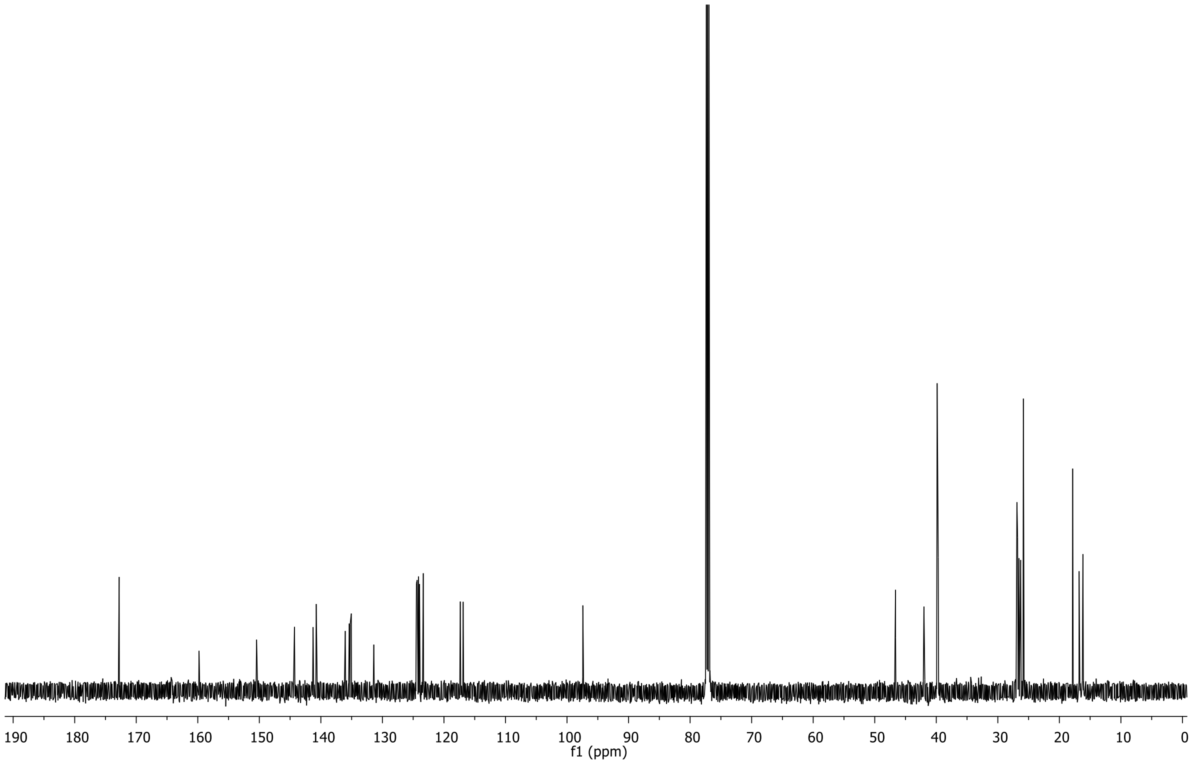

Figure S112. ^{13}C NMR spectrum (150 MHz, $CDCl_3$) of **64**.

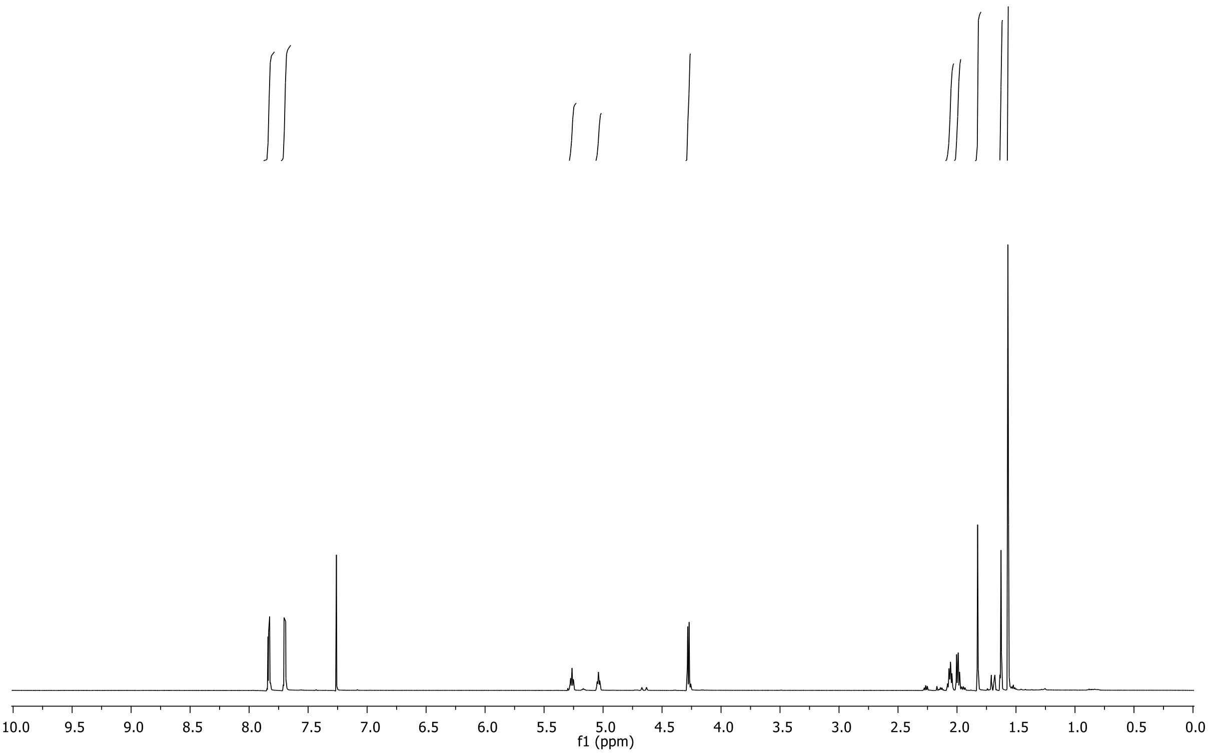

Figure S113. ^{1}H NMR spectrum (600 MHz, $CDCl_3$) of **67**.

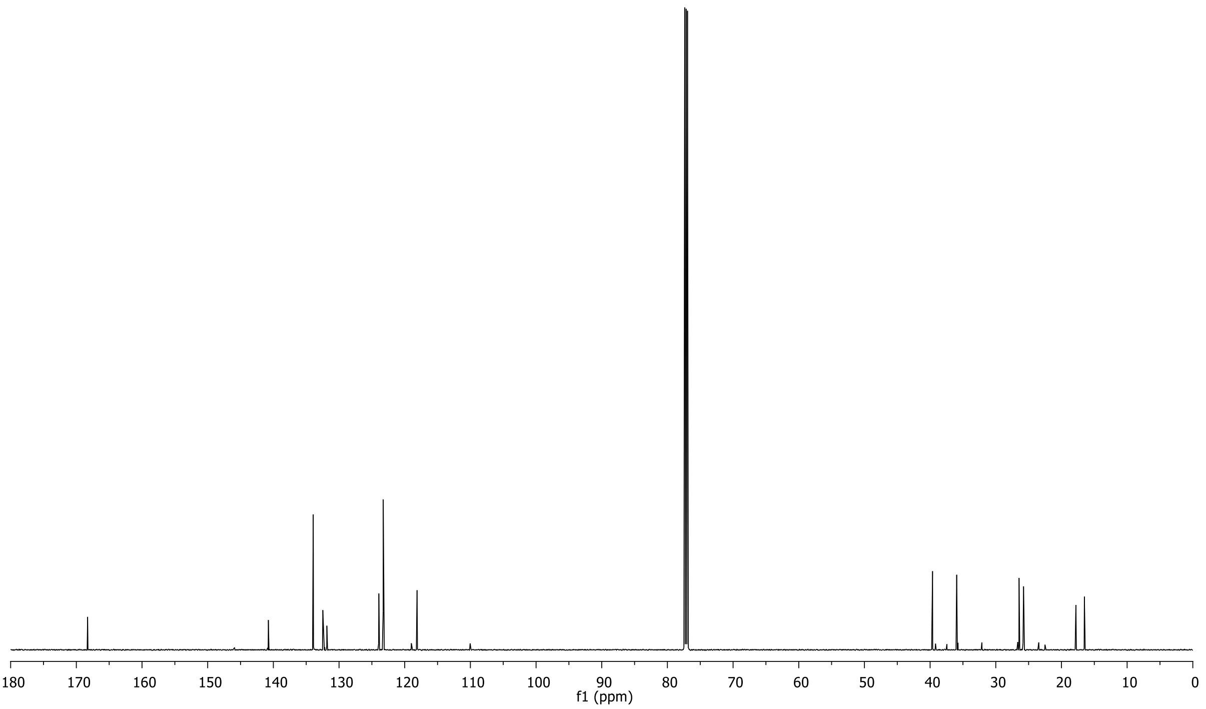

Figure S114. ^{13}C NMR spectrum (150 MHz, $CDCl_3$) of **67**.

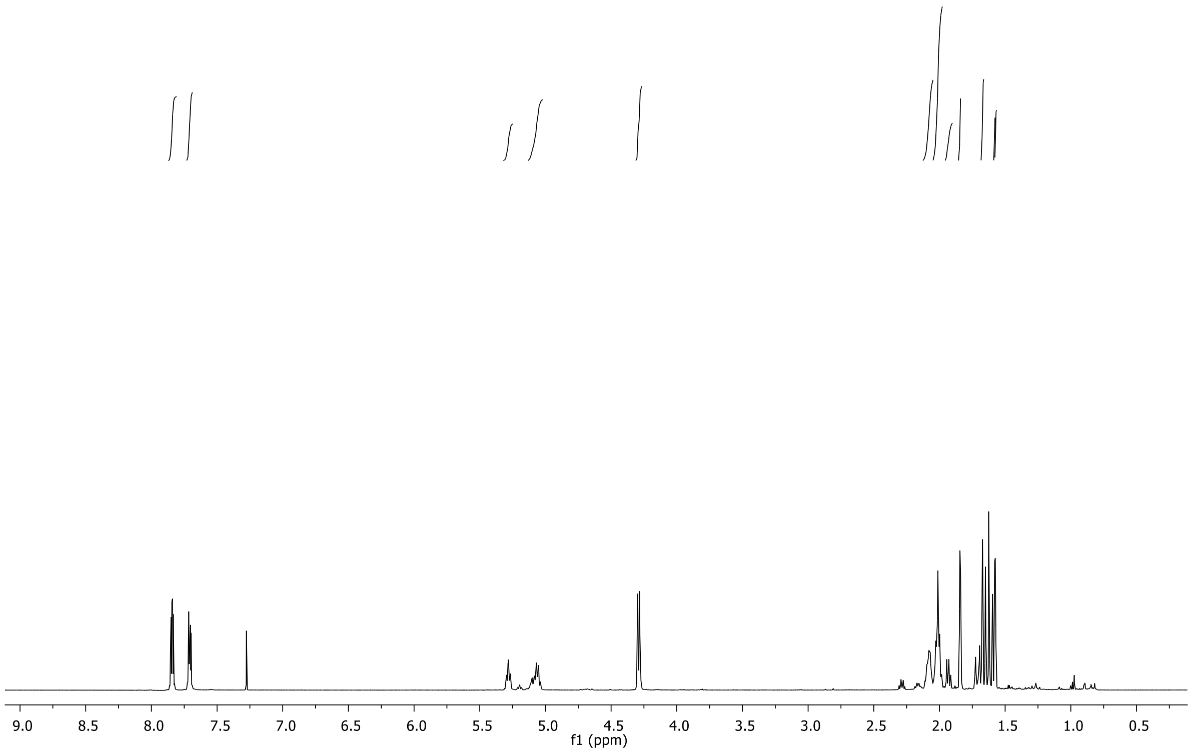

Figure S115. ^{1}H NMR spectrum (500 MHz, $CDCl_3$) of **68**.

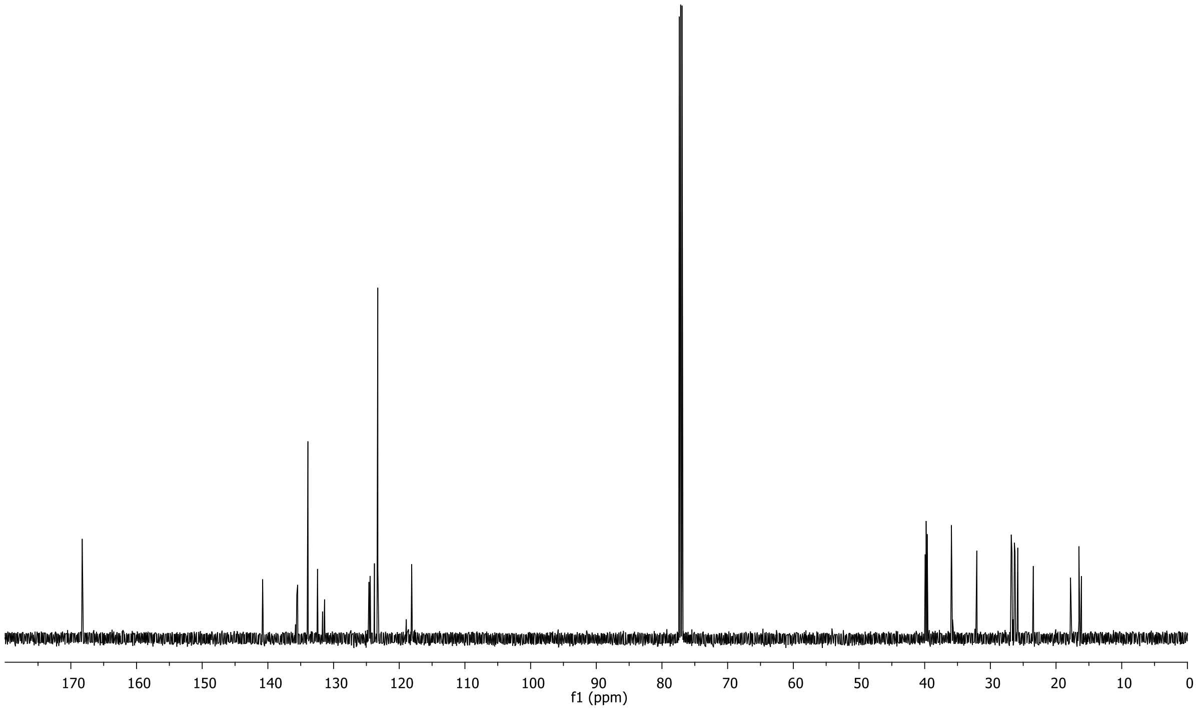

Figure S116. ^{13}C NMR spectrum (150 MHz, $CDCl_3$) of **68**.

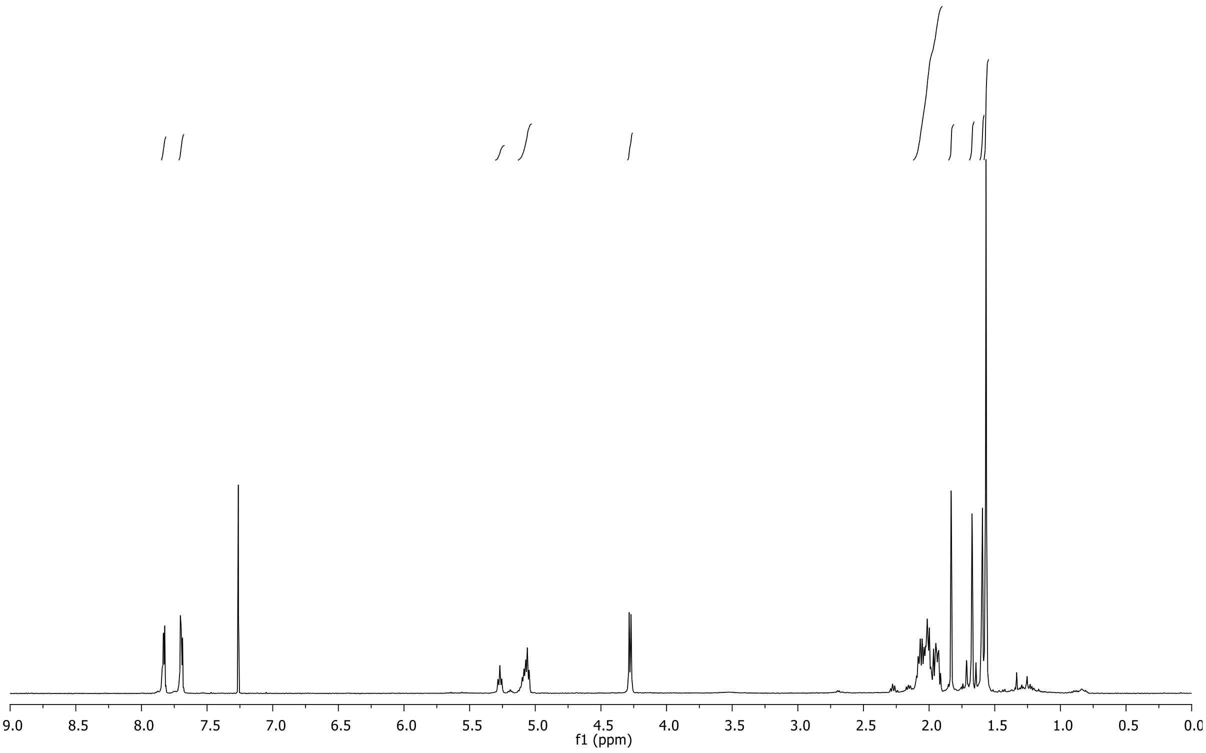

Figure S117. ^{1}H NMR spectrum (500 MHz, $CDCl_3$) of **69**.

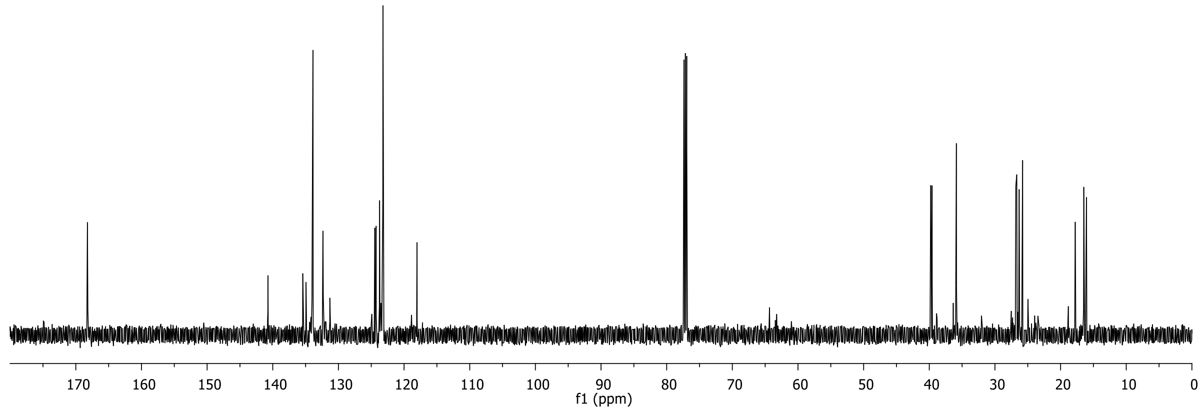

Figure S118. ^{13}C NMR spectrum (150 MHz, $CDCl_3$) of **69**.

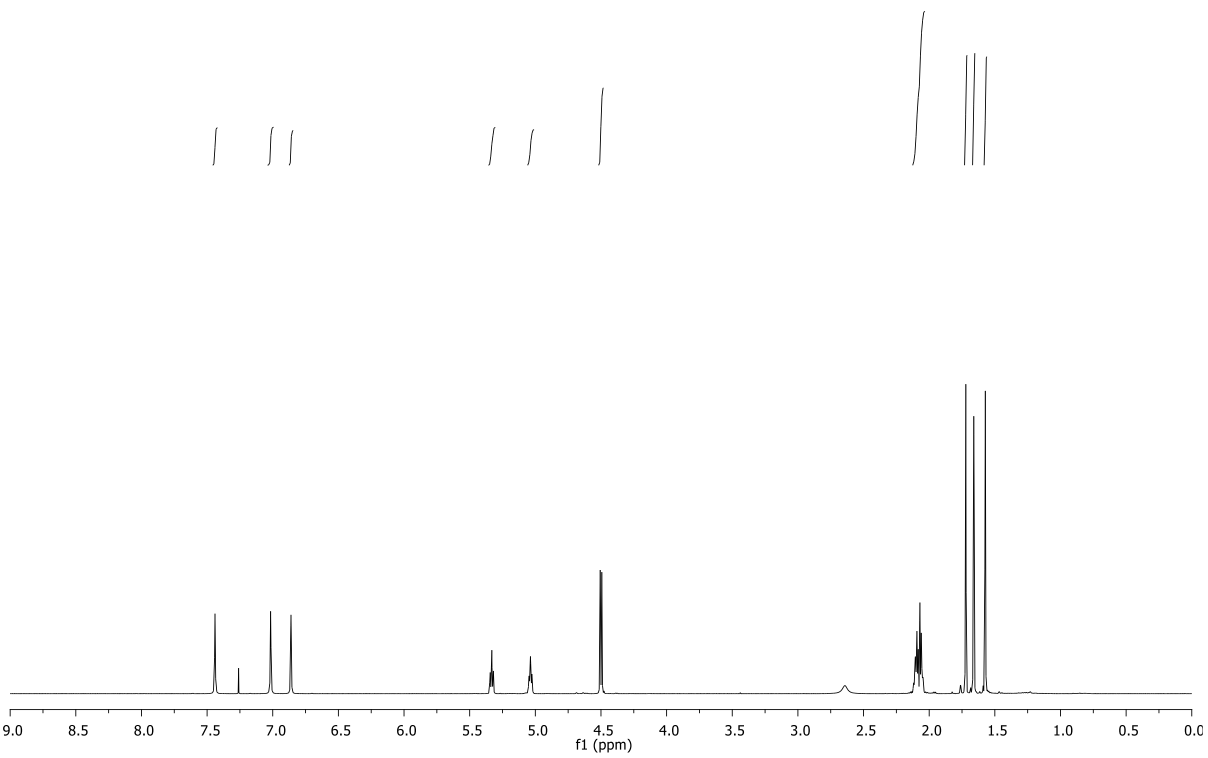

Figure S119. ^{1}H NMR spectrum (600 MHz, $CDCl_3$) of **70**.

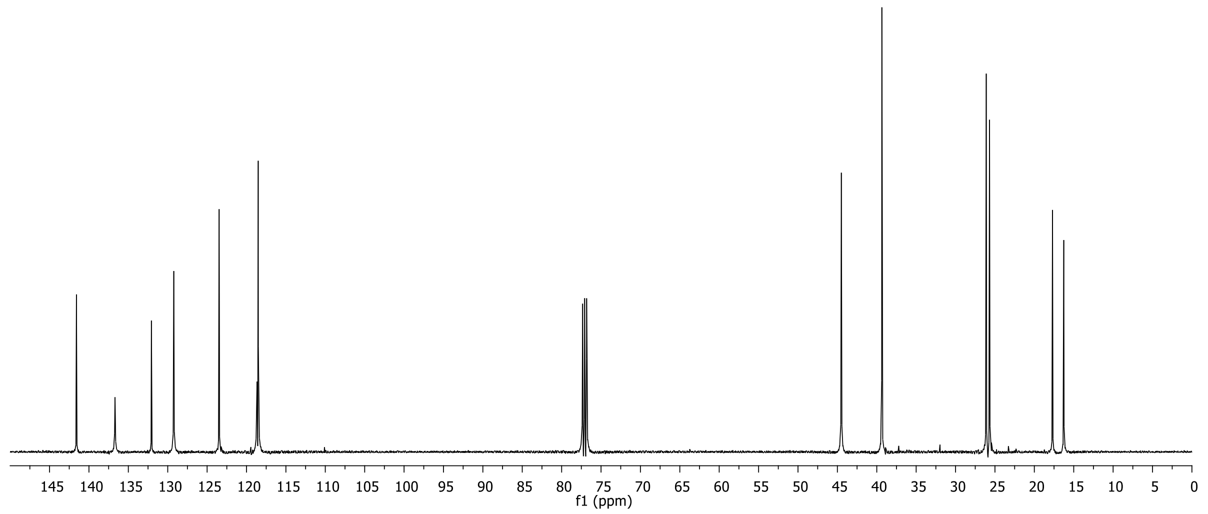

Figure S120. ^{13}C NMR spectrum (150 MHz, $CDCl_3$) of **70**.

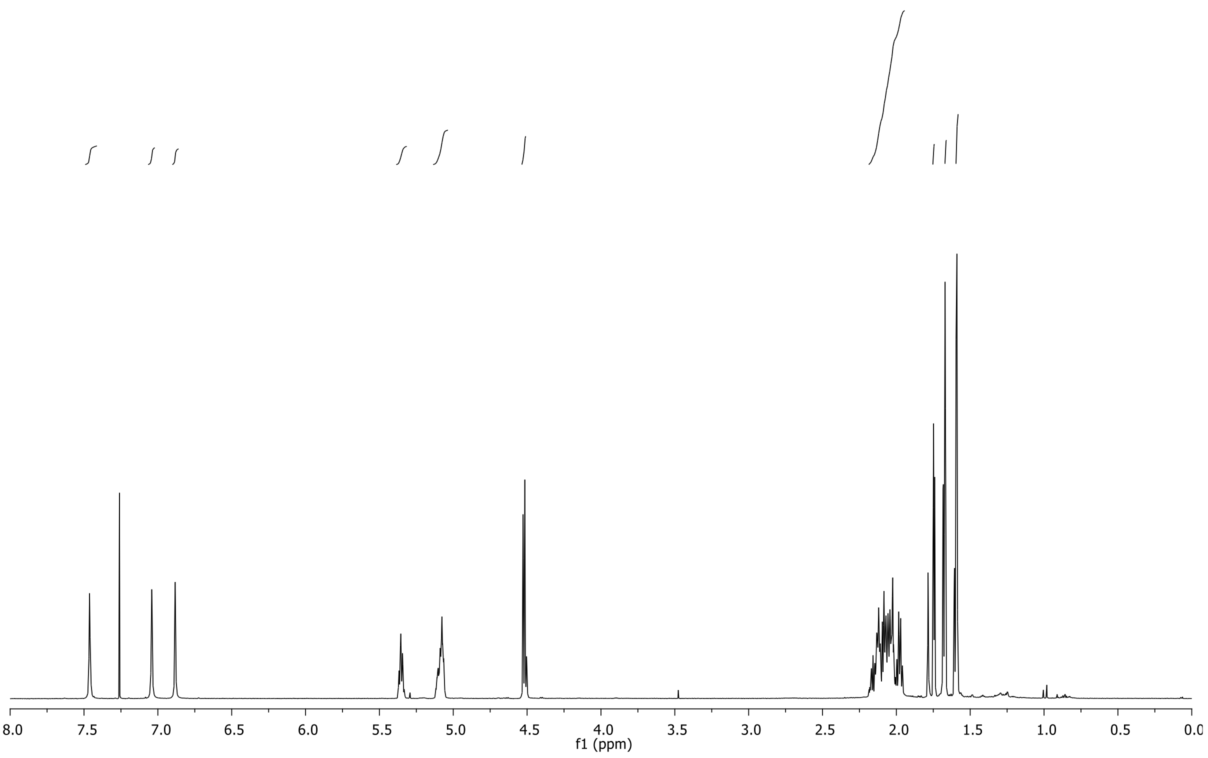

Figure S121. ^{1}H NMR spectrum (600 MHz, $CDCl_3$) of **71**.

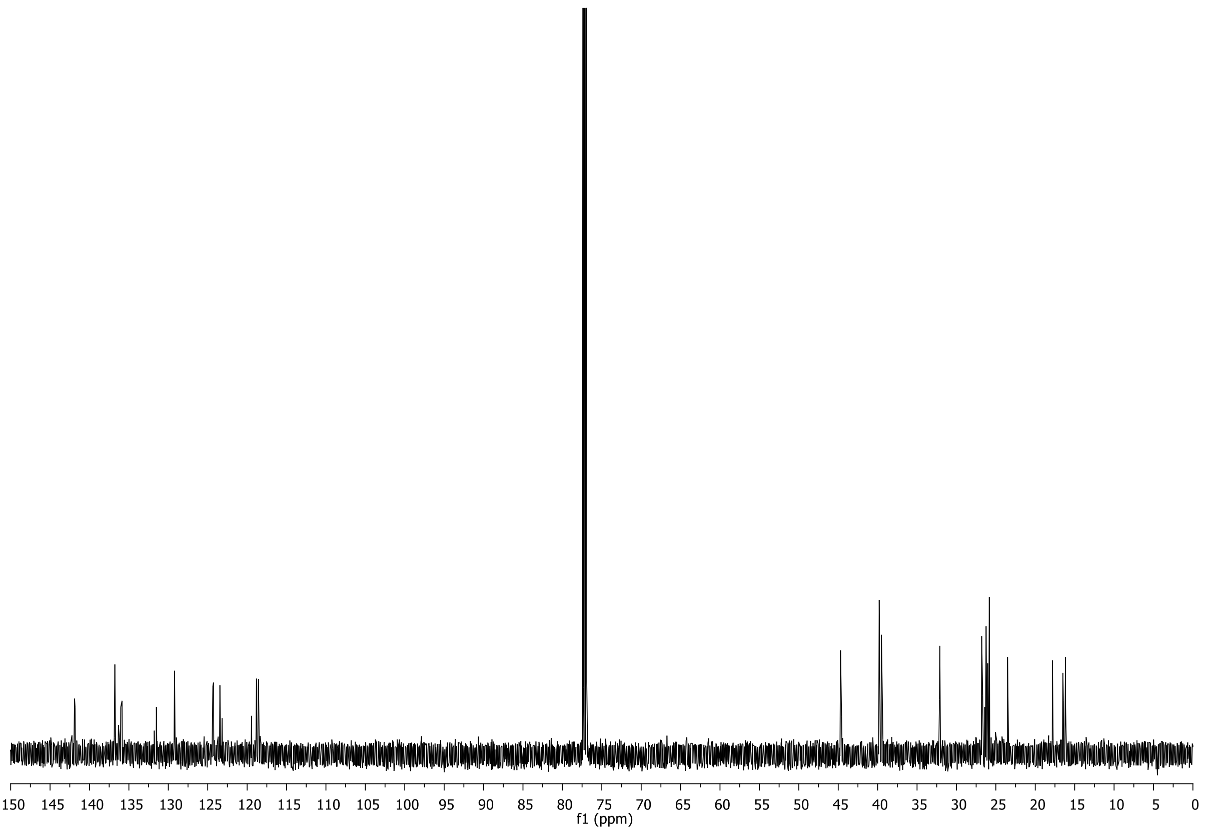

Figure S122. ^{13}C NMR spectrum (150 MHz, $CDCl_3$) of **71**.

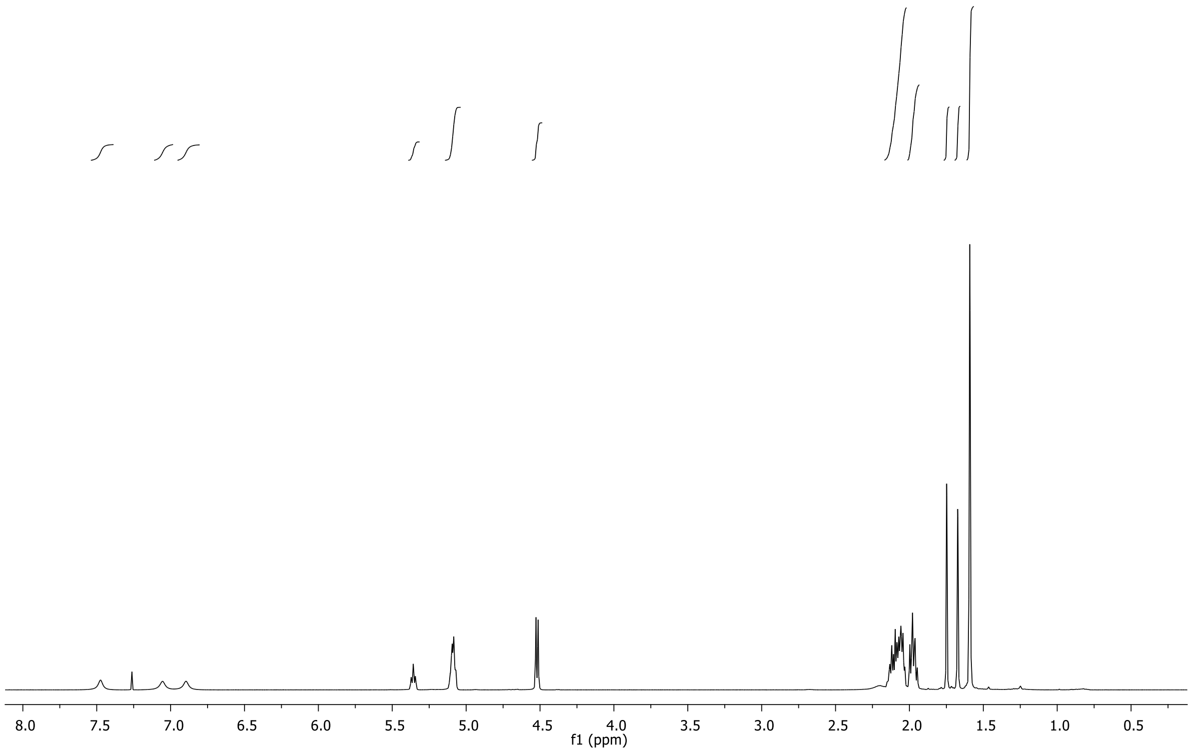

Figure S123. ^{1}H NMR spectrum (150 MHz, $CDCl_3$) of **72**.

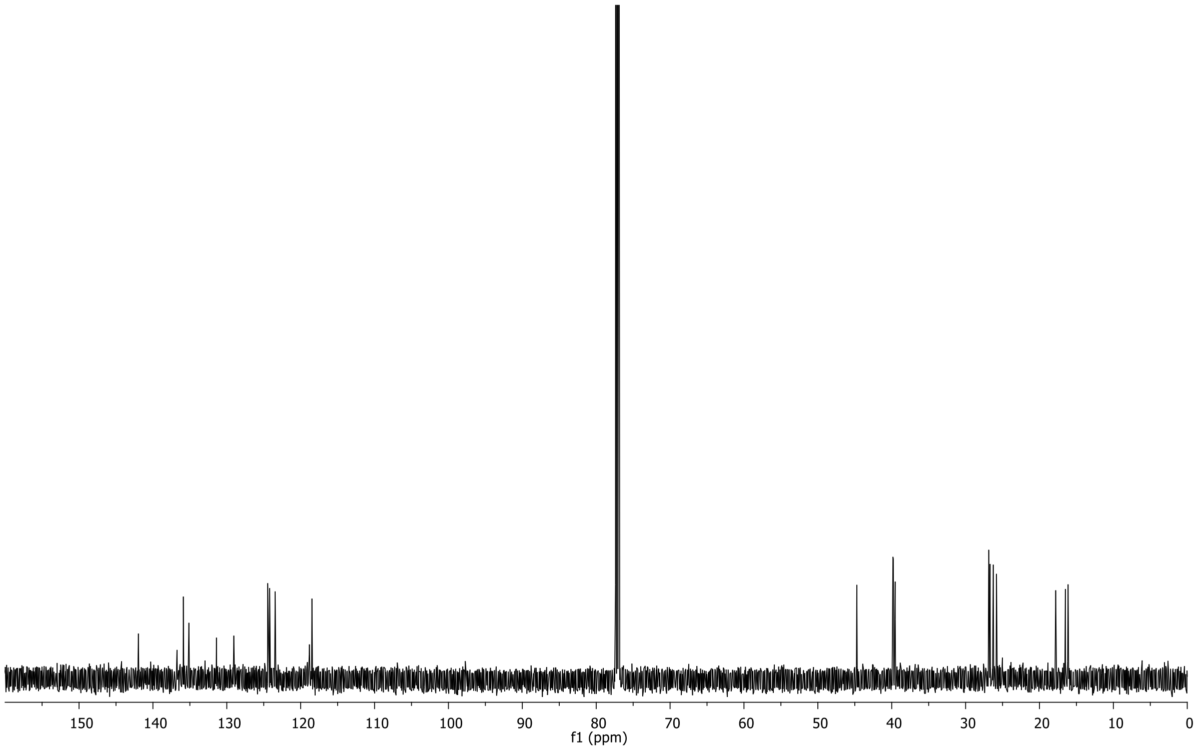

Figure S124. ^{13}C NMR spectrum (150 MHz, $CDCl_3$) of **72**.

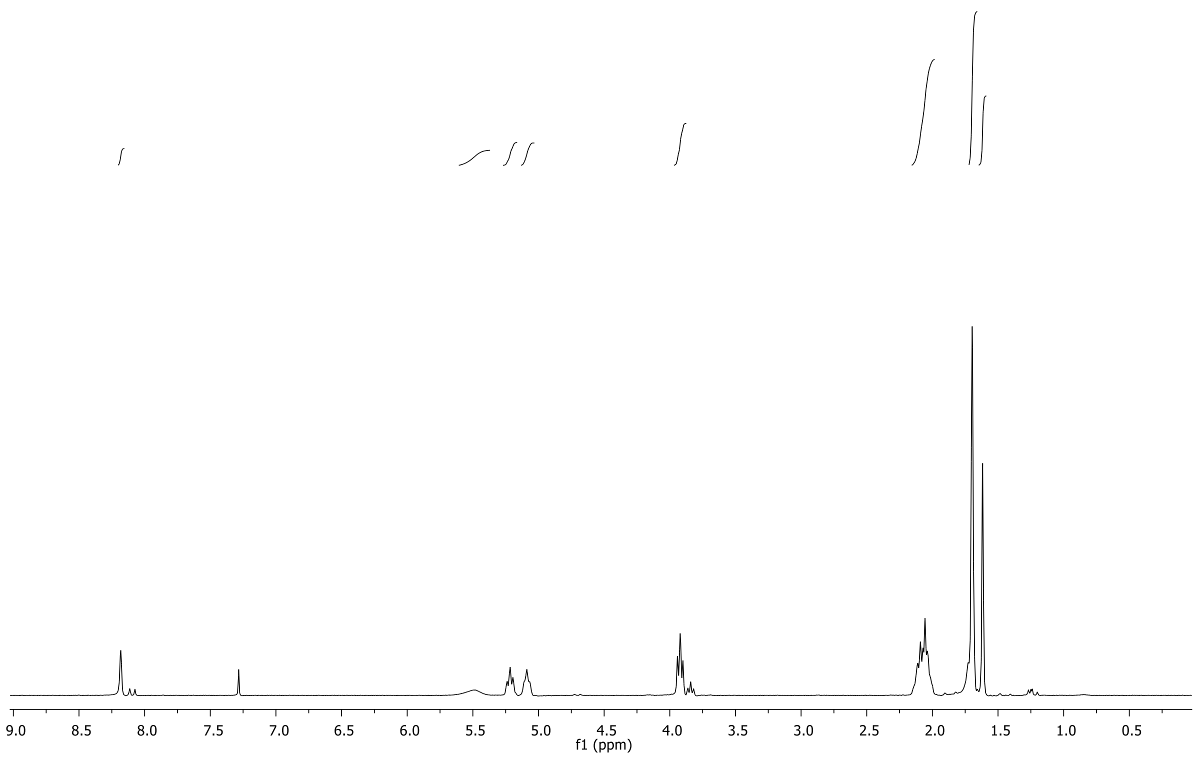

Figure S125. ^{1}H NMR spectrum (300 MHz, $CDCl_3$) of **73**.

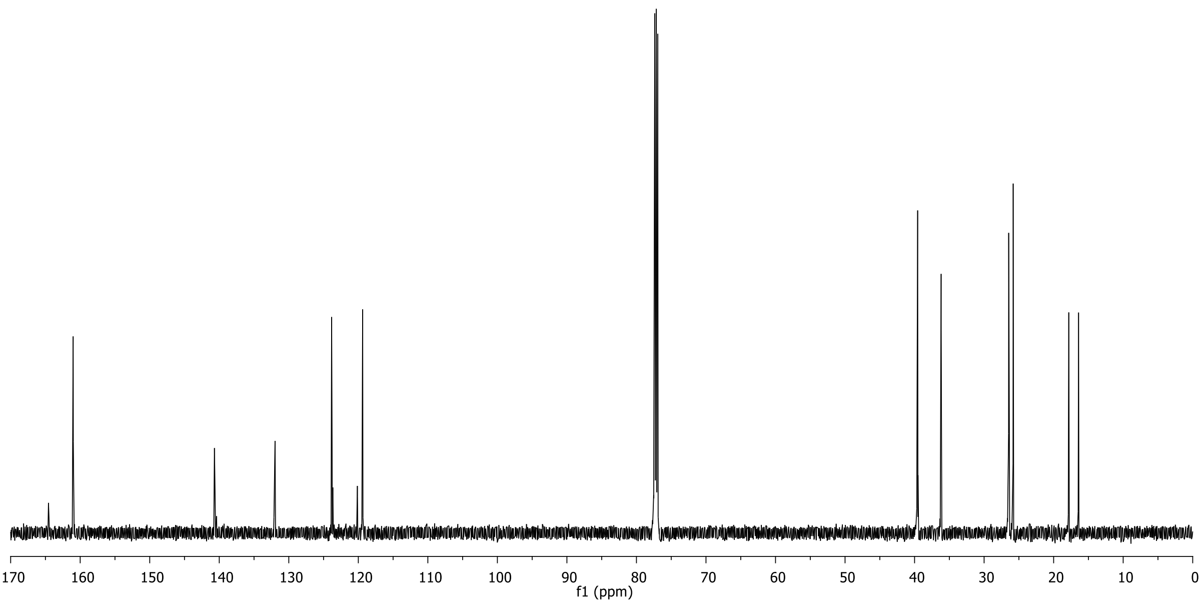

Figure S126. ^{13}C NMR spectrum (150 MHz, $CDCl_3$) of **73**.

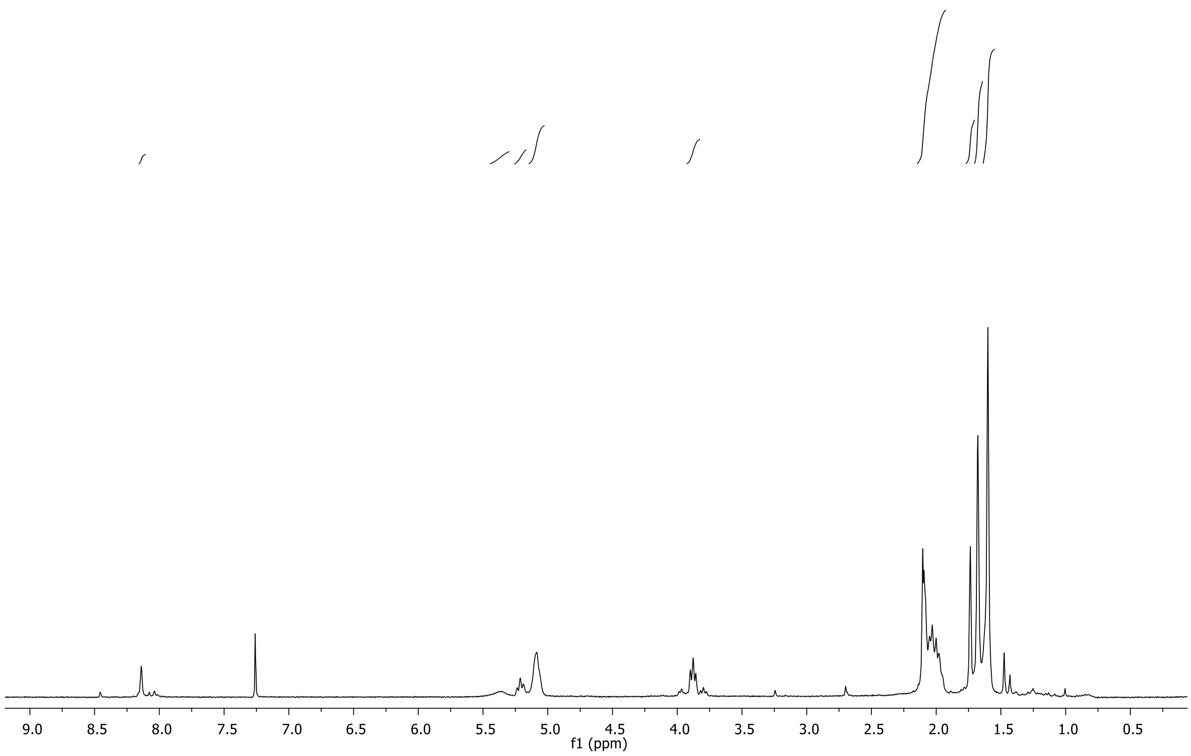

Figure S127. ^{1}H NMR spectrum (300 MHz, $CDCl_3$) of **74**.

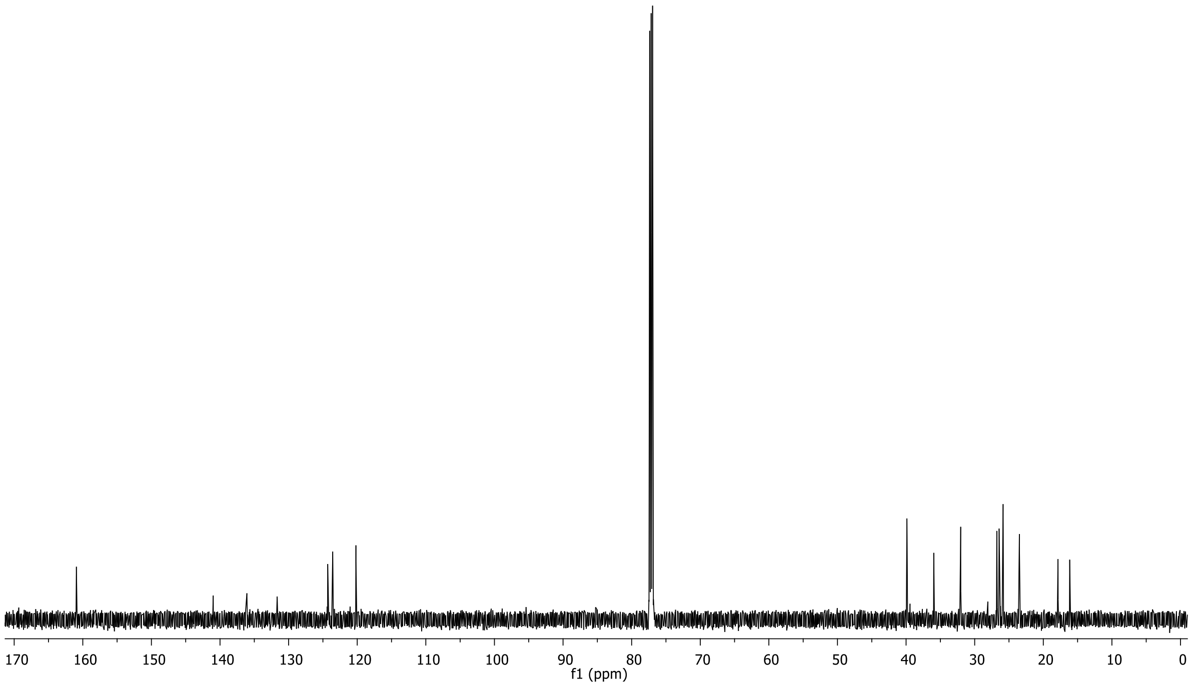

Figure S128. ^{13}C NMR spectrum (150 MHz, $CDCl_3$) of **74**.

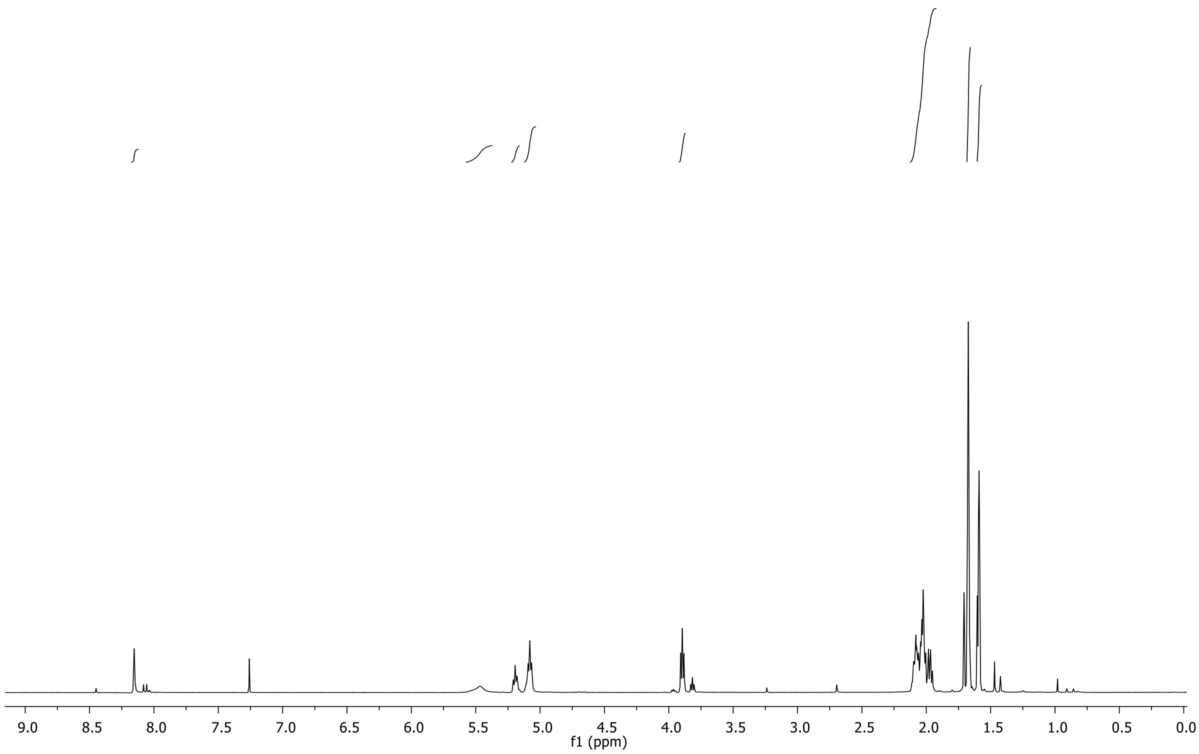

Figure S129. ^{1}H NMR spectrum (500 MHz, $CDCl_3$) of **75**.

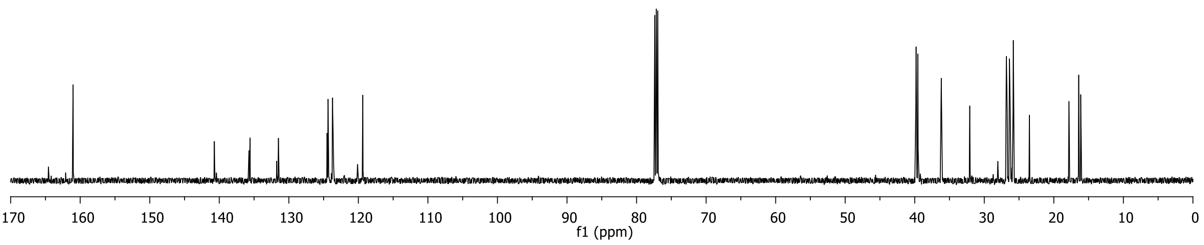

Figure S130. ^{13}C NMR spectrum (150 MHz, $CDCl_3$) of **75**.

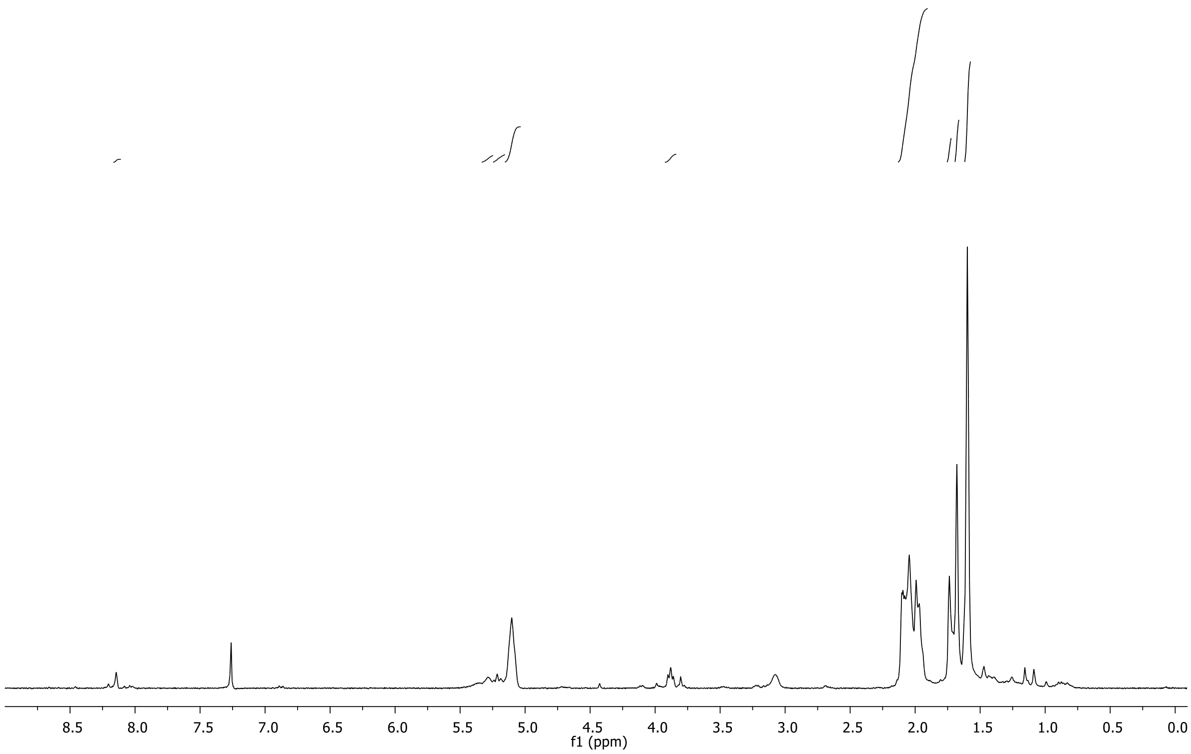

Figure S131. ^{13}C NMR spectrum (300 MHz, $CDCl_3$) of **76**.

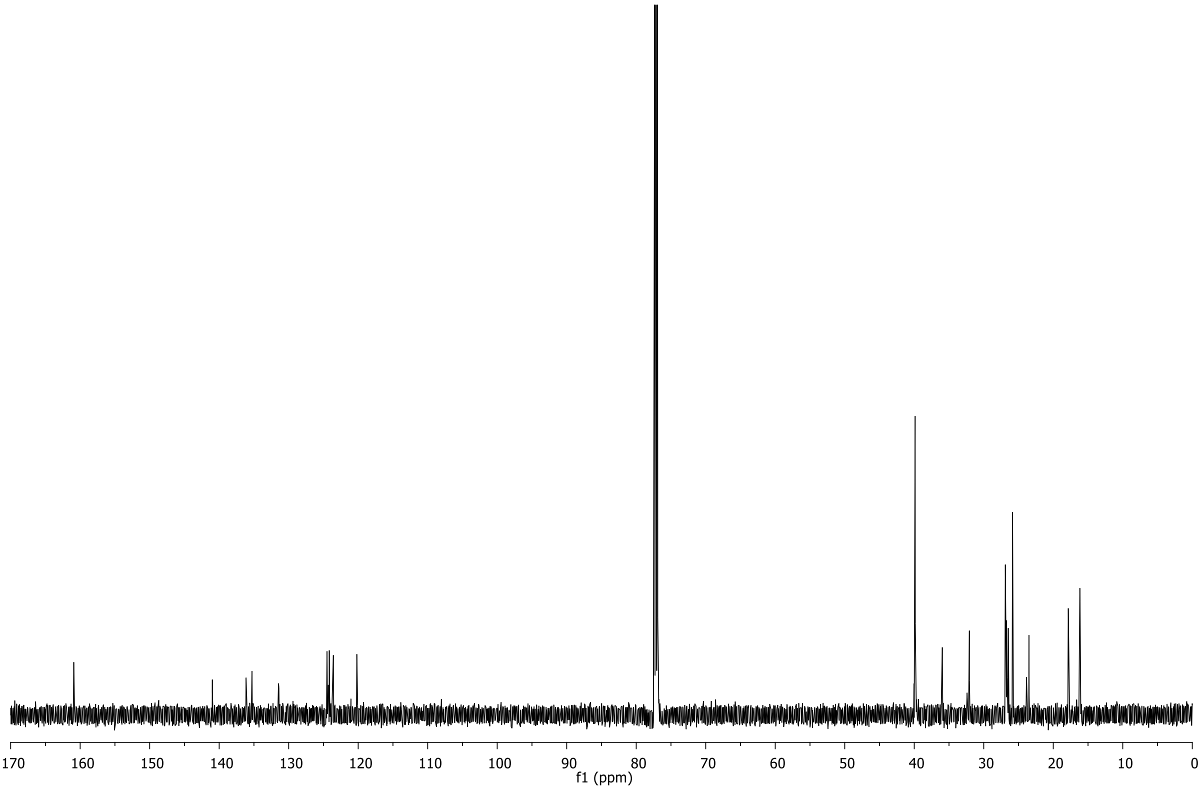

Figure S132. ^{13}C NMR spectrum (150 MHz, $CDCl_3$) of **76**.

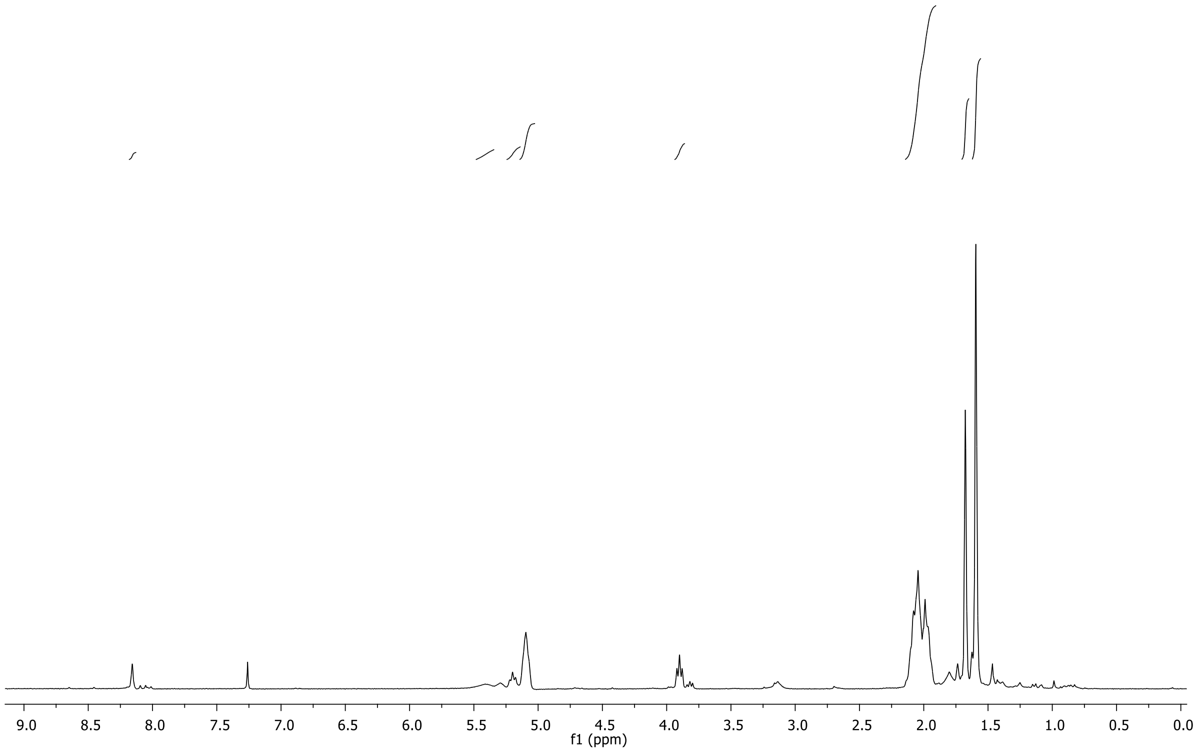

Figure S133. ^{1}H NMR spectrum (300 MHz, $CDCl_3$) of **77**.

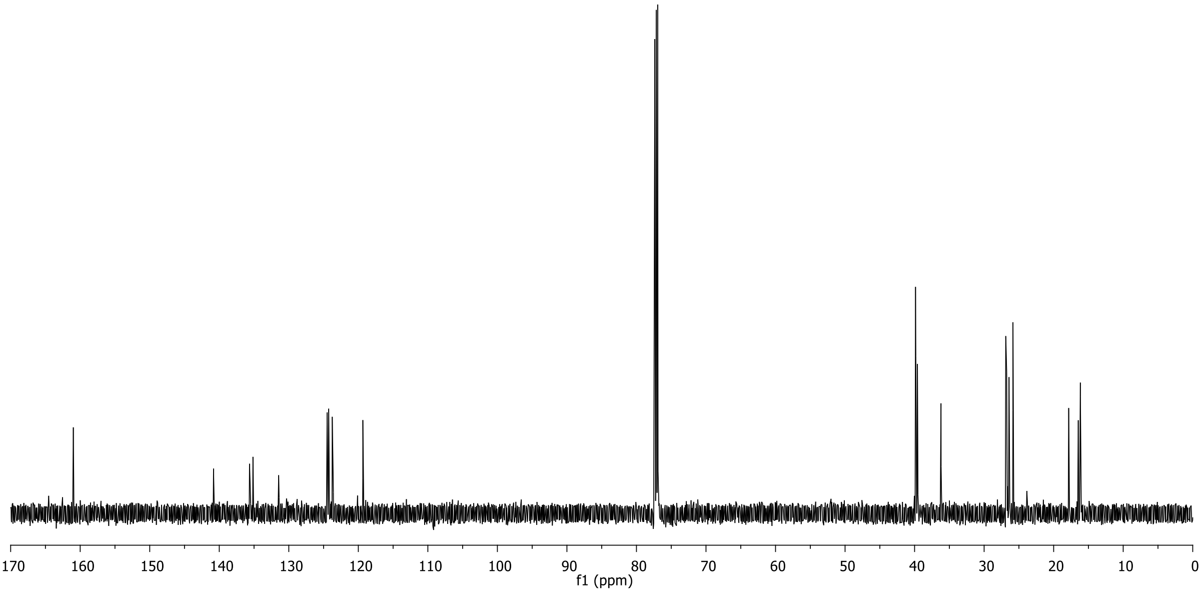

Figure S134. ^{13}C NMR spectrum (150 MHz, $CDCl_3$) of **77**.

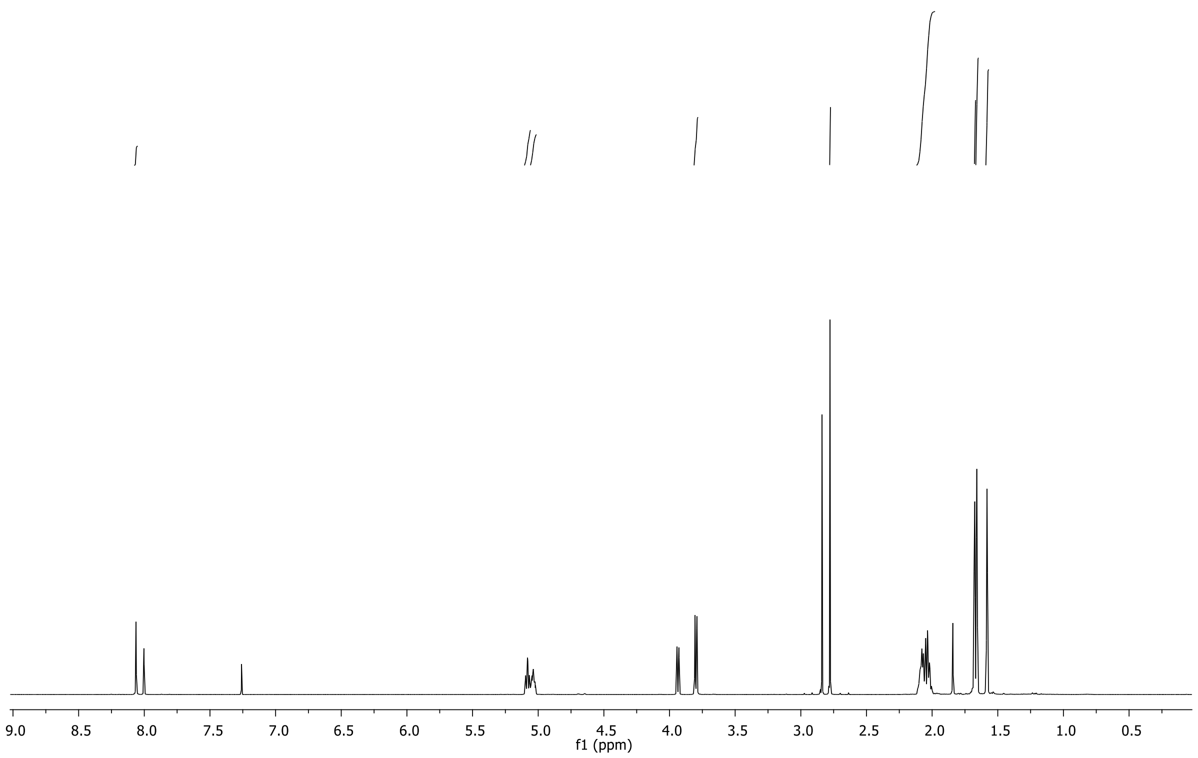

Figure S135. ^{1}H NMR spectrum (500 MHz, $CDCl_3$) of **78**.

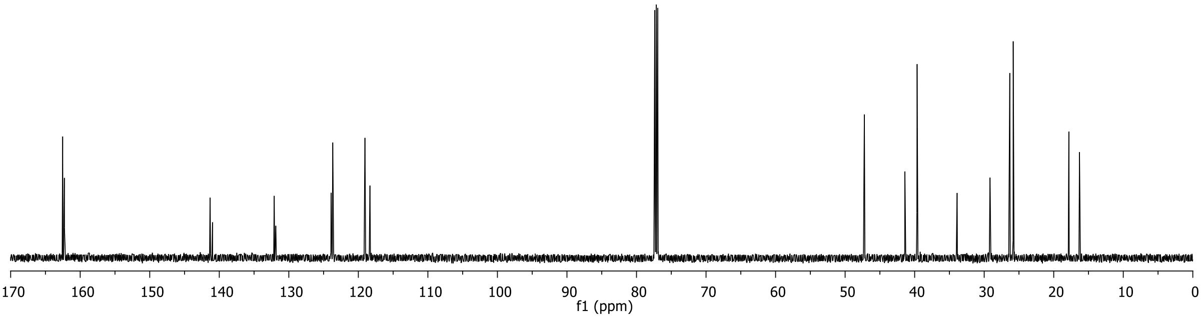

Figure S136. ^{13}C NMR spectrum (150 MHz, $CDCl_3$) of **78**.

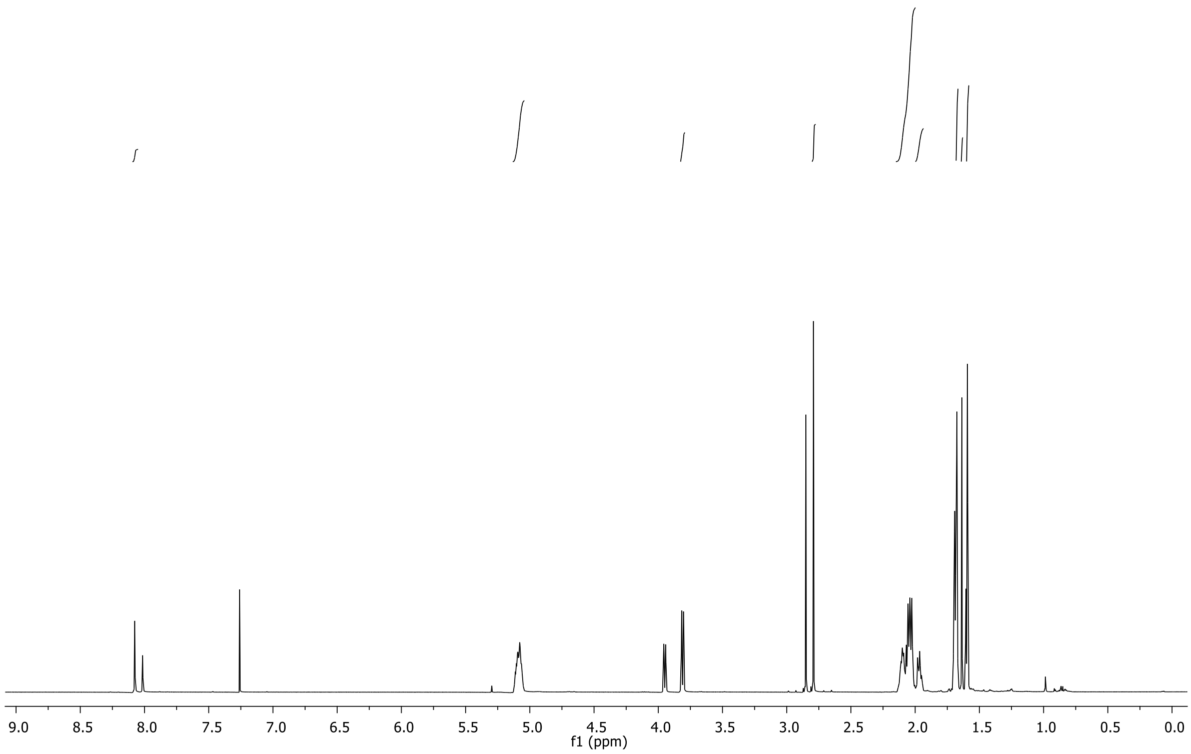

Figure S137. ^{1}H NMR spectrum (500 MHz, $CDCl_3$) of **79**.

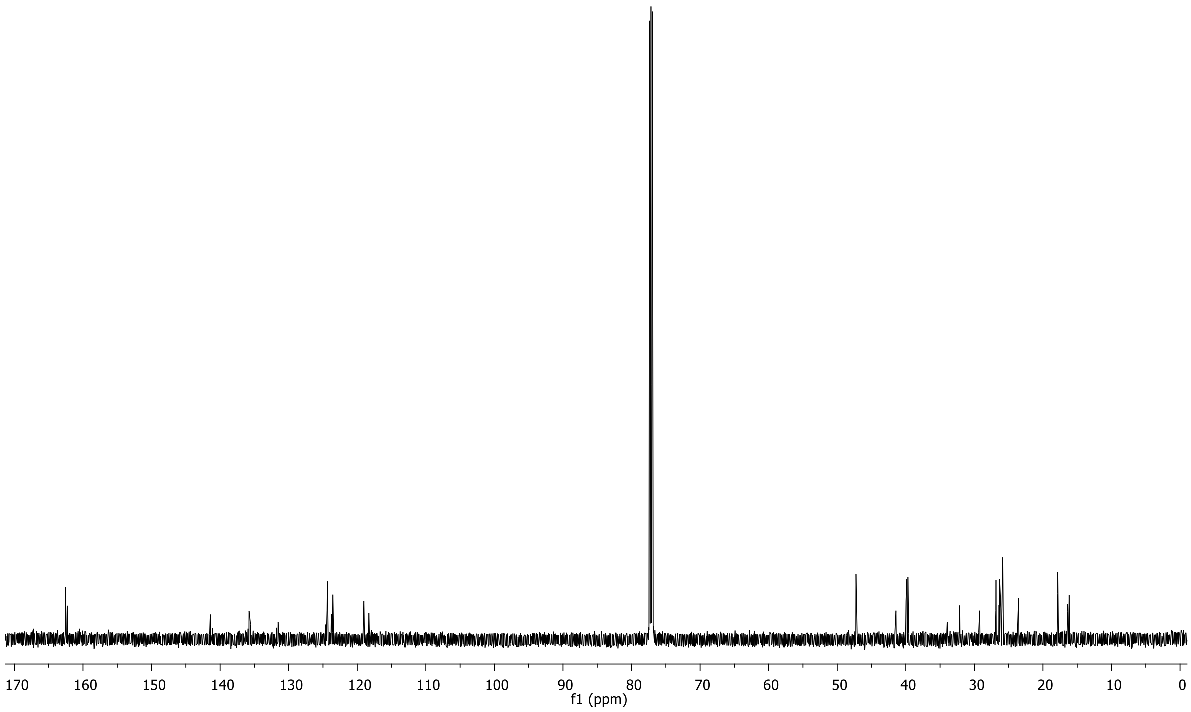

Figure S138. ^{13}C NMR spectrum (150 MHz, $CDCl_3$) of **79**.

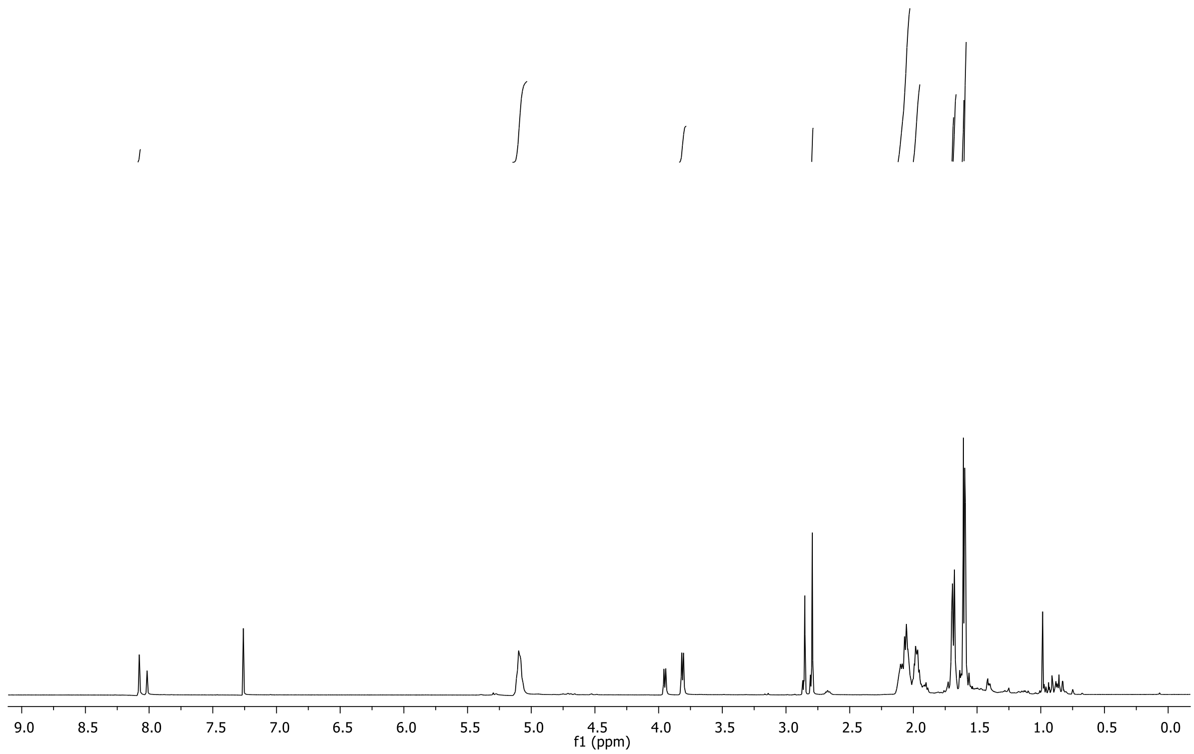

Figure S139. ^{1}H NMR spectrum (500 MHz, $CDCl_3$) of **80**.

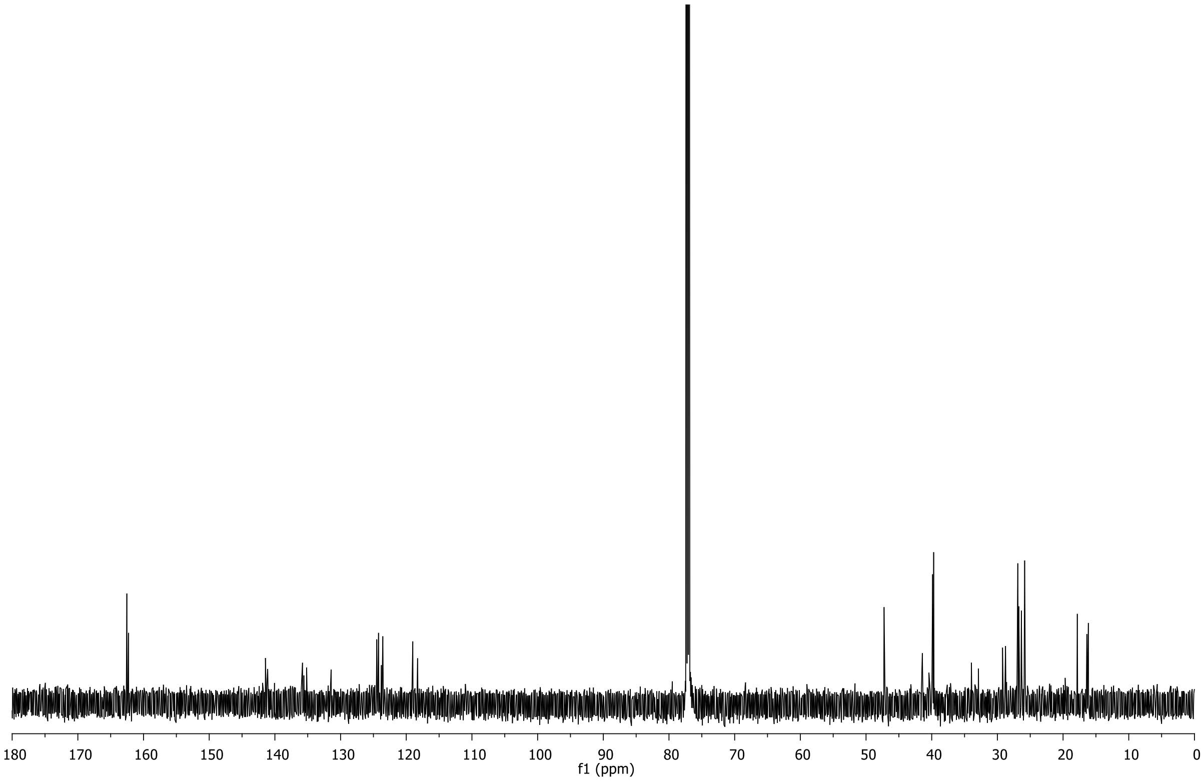

Figure S140. ^{13}C NMR spectrum (150 MHz, $CDCl_3$) of **80**.

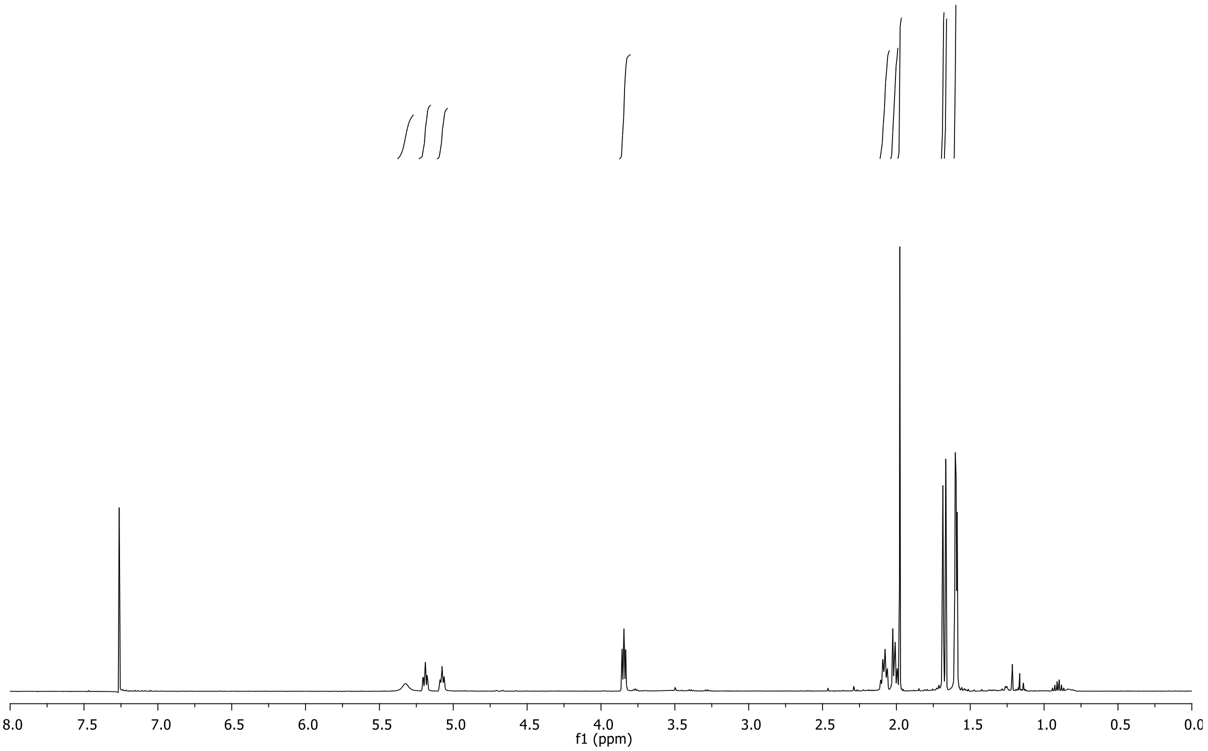

Figure S141. ^{1}H NMR spectrum (500 MHz, $CDCl_3$) of **81**.

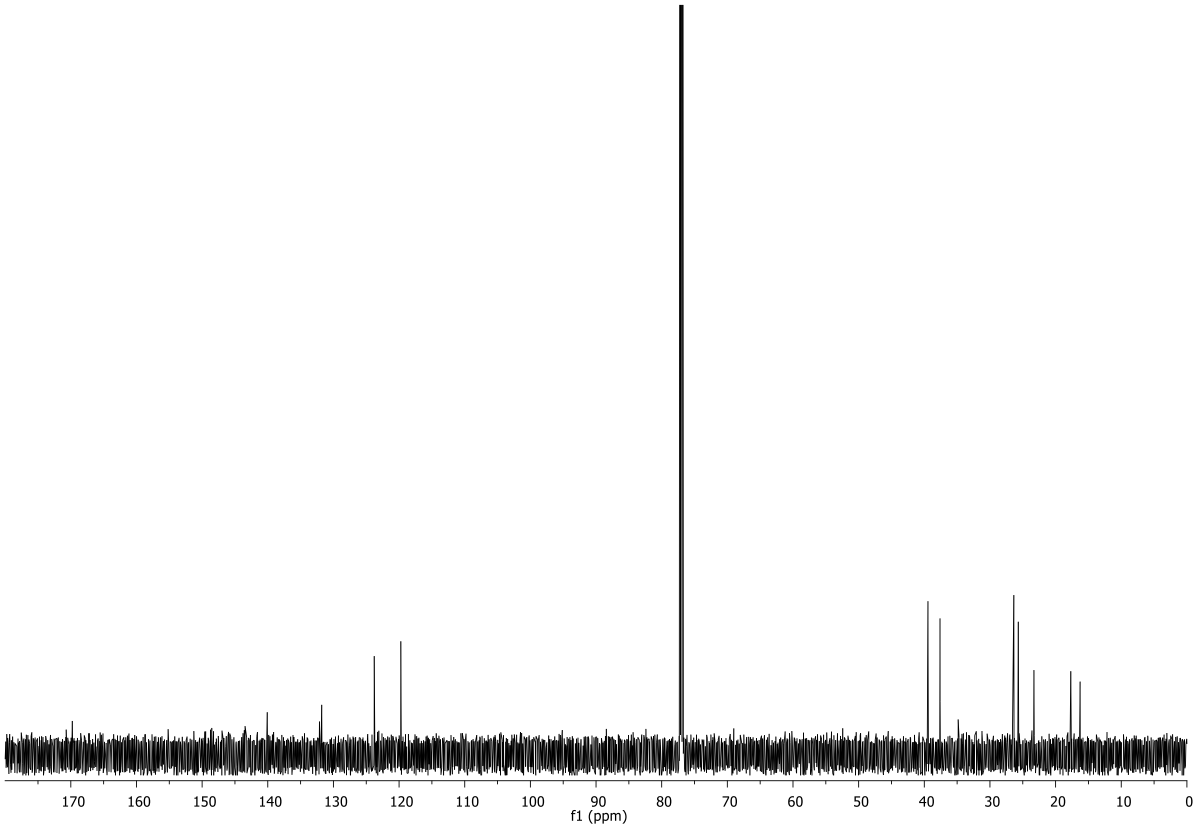

Figure S142. ^{13}C NMR spectrum (150 MHz, $CDCl_3$) of **81**.

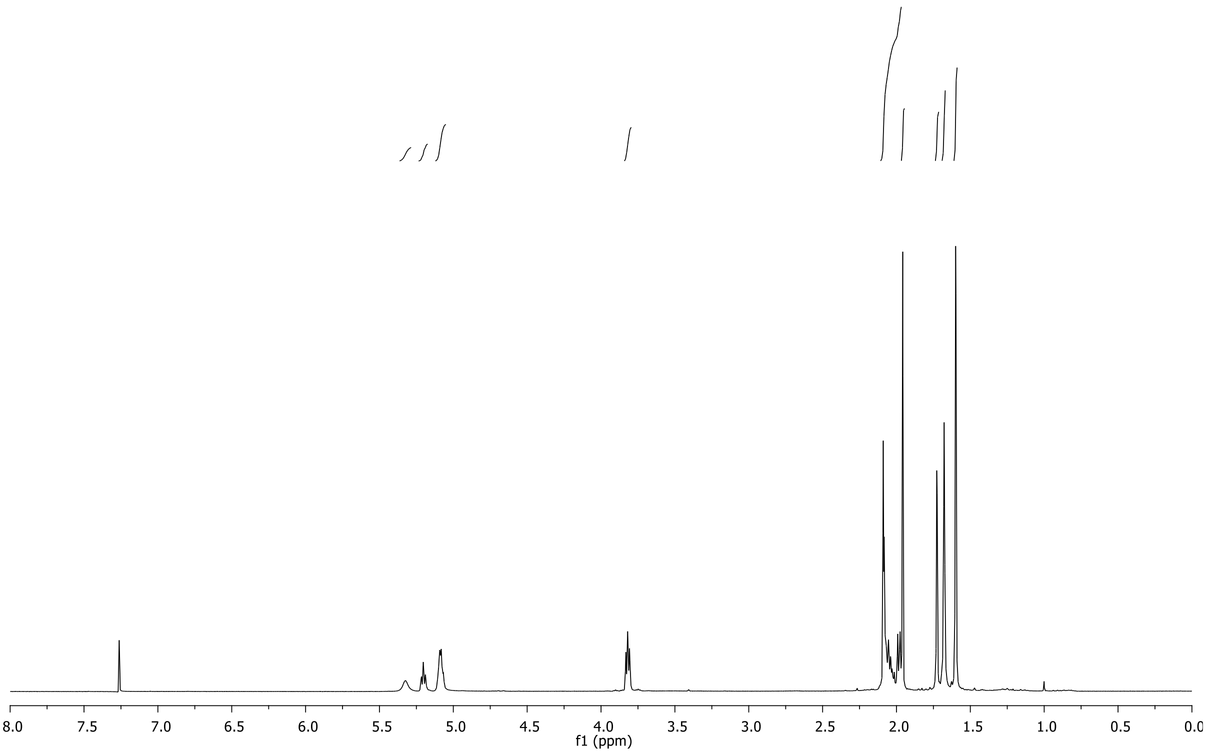

Figure S143. ^{1}H NMR spectrum (500 MHz, $CDCl_3$) of **82**.

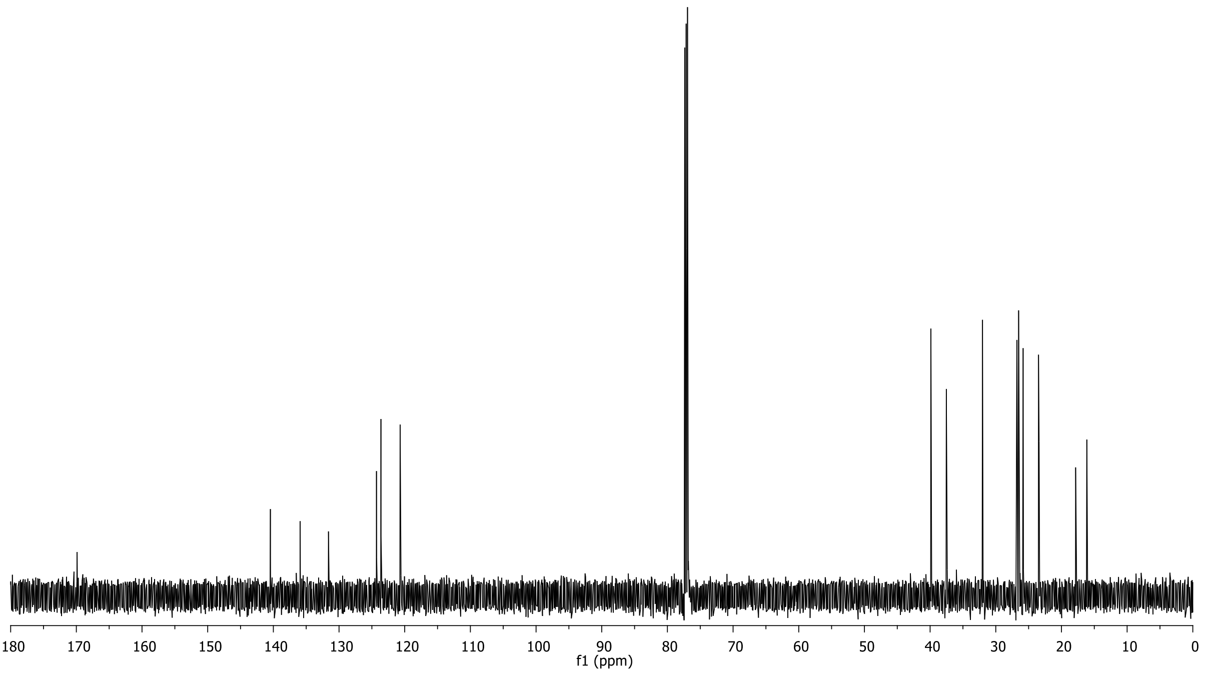

Figure S144. ^{13}C NMR spectrum (150 MHz, $CDCl_3$) of **82**.

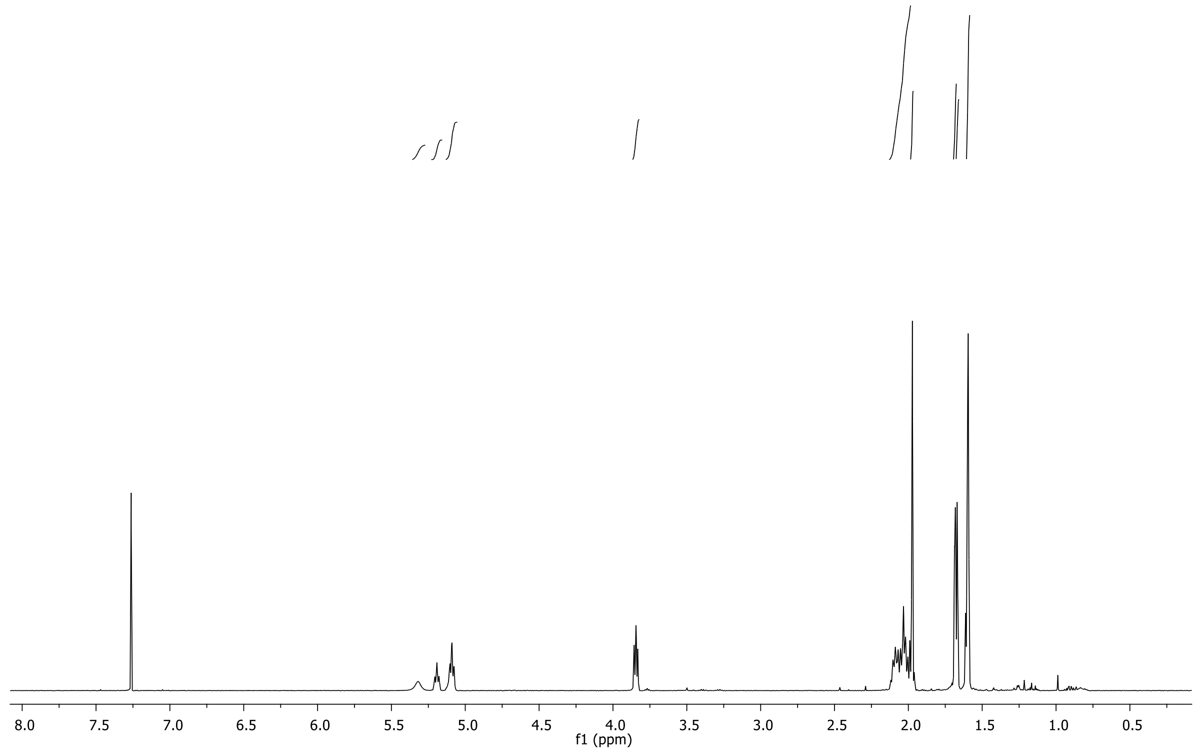

Figure S145. ^{1}H NMR spectrum (500 MHz, $CDCl_3$) of **83**.

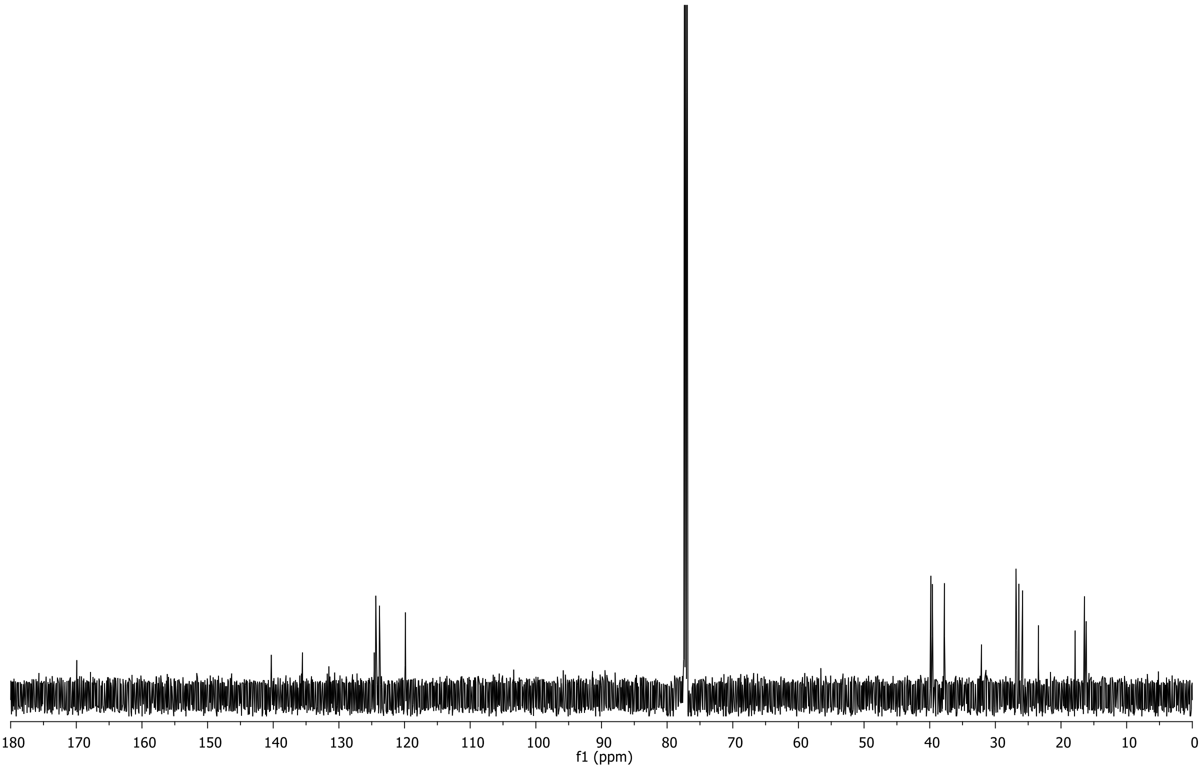

Figure S146. ^{13}C NMR spectrum (150 MHz, $CDCl_3$) of **83**.

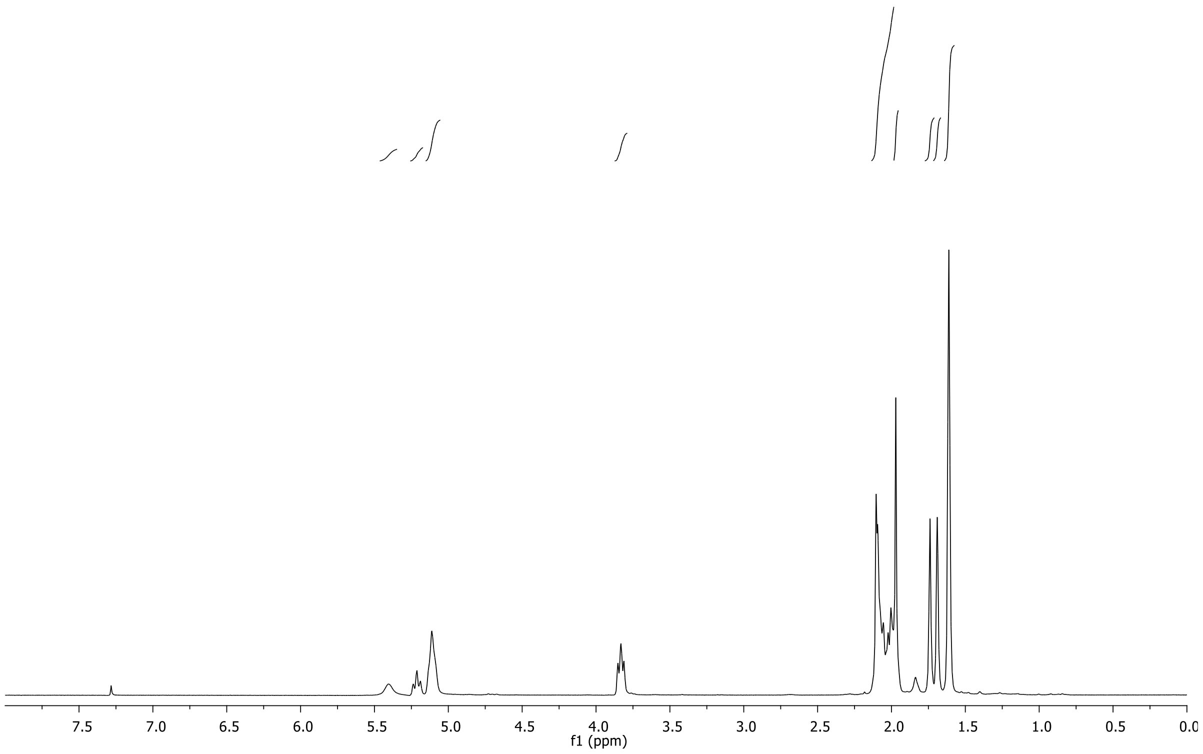

Figure S147. ^{1}H NMR spectrum (300 MHz, $CDCl_3$) of **84**.

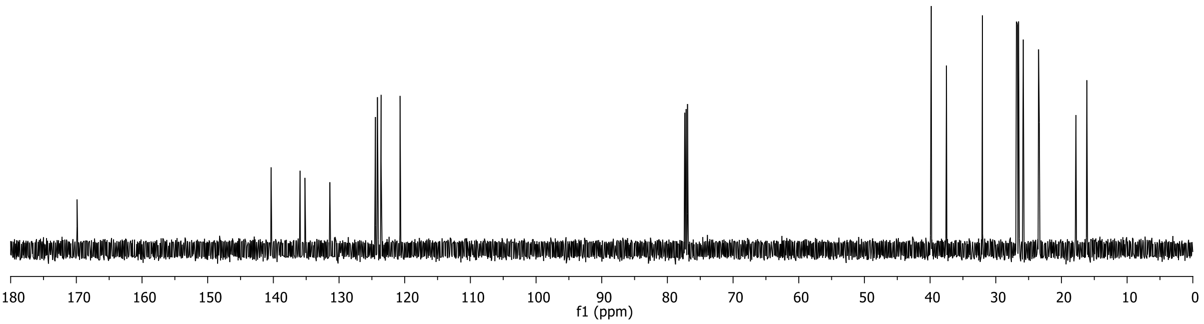

Figure S148. ^{13}C NMR spectrum (150 MHz, $CDCl_3$) of **84**.

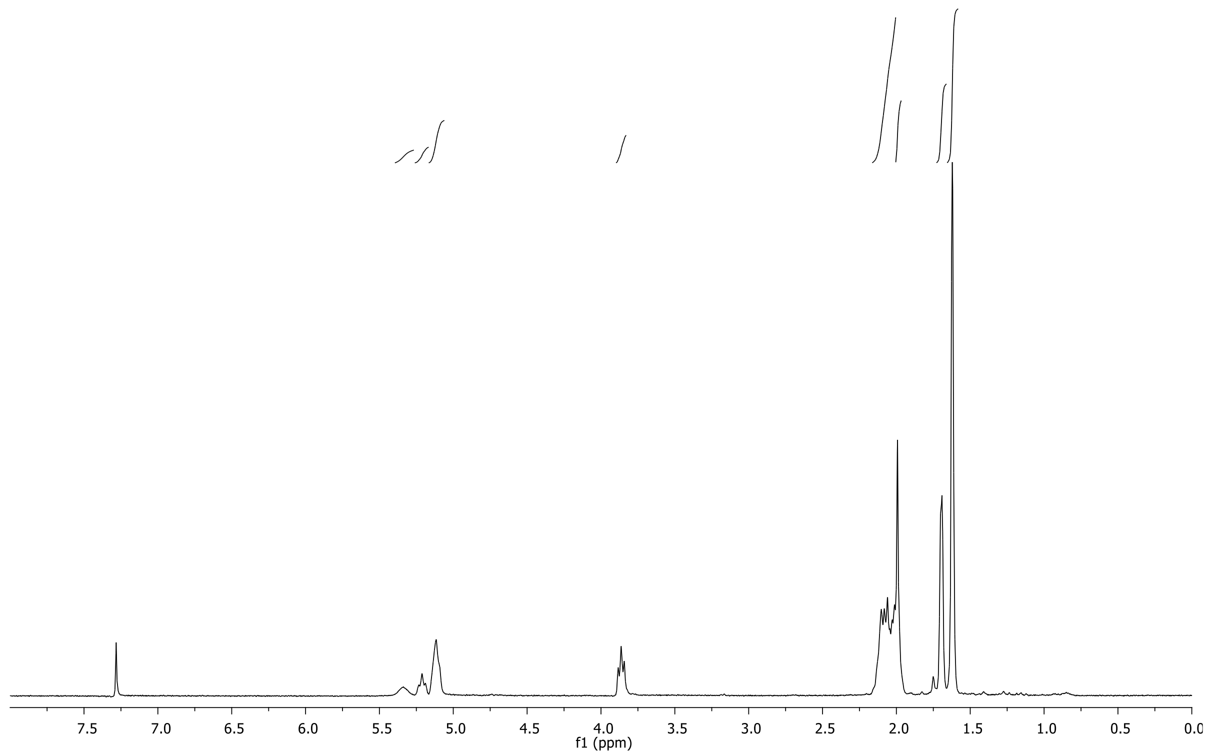

Figure S149. ^{1}H NMR spectrum (300 MHz, $CDCl_3$) of **85**.

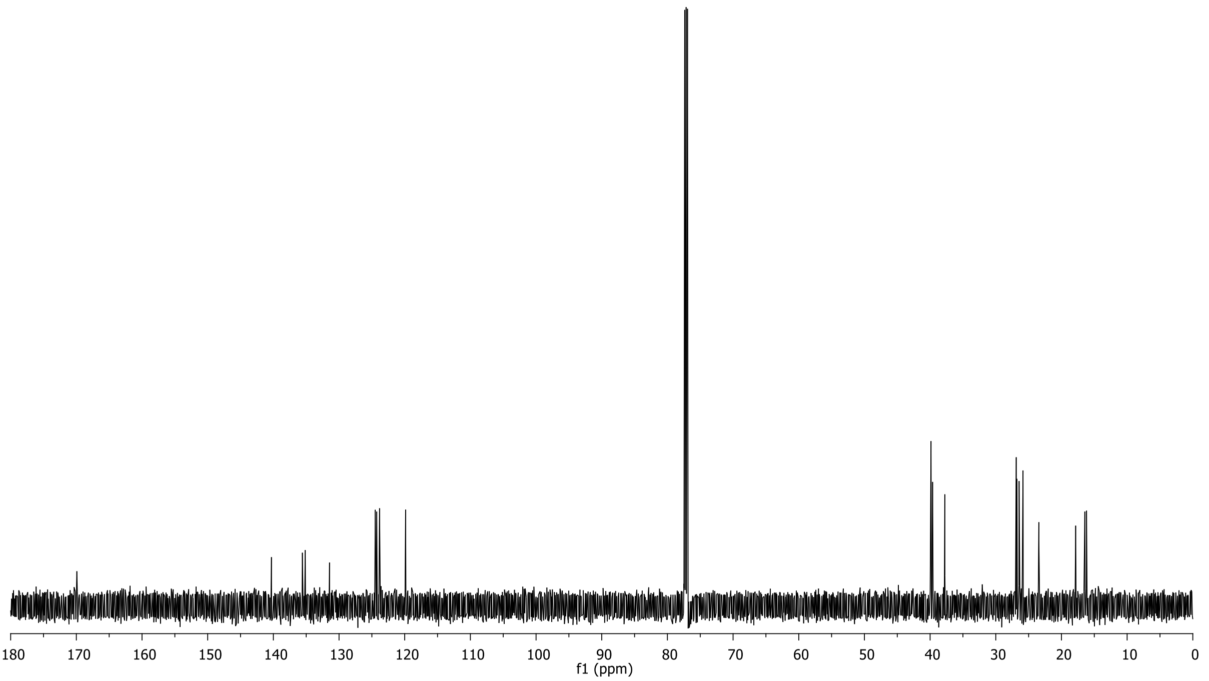

Figure S150. ^{13}C NMR spectrum (150 MHz, $CDCl_3$) of **85**.

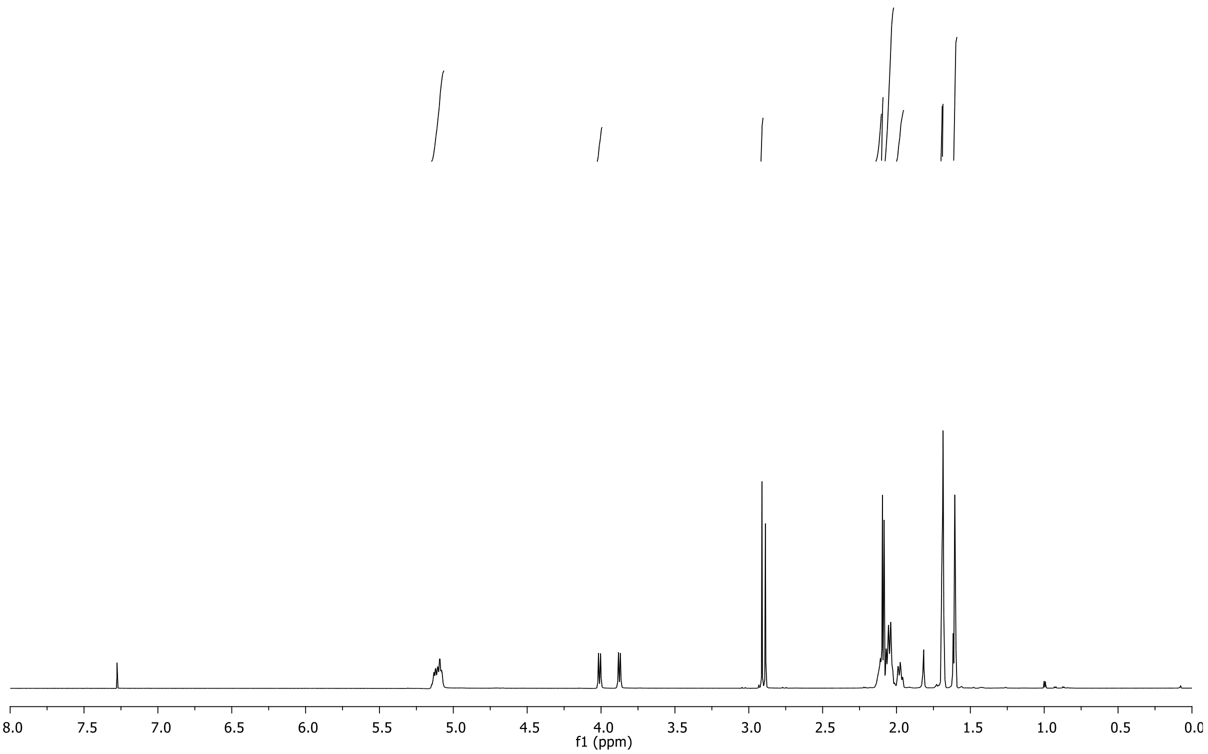

Figure S151. ^{1}H NMR spectrum (500 MHz, $CDCl_3$) of **86**.

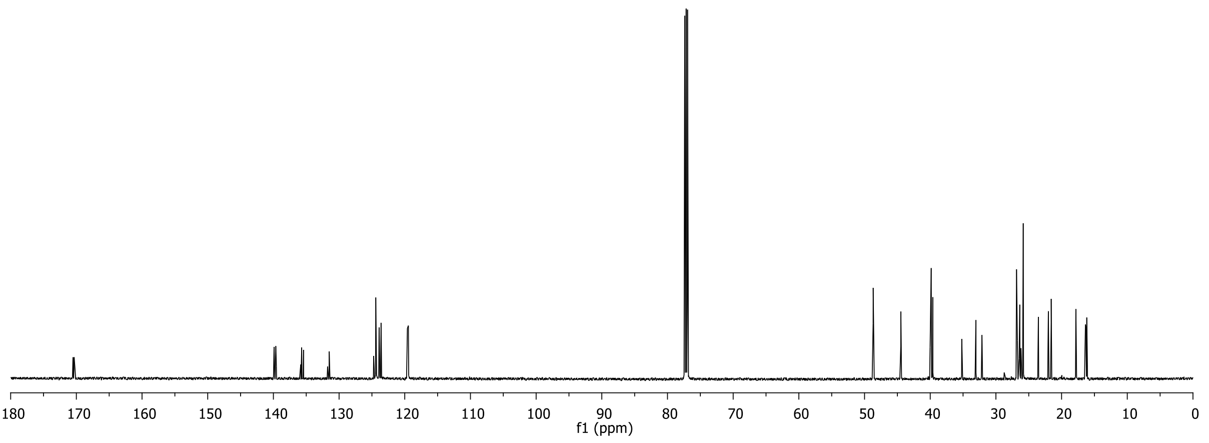

Figure S152. ^{13}C NMR spectrum (150 MHz, $CDCl_3$) of **86**.

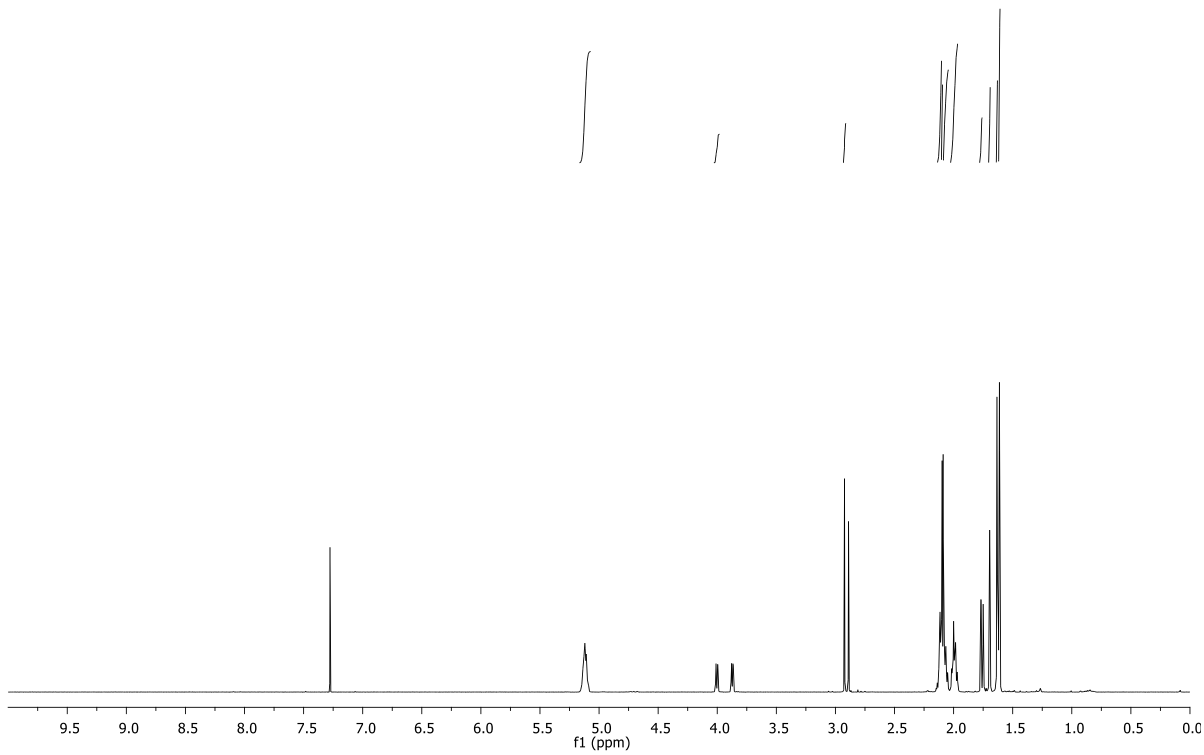

Figure S153. ^{1}H NMR spectrum (500 MHz, $CDCl_3$) of **87**.

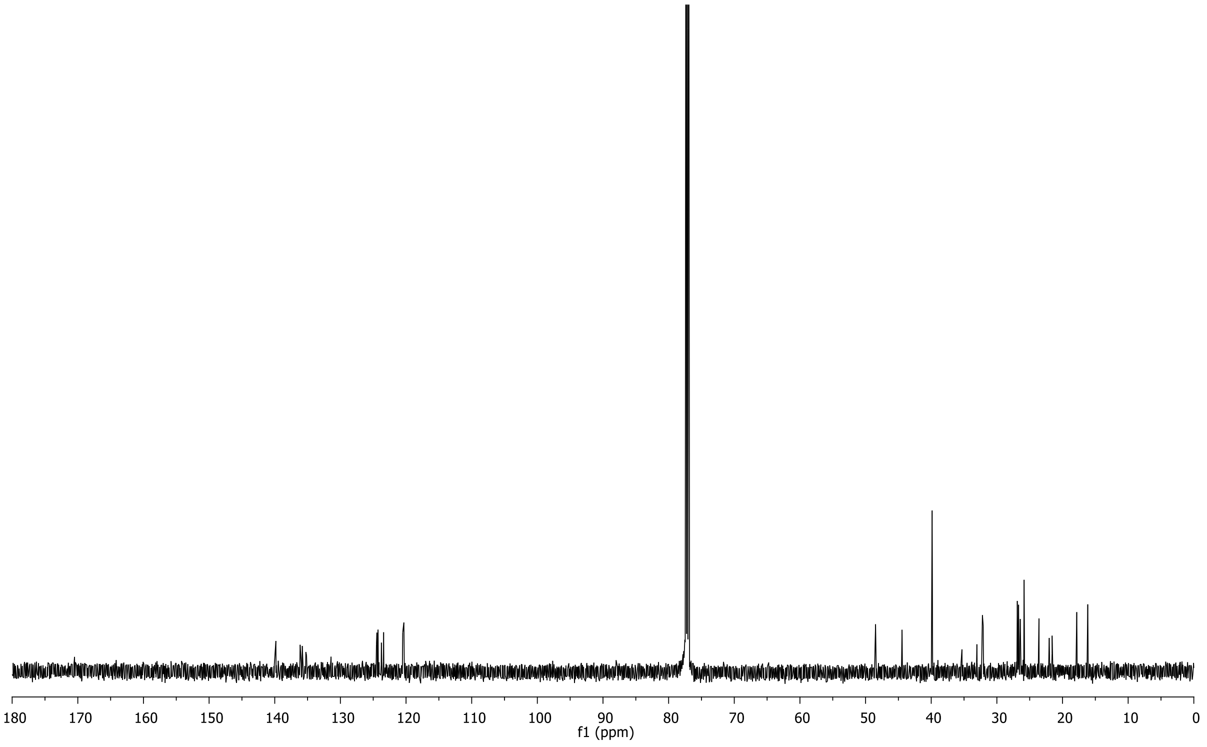

Figure S154. ^{13}C NMR spectrum (150 MHz, $CDCl_3$) of **87**.

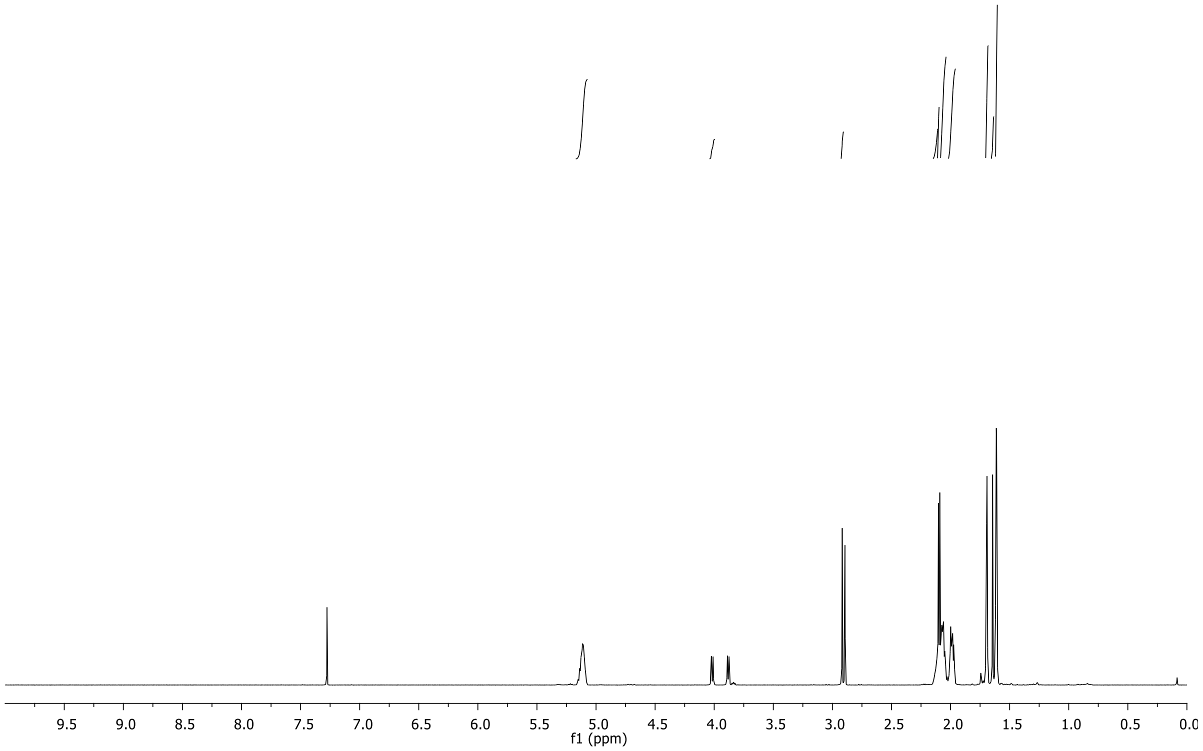

Figure S155. ^{1}H NMR spectrum (500 MHz, $CDCl_3$) of **88**.

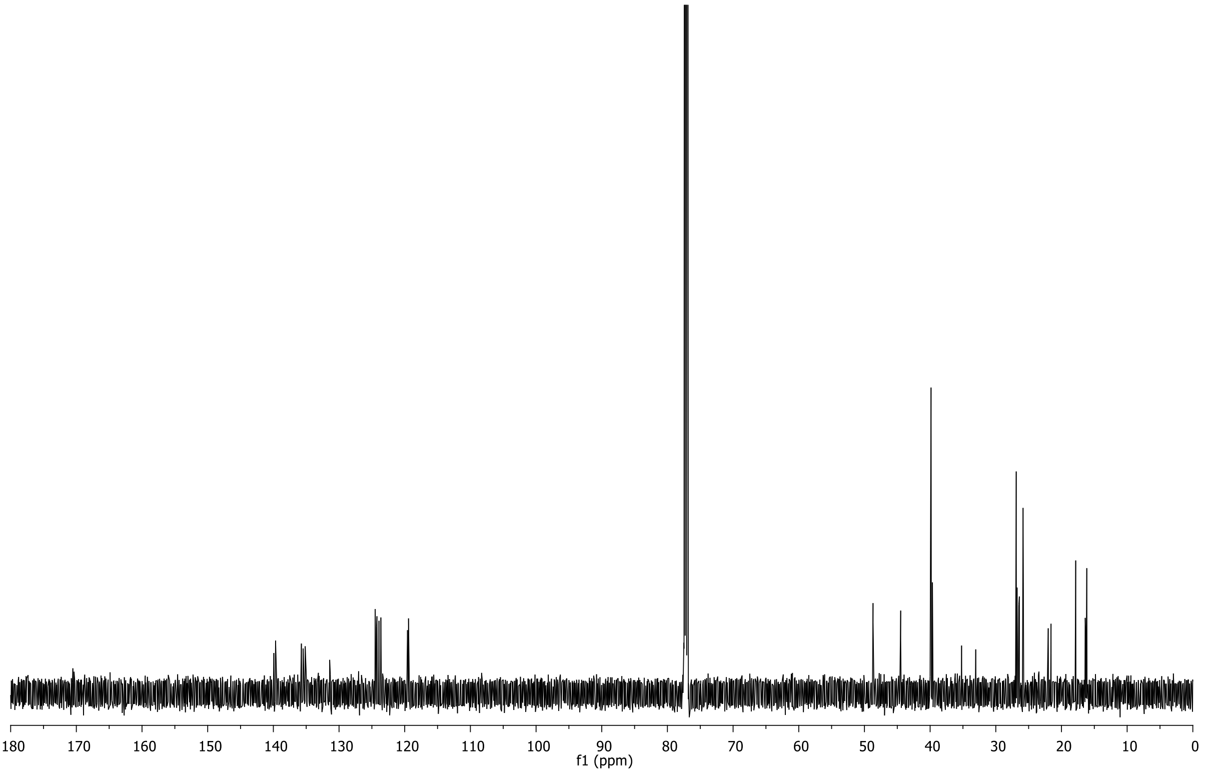

Figure S156. ^{13}C NMR spectrum (150 MHz, $CDCl_3$) of **88**.
